# Generation and application of Cu-bound alkyl nitrenes for the catalyst-controlled synthesis of cyclic β-amino acids†

**DOI:** 10.1039/d1sc01419f

**Published:** 2021-04-27

**Authors:** Raj K. Tak, Fuyuki Amemiya, Hidetoshi Noda, Masakatsu Shibasaki

**Affiliations:** Institute of Microbial Chemistry (BIKAKEN) Tokyo3-14-23 Kamiosaki, Shinagawa-ku Tokyo 141-0021 Japan hnoda@bikaken.or.jp mshibasa@bikaken.or.jp

## Abstract

The advent of saturated N-heterocycles as valuable building blocks in medicinal chemistry has led to the development of new methods to construct such nitrogen-containing cyclic frameworks. Despite the apparent strategic clarity, intramolecular C–H aminations with metallonitrenes have only sporadically been explored in this direction because of the intractability of the requisite alkyl nitrenes. Here, we report copper-catalysed intramolecular amination using an alkyl nitrene generated from substituted isoxazolidin-5-ones upon N–O bond cleavage. The copper catalysis exclusively aminates aromatic C(sp^2^)–H bonds among other potentially reactive groups, offering a solution to the chemoselectivity problem that has been troublesome with rhodium catalysis. A combined experimental and computational study suggested that the active species in the current cyclic β-amino acid synthesis is a dicopper alkyl nitrene, which follows a cyclisation pathway distinct from the analogous alkyl metallonitrene.

## Abstract 2

Copper-catalysed conditions have been developed for the chemoselective synthesis of cyclic β-amino acids.

## Graphical abstract

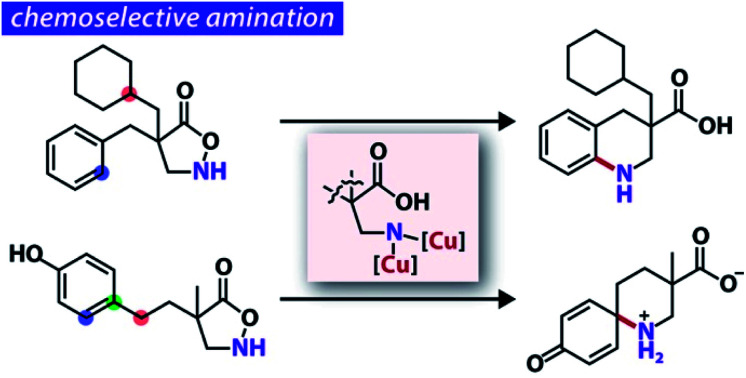

## Introduction

Catalyst-controlled transformations can streamline chemical synthesis, as they channel reactions along a desired direction among many possible pathways.^1^ Although syntheses often encounter various selectivity problems such as with enantio-, diastereo-, or regioselectivity, controlling the chemoselectivity is arguably the most difficult and has been the least developed. To achieve such feats, catalysts must be able to differentiate between multiple transition states that are energetically close but structurally less similar. Challenges associated with the development of chemoselective reactions have been documented in several review articles,^2^ and taming highly reactive intermediates represents a formidable task due to the small differences in activation energies (ΔΔ*G*^‡^).

**Scheme 1:**
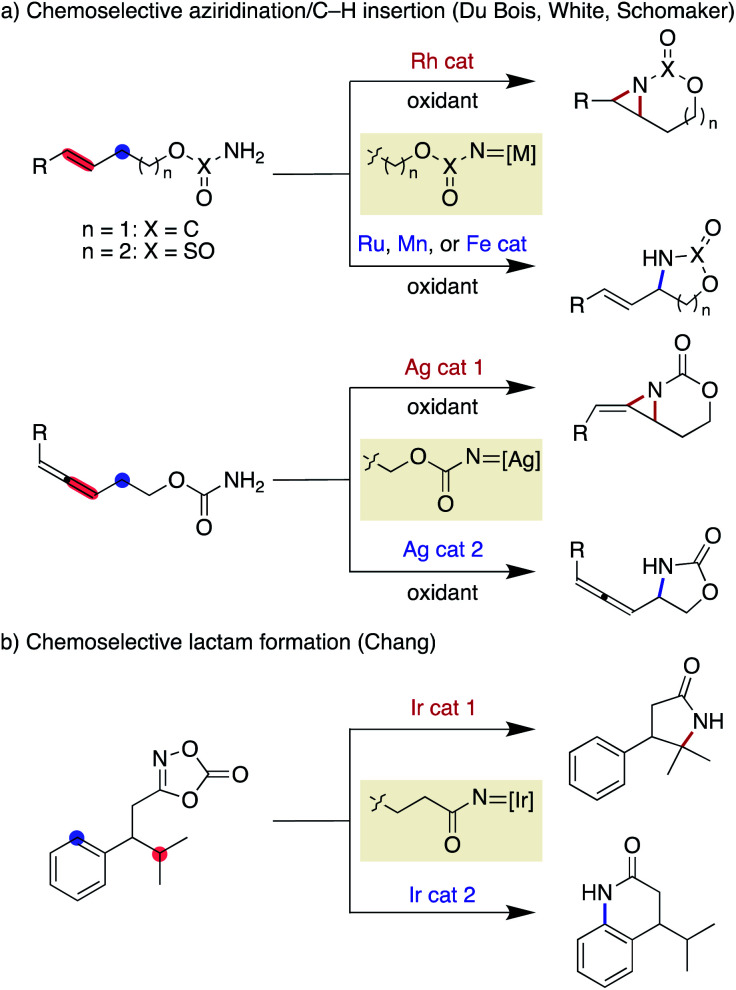
Previously reported chemoselective nitrene transfer reactions.

Nitrene is a representative reactive intermediate in organic chemistry. Among the multiple reactivities associated with nitrenes, intramolecular C–H amination offers valuable means for the reliable installation of a nitrogen atom at the preferred position in a molecule. Since the pioneering work on a metal porphyrin-catalysed cyclisation through C(sp^3^)–H amination,^3^ remarkable advances in metal nitrene chemistry have been made. Notable contributions include a series of reports from the Du Bois group, who used a rhodium dimer complex as a catalyst and carbamates or sulfamates as nitrene precursors under oxidative conditions for the synthesis of 1,2- and 1,3-aminoalcohols, respectively.^4^ Although the rhodium conditions favour aziridination over C(sp^3^)–H amination,^5^ the identification of new catalysts by the Du Bois^6^ and White^7^ groups has enabled the selective allylic C(sp^3^)–H amination. Schomaker has also reported a set of silver catalysts that allow for the chemoselective synthesis using similar carbamate substrates (Scheme 1a).^8^ Furthermore, Chang has recently documented tunable Ir catalysts that are suitable for chemoselective nitrene transfer towards C(sp^3^)–H or C(sp^2^)–H amination using 1,4,2-dioxazol-5-ones as acyl nitrene precursors,^9^ affording a range of lactam products (Scheme 1b).^10,11^

**Scheme 2:**
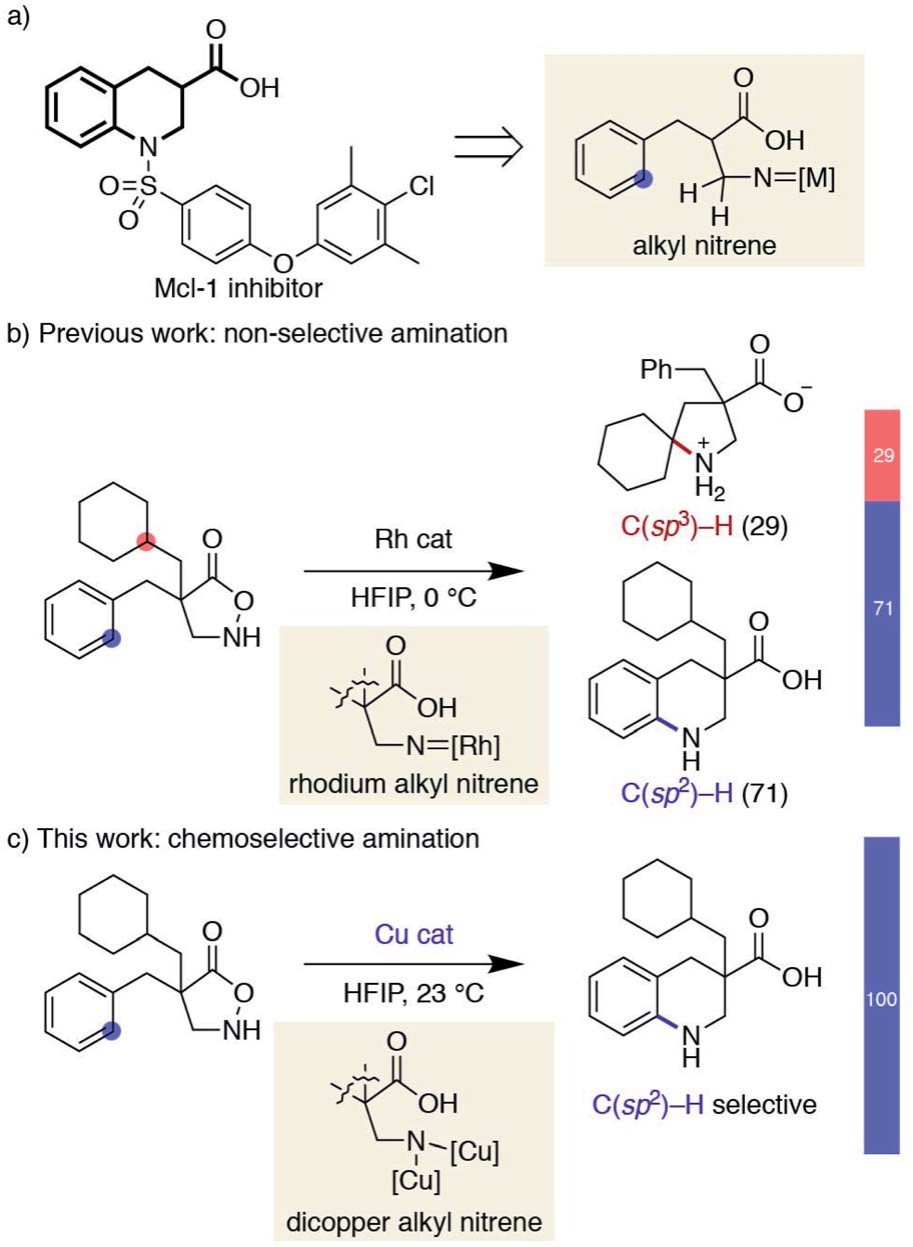
Overview of chemoselectivity challenge and this work.

Saturated N-heterocycles have recently received significant attention in drug discovery programs.^12^ Despite the reported means of constructing nitrogen-containing cyclic skeletons by the intramolecular C–H amination of nitrenes, progress in this direction has been sluggish. Although the above examples represent the state-of-the-art in nitrene transfer chemistry, their straightforward extension to the synthesis of pyrrolidines or piperidines is not trivial; alkyl nitrenes bearing one or more hydrogen atoms adjacent to the nitrogen are known to readily isomerize to the corresponding imines,^13^ and are thus reluctant to undergo productive cyclisations.^14^ As a part of our research program exploiting substituted isoxazolidin-5-ones as β-amino acid surrogates,^15,16^ we have identified that the cyclic hydroxylamines can serve as alkyl nitrene precursors through the N–O bond cleavage in the presence of a rhodium catalyst. The generated rhodium alkyl nitrene undergoes intramolecular C(sp^3^)–H insertion to afford unprotected β-proline derivatives.^17^ Furthermore, the same reactive intermediate also participates in aromatic C(sp^2^)–H amination if the substrate is suitably decorated with an aromatic ring, providing benzo-fused cyclic β-amino acids.^18^ The latter class of cyclic amino acids, tetrahydroquinoline-3-carboxylic acids, is an important building block in medicinal chemistry, as exemplified by the structures of myeloid cell leukemia-1 (Mcl-1) inhibitors,^19^ and our method offers a straightforward means to access functionalized building blocks otherwise difficult to obtain (Scheme 2a).^20^ Given the difficulty of accessing compounds bearing a quaternary carbon at the α-position of the carboxylic acid by catalytic hydrogenation, we attempted to expand the scope of the Rh-catalysed reaction. The similar kinetics between the two competitive C–H amination pathways, however, led to a mixture of products, limiting the synthetic utility of the Rh-catalysed protocol (Scheme 2b). In a related rhodium nitrene chemistry, Falck reported a reagent-controlled approach to combat a chemoselectivity issue between an olefin and an aromatic C–H bond, where the choice of aminating reagents governs the reaction site.^21,22^ Herein, we report a catalyst-controlled approach employing copper^23^ instead of rhodium to overcome the aforementioned chemoselectivity problem. This work, as far as we are aware, represents the first synthetic application of a copper alkyl nitrene. The copper-catalysed conditions selectively promote the desired C(sp^2^)–H amination even in the presence of reactive functionalities toward nitrenes (Scheme 2c). Our mechanistic study reveals that a subtle difference in reaction conditions toggles a distinctive cyclisation pathway of an otherwise analogous transformation, highlighting a nuanced reactivity of the underexplored reactive species.

## Results and discussion

### Screening of conditions

Inspired by the fact that different metals have often brought about notable reactivity differences in carbene and nitrene chemistry, we sought to employ different metallonitrenes to overcome the chemoselectivity problem. While our preliminary attempts to use various metals, including Co, Ru, Pd, Ag, and Ir, failed to exhibit any catalytic activity toward substrate **1a**, the addition of a copper salt converted **1a** to the desired cyclic compound, albeit in low yield. This promising result, together with the generally less proficient C(sp^3^)–H insertion of copper carbenes,^24^ led us to further optimise the copper-catalysed conditions. Table 1 summarizes our efforts using **1a** as a model substate. The choice of ligand on copper proved crucial in promoting the desired cyclisation and suppressing decomposition. Popular nitrogen-based ligands, such as bipyridine and phenanthroline, did not deliver product **2a** effectively (entries 1 and 2, Table 1. Also see the ESI† for further details). Salen ligand **L3** exhibited a higher reactivity, while **L4**, a hybrid ligand of pyridine and benzoxazole, considerably improved the yield (entries 3 and 4). Increasing the reaction temperature from 0 to 23 °C significantly accelerated the reaction (entry 5), whereas modulating the electronic factors on the pyridine ring did not provide beneficial effects (entries 6–8). Of note, the use of HFIP is essential to attain the desired reactivity, while cyclisation did not occur even in the similarly utilised fluorinated alcohol TFE, let alone in other non-fluorinated solvents such as MeOH, THF, and CH_2_Cl_2_ (entries 9–12). The result is in stark contrast to the previous Rh-catalysed reaction, where the preferred solvent was also HFIP but the reaction still proceeded in TFE at a slower rate. Changing the counter anion of copper resulted in a diminished yield, and employing Cu(ii) instead of Cu(i) was similarly less effective (entries 13 and 14). No background reaction was observed in the absence of an added copper salt (entry 15). The use of an unprotected N–H substrate was mandatory to achieve the desired reactivity (entry 16).

**Table 1:** Screening of conditions^a^

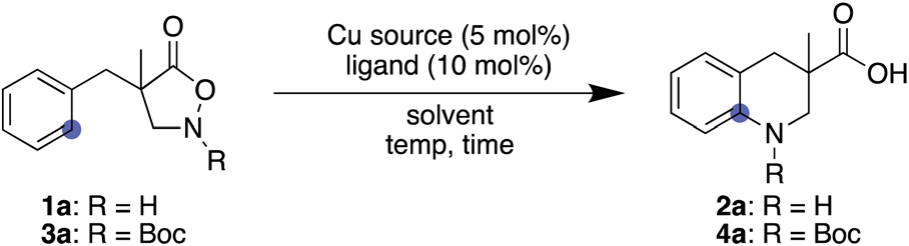							
Entry	Substrate	Cu source	Ligand	Solvent	Temp (°C)	Time (h)	Yield (%)^b^
1	**1a**	CuOTf·0.5C_6_H_6_	**L1**	HFIP	0	72	<5
2	**1a**	CuOTf·0.5C_6_H_6_	**L2**	HFIP	0	72	<5
3	**1a**	CuOTf·0.5C_6_H_6_	**L3**	HFIP	0 to 23	24 + 16	28
4	**1a**	CuOTf·0.5C_6_H_6_	**L4**	HFIP	0	56	56
5	**1a**	CuOTf·0.5C_6_H_6_	**L4**	HFIP	23	12	98
6	**1a**	CuOTf·0.5C_6_H_6_	**L5**	HFIP	23	14	98
7	**1a**	CuOTf·0.5C_6_H_6_	**L6**	HFIP	23	12	84
8	**1a**	CuOTf·0.5C_6_H_6_	**L7**	HFIP	23	24	52
9	**1a**	CuOTf·0.5C_6_H_6_	**L4**	TFE	23	36	0
10	**1a**	CuOTf·0.5C_6_H_6_	**L4**	MeOH	23	36	0
11	**1a**	CuOTf·0.5C_6_H_6_	**L4**	THF	23	36	0
12	**1a**	CuOTf·0.5C_6_H_6_	**L4**	CH_2_Cl_2_	23	36	0
13	**1a**	[Cu(CH_3_CN)_4_]PF_6_	**L4**	HFIP	23	12	79
14	**1a**	Cu(OTf)_2_	**L4**	HFIP	23	24	55
15	**1a**	—	—	HFIP	23	36	0
16	**3a**	CuOTf·0.5C_6_H_6_	**L4**	HFIP	23	36	0
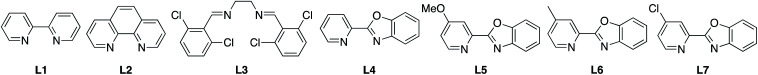							

a **1a** (0.1 mmol), 0.1 M.

b Yields were determined by ^1^H NMR analysis of the unpurified reaction mixture.

### Scope and limitations of Cu(i)-catalysed cyclic β-amino acid synthesis

With ligand **L4** identified as suitable for the desired C(sp^2^)–H amination, the scope and limitations of the β-amino acid synthesis were examined (Table 2). Cyclisation at the aromatic ring proceeded smoothly in the presence of various α-substituents (entries 1–9). It is noteworthy that no C–H insertion was observed into the methylene (**1b**), benzylic (**1c**), or even methine (**1d**) C–H bonds, which previously competed with the desired aromatic amination. A range of potentially detrimental π-bonds at the α-position such as an alkene (**1g**), a nitrile (**1h**), and an alkyne (**1i**) did not interfere with the catalysis, selectively forming tetrahydroquinoline cores. For a substrate having a propargyl moiety, further lactonization took place following the aromatic amination, affording **5** in good yield. In this study, we used 2 equiv. of the low-molecular-weight ligand with respect to the metal to ensure that sufficient amounts of **L4** were available for the copper on a 0.1 mmol scale. A comparable result was obtained with 1.2 equiv. of **L4** on a larger scale, suggesting that a 1 : 1 complex is likely responsible for the cyclisation (**2a**). Regarding the substituent effect on the aromatic ring, the copper catalysis was found to be relatively sensitive to its electronic nature. While good yields were obtained with methyl substituted substrates (entries 10 and 11), the desired reactions were sluggish with substrates bearing a halogen atom instead of a methyl group at the same position (*para* or *ortho*), and unfavourable decomposition was again observed for these electron-deficient substrates (entries 12 and 13). This reactivity trend is consistent with the involvement of electrophilic nitrogen species. All cyclisations appeared to occur from the *ortho* carbon, and *meta*-substituted substrates formed a mixture of regioisomers with varied ratios (80 : 20–40 : 60), depending on the substituent structure (entries 14–16). For methyl and fluoro substituents, cyclisation proceeded in favour of the sterically less demanding position. The reversed selectivity of 3-MeO substrate **1o** is not clear at the stage of this investigation. Protected aldehyde or alkyne containing substrates also provided the corresponding products (entries 17 and 18). Although the main focus of the current work was to broaden the α-substituent scope from the previous rhodium system, a substrate lacking an α-substituent worked equally well under the copper conditions (entry 19). We also found that the addition of 1 equiv. of TFA tended to make the reaction cleaner by reducing the rate of undesired decomposition (**2l**). Furthermore, **2p** was obtained with similar regioisomer ratios with or without added TFA, indicating that the structure of the electrophilic nitrogen species in the C–N bond forming step is likely comparable in both cases. TFA alone did not induce a detectable background reaction. The current conditions were also suitable for the cyclisation of 3-substituted isoxazolidin-5-one to afford **7** (eqn (1)). Several of the cyclic amino acids shown in Table 2 were previously not accessible in an efficient manner due to competitive undesired reactions, highlighting the benefit of the current copper catalysis.
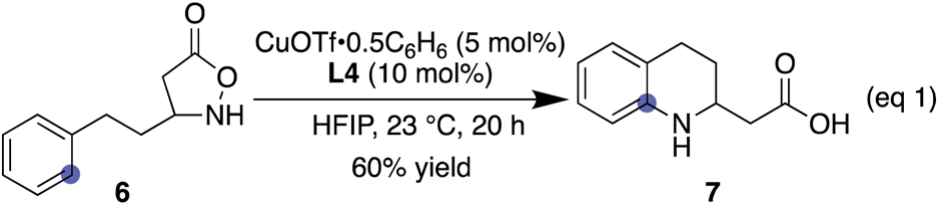


**Table 2:** Substrate scope for the synthesis of benzo-fused cyclic β-amino acids^a^

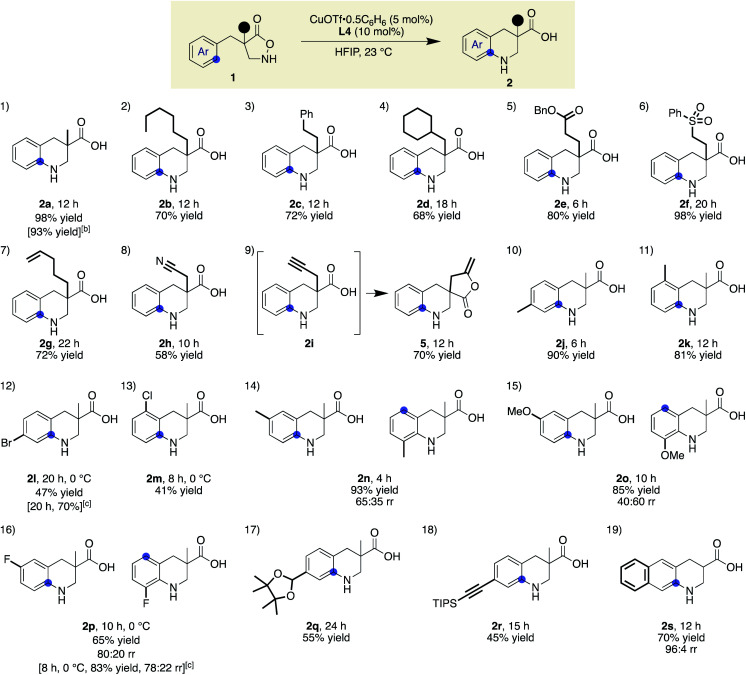

a **1** (0.1 mmol), CuOTf·0.5C_6_H_6_ (5 mol%), **L4** (10 mol%), HFIP (0.1 M), 23 °C.

b **1a**(1.0 mmol), CuOTf·0.5C_6_H_6_ (5 mol%), **L4** (6 mol%).

c TFA (1 equiv.) was added.

### Copper-catalysed asymmetric desymmetrization

**Scheme 3:**
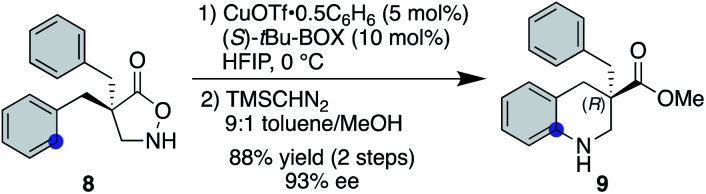
Cu-catalysed asymmetric desymmetrization.

Another advantage of the copper catalysis over rhodium was observed in the asymmetric desymmetrization of **8**—another class of catalyst-controlled synthesis. While well-established chiral dirhodium catalysts provided only marginal enantioselectivity for this transformation (see the ESI† for details), copper(i) salts ligated with a series of chiral ligands offered significantly higher enantioselectivities. Among those examined, *t*Bu-BOX ligand exhibited the best selectivity, affording the corresponding product in 93% ee in favour of the (*R*)-enantiomer (Scheme 3).^25^ With respect to the nature of the active copper species, a positive nonlinear effect was observed when ees of the employed ligand were varied. In some reported cases,^26^ the origin of nonlinear effects was ascribed to the formation of a stable racemic complex, where the minor enantiomer of the ligand is arrested in the stable, inactive hetero complex and thus a smaller amount of enantiopure, active complex is available for the catalysis. Given that similar reaction rates were observed throughout the range of ligand ees, the active catalyst concentration appeared to be constant for all cases, and thus the current system seems not to follow this scenario.

### Role of the copper catalyst and nature of the active species

The results obtained through the substrate scope study indicated the involvement of an electrophilic nitrogen intermediate. We postulated three scenarios for the role of the copper catalyst. The first is a simple Lewis acid catalysis to enhance the electrophilicity of the substrate through coordination to the carbonyl oxygen or the nitrogen atom. The second mechanism involves a nitrogen-centred radical upon the homolytic cleavage of the N–O bond. In this scenario, copper acts as an electron shuttle between Cu(i) and Cu(ii) akin to the case involving Fe(ii)/Fe(iii) found in the literature employing a hydroxylamine-derived aminating reagent.^27^ The last mechanism is based on a provisional copper-bound alkyl nitrene that undergoes downstream transformations.

The results obtained through our optimization studies show that noncoordinating solvents such as CH_2_Cl_2_ and toluene are universally ineffective for the current system. The expected enhanced Lewis acidity of copper by modulation of the ligand structure did not lead to a higher reactivity. Furthermore, the use of less coordinative anions, for example, PF_6_, did not improve the reactivity. An N-Boc substrate, which is presumably more electrophilic than the corresponding N–H substrate, did not participate in the reaction (entry 16, Table 1). These observations collectively disfavour the simple Lewis acid catalysis mechanism for the current system.

**Scheme 4:**
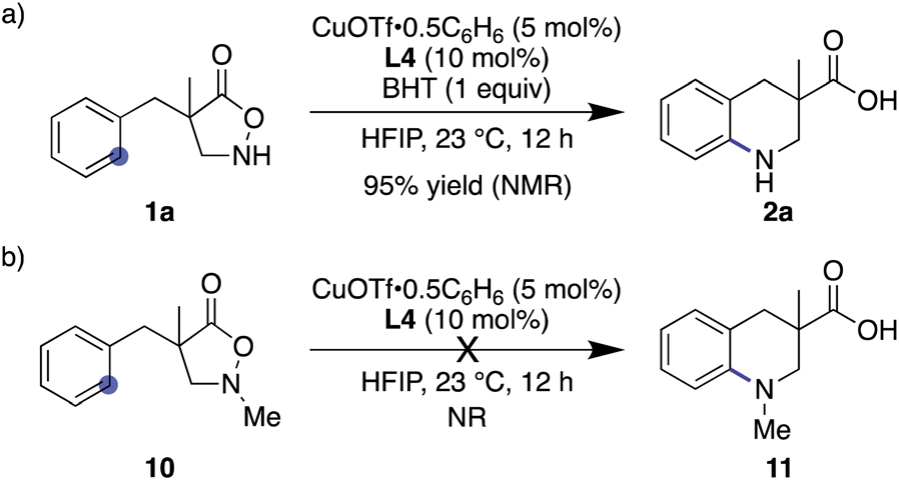
Control experiments that disfavour the nitrogen-centred radical pathway.

Given the ample literature precedents, especially with regard to enhanced reactivity in HFIP,^28^ the nitrogen-centred radical pathway appears rather feasible. Nevertheless, this mechanism is likely not operative in the current system because (1) the addition of 1 equiv. of BHT did not affect the reaction outcome at all (Scheme 4a), (2) catalyst-controlled synthesis was possible in catalytic asymmetric desymmetrization (*vide supra*), and (3) N–Me substrate **10** exhibited no reactivity and remained unchanged under otherwise identical conditions (Scheme 4b). These results rule out, at least, the nitrogen-centred naked radical pathway.

**Fig. 1:**
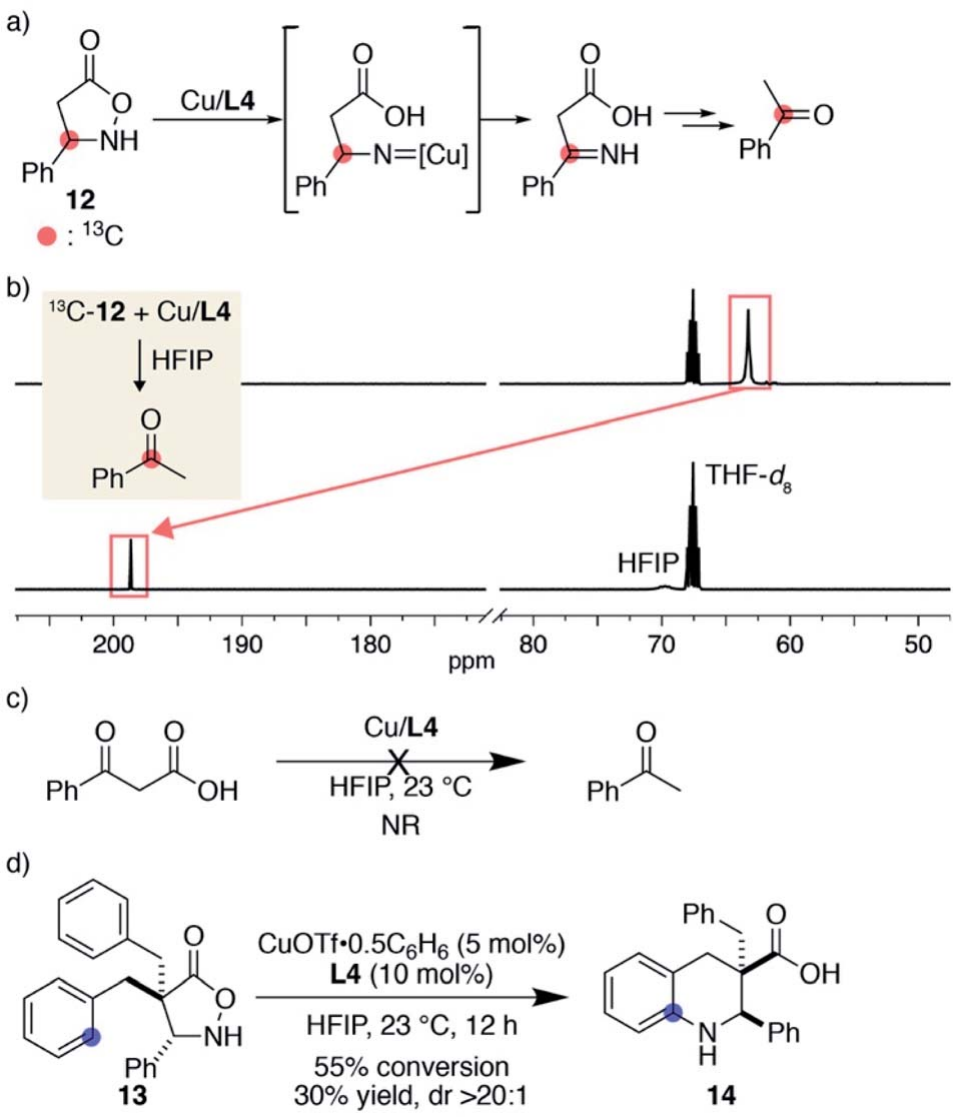
Mechanistic support for the nitrene formation. (a) Structure of designed substrate **12** and its expected decomposition pathway to acetophenone. (b) Stacked ^13^C NMR spectra. (c) A control experiment to evaluate the reactivity of benzoylacetic acid under the influence of the copper catalyst. (d) Cyclic β-amino acid formation from **13**.

During the substrate scope study, we did not observe detectable products derived from tentative nitrene decomposition, such as imines or hydrolysed ketones. To verify the formation of a nitrene, we synthesized substrate **12** bearing a phenyl group on the same carbon as the nitrogen atom. The designed substrate was expected to facilitate the isomerization to the imine if the corresponding nitrene was formed (Fig. 1a). When ^13^C-labelled substrate **12** was treated with 1 equiv. of Cu/**L4** in HFIP, **12** was immediately consumed. The ^13^C NMR spectrum of the solution in 1 : 1 THF-*d*_8_/HFIP displayed a major peak at 198.6 ppm, which was shifted from that of the substrate at 63.2 ppm (Fig. 1b). The major product was isolated from the crude sample and identified as ^13^C-labelled acetophenone. An HRMS analysis of the unpurified solution also supported the existence of acetophenone. A separate experiment revealed that benzoylacetic acid did not undergo decarboxylation and remained unchanged in the presence of Cu/**L4** in HFIP (Fig. 1c). Collectively, the formation of the labelled ketone likely reflects the generation of an alkyl nitrene, which isomerizes to the imine, followed by decarboxylation and hydrolysis to form acetophenone. Importantly, a productive 6-membered ring formation was possible with the designed substrate once reactive benzyl appendages were installed at the α-carbonyl carbon, suggesting that the nitrene is a viable intermediate rather than an irrelevant one (Fig. 1d). Given the comparison of the conversions and product yields, the amination and decomposition pathways likely occur at a similar rate. The observed high diastereoselectivity also indicates that the cyclisation took place from the opposite direction of the added aromatic ring.

### Regioselectivity consideration: two possible cyclisation pathways

**Scheme 5:**
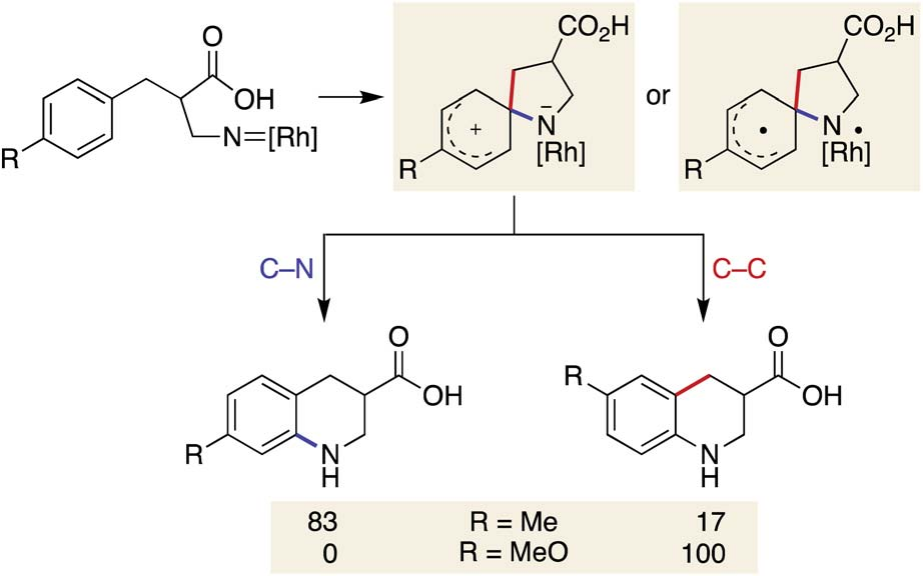
Cyclisation pathway of previous Rh-catalysed reaction.

**Scheme 6:**
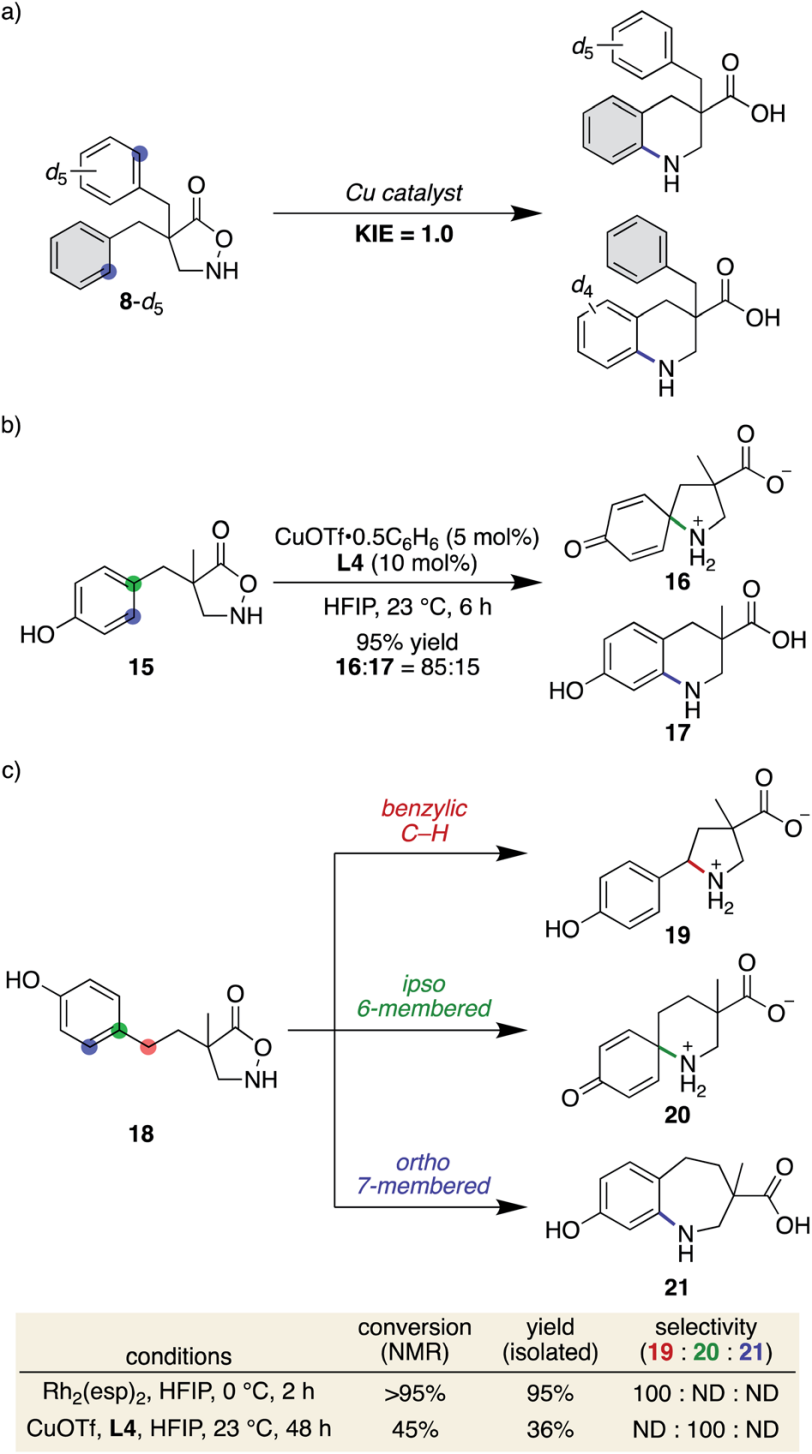
Mechanistic insights for the cyclisation event.

Our previous study on the Rh-catalysed intramolecular C(sp^2^)–H amination experimentally revealed that the cyclisation comprises two steps: spirocycle formation from the *ipso* carbon and subsequent C–N or C–C bond migration.^29^ The migration aptitude is determined by the nature of the substituents: C–N migration is the major pathway for 4-Me, whereas only C–C migration is observed for 4-MeO (Scheme 5). In stark contrast, the current catalytic system did not form any migrated regioisomers, as shown in Table 2, and instead leaned toward the direct 6-membered ring formation mechanism from the *ortho* carbon rather than the two-step mechanism. In addition, the lack of kinetic isotope effects with **8**-*d*_5_ disfavoured an archetypal C–H insertion-type transition state for direct cyclisation with the copper nitrene (Scheme 6a).^30^ Nevertheless, an exclusive C–N bond migration following the *ipso* cyclisation was not completely ruled out. To obtain further insights, we employed 4-OH substituted substrate **15**, which would stabilize and trap a spiro intermediate by isomerization to the corresponding dienone if it were to form under these conditions.^31^ The outcome was rather unexpected and distinct from that of the Rh catalysis (Scheme 6b). Both spirocycle **16** and 6-membered ring **17** were formed in excellent combined yields. A similar trend was observed with homologated substrate **18**, which presents three possible reaction sites. With **18**, a distinct chemoselectivity of the copper catalysis from the rhodium one was again manifested (Scheme 6c). Whereas the rhodium catalyst exclusively formed pyrrolidine **19** through a benzylic C–H insertion, the copper catalyst selectively afforded spirocycle **20**, presumably due to much slower kinetics for the 7-membered ring formation. These data indicate that the two cyclisation pathways from an aromatic ring are both energetically accessible under the copper-catalysed conditions in favour of the formation of the spiro intermediate, given the assumption that the reaction is kinetically controlled.

### Computational investigations

The experimental results discussed above supported the involvement of a copper alkyl nitrene in the catalytic cycle, but did not reach a conclusion regarding the subsequent reaction pathway from the reactive intermediate. Hence, a computational investigation was performed at the DFT level^32^ to gain further insight into the energetic landscape of the current system. In accordance with previous computational studies on nitrene chemistry, a triplet nitrene is more stable than the singlet at the DFT level, and thus only the triplet potential energy surface was extensively explored in this study.^33^

**Fig. 2:**
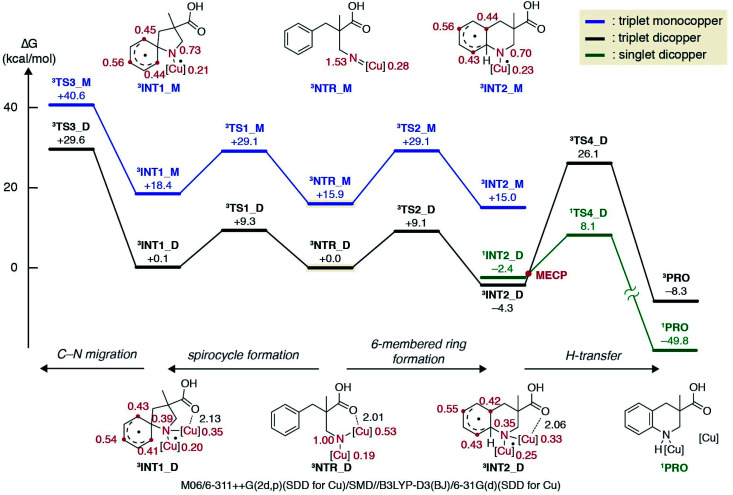
Calculated free energies for spiro formation and direct 6-membered ring formation at the DFT level. The numbers next to the structures in black and red denote bond lengths (Å) and Mulliken spin densities, respectively.

**Fig. 3:**
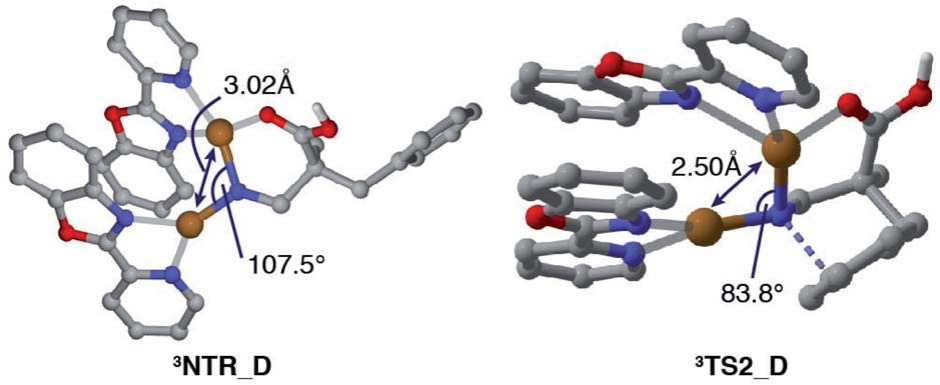
Calculated structures of **3NTR_D** and **3TS2_D**.

Fig. 2 displays a free energy profile for spirocycle formation and direct 6-membered ring formation from triplet nitrenes **3NTR**. The general trend is in line with the experimental observations and shows that both pathways are energetically accessible (**3TS1***vs.***3TS2**). Furthermore, the computations revealed that the activation energy for the subsequent C–N bond migration from spiro intermediate **3INT1** is significantly high (**3TS3**), and thus the spiro intermediate must return to the nitrene unless it has another low-energy lying pathway to follow, such as isomerization to the dienone. Therefore, the Curtin–Hammett scenario is consistent with the observed regioisomer formation. With respect to the structure of the interim copper nitrene,^34^ the observed positive non-linear effect in the asymmetric desymmetrization (*vide supra*) may suggest the involvement of two copper complexes in the C–N bond forming step. Our kinetics study under synthetically relevant conditions also suggested that more than one copper complexes were involved in the catalytic cycle (see the ESI† for details). Based on the structures of reported dinuclear copper nitrenes,^35,36^ we computed an analogous pathway involving a tentative dicopper nitrene (black line, Fig. 2). The energy landscape associated with the dicopper complex qualitatively reproduces that of the monocopper complex (blue line). Furthermore, the calculated free energies of all intermediates and transition states are universally lower for the dicopper nitrene, suggesting that a dinuclear copper complex is possibly responsible for the C–N bond formation. Close inspection of the nitrene and transition states of the dicopper complex reveals that, in addition to the delocalized spin density, they are stabilized by additional interactions that are absent in the monocopper complex: they include (1) an interaction between the carboxyl oxygen and second copper atom and (2) attractive noncovalent interactions between the copper complexes such as π-interaction of the ligands. The latter factor also contributes to the lower activation energies for cyclisations (**3TS1_M***vs.***3TS1_D**, **3TS2_M***vs.***3TS2_D**). For instance, the angle of Cu–N–Cu changes from 107.5° in **3NTR_D** to 83.8° in **3TS2_D**, resulting in a closer contact between the copper complexes and leading to a stronger attractive interaction (Fig. 3). Counterpoise corrected energy using the truncated structures corroborated the higher interaction energy. The existence of a minimum energy crossing point (MECP) between the singlet and the triplet energy surfaces^37^ allows for the formation of singlet copper-bound 6-membered ring product **1PRO***via***1TS4_D**. The interaction energy of the substrate with Cu/**L4** is higher than that of the product, preventing product inhibition and thus facilitating the closing of the catalytic cycle. Therefore, in contrast to the reported example involving an isolated dicopper nitrene, where a dissociated monocopper complex is responsible for intermolecular C(sp^3^)–H amination,^35*c*^ a dicopper nitrene seems likely to be responsible for the intramolecular C(sp^2^)–H amination in the current system.

## Conclusions

We have developed copper-catalysed conditions for the chemoselective synthesis of cyclic β-amino acids from substituted isoxazolidin-5-ones. This work represents the first synthetic application of a copper-bound alkyl nitrene. The proposed copper catalysis allows for the selective amination of aromatic C(sp^2^)–H bonds, even in the presence of potentially detrimental functionalities including double and triple bonds. The advantage of copper catalysis was also evidenced by catalytic asymmetric desymmetrization. A combined experimental and computational investigation suggested that a dicopper nitrene seems likely to be responsible for the key C–N bond forming step. Our mechanistic study also reveals that switching the metal from rhodium to copper triggers a distinctive pathway in the 6-membered ring forming amination, showcasing a nuanced nature of the underexplored reactive intermediate. The development of other catalyst-controlled reactions involving alkyl nitrenes is currently underway in our laboratory.

## Author contributions

H. N. and M. S. conceived the project. R. K. T., F. A., and H. N. conducted experiments and analysed data. H. N. performed the density functional theory calculations. H. N. and M. S. wrote the manuscript with help from all authors.

## Conflicts of interest

There are no conflicts to declare.

## Supplementary Material

SC-012-D1SC01419F-s001

SC-012-D1SC01419F-s002
