# Supplementary material for: Generation and application of Cu-bound alkyl nitrenes for the catalyst-controlled synthesis of cyclic β-amino acids
Source: Chem Sci. 2021 Apr 27;12(22):7809–17. doi: 10.1039/d1sc01419f (PMC8188474; doi:10.1039/d1sc01419f)

Electronic Supplementary Material (ESI) for Chemical Science.
This journal is © The Royal Society of Chemistry 2021

Generation and application of Cu-bound alkyl nitrene for the catalyst-controlled synthesis of cyclic β-amino acids

Raj K. Tak,‡ Fuyuki Amemiya,‡ Hidetoshi Noda,* and Masakatsu Shibasaki*

Institute of Microbial Chemistry (BIKAKEN), Tokyo, Japan

Email: hnoda@bikaken.or.jp; mshibasa@bikaken.or.jp

‡These authors equally contributed to this work.

Table of Contents

1. General information S2
   1-1. Reactions and purifications S2
   1-2. Characterizations S2
   1-3. Solvents and reagents S2
2. Synthesis of substituted isoxazolidin-5-ones S3
   2-1. Structure of substrate precursors S3
   2-2. Substrate synthesis S3
3. Initial ligand screening S16
4. Synthesis of cyclic β-amino acid S17
   4-1. Scope of benzo-fused β-amino acid synthesis S17
   4-2. Mechanistic investigation S23
   4-3. Kinetic study S24
5. Catalytic asymmetric desymmetrization of **8** S26
   5-1. Catalyst screening S26
   5-2. Copper catalyzed asymmetric desymmetrization S27
   5-3. Nonlinear effect S29
6. Computational study S30
   6-1. Computational details S30
   6-2. Evaluation of interaction energies S30
7. References S31
8. Optimized coordinates S32
9. NMR spectra S48

1. General information

1-1. Reactions and purifications

Unless otherwise noted, all reactions were carried out under an argon atmosphere and were stirred with Teflon-coated magnetically stirred bars. All work-up and purification procedures were carried out with reagent-grade solvents under ambient atmosphere. Thin layer chromatography (TLC) was performed on Merck TLC plates (0.25 mm) pre-coated with silica gel 60 F254 and visualized by UV quenching and staining with ninhydrin or $KMnO_4$. Flash column chromatography was performed on a Biotage Isolera Spektra One.

1-2. Characterizations

Infrared (IR) spectra were recorded on a HORIBA FT210 Fourier transform infrared spectrophotometer. NMR spectra were recorded on a Bruker AVANCE III HD400 NMR spectrometers at 298K. Chemical shifts (δ) are given in ppm relative to residual solvent peaks.[1] Data for ^{1}H NMR are reported as follows: chemical shift (multiplicity, coupling constants where applicable, number of hydrogens). Abbreviations are as follows: s (singlet), d (doublet), t (triplet), dd (doublet of doublet), dt (doublet of triplet), ddd (doublet of doublet of doublet), q (quartet), m (multiplet), br (broad). Optical rotation was measured using a 1.0 mL cell with a 1.0 dm path length on a JASCO polarimeter P-1030. Single-crystal X-ray data were collected on a Rigaku R-AXIS RAPID II imaging plate area detector with graphite-monochromated Cu-Ka radiation. Melting points were measured on a Yanagimoto Seisakusho Micro Melting Point Apparatus. High-resolution mass spectra (ESI TOF (+)) were measured on a Thermo Fisher Scientific LTQ Orbitrap XL. Normal phase HPLC analysis was conducted on a JASCO HPLC system equipped with Daicel chiral-stationary-phase column (ϕ 0.46 cm x 25 cm).

1-3. Solvents and reagents

Unless otherwise noted, materials were purchased from commercial suppliers and were used without further purification. Anhydrous MeOH were purchased from commercial suppliers. THF and CH_2Cl_2 were purified by passing through a solvent purification system (Glass Contour). TFE and **L4** were purchased from TCI Co., Ltd., and used as received. HFIP and CuOTf•0.5C_6H_6 were purchased from Sigma-Aldrich, and used as received. Benzaldehyde-d_5 was purchased from CDI Isotope.

2. Synthesis of substituted isoxazolidin-5-ones

2-1. Structure of substrate precursors

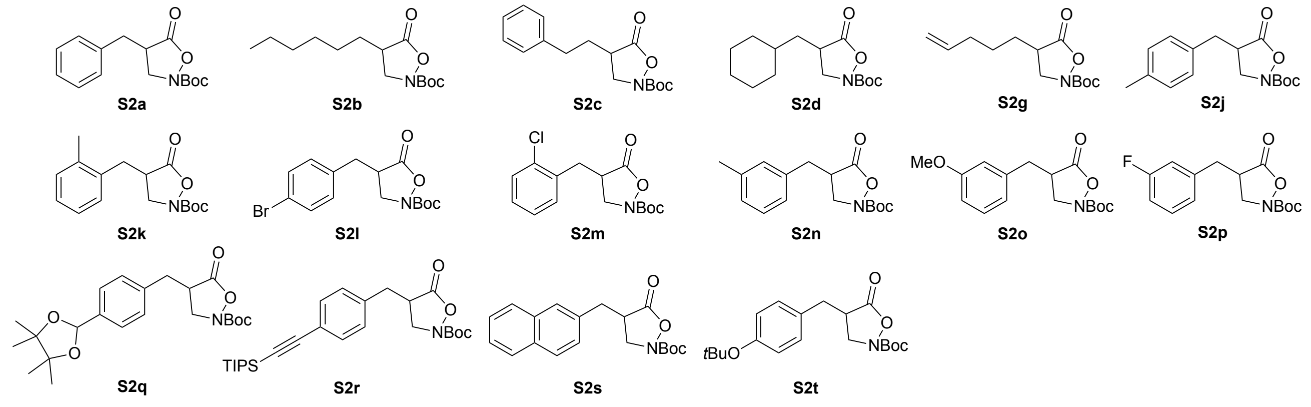

Substituted isoxazolidin-5-ones **S2a**, **S2d**, and **S2j–t** are known.[2,3]

2-2. Substrate synthesis

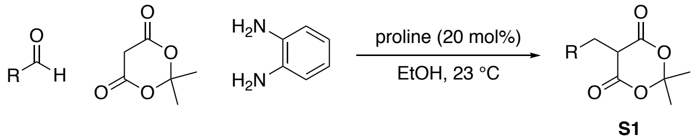

S1

General procedure A:[2] To a solution of *ortho*-phenylenediamine (1.0 equiv) in EtOH (0.05 M) at 23 °C were added aldehyde (2.0 equiv), Meldrum's acid (1.0 equiv), and (DL)-proline (20 mol%). The resulting solution was stirred for 24 h and evaporated under reduced pressure to give the residue, which was dissolved in $CHCl_3$, washed with 1M aq HCl. The resulting mixture was filtered through a pad of Celite and washed with $CHCl_3$, and the filtrates were extracted with $CHCl_3$ (3x). The combined organic layers were washed with water, dried over Na_2SO_4, filtered and concentrated in vacuo to afford crude **S1**, which was used directly in the next step without further purification.

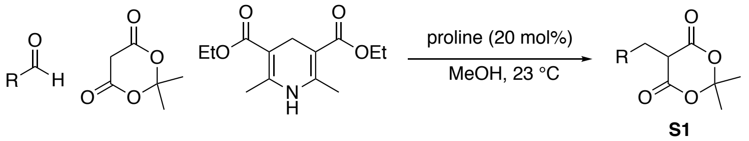

S1

General procedure B:[3] Meldrum's acid (1.0 equiv), aldehyde (1.0 equiv), (DL)-proline (20 mol%) and diethyl 2,6-dimethyl-1,4-dihydropyridine-3,5-dicarboxylate (1.0 equiv) were dissolved in MeOH (0.3 M). The solution was vigorously stirred until full conversion at room temperature, and then concentrated under reduced pressure. The obtained crude residue was purified directly by silica gel column chromatography, eluting with hexane/EtOAc to provide the corresponding Meldrum's acid derivatives **S1**.

5-(4-((*tert*-Butyldimethylsilyl)oxy)phenethyl)-2,2-dimethyl-1,3-dioxane-4,6-dione (S1u): Prepared by the general procedure B from 2-(4-((*tert* butyldimethylsilyl)oxy)phenyl)acetaldehyde[4] (4.50 g, 18.0 mmol) and isolated as a white solid (3.70 g, 55% yield). **m.p.** 92–94 °C;

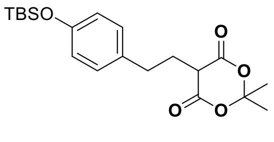

^{1}H NMR (400 MHz, $CDCl_3$): δ 7.08 – 7.05 (m, 2H), 6.78 – 6.75 (m, 2H), 3.44 (t, *J* = 5.4 Hz, 1H), 2.79 (dd, *J* = 8.6, 6.8 Hz, 2H), 2.39 – 2.34 (m, 2H), 1.74 (s, 3H), 1.71 (s, 3H), 0.97 (s, 9H), 0.17 (s, 6H); **^{13}C NMR** (101 MHz, $CDCl_3$): δ 165.5, 154.2, 132.9, 129.6, 120.2, 104.9, 44.9, 31.6, 28.5, 28.1, 26.6, 25.7, 18.2, –4.43**; IR** (thin film): 2955, 2930, 1792, 1752, 1608, 1509, 1379, 1255, 1055, 919, 838, 780 cm^{-1}; **HRMS (ESI)** *m/z* calc'd for $C_{20}H_{30}O_5NaSi$ [M + Na]$^+$: 401.1755, found: 401.1759.

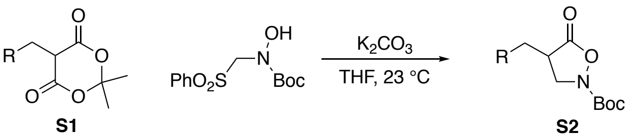

General procedure C:[2] To a solution of **S1** in THF (0.1 M) were added *tert*-butyl hydroxy((phenylsulfonyl)methyl)carbamate[2] (1.0 equiv) and K_2CO_3 (2.5 equiv) at 23 °C. After being stirred at 23 °C for 24 h, the mixture was filtered through a pad of Celite and washed with EtOAc, and the filtrates were evaporated under reduced pressure to give the crude residue, which was purified by silica gel column chromatography, eluting with hexane/EtOAc to afford **S2.**

***tert*-Butyl 4-hexyl-5-oxoisoxazolidine-2-carboxylate (S2b):** Prepared by the general procedure B and C from

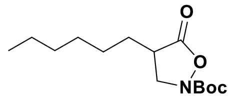

hexanal (0.85 mL, 6.9 mmol), and isolated as a colourless liquid (1.3 g, 67% yield for 2 steps). **^{1}H NMR** (400 MHz, $CDCl_3$): δ 4.27 (dd, *J* = 11.0, 8.6 Hz, 1H), 3.65 (dd, *J* = 11.0, 9.1 Hz, 1H), 2.84 (qd, *J* = 8.9, 5.2 Hz, 1H), 1.91 – 1.82 (m, 1H), 1.58 - 153 (m, 1H), 1.51 (s, 9H), 1.40 – 1.28(m, 8H), 0.88 (t, *J* = 6.8 Hz, 3H); **^{13}C NMR** (101 MHz, $CDCl_3$): δ 174.02, 155.06, 83.01, 52.53, 39.31, 30.43, 27.85, 27.77, 27.03, 26.01, 21.48, 12.97; **IR** (thin film): 2959, 2931, 1800, 1716, 1458, 1371, 1335, 1216, 1146, 847, 756 cm^{-1}; **HRMS** (ESI) *m/z* calc'd for $C_{15}H_{29}O_5NNa$ [M+Na+MeOH]$^+$: 326.1938, found: 326.1938.

***tert*-Butyl 5-oxo-4-phenethylisoxazolidine-2-carboxylate (S2c):** Prepared by the general procedure A and C

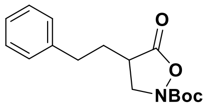

from 2-phenylacetaldehyde (2.5 g, 21.3 mmol), and isolated as a pale yellow liquid (65% yield for 2 steps).**^{1}H NMR** (400 MHz, $CDCl_3$): δ 7.33 – 7.29 (m, 2H), 7.24 – 7.17 (m, 3H), 4.22 (dd, *J* = 11.0, 8.6 Hz, 1H), 3.62 (dd, *J* = 11.0, 9.4 Hz, 1H), 2.86 – 2.70 (m, 3H), 2.28 – 2.17 (m, 1H), 1.91 – 1.81 (m, 1H), 1.51 (s, 9H); **^{13}C NMR** (101 MHz, $CDCl_3$): δ 174.80, 155.98, 139.86, 129.01, 128.72, 128.40, 126.58, 84.14, 53.59, 39.48, 33.03, 30.43, 28.04; **IR** (thin film): 2981, 2934, 1799, 1718, 1455, 1371, 1336, 1142, 847, 756 cm^{-1}; **HRMS** (ESI) *m/z* calc'd for $C_{17}H_{25}O_5NNa$ [M+Na+MeOH]$^+$: 346.1625, found: 346.1628.

***tert*-Butyl 5-oxo-4-(pent-4-en-1-yl)isoxazolidine-2-carboxylate (S2g):** Prepared by the general procedure A

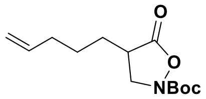

and C from pent-4-enal (2.0 g, 20.2 mmol), and isolated as a pale yellow liquid (63% yield for 2 steps). **^{1}H NMR** (400 MHz, $CDCl_3$): δ 5.76 (ddt, *J* = 16.9, 10.2, 6.7 Hz, 1H), 5.04 – 4.97 (m, 2H), 4.28 (dd, *J* = 11.0, 8.6 Hz, 1H), 3.65 (dd, *J* = 11.0, 9.2 Hz, 1H), 2.89 – 2.81 (m,

1H), 2.12 – 2.06 (m, 2H), 1.92 – 1.82 (m, 1H), 1.60 – 1.53 (m, 2H), 1.51 (s, 9H), 1.49 – 1.44 (m, 1H); **^{13}C NMR** (101 MHz, $CDCl_3$): δ 174.87, 156.05, 137.48, 115.51, 84.09, 53.55, 40.22, 33.23, 28.20, 28.05, 26.25; **IR** (thin film): 3031, 2983, 2935, 1801, 1716, 1458, 1371, 1336, 1216, 1145, 916, 847, 757 cm^{-1}; **HRMS** (ESI) *m/z* calc'd for $C_{14}H_{25}O_5NNa$ [M+Na+MeOH]$^+$: 310.1625, found: 310.1628.

***tert*-Butyl 4-(4-((*tert*-butyldimethylsilyl)oxy)phenethyl)-5-oxoisoxazolidine-2-carboxylate (S2u):** Prepared

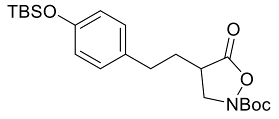

by the general procedure A from **S1u** (2.70 g, 7.14 mmol) and isolated as a colorless liquid (2.70 g, 90% yield). **^{1}H NMR** (400 MHz, $CDCl_3$): δ 7.04 – 7.01 (m, 2H), 6.78 – 6.75 (m, 2H), 4.22 (dd, J = 11.0, 8.6 Hz, 1H), 3.62 (dd, J = 11.0, 9.4 Hz, 1H), 2.84 – 2.75 (m, 1H), 2.73 – 2.62 (m, 2H), 2.37 – 2.15 (m, 1H), 1.86-1.76 (m, 1H), 1.51 (s, 9H), 0.97 (s, 9H), 0.18 (s, 6H); **^{13}C NMR** (101 MHz, $CDCl_3$): δ 174.9, 156.0, 154.3, 132.4, 129.3, 120.2, 84.1, 53.6, 39.4, 32.2, 30.6, 28.0, 25.7, 18.2, –4.4; **IR** (thin film): 2955, 2931, 2858, 1800, 1749, 1719, 1609, 1509, 1461, 1370, 1258, 1142, 1007, 916, 814, 781, 690 cm^{-1}; **HRMS (ESI)** *m/z* calc'd for $C_{22}H_{34}O_5NSi$ [M - H]$^-$: 420.2212, found: 420.2214.

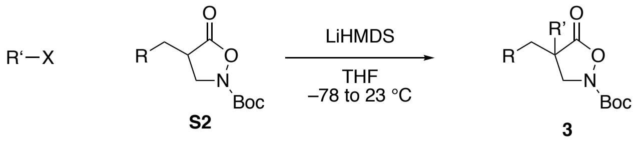

General procedure D: To a solution of **S2** (1.0 equiv) in THF (0.3 M) was slowly added LiHMDS (1M in THF, 1.6 equiv) at –78 °C and the resulting solution was stirred for 1h at –78 °C. To the mixture, alkyl halide (2 equiv) was added at the same temperature, and the solution was gradually warmed to an ambient temperature. The reaction progress was monitored by TLC, and the reaction was quenched after completion by the addition of saturated aqueous NH_4Cl solution. The aqueous phase was extracted with EtOAc (3x) and the organic phase was concentrated under reduced pressure. The obtained crude residue was purified by flash column chromatography, eluting with hexane/EtOAc to provide the **S3**.[2]

***tert*-Butyl 4-benzyl-4-methyl-5-oxoisoxazolidine-2-carboxylate (3a):** Prepared by the general procedure D

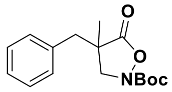

from **S2a** (1.50 g, 5.15 mmol) and methyl iodide (673 μL, 10.8 mmol), and isolated as a white solid (1.29 g, 82% yield). **m.p.** 100–102 °C; **^{1}H NMR** (400 MHz, $CDCl_3$): δ 7.15 – 7.10 (m, 3H), 6.99-6.97 (m, 2H), 3.86 (d, J = 11.2 Hz, 1H), 3.49 (d, J = 11.2 Hz, 1H), 2.79 (d, J = 13.6 Hz, 1H), 2.64 (d, J = 13.6 Hz, 1H), 1.32 (s, 9H), 1.08 (s, 3H); **^{13}C NMR** (101 MHz, $CDCl_3$): δ 177.16, 156.05, 135.06, 130.19, 128.68, 127.43, 84.03, 57.64, 45.27, 40.82, 28.06, 20.79; **IR** (thin film): 2980, 2936, 1802, 1747, 1722, 1448, 1371, 1150, 1064, 763, 698 cm^{-1}; **HRMS (ESI)** *m/z* calc'd for $C_{16}H_{21}O_4NNa$ [M + Na]$^+$: 314.1363, found: 314.1362.

***tert*-Butyl 4-benzyl-4-hexyl-5-oxoisoxazolidine-2-carboxylate (3b):** Prepared by the general procedure D from

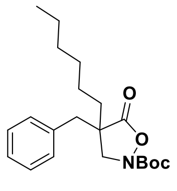

S2b (500 mg, 1.84 mmol) and benzyl bromide (438 μL, 3.68 mmol), and isolated as a white solid (450 mg, 69% yield). **m.p.** 52–54 °C; **^{1}H NMR** (400 MHz, $CDCl_3$): δ 7.31–7.26 (m, 3H), 7.17 – 7.15 (m, 2H), 3.96 (d, *J* = 11.2 Hz, 1H), 3.86 (d, *J* = 11.2 Hz, 1H), 3.05 (d, *J* = 13.6 Hz, 1H), 2.84 (d, *J* = 14.0 Hz, 1H), 1.68-1.59 (m, 2H), 1.48 (s, 9H), 1.32 – 1.27 (m, 8H), 0.89 – 0.85 (m, 3H); **^{13}C NMR** (101 MHz, $CDCl_3$): δ 176.39, 155.72, 135.23, 130.12, 128.70, 127.40, 83.76, 55.16, 49.38, 40.21, 35.18, 31.49, 29.44, 28.07, 24.01, 22.51, 13.98; **IR** (thin film): 3020, 2932, 2859, 1791, 1712, 1456, 1371, 1215, 1147, 1030, 847, 759 cm^{-1}; **HRMS** (ESI) *m/z* calc'd for $C_{21}H_{31}O_4NNa$ $[M + Na]^+$: 384.2145, found: 384.2147.

***tert*-Butyl 4-benzyl-5-oxo-4-phenethylisoxazolidine-2-carboxylate (3c):** Prepared by the general procedure D

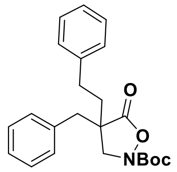

from **S2c** (400 mg, 1.37 mmol) and benzyl bromide (327 μL, 2.75 mmol), and isolated as a pale pink liquid (340 mg, 65% yield). **^{1}H NMR** (400 MHz, $CDCl_3$): δ 7.34 – 7.25 (m, 5H), 7.22-7.18 (m, 3H), 7.15 – 7.13 (m, 2H), 4.03 (d, *J* = 11.2 Hz, 1H), 3.93 (d, *J* = 11.2 Hz, 1H), 3.12 (d, *J* = 14.0 Hz, 1H), 2.93 (d, *J* = 14.0 Hz, 1H), 2.73 – 2.68 (m, 2H), 2.04-1.87 (m, 2H), 1.48 (s, 9H); **^{13}C NMR** (101 MHz, $CDCl_3$): δ 175.99, 155.73, 140.45, 134.94, 130.40, 130.12, 128.80, 128.62, 128.28, 127.53, 126.37, 84.00, 55.35, 49.41, 40.09, 36.83, 30.47, 28.06; **IR** (thin film): 3029, 2982, 2933, 1792, 1715, 1459, 1455, 1370, 1221, 1146, 1030, 847, 752 cm^{-1}; **HRMS** (ESI) *m/z* calc'd for $C_{23}H_{27}O_4NNa$ $[M + Na]^+$: 404.1832, found: 404.1831.

***tert*-Butyl 4-benzyl-5-oxo-4-(pent-4-en-1-yl)isoxazolidine-2-carboxylate (3g):** Prepared by the general

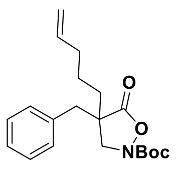

procedure D from **S2g** (700 mg, 2.74 mmol) and benzyl bromide (652 μL, 5.48 mmol), and isolated as a light brown liquid (520 mg, 55% yield). **^{1}H NMR** (400 MHz, $CDCl_3$): δ 7.33 – 7.27 (m, 3H), 7.17 – 7.15 (m, 2H), 5.75 (ddt, *J* = 16.9, 10.2, 6.7 Hz, 1H), 5.04 – 4.96 (m, 2H), 3.96 (d, *J* = 11.2 Hz, 1H), 3.85 (d, *J* = 10.8 Hz, 1H), 3.06 (d, *J* = 14.0 Hz, 1H), 2.84 (d, *J* = 14.0 Hz, 1H), 2.09 – 2.03 (m, 2H), 1.73-1.58 (m, 2H), 1.54 – 1.50 (m, 2H), 1.48 (s, 9H); **^{13}C NMR** (101 MHz, $CDCl_3$): δ 181.06, 137.63, 135.74, 129.32, 129.16, 127.80, 115.48, 55.51, 49.19, 41.52, 35.52, 33.75, 23.50; **IR** (thin film): 3030, 2980, 2936, 1796, 1716, 1456, 1370, 1147, 916, 755 cm^{-1}; **HRMS** (ESI) *m/z* calc'd for $C_{21}H_{31}O_5NNa$ $[M + Na + MeOH]^+$: 400.2094, found: 400.2098.

***tert*-Butyl 4-benzyl-4-(cyanomethyl)-5-oxoisoxazolidine-2-carboxylate (3h):** Prepared by the general

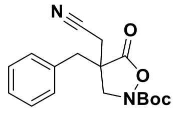

procedure D from **S2a** (500 mg, 1.80 mmol) and bromoacetonitrile (251 μL, 3.60 mmol), and isolated as a colourless liquid (239 mg, 42% yield). **^{1}H NMR** (400 MHz, $CDCl_3$): δ 7.39 – 7.33 (m, 3H), 7.22 – 7.20 (m, 2H), 4.22 (d, *J* = 11.6 Hz, 1H), 3.91 (d, *J* = 11.6 Hz, 1H), 3.08 (q, *J* = 14.1 Hz, 2H), 2.66 (q, *J* = 17.1 Hz, 2H), 1.53 (s, 9H); **^{13}C NMR** (101 MHz, $CDCl_3$): δ 172.91, 155.33, 132.87, 130.13, 129.16, 128.29, 115.29, 84.98, 55.55, 47.41, 38.94, 28.03, 22.52; **IR** (thin film): 3025, 2984, 2935, 2253, 1800, 1719, 1456, 1372, 1216, 1147, 910, 844, 771 cm^{-1}; **HRMS** (ESI) *m/z* calc'd for $C_{17}H_{21}O_4N_2$ $[M + H]^+$: 317.1496, found: 317.1494.

***tert*-Butyl 4-benzyl-5-oxo-4-(prop-2-yn-1-yl)isoxazolidine-2-carboxylate (3i):** Prepared by the general

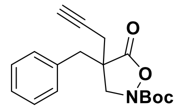

procedure D from **S2a** (400 mg, 1.44 mmol) and propargyl bromide (217 µL, 2.88 mmol), and isolated as a pale yellow liquid (227 mg, 50% yield). **^{1}H NMR** (400 MHz, $CDCl_3$): δ 7.35 – 7.29 (m, 3H), 7.21 – 7.18 (m, 2H), 4.06 (q, *J* = 11.3 Hz, 2H), 3.04 (dd, *J* = 37.2, 13.9 Hz, 2H), 2.54 (ddd, *J* = 50.0, 17.0, 2.7 Hz, 2H), 2.16 (t, *J* = 2.6 Hz, 1H), 1.51 (s, 9H); **^{13}C NMR** (101 MHz, $CDCl_3$): δ 174.75, 155.70, 134.30, 130.24, 128.87, 127.78, 84.19, 78.10, 72.74, 55.19, 49.06, 39.28, 28.11, 24.41; **IR** (thin film): 3308, 3019, 2984, 1796, 1716, 1371, 1215, 1147, 1078, 846, 757 cm^{-1}; **HRMS** (ESI) *m/z* calc'd for $C_{18}H_{21}O_4NNa$ $[M + Na]^+$: 338.1363, found: 338.1363.

***tert*-Butyl 4-methyl-4-(4-methylbenzyl)-5-oxoisoxazolidine-2-carboxylate (3j):** Prepared by the general

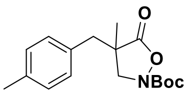

procedure D from **2j** (500 mg, 1.71 mmol) and methyl iodide (213 µL, 3.41 mmol), and isolated as a colorless liquid (408 mg, 78% yield). **^{1}H NMR** (400 MHz, $CDCl_3$): δ 7.13 (d, *J* = 7.6 Hz, 2H), 7.06 (d, *J* = 8.0 Hz, 2H), 4.06 (d, *J* = 11.2 Hz, 1H), 3.67 (d, *J* = 11.2 Hz, 1H), 2.94 (d, *J* = 14.0 Hz, 1H), 2.79 (d, *J* = 14.0 Hz, 1H), 2.33 (s, 3H), 1.51 (s, 9H), 1.27 (s, 3H); **^{13}C NMR** (101 MHz, $CDCl_3$): δ 177.26, 156.04, 137.08, 131.93, 130.06, 129.35, 83.97, 57.64, 45.30, 40.44, 28.05, 21.04, 20.78; **IR** (thin film): 3023, 2981, 2935, 1798, 1718, 1515, 1371, 1150, 1071, 847, 757 cm^{-1}; **HRMS** (ESI) *m/z* calc'd for $C_{18}H_{27}O_5NNa$ $[M + Na + MeOH]^+$: 360.1781, found: 360.1777.

***tert*-Butyl 4-methyl-4-(2-methylbenzyl)-5-oxoisoxazolidine-2-carboxylate (3k):** Prepared by the general

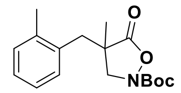

procedure D from **S2k** (400 mg, 1.37 mmol) and methyl iodide (169 µL, 2.74 mmol), and isolated as a colorless liquid (339 mg, 81% yield). **^{1}H NMR** (400 MHz, $CDCl_3$): δ 7.19 – 7.17 (m, 2H), 7.16 – 7.13 (m, 2H), 3.93 (d, *J* = 10.8 Hz, 1H), 3.78 (d, *J* = 11.2 Hz, 1H), 3.00 (s, 2H), 2.31 (s, 3H), 1.51 (s, 9H), 1.31 (s, 3H); **^{13}C NMR** (101 MHz, $CDCl_3$): δ 177.05, 155.97, 137.01, 133.77, 130.86, 130.55, 127.48, 126.31, 84.03, 57.94, 45.93, 36.96, 28.05, 21.13, 20.10; **IR** (thin film): 3022, 2981, 2936, 1795, 1718, 1457, 1371, 1216, 1149, 1072, 847, 756 cm^{-1}; **HRMS** (ESI) *m/z* calc'd for $C_{17}H_{23}O_4NNa$ $[M + Na]^+$: 328.1519, found: 328.1523.

***tert*-Butyl 4-(4-bromobenzyl)-4-methyl-5-oxoisoxazolidine-2-carboxylate (3l):** Prepared by the general

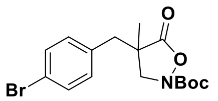

procedure D from **S2l** (500 mg, 1.40 mmol) and methyl iodide (174 µL, 2.80 mmol), and isolated as a white solid (400 mg, 77% yield). **m.p.** 102–104 °C; **^{1}H NMR** (400 MHz, $CDCl_3$): δ 7.47 – 7.43 (m, 2H), 7.07 – 7.04 (m, 2H), 4.00 (d, *J* = 11.2 Hz, 1H), 3.70 (d, *J* = 11.2 Hz, 1H), 2.94 (d, *J* = 14.0 Hz, 1H), 2.79 (d, *J* = 13.6 Hz, 1H), 1.51 (s, 9H), 1.28 (s, 3H); **^{13}C NMR** (101 MHz, $CDCl_3$): δ 176.78, 155.93, 134.00, 131.85, 131.82, 121.65, 84.20, 57.50, 45.19, 40.33, 28.05, 20.90; **IR** (thin film): 3020, 2983, 2936, 1795, 1715, 1589, 1489, 1371, 1215, 1149, 1073, 1012, 844, 759 cm^{-1}; **HRMS** (ESI) *m/z* calc'd for $C_{17}H_{24}O_5NBrNa$ $[M+Na+MeOH]^+$ 424.0730, found: 424.0735.

***tert*-Butyl 4-(2-chlorobenzyl)-4-methyl-5-oxoisoxazolidine-2-carboxylate (3m):** Prepared by the general

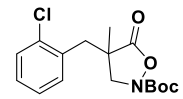

procedure D from **S2m** (500 mg, 1.60 mmol) and methyl iodide (199 μL, 3.21 mmol), and isolated as a white solid (412 mg, 79% yield). **m.p.** 65–67 °C; **^{1}H NMR** (400 MHz, $CDCl_3$): δ 7.40 – 7.36 (m, 1H), 7.31 – 7.27 (m, 1H), 7.24 – 7.20 (m, 2H), 3.92 – 3.86 (m, 2H), 3.17 (dd, *J* = 45.9, 14.1 Hz, 2H), 1.50 (s, 9H), 1.36 (s, 3H); **^{13}C NMR** (101 MHz, $CDCl_3$): δ 176.72, 155.93, 134.99, 133.44, 132.10, 129.83, 128.96, 127.27, 84.01, 57.75, 45.92, 37.31, 28.04, 21.63; **IR** (thin film): 3062, 2980, 2936, 1796, 1371, 1259, 1149, 1072, 847, 755 cm^{-1}; **HRMS** (ESI) *m/z* calc'd for $C_{16}H_{20}O_4NClNa$ $[M + Na]^+$: 348.0973, found: 348.0975.

***tert*-Butyl 4-methyl-4-(3-methylbenzyl)-5-oxoisoxazolidine-2-carboxylate (3n):** Prepared by the general

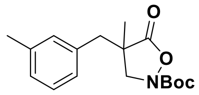

procedure D from **S2n** (400 mg, 1.37 mmol) and methyl iodide (171 μL, 2.74 mmol), and isolated as a white solid (327 mg, 78% yield). **m.p.** 78–80 °C; **^{1}H NMR** (400 MHz, $CDCl_3$): δ 7.23 – 7.19 (m, 1H), 7.10 – 7.08 (m, 1H), 6.97 (d, *J* = 7.2 Hz, 2H), 4.05 (d, *J* = 11.2 Hz, 1H), 3.69 (d, *J* = 11.2 Hz, 1H), 2.95 (d, *J* = 14.0 Hz, 1H), 2.79 (d, *J* = 13.6 Hz, 1H), 2.34 (s, 3H), 1.51 (s, 9H), 1.27 (s, 3H); **^{13}C NMR** (101 MHz, $CDCl_3$): δ 177.24, 156.04, 138.33, 134.99, 130.90, 128.54, 128.17, 127.22, 83.99, 57.68, 45.25, 40.77, 28.05, 21.40, 20.86; **IR** (thin film): 3023, 2981, 2936, 1795, 1717, 1458, 1371, 1216, 1149, 1072, 847, 756 cm^{-1}; **HRMS** (ESI) *m/z* calc'd for $C_{17}H_{23}O_4NNa$ $[M + Na]^+$: 328.1519, found: 328.1521.

***tert*-Butyl 4-(3-methoxybenzyl)-4-methyl-5-oxoisoxazolidine-2-carboxylate (3o):** Prepared by the general

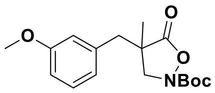

procedure D from **S2o** (500 mg, 1.62 mmol) and methyl iodide (202 μL, 3.25 mmol), and isolated as a colorless liquid (340 mg, 65% yield). **^{1}H NMR** (400 MHz, $CDCl_3$): δ 7.26 – 7.21 (m, 1H), 6.84-6.81 (m, 1H), 6.76 – 6.71 (m, 2H), 4.06 (d, *J* = 11.2 Hz, 1H), 3.80 (s, 3H), 3.70 (d, *J* = 11.2 Hz, 1H), 2.96 (d, *J* = 13.6 Hz, 1H), 2.80 (d, *J* = 13.6 Hz, 1H), 1.51 (s, 9H), 1.29 (s, 3H); **^{13}C NMR** (101 MHz, $CDCl_3$): δ 177.22, 159.74, 156.02, 136.56, 129.66, 122.48, 115.94, 112.76, 84.02, 57.65, 55.21, 45.26, 40.89, 28.05; **IR** (thin film): 3019, 2983, 2938, 1794, 1714, 1585, 1490, 1371, 1215, 1151, 1051, 846, 756 cm^{-1}; **HRMS** (ESI) *m/z* calc'd for $C_{17}H_{23}O_5NNa$ $[M + Na]^+$: 344.1468, found: 344.1469.

***tert*-Butyl 4-(3-fluorobenzyl)-4-methyl-5-oxoisoxazolidine-2-carboxylate (3p):** Prepared by the general

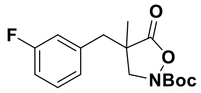

procedure D from **S2p** (500 mg, 1.69 mmol) and methyl iodide (211 μL, 3.39 mmol), and isolated as a white solid (319 mg, 61% yield). **m.p.** 88–90 °C; **^{1}H NMR** (400 MHz, $CDCl_3$): δ 7.30 (td, *J* = 8.0, 6.0 Hz, 1H), 7.01-6.95 (m, 2H), 6.91-6.88 (m, 1H), 4.01 (d, *J* = 10.8 Hz, 1H), 3.72 (d, *J* = 10.8 Hz, 1H), 2.98 (d, *J* = 13.6 Hz, 1H), 2.83 (d, *J* = 13.6 Hz, 1H), 1.51 (s, 9H), 1.29 (s, 3H); **^{13}C NMR** (101 MHz, $CDCl_3$): δ 176.80, 163.0 (d, *J* = 247.7 Hz), 155.96, 137.5 (d, *J* = 7.2 Hz), 130.21 (d, *J* = 8.2 Hz), 125.85 (d, *J* = 2.9 Hz), 117.0 (d, *J* = 21.3 Hz), 114.5 (d, *J* = 21.1 Hz), 84.19, 57.61, 45.22, 40.55, 40.54, 28.04, 20.88; **^{19}F NMR** (376 MHz, $CDCl_3$): δ -112.4 ; **IR** (thin film): 3021, 2983, 2937, 1795, 1715, 1589, 1488, 1450, 1371, 1215, 1149, 1078, 846, 754 cm^{-1}; **HRMS** (ESI) *m/z* calc'd for $C_{17}H_{24}O_5NFNa$ $[M + Na + MeOH]^+$: 364.1531, found: 364.1532.

***tert*-Butyl 4-methyl-5-oxo-4-(4-(4,4,5,5-tetramethyl-1,3-dioxolan-2-yl)benzyl)isoxazolidine-2-carboxylate (3q):**

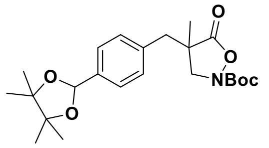

Prepared by the general procedure D from **S2q** (600 mg, 1.48 mmol) and methyl iodide (184 µL, 2.96 mmol), and isolated as a colorless liquid (290 mg, 47% yield). **^{1}H NMR** (400 MHz, $CDCl_3$): δ 7.46 (d, *J* = 8.0 Hz, 2H), 7.18 (d, *J* = 8.4 Hz, 2H), 5.95 (s, 1H), 4.02 (d, *J* = 11.2 Hz, 1H), 3.66 (d, *J* = 11.2 Hz, 1H), 2.97 (d, *J* = 14.0 Hz, 1H), 2.83 (d, *J* = 14.0 Hz, 1H), 1.52 (s, 9H), 1.32 (s, 6H), 1.27 (s, 6H), 1.26 (s, 3H); **^{13}C NMR** (101 MHz, $CDCl_3$) δ 177.13, 156.08, 138.88, 135.52, 130.17, 126.78, 99.65, 84.07, 82.78, 57.61, 45.25, 40.38, 28.06, 24.40, 22.19, 20.65; **IR** (thin film): 3018, 2982, 2936, 1795, 1715, 1457, 1371, 1216, 1151, 1074, 991, 846, 750 cm^{-1}; **HRMS** (ESI) *m/z* calc'd for $C_{23}H_{33}O_6NNa$ $[M + Na]^+$: 442.2200, found: 442.2202.

***tert*-Butyl 4-methyl-5-oxo-4-(4-(4,4,5,5-tetramethyl-1,3-dioxolan-2-yl)benzyl)isoxazolidine-2-carboxylate (3r):**

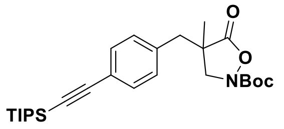

Prepared by the general procedure D from **S2r** (600 mg, 1.3 mmol) and methyl iodide (163 µL, 2.62 mmol), and isolated as a colorless liquid (306 mg, 50% yield). **^{1}H NMR** (400 MHz, $CDCl_3$): δ 7.43 (d, *J* = 8.4 Hz, 2H), 7.11 (d, *J* = 8.0 Hz, 2H), 4.01 (d, *J* = 11.2 Hz, 1H), 3.68 (d, *J* = 11.2 Hz, 1H), 2.98 (d, *J* = 13.6 Hz, 1H), 2.82 (d, *J* = 13.6 Hz, 1H), 1.50 (s, 9H), 1.27 (s, 3H), 1.12 (s, 21H); **^{13}C NMR** (101 MHz, $CDCl_3$): δ 176.89, 155.86, 135.30, 132.31, 130.01, 122.82, 106.49, 91.29, 84.11, 57.53, 45.32, 40.86, 28.05, 20.94, 18.66, 11.30; **IR** (thin film): 2943, 2865, 2156, 1796, 1718, 1507, 1461, 1370, 1222, 1150, 1071, 848, 755 cm^{-1}; **HRMS** (ESI) *m/z* calc'd for $C_{28}H_{45}O_5NNaSi$ [M + Na + MeOH]$^+$: 526.2959, found: 526.2961.

***tert*-Butyl 4-(4-(*tert*-butoxy)benzyl)-4-methyl-5-oxoisoxazolidine-2-carboxylate (3t):** Prepared by the general

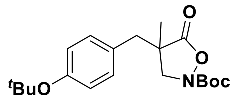

procedure D from **S2t** (700 mg, 2.0 mmol) and methyl iodide (249 µL, 4.0 mmol), and isolated as a white solid (567 mg, 78% yield). **m.p.** 83–85 °C; **^{1}H NMR** (400 MHz, $CDCl_3$): δ 7.08 – 7.04 (m, 2H), 6.95 – 6.92 (m, 2H), 4.04 (d, *J* = 11.2 Hz, 1H), 3.68 (d, *J* = 10.8 Hz, 1H), 2.93 (d, *J* = 14.0 Hz, 1H), 2.78 (d, *J* = 14.0 Hz, 1H), 1.52 (s, 9H), 1.34 (s, 9H), 1.26 (s, 3H); **^{13}C NMR** (101 MHz, $CDCl_3$): δ 177.25, 156.15, 154.85, 130.65, 129.71, 124.12, 84.04, 78.56, 57.63, 45.33, 40.11, 28.85, 28.06, 20.73; **IR** (thin film): 2979, 2935, 1795, 1717, 1508, 1456, 1368, 1215, 1153, 1071, 895, 754 cm^{-1}; **HRMS** (ESI) *m/z* calc'd for $C_{20}H_{29}O_5NNa$ $[M + Na]^+$: 386.1938, found: 386.1937.

***tert*-Butyl 4-(4-((*tert*-butyldimethylsilyl)oxy)phenethyl)-4-methyl-5-oxoisoxazolidine-2-carboxylate (3u):**

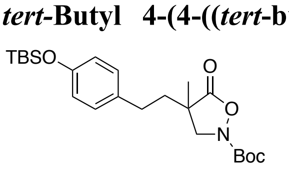

Prepared by the general procedure D from **S2u** (1.0 g, 2.37 mmol) and isolated as a colorless liquid (610 mg, 60% yield). **^{1}H NMR** (400 MHz, $CDCl_3$): δ 7.01 (d, *J* = 8.4 Hz, 2H), 6.75 (d, *J* = 8.4 Hz, 2H), 4.00 (d, *J* = 11.1 Hz, 1H), 3.80 (d, *J* = 11.1 Hz, 1H), 2.72 – 2.64 (m, 1H), 2.55 – 2.48 (m, 1H), 1.96 – 1.81 (m, 2H), 1.52 (s, 9H), 1.36 (s, 3H), 0.97 (s, 9H), 0.17 (s, 6H); **^{13}C NMR** (101 MHz, $CDCl_3$): δ 177.2, 156.2, 154.1, 133.1, 129.1, 120.2, 84.0, 58.8, 44.1, 37.7, 29.7, 28.1, 25.7, 20.6, 18.2, –4.5; **IR** (thin film): 2955, 2932, 2858, 1799, 1748, 1718, 1609, 1509, 1461, 1392, 1370, 1258, 1150, 1072, 916, 841, 781, 694 cm^{-1}; **HRMS (ESI)** *m/z* calc'd for $C_{23}H_{37}O_5NNaSi$ $[M + Na]^+$: 458.2333, found: 458.2332.

4-Benzyl-2,4-dimethylisoxazolidin-5-one (10): Prepared by the general procedure D from 2,4-

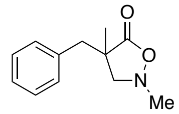

dimethylisoxazolidin-5-one[5] (150.9 mg, 1.31 mmol) and benzyl bromide (310 μL, 2.62 mmol), and isolated as a colorless oil (73 mg, 48% yield). Due to the slow nitrogen inversion,[6] spectra was obtained as a mixture of two conformational isomers. **^{1}H NMR** δ 7.37–7.26 (m, 3H), 7.18 (br, 2H), 3.62–3.10 (br, 2H), 2.89–2.84 (br, 3H), 2.80–2.56 (br, 2H), 1.39–1.20 (br, 3H); **^{13}C NMR** (101 MHz, $CDCl_3$): δ 178.3, 136.4, 130.8, 130.6, 130.0, 128.5, 127.1, 67.7, 67.2, 48.6, 46.9, 41.5, 39.9, 21.1, 19.4; **IR** (thin film): 3028, 2970, 1770, 1496, 1455, 1248, 1106, 938, 842, 769, 701 cm^{-1}; **HRMS** (ESI) *m/z* calc'd for $C_{12}H_{15}O_2NNa$ [M+Na]$^{+}$: 228.0995, found: 228.0995.

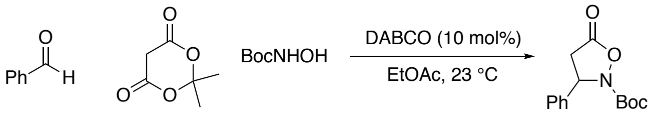

S3

***tert*-Butyl 5-oxo-3-phenylisoxazolidine-2-carboxylate (S3):**[2] To a solution of benzaldehyde (1.0 mL, 9.8 mmol, 1 equiv), Meldrum's acid (1.4 g, 9.8 mmol, 1 equiv) and *N*-Boc hydroxylamine (1.3 g, 9.8 mmol, 1 equiv) in EtOAc (39 mL, 0.25 M) was added DABCO (110 mg, 0.98 mmol, 10 mol%). The solution was stirred at 23 °C for 20 h and sat aq. NH_4Cl was added. The aqueous phase was extracted with EtOAc (3x). The combined organic layers were washed with brine, dried over Na_2SO_4, filtered, and concentrated under reduced pressure to give the residue, which was purified by silica gel column chromatography to give **S3** as a white solid (1.99 g, 77% yield). **m.p.** 56–58 °C; **^{1}H NMR** (400 MHz, $CDCl_3$): δ 7.41 – 7.31 (m, 5H), 5.54 (dd, *J* = 9.3, 4.1 Hz, 1H), 3.33 (dd, *J* = 17.7, 9.3 Hz, 1H), 2.85 (dd, *J* = 17.7, 4.1 Hz, 1H), 1.44 (s, 9H); **^{13}C NMR** (101 MHz, $CDCl_3$): δ 171.71, 155.27, 138.87, 129.08, 128.51, 125.75, 84.26, 62.99, 37.56, 27.98; **IR** (thin film): 3033, 2981, 2935, 1807, 1720, 1604, 1456, 1370, 1297, 1146, 962, 850, 761 cm^{-1}; **HRMS** (ESI) *m/z* calc'd for $C_{15}H_{21}O_5NNa$ [M + Na + MeOH]$^{+}$: 318.1312, found: 318.1316.

***tert*-Butyl 4,4-dibenzyl-5-oxo-3-phenylisoxazolidine-2-carboxylate (S4):** Prepared by the general procedure D

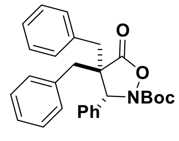

from **S3** (320 mg, 1.21 mmol) and benzyl bromide (289 μL, 2.43 mmol), and isolated as a white solid (151 mg, 34% yield). **m.p.** 55–57 °C; **^{1}H NMR** (400 MHz, $CDCl_3$): δ 7.38 – 7.27 (m, 9H), 7.25 – 7.20 (m, 5H), 7.00 – 6.97 (m, 2H), 5.15 (s, 1H), 3.31 (d, *J* = 14.4 Hz, 1H), 3.06 (d, *J* = 14.4 Hz, 1H), 2.73 (d, *J* = 14.4 Hz, 1H), 2.43 (d, *J* = 14.4 Hz, 1H), 1.25 (s, 9H); **^{13}C NMR** (101 MHz, $CDCl_3$): δ 173.34, 154.79, 135.46, 135.36, 134.64, 130.76, 130.42, 129.19, 129.04, 128.92, 128.84, 128.59, 128.44, 128.01, 127.57, 127.45, 127.10, 125.66, 83.78, 69.39, 54.48, 39.94, 39.18, 27.76; **IR** (thin film): 3030, 2934, 1793, 1716, 1605, 1455, 1370, 1237, 1147, 1078, 847, 754 cm^{-1}; **HRMS** (ESI) *m/z* calc'd for $C_{28}H_{30}O_4N$ [M + H]$^{+}$: 444.2169, found: 444.2165.

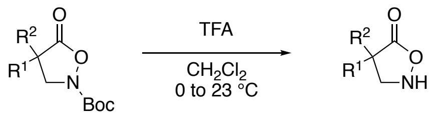

General procedure E:[2] To a solution of Boc-protected isoxazolidin-5-ones in CH_2Cl_2 (0.1 M) was added TFA (0.2 M) at 0 °C, and the resulting mixture was stirred at room temperature for 1 h. The reaction mixture was concentrated under reduced pressure. The resulting residue was diluted with EtOAc, followed by the addition of sat aq $NaHCO_3$. The aqueous phase was extracted with EtOAc (3x). The combined organic layers were washed with brine, dried over Na_2SO_4, filtered, and concentrated under reduced pressure to give the crude product, which was purified by silica gel column chromatography to afford the corresponding unprotected isoxazolidin-5-ones.

4-Benzyl-4-methylisoxazolidin-5-one (1a): Prepared by the general procedure E from **3a** (1.2 g, 4.12 mmol) and isolated as a white solid (669 mg, 85% yield).

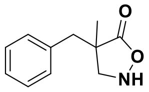

m.p. 54–56 °C; **^{1}H NMR** (400 MHz, $CDCl_3$): δ 7.36 – 7.30 (m, 3H), 7.23 – 7.21 (m, 2H), 3.63 (d, J = 11.6 Hz, 1H), 3.38 (d, J = 8.4 Hz, 1H), 3.10 (d, J = 13.6 Hz, 1H), 2.77 (d, J = 13.6 Hz, 1H), 1.35 (s, 3H); **^{13}C NMR** (101 MHz, $CDCl_3$): δ 181.57, 135.81, 129.43, 129.01, 127.67, 57.93, 45.43, 42.58, 22.35; **IR** (thin film): 3250, 3028, 2975, 2937, 1767, 1449, 1455, 1381, 1214, 1096, 967, 872, 757, 702 cm^{-1}; **HRMS** (ESI) *m/z* calc'd for $C_{11}H_{14}O_2N$ $[M + H]^+$: 192.1019, found: 192.1020.

4-Benzyl-4-hexylisoxazolidin-5-one (1b): Prepared by the general procedure E from **3b** (400 mg, 1.10 mmol) and isolated as a colorless liquid (202mg, 70% yield).

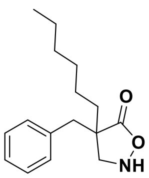

^{1}H NMR (400 MHz, $CDCl_3$): δ 7.35 – 7.28 (m, 3H), 7.23 – 7.21 (m, 2H), 5.67 (s, 1H), 3.52 (s, 2H), 3.06 (d, J = 13.6 Hz, 1H), 2.82 (d, J = 13.6 Hz, 1H), 1.72 – 1.67 (m, 2H), 1.51 – 1.45 (m, 1H), 1.36 – 1.23 (m, 7H), 0.89 (t, J = 6.8 Hz, 3H); **^{13}C NMR** (101 MHz, $CDCl_3$): δ 181.23, 135.87, 129.35, 129.10, 127.71, 55.53, 49.27, 41.48, 36.11, 31.54, 29.53, 24.22, 22.53, 14.02; **IR** (thin film): 3252, 3019, 2931, 2859, 1770, 1495, 1455, 1215, 1175, 1029, 871, 756 cm^{-1}; **HRMS** (ESI) *m/z* calc'd for $C_{16}H_{24}O_2N$ $[M + H]^+$: 262.1802, found: 262.1804.

4-Benzyl-4-phenethylisoxazolidin-5-one (1c): Prepared by the general procedure E from **3c** (300 mg, 0.78 mmol) and isolated as a white solid (163 mg, 74% yield).

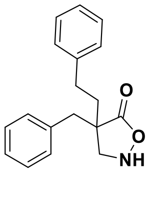

m.p. 70–72 °C; **^{1}H NMR** (400 MHz, $CDCl_3$): δ 7.36 – 7.29 (m, 5H), 7.25 – 7.19 (m, 5H), 3.56 (br, 2H), 3.15 (d, J = 13.6 Hz, 1H), 2.89 (d, J = 13.6 Hz, 1H), 2.85 – 2.80 (m, 1H), 2.72-264 (m, 1H), 2.07-1.99 (m, 2H); **^{13}C NMR** (101 MHz, $CDCl_3$): δ 180.82, 140.70, 135.61, 129.34, 129.20, 128.65, 128.31, 127.86, 126.37, 55.66, 49.26, 41.50, 37.88, 30.59; **IR** (thin film): 3250, 3064, 2927, 2861, 1769, 1602, 1496, 1455, 1348, 1216, 1167, 1040, 872, 756 cm^{-1}; **HRMS** (ESI) *m/z* calc'd for $C_{18}H_{20}O_2N$ $[M + H]^+$: 282.1489, found: 282.1486.

4-Benzyl-4-(pent-4-en-1-yl)isoxazolidin-5-one (1g): Prepared by the general procedure E from **3g** (400 mg, 1.15 mmol) and isolated as a light brown liquid (227 mg, 80% yield).

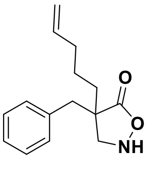

^{1}H NMR (400 MHz, $CDCl_3$): δ 7.36 – 7.31 (m, 3H), 7.26 – 7.21 (m, 2H), 5.79 (ddt, J = 16.9, 10.2, 6.7 Hz, 1H), 5.21 (br, 1H), 5.06 – 4.98 (m, 2H), 3.53 (s, 2H), 3.07 (d, J = 13.6 Hz, 1H), 2.82 (d, J = 13.6 Hz, 1H), 2.13 – 2.07

(m, 2H), 1.74 – 1.67 (m, 2H), 1.65 – 1.60 (m, 1H), 1.48 – 1.39 (m, 1H); **^{13}C NMR** (101 MHz, $CDCl_3$): δ 181.07, 137.63, 135.74, 129.32, 129.15, 127.79, 115.47, 55.51, 49.18, 41.51, 35.51, 33.75, 23.50; **IR** (thin film): 3251, 3077, 3027, 2942, 2862, 1769, 1641, 1496, 1455, 1216, 1175, 997, 916, 869, 757 cm^{-1}; **HRMS** (ESI) *m/z* calc'd for $C_{15}H_{20}O_2N$ $[M+H]^+$: 246.1489, found: 246.1490.

2-(4-Benzyl-5-oxoisoxazolidin-4-yl)acetonitrile (1h): Prepared by the general procedure E from **3h** (300 mg, 0.94 mmol) and isolated as a colorless liquid (118 mg, 58% yield). **m.p.** 75–77 °C; **^{1}H NMR** (400 MHz, $CDCl_3$): δ 7.39-7.35 (m, 3H), 7.26 – 7.24 (m, 2H), 3.75-3.67 (m, 2H), 3.17 (d, J = 13.6 Hz, 1H), 3.05 (d, J = 14.0 Hz, 1H), 2.73 (s, 2H); **^{13}C NMR** (101 MHz, $CDCl_3$): δ 177.76, 133.66, 129.43, 128.46, 115.99, 55.61, 47.34, 40.37, 28.62; **IR** (thin film): 3253, 3020, 2929, 2253, 1778, 1669, 1496, 1455, 1215, 1178, 1030, 927, 869, 754 cm^{-1}; **HRMS** (ESI) *m/z* calc'd for $C_{12}H_{13}O_2N_2$ $[M + H]^+$: 217.0972, found: 217.0974.

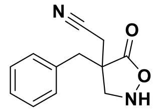

4-Benzyl-4-(prop-2-yn-1-yl)isoxazolidin-5-one (1i): Prepared by the general procedure E from **3i** (200 mg, 0.63 mmol) and isolated as a colorless liquid (68 mg, 50% yield). **^{1}H NMR** (400 MHz, $CDCl_3$): δ 7.37-7.31 (m, 3H), 7.24-7.22 (m, 2H), 5.91 (s, 1H), 3.74 (d, J = 12.0 Hz, 1H), 3.64 (d, J = 12.0 Hz, 1H), 3.09 (d, J = 13.6 Hz, 1H), 2.99 (d, J = 13.6 Hz, 1H), 2.59 (qd, J = 16.9, 2.7 Hz, 2H), 2.16 (dd, J = 5.9, 3.2 Hz, 1H); **^{13}C NMR** (101 MHz, $CDCl_3$): δ 179.74, 134.98, 129.46, 129.17, 127.96, 78.76, 72.10, 55.58, 48.84, 40.98, 25.95; **IR** (thin film): 3292, 3029, 2944, 1773, 1495, 1455, 1176, 1082, 1031, 917, 869, 757 cm^{-1}; **HRMS** (ESI) *m/z* calc'd for $C_{13}H_{14}O_2N$ $[M + H]^+$: 216.1019, found: 216.1020.

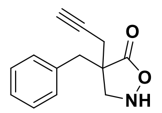

4-Methyl-4-(4-methylbenzyl)isoxazolidin-5-one (1j): Prepared by the general procedure E from **3j** (400 mg, 1.31 mmol) and isolated as a white solid (209 mg, 78% yield). **m.p.** 40–42 °C; **^{1}H NMR** (400 MHz, $CDCl_3$): δ 7.15-7.09 (m, 4H), 4.56 (br, 1H), 3.61 (d, J = 11.6 Hz, 1H), 3.39 (d, J = 11.6 Hz, 1H), 3.06 (d, J = 13.6 Hz, 1H), 2.72 (d, J = 14.0 Hz, 1H), 2.33 (s, 3H), 1.34 (s, 3H); **^{13}C NMR** (101 MHz, $CDCl_3$): δ 181.71, 137.46, 132.63, 129.76, 129.73, 129.23, 57.97, 45.44, 42.37, 22.35, 21.06; **IR** (thin film): 3249, 3022, 2975, 2935, 1766, 1514, 1455, 1380, 1211, 1092, 997, 966, 872, 809, 757 cm^{-1}; **HRMS** (ESI) *m/z* calc'd for $C_{12}H_{16}O_2N$ $[M + H]^+$: 206.1176, found: 206.1177.

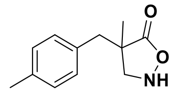

4-Methyl-4-(2-methylbenzyl)isoxazolidin-5-one (1k): Prepared by the general procedure E from **3k** (300 mg, 0.98 mmol) and isolated as a white solid (163 mg, 81% yield). **m.p.** 87–89 °C; **^{1}H NMR** (400 MHz, $CDCl_3$): δ 7.20 – 7.16 (m, 4H), 3.59 (d, J = 11.6 Hz, 1H), 3.39 (d, J = 11.6 Hz, 1H), 3.11 (d, J = 14.4 Hz, 1H), 2.95 (d, J = 14.4 Hz, 1H), 2.35 (s, 3H), 1.38 (s, 3H); **^{13}C NMR** (101 MHz, $CDCl_3$): δ 181.71, 136.63, 134.28, 131.12, 130.02, 127.64, 126.53, 57.87, 45.57, 38.47, 22.37, 20.02; **IR** (thin film): 3248, 2973, 2935, 1767, 1607, 1455, 1380, 1247, 1100, 1092, 997, 872, 757 cm^{-1}; **HRMS** (ESI) *m/z* calc'd for $C_{12}H_{16}O_2N$ $[M + H]^+$: 206.1176, found: 206.1178.

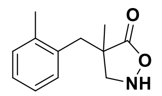

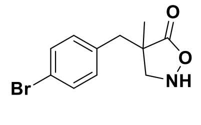

4-(4-Bromobenzyl)-4-methylisoxazolidin-5-one (1l): Prepared by the general procedure E from **3l** (400 mg, 1.08 mmol) and isolated as a white solid (244 mg, 84% yield). **m.p.** 73–75 °C; **^{1}H NMR** (400 MHz, $CDCl_3$): δ 7.48 – 7.44 (m, 2H), 7.10 – 7.06 (m, 2H), 3.59 (d, *J* = 11.2 Hz, 1H), 3.36 (d, *J* = 11.2 Hz, 1H), 3.04 (d, *J* = 13.6 Hz, 1H), 2.76 (d, *J* = 14.0 Hz, 1H), 1.33 (s, 3H); **^{13}C NMR** (101 MHz, $CDCl_3$): δ 180.93, 134.76, 132.06, 131.30, 121.71, 57.70, 45.29, 41.55, 21.78; **IR** (thin film): 3250, 3023, 2974, 2876, 1766, 1591, 1488, 1455, 1213, 1073, 1012, 802, 756 cm^{-1}; **HRMS** (ESI) *m/z* calc'd for $C_{11}H_{12}O_2NBrNa$ $[M+Na]^+$: 291.9944, found: 291.9944.

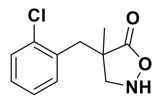

4-(2-Chlorobenzyl)-4-methylisoxazolidin-5-one (1m): Prepared by the general procedure E from **3m** (400 mg, 1.23 mmol) and isolated as a white solid (251 mg, 91% yield). **m.p.** 85–87 °C; **^{1}H NMR** (400 MHz, $CDCl_3$): δ 7.42 – 7.38 (m, 1H), 7.32-7.28 (m, 1H), 7.25 – 7.22 (m, 2H), 3.66 (d, *J* = 11.6 Hz, 1H), 3.38 (d, *J* = 11.6 Hz, 1H), 3.21 – 3.12 (m, 2H), 1.40 (s, 3H); **^{13}C NMR** (101 MHz, $CDCl_3$): δ 181.12, 134.62, 133.88, 131.90, 129.93, 129.04, 127.45, 57.44, 45.86, 37.87, 21.90; **IR** (thin film): 3254, 3019, 2977, 2935, 2878, 1769, 1474, 1445, 1215, 1091, 1038, 872, 757 cm^{-1}; **HRMS** (ESI) *m/z* calc'd for $C_{11}H_{13}O_2NCl$ $[M + H]^+$: 226.0629, found: 226.0631.

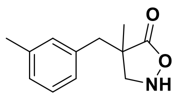

4-Methyl-4-(3-methylbenzyl)isoxazolidin-5-one (1n): Prepared by the general procedure E from **3n** (300 mg, 0.98 mmol) and isolated as a colorless liquid (159 mg, 79% yield). **^{1}H NMR** (400 MHz, $CDCl_3$): δ 7.22 (t, *J* = 7.4 Hz, 1H), 7.11 (d, *J* = 7.6 Hz, 1H), 7.02 (d, *J* = 7.2 Hz, 2H), 4.35 (br, 1H), 3.62 (d, *J* = 11.6 Hz, 1H), 3.39 (d, *J* = 11.6 Hz, 1H), 3.07 (d, *J* = 13.6 Hz, 1H), 2.72 (d, *J* = 13.6 Hz, 1H), 2.33 (s, 3H), 1.35 (s, 3H); **^{13}C NMR** (101 MHz, $CDCl_3$): δ 181.71, 138.81, 135.73, 130.08, 128.91, 128.46, 126.37, 57.99, 45.41, 42.67, 22.40, 21.40; **IR** (thin film): 3248, 2972, 2935, 2874, 1766, 1607, 1488, 1455, 1380, 1247, 1203, 1099, 967, 871 cm^{-1}; **HRMS** (ESI) *m/z* calc'd for $C_{12}H_{16}O_2N$ $[M + H]^+$: 206.1176, found: 206.1176.

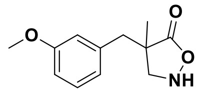

4-(3-Methoxybenzyl)-4-methylisoxazolidin-5-one (1o): Prepared by the general procedure E from **3o** (300 mg, 0.93 mmol) and isolated as a colorless liquid (167 mg, 81% yield). **^{1}H NMR** (400 MHz, $CDCl_3$): δ 7.25 (t, *J* = 8.0 Hz, 1H), 6.85 (ddd, *J* = 8.3, 2.5, 0.7 Hz, 1H), 6.80 (d, *J* = 7.6 Hz, 1H), 6.75 (t, *J* = 2.0 Hz 1H), 3.79 (s, 3H), 3.63 (d, *J* = 11.6 Hz, 1H), 3.40 (d, *J* = 11.6 Hz, 1H), 3.08 (d, *J* = 13.6 Hz, 1H), 2.73 (d, *J* = 13.6 Hz, 1H), 1.36 (s, 3H); **^{13}C NMR** (101 MHz, $CDCl_3$): δ 181.63, 159.95, 137.33, 130.05, 121.56, 115.13, 112.97, 58.01, 55.24, 45.43, 42.77, 22.42; **IR** (thin film): 3248, 3019, 2939, 2837, 1769, 1600, 1489, 1455, 1265, 1214, 1156, 1048, 872, 755 cm^{-1}; **HRMS** (ESI) *m/z* calc'd for $C_{12}H_{15}O_3NNa$ $[M + Na]^+$: 244.0944, found: 244.0946.

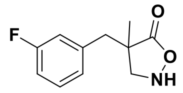

4-(3-Fluorobenzyl)-4-methylisoxazolidin-5-one (1p): Prepared by the general procedure E from **3p** (300 mg, 0.97 mmol) and isolated as a colorless liquid (178 mg, 88% yield). **^{1}H NMR** (400 MHz, $CDCl_3$): δ 7.33 – 7.28 (m, 1H), 7.03 – 6.98 (m, 2H), 6.94 – 6.90 (m, 1H), 5.38 (br, 1H), 3.63 (d, *J* = 11.2 Hz, 1H), 3.37 (d, *J* = 11.6 Hz, 1H), 3.09 (d, *J* = 13.6 Hz, 1H), 2.80 (d, *J* = 13.6 Hz, 1H), 1.35 (s, 3H); **^{13}C NMR** (101 MHz, $CDCl_3$): δ 180.97, 162.8 (d, *J* = 248.3 Hz), 138.2 (d, *J* = 7.2 Hz), 130.5 (d, *J* = 8.4

Hz), 125.3 (d, *J* = 2.9 Hz), 116.6 (d, *J* = 21.2 Hz), 114.6 (d, *J* = 21.0 Hz), 57.76, 45.35, 41.8 (d, *J* = 1.2 Hz), 30.93, 21.82; **^{19}F NMR** (376 MHz, $CDCl_3$): δ -112.02 ; **IR** (thin film): 3250, 2976, 2937, 1768, 1616, 1588, 1382, 1256, 1205, 1144, 1097, 945, 874, 793, 692 cm^{-1}; **HRMS** (ESI) *m/z* calc'd for $C_{11}H_{13}O_2NF$ [M + H]$^+$: 210.0925, found: 210.0926.

4-Methyl-4-(4-(4,4,5,5-tetramethyl-1,3-dioxolan-2-yl)benzyl)isoxazolidin-5-one (1q): Prepared by the general procedure E from **3q** (400 mg, 0.95 mmol) and isolated as a colorless liquid (106 mg, 35% yield). **^{1}H NMR** (400 MHz, $CDCl_3$): δ 7.47 (d, *J* = 8.4 Hz, 2H), 7.21 (d, *J* = 8.0 Hz, 2H), 5.95 (s, 1H), 3.61 (d, *J* = 11.6 Hz, 1H), 3.35 (d, *J* = 10.4 Hz, 1H), 3.10 (d, *J* = 13.6 Hz, 1H), 2.78 (d, *J* = 13.6 Hz, 1H), 1.34 (s, 3H), 1.32 (s, 6H), 1.27 (d, *J* = 2.1 Hz, 6H); **^{13}C NMR** (101 MHz, $CDCl_3$): δ 181.51, 139.22, 136.23, 129.44, 127.06, 99.54, 82.83, 82.81, 75.06, 57.78, 45.40, 42.12, 24.86, 24.36, 22.19; **IR** (thin film): 3468, 3250, 2981, 2938, 2877, 1769, 1462, 1389, 1215, 1156, 1075, 994, 950, 882, 770 cm^{-1}; **HRMS** (ESI) *m/z* calc'd for $C_{18}H_{25}O_4NNa$ [M+Na]$^+$: 342.1676, found: 342.1678.

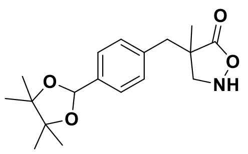

4-Methyl-4-(4-((triisopropylsilyl)ethynyl)benzyl)isoxazolidin-5-one (1r): Prepared by the general procedure E from **3r** (300 mg, 0.63 mmol) and isolated as a colorless liquid (118 mg, 50% yield); **^{1}H NMR** (400 MHz, $CDCl_3$): δ 7.45 – 7.42 (m, 2H), 7.15 (d, *J* = 8.4 Hz, 2H), 3.60 (d, *J* = 11.6 Hz, 1H), 3.36 (d, *J* = 11.6 Hz, 1H), 3.08 (d, *J* = 13.6 Hz, 1H), 2.78 (d, *J* = 13.6 Hz, 1H), 1.34 (s, 3H), 1.13 (s, 21H); **^{13}C NMR** (101 MHz, $CDCl_3$): δ 181.15, 136.02, 132.55, 129.41, 123.00, 106.31, 91.56, 57.79, 45.42, 42.24, 21.98, 18.66, 11.30; **IR** (thin film): 3251, 3027, 2943, 2865, 2156, 1769, 1507, 1461, 1382, 1215, 1175, 997, 916, 882, 758 cm^{-1}; **HRMS** (ESI) *m/z* calc'd for $C_{22}H_{33}O_2NNaSi$ [M+Na]$^+$: 394.2173, found: 394.2170.

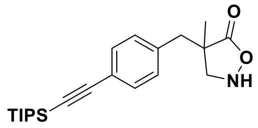

4-(4-Hydroxybenzyl)-4-methylisoxazolidin-5-one (15): Prepared by the general procedure E from **3t** (500 mg, 1.37 mmol) and isolated as a white solid (256 mg, 90% yield). **m.p.** 138–140 °C; **^{1}H NMR** (400 MHz, CD_3OD): δ 7.03 (d, *J* = 8.4 Hz, 2H), 6.74-6.71 (m, 2H), 3.60 (d, *J* = 11.2 Hz, 1H), 3.23 (d, *J* = 9.2 Hz, 1H), 2.93 (d, *J* = 13.6 Hz, 1H), 2.72 (d, *J* = 14.0 Hz, 1H), 1.25 (s, 3H); **^{13}C NMR** (101 MHz, CD_3OD): δ 182.42, 156.35, 130.73, 126.77, 114.87, 56.65, 45.35, 39.98, 19.87; **IR** (thin film): 3414, 2965, 2932, 2841, 1768, 1455, 1215, 1091, 1016, 967, 873, 806, 755 cm^{-1}; **HRMS** (ESI) *m/z* calc'd for $C_{11}H_{13}O_3NNa$ [M+Na]$^+$: 230.0788, found: 230.0791.

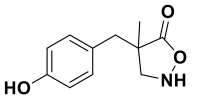

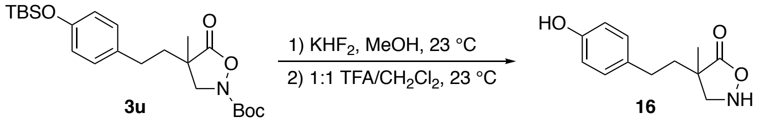

4-(4-Hydroxyphenethyl)-4-methylisoxazolidin-5-one (18): In an oven-dried 25 mL round-bottom flask, a mixture of **3u** (500 mg, 1.14 mmol) and KHF_2 (223 mg, 2.85 mmol) in anhydrous MeOH (10.0 mL) was stirred at 23 °C for 2 h.[7] The mixture was evaporated under reduced pressure to give crude sample of the free phenol,

which was dissolved in DCM (0.1 M). To this was added TFA at 0 °C, and the resulting mixture was stirred at 23 °C for 1 h. The reaction mixture was concentrated under reduced pressure. The resulting residue was diluted with EtOAc, followed by the addition of sat aq $NaHCO_3$. The aqueous phase was extracted with EtOAc (3x). The combined organic layers were washed with brine, dried over Na_2SO_4, filtered, and concentrated under reduced pressure to give the crude product, which was purified by silica gel column chromatography to afford the corresponding unprotected isoxazolidin-5-ones **18** as a white solid (140 mg, 55% yield). **m.p.** 120–122 °C; **^{1}H NMR** (400 MHz, $CDCl_3$): δ 7.06-7.03 (m, 2H), 6.78-6.74 (m, 2H), 3.56 (d, *J* = 11.2 Hz, 1H), 3.39 (d, *J* = 11.2 Hz, 1H), 2.73 (ddd, *J* = 13.6, 11.6, 5.4 Hz, 1H), 2.54 (ddd, *J* = 13.7, 11.6, 5.7 Hz, 1H), 2.04-1.84 (m, 2H), 1.37 (s, 3H); **^{13}C NMR** (101 MHz, $CDCl_3$): δ 181.0, 154.0, 132.9, 115.4, 58.8, 43.8, 37.5, 29.7, 20.3; **IR** (KBr): 3246, 3030, 2978, 2936, 1767, 1558, 1517, 1455, 1383, 1237, 1106, 891, 829, 704 cm^{-1}; **HRMS (ESI)** *m/z* calc'd for $C_{12}H_{15}O_3NNa$ $[M + Na]^+$: 244.0944, found: 244.0945.

3-Phenylisoxazolidin-5-one (12): Prepared by the general procedure E from **S3** (300 mg, 1.14 mmol) and

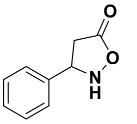

isolated as a colorless liquid (157 mg, 85% yield). **^{1}H NMR** (400 MHz, $CDCl_3$) δ 7.42 – 7.38 (m, 5H), 5.02 (dd, *J* = 9.4, 7.4 Hz, 1H), 3.10 (dd, *J* = 17.1, 7.3 Hz, 1H), 2.95 (dd, *J* = 17.1, 9.5 Hz, 1H); **^{13}C NMR** (101 MHz, $CDCl_3$) δ 176.27, 135.92, 129.22, 129.14, 126.70, 62.55, 36.91; **IR** (thin film): 3236, 3033, 2927, 1784, 1495, 1455, 1411, 1310, 1181, 1105, 889, 757cm^{-1}; **HRMS** (ESI) *m/z* calc'd for $C_9H_{10}O_2N$ $[M+H]^+$: 164.0712, found: 164.0705.

3-Phenyl-3λ^3-isoxazolidin-5-one-3-^{13}C (^{13}C-12): Prepared by the general procedure E from ^{13}C-**S3** (300 mg,

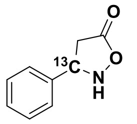

1.14 mmol) and isolated as a colorless liquid (155 mg, 83% yield). **^{1}H NMR** (400 MHz, $CDCl_3$): δ 7.44 – 7.36 (m, 5H), 5.00 (ddd, *J* = 144, 9.4, 7.4 Hz, 1H), 3.09 (ddd, *J* = 17.1, 7.3, 1.8 Hz, 1H), 2.94 (ddd, *J* = 17.1, 9.5, 4.0 Hz, 1H); **^{13}C NMR** (101 MHz, $CDCl_3$): δ 66.5; **IR** (thin film): 3237, 3020, 2927, 1787, 1495, 1455, 1412, 1215, 1182, 1093, 889, 756 cm^{-1}.

4,4-Dibenzyl-3-phenylisoxazolidin-5-one (13): Prepared by the general procedure E from **S4** (90 mg, 0.20

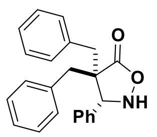

mmol) and isolated as a colorless liquid (55 mg, 79% yield). **m.p.** 50–52 °C; **^{1}H NMR** (400 MHz, $CDCl_3$): δ 7.47– 7.28 (m, 10H), 7.23 – 7.17 (m, 3H), 6.89 (d, *J* = 6.0 Hz, 2H), 4.81 (s, 1H), 3.43 (d, *J* = 14.4 Hz, 1H), 3.19 (d, *J* = 14.0 Hz, 1H), 2.83 (d, *J* = 14.4 Hz, 1H), 2.63 (d, *J* = 14.0 Hz, 1H); **^{13}C NMR** (101 MHz, $CDCl_3$): δ 178.69, 135.97, 134.81, 134.23, 130.54, 130.27, 128.94, 128.86, 128.56, 128.19, 127.43, 127.30, 65.60, 53.76, 40.42, 39.33; **IR** (thin film): 3269, 3030, 2983, 2929, 1778, 1602, 1496, 1455, 1370, 1216, 1146, 1029, 850, 757 cm^{-1}; **HRMS** (ESI) *m/z* calc'd for $C_{23}H_{21}O_2NNa$ $[M + Na]^+$: 366.1465, found: 366.1465.

3. Initial ligand screening

Table S1. Screening of ligands.[a]

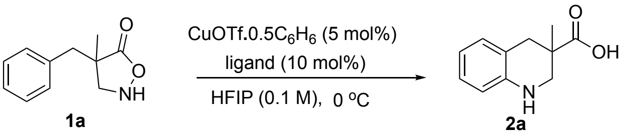

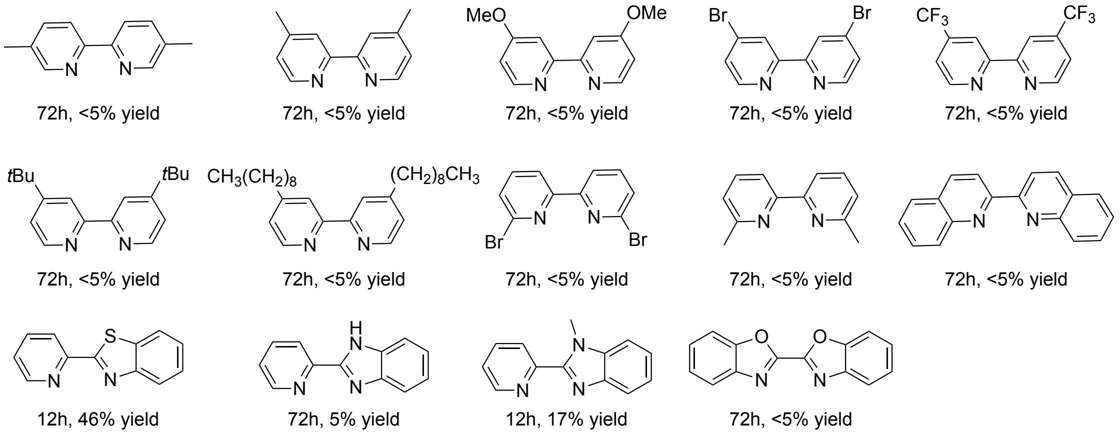

[a]**1a** (0.1 mmol). [b]Yields were determined by ^{1}H NMR analysis of the unpurified mixture.

4. Synthesis of cyclic β-amino acid

4-1. Scope of benzo-fused β-amino acid synthesis

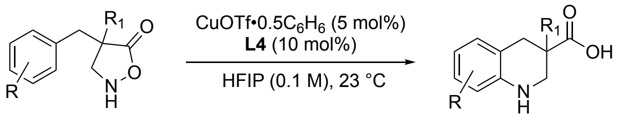

1 2

General procedure F: To a flame-dried 10 mL test tube equipped with a magnetic stirring bar were added **L4** (2.0 mg, 10 mol%) and Cu(OTf)•$0.5C_6H_6$ (90%, 1.4 mg, 5 mol%) in a glove box. After it was taken out from the glove box, HFIP (1.0 mL, 0.1 M) was added and the mixture was stirred for 10 minutes at an ambient temperature. Subsequently, the reaction was cooled with an ice bath, and substrate **1** (0.1 mmol) was added. After the bath was removed, the solution was stirred for the indicated time. The reaction mixture was quenched with aq EDTA and water. The aqueous layer was extracted with EtOAc (3x). The combined organic layers were concentrated under reduced pressure and the resulting residue was purified by silica gel column chromatography (0 to 20% MeOH in $CHCl_3$) to afford analytically pure products.

3-Methyl-1,2,3,4-tetrahydroquinoline-3-carboxylic acid (2a): Prepared by the general procedure F from **1a**

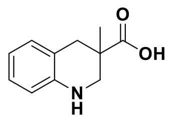

(19.1 mg, 0.1 mmol) and isolated as a white solid (18.7 mg, 98% yield). **m.p.** 117–120 °C; **^{1}H NMR** (400 MHz, $CDCl_3$): δ 7.01 (m, 2H), 6.68 (td, J = 7.2, 1.2 Hz, 1H), 6.54 (d, J = 7.6 Hz, 1H), 3.52 (dd, J = 11.6, 1.6 Hz, 1H), 3.20 – 3.10 (m, 2H), 2.68 (d, J = 16.4 Hz, 1H), 1.32 (s, 3H); **^{13}C NMR** (101 MHz, $CDCl_3$): δ 181.50, 142.67, 129.75, 127.02, 119.57, 118.16, 114.55, 49.01, 40.27, 36.38, 22.58; **IR** (thin film): 3413, 3018, 2968, 2932, 1698, 1607, 1586, 1503, 1468, 1322, 1285, 1133, 1077, 916, 748 cm^{-1}; **HRMS** (ESI) m/z calc'd for $C_{11}H_{14}O_2N$ [M + H]+: 192.1019, found: 192.1022.

1 mmol scale: To a flame-dried 25 mL flask equipped with a magnetic stirring bar were added **L4** (11.8 mg, 6 mol%) and Cu(OTf)•$0.5C_6H_6$ (90%, 14.0 mg, 5 mol%) in a glove box. After it was taken out from the glove box, HFIP (5.0 mL, 0.2 M) was added and the mixture was stirred for 10 minutes at an ambient temperature. Subsequently, the reaction was cooled with an ice bath, and **1a** (191 mg, 1.0 mmol) was added. After the bath was removed, the solution was stirred for 12. The reaction mixture was quenched with aq EDTA and water. The aqueous layer was extracted with EtOAc (3x). The combined organic layers were concentrated under reduced pressure and the resulting residue was purified by silica gel column chromatography (0 to 20% MeOH in $CHCl_3$) to afford **2a** (178 mg, 93%).

3-Hexyl-1,2,3,4-tetrahydroquinoline-3-carboxylic acid (2b): Prepared by the general procedure F from **1b**

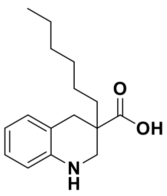

(26.1 mg, 0.1 mmol) and isolated as a white solid (18.2 mg, 70% yield). **m.p.** 130–132 °C; **^{1}H NMR** (400 MHz, $CDCl_3$): δ 7.00 – 6.97 (m, 2H), 6.67 (td, J = 7.4, 1.1 Hz, 1H), 6.52 (dd, J = 8.3, 1.1 Hz, 1H), 3.52 (dd, J = 11.6, 1.7 Hz, 1H), 3.17 (dd, J = 13.7, 9.1 Hz, 2H), 2.71 (d, J = 16.4 Hz, 1H), 1.66-1.60 (m, 2H), 1.32-1.24 (m, 8H), 0.87 (t, J = 6.8 Hz, 3H); **^{13}C NMR** (101 MHz, $CDCl_3$): δ 179.84, 143.08, 129.74, 126.93, 119.72, 118.15, 114.51, 47.77, 44.26, 36.42,

35.21, 31.62, 29.62, 24.19, 22.57, 14.03; **IR** (thin film): 3424, 3012, 2931, 2848, 1691, 1603, 1508, 1465, 1244, 1218, 772 cm^{-1}; **HRMS** (ESI) *m/z* calc'd for $C_{16}H_{24}O_2N$ $[M + H]^+$: 262.1802, found: 262.1805.

3-Phenethyl-1,2,3,4-tetrahydroquinoline-3-carboxylic acid (2c): Prepared by the general procedure F from **1c** (28.1 mg, 0.1 mmol) and isolated as a white solid (20.2 mg, 72% yield). **m.p.** 143–145 °C; **^{1}H NMR** (400 MHz, $CDCl_3$): δ 7.26-7.23 (m, 2H), 7.17-7.15 (m, 3H), 7.00 (t, J = 7.2 Hz, 2H), 6.68 (td, J = 7.5, 1.1 Hz, 1H), 6.53 (dd, J = 8.4, 1.1 Hz, 1H), 3.57 (dd, J = 11.6, 1.6 Hz, 1H), 3.25 (t, J = 12.7 Hz, 2H), 2.79 (d, J = 16.3 Hz, 1H), 2.71-2.61 (m, 2H), 2.04-1.89 (m, 2H); **^{13}C NMR** (101 MHz, $CDCl_3$): δ 179.70, 143.03, 141.46, 129.77, 128.41, 128.34, 127.04, 126.03, 119.39, 118.22, 114.52, 47.67, 44.31, 37.92, 35.27, 30.72. **IR** (thin film): 3403, 3057, 3024, 2927, 2855, 1701, 1606, 1497, 1452, 1280, 1241, 1219, 771 cm^{-1}; **HRMS** (ESI) *m/z* calc'd for $C_{18}H_{20}O_2N$ $[M + H]^+$: 282.1489, found: 282.1490.

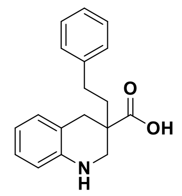

3-(Cyclohexylmethyl)-1,2,3,4-tetrahydroquinoline-3-carboxylic acid (2d): Prepared by the general procedure F from **1d** (27.3 mg, 0.1 mmol) and isolated as a white solid (18.5 mg, 68% yield). **m.p.** 158–160 °C; **^{1}H NMR** (400 MHz, $CDCl_3$): δ 6.97 (t, J = 6.9 Hz, 2H), 6.66 (td, J = 7.4, 1.1 Hz, 1H), 6.50 (dd, J = 8.4, 1.0 Hz, 1H), 3.50 (dd, J = 11.5, 1.7 Hz, 1H), 3.16 (dd, J = 21.3, 13.9 Hz, 2H), 2.69 (d, J = 16.3 Hz, 1H), 1.70-1.45 (m, 8H), 1.26-1.09 (m, 3H), .98-0.90 (m, 2H); **^{13}C NMR** (101 MHz, $CDCl_3$): δ 181.25, 143.17, 129.67, 126.87, 119.80, 118.08, 114.44, 48.65, 43.72, 43.65, 35.54, 34.53, 34.14, 33.87, 26.32, 26.30, 26.17; **IR** (thin film): 3412, 2922, 2850, 1698, 1607, 1503, 1447, 1282, 1218, 771 cm^{-1}; **HRMS** (ESI) *m/z* calc'd for $C_{17}H_{24}O_2N$ $[M + H]^+$: 274.1802, found: 274.1803.

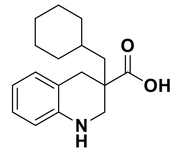

3-(3-(Benzyloxy)-3-oxopropyl)-1,2,3,4-tetrahydroquinoline-3-carboxylic acid (2e): Prepared by the general procedure F from **1e** (33.9 mg, 0.1 mmol) and isolated as a colorless liquid (27.1 mg, 80% yield). **^{1}H NMR** (400 MHz, $CDCl_3$): δ 7.38 – 7.30 (m, 5H), 7.00 – 6.95 (m, 2H), 6.66 (td, J = 7.4, 1.1 Hz, 1H), 6.51 – 6.49 (m, 1H), 5.08 (s, 2H), 3.50 (dd, J = 11.7, 1.4 Hz, 1H), 3.19 – 3.15 (m, 2H), 2.70 (d, J = 16.0 Hz, 1H), 2.54 – 2.41 (m, 2H), 2.09 – 1.95 (m, 2H); **^{13}C NMR** (101 MHz, $CDCl_3$): δ 179.75, 172.77, 142.86, 135.76, 129.78, 128.59, 128.37, 128.31, 127.12, 118.94, 118.23, 114.55, 66.50, 47.34, 43.49, 34.87, 30.39, 29.43. Spectroscopic data matched those reported.[2]

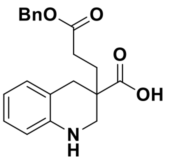

3-(2-(Phenylsulfonyl)ethyl)-1,2,3,4-tetrahydroquinoline-3-carboxylic acid (2f): Prepared by the general procedure F from **1f** (34.5 mg, 0.1 mmol) and isolated as a white solid (33.8 mg, 98% yield). **^{1}H NMR** (400 MHz, $CDCl_3$) δ 7.88– 7.86 (m, 2H), 7.66 – 7.62 (m, 1H), 7.56-7.52 (m, 2H), 7.03 – 6.96 (m, 2H), 6.78 (t, J = 7.4 Hz, 1H), 6.64 (d, J = 8.0 Hz, 1H), 3.50 (d, J = 12.0 Hz, 1H), 3.23 – 3.08 (m, 4H), 2.66 (d, J = 16.4 Hz, 1H), 2.11 – 1.98 (m, 2H); **^{13}C NMR** (101 MHz, $CDCl_3$): δ 177.27, 138.59, 133.93, 129.79, 129.40, 128.01, 127.51, 120.49, 119.88, 116.25, 77.33, 77.01, 76.70, 51.69, 47.09, 42.86, 34.82, 28.09. Spectroscopic data matched those reported.[2]

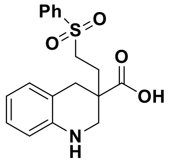

3-(Pent-4-en-1-yl)-1,2,3,4-tetrahydroquinoline-3-carboxylic acid (2g): Prepared by the general procedure F from **1g** (24.5 mg, 0.1 mmol) and isolated as a white solid (18.1 mg, 74% yield); **m.p.** 120–122 °C; **^{1}H NMR** (400 MHz, $CDCl_3$): δ 7.00 – 6.97 (m, 2H), 6.67 (td, *J* = 7.4, 1.1 Hz, 1H), 6.52 (dd, *J* = 8.3, 1.0 Hz, 1H), 5.77 (ddt, *J* = 16.9, 10.2, 6.6 Hz, 1H), 5.02 – 4.93 (m, 2H), 3.51 (dd, *J* = 11.6, 1.6 Hz, 1H), 3.17 (t, *J* = 12.4 Hz, 2H), 2.70 (d, *J* = 16.3 Hz, 1H), 2.05 (q, *J* = 7.2 Hz, 2H), 1.72 – 1.57 (m, 2H), 1.50 – 1.42 (m, 2H); **^{13}C NMR** (101 MHz, $CDCl_3$): δ 180.66, 143.13, 138.18, 129.74, 126.95, 119.55, 118.06, 114.90, 114.46, 47.70, 44.25, 35.69, 35.06, 33.89, 23.50; **IR** (thin film): 3422, 3015, 2938, 1698, 1606, 1502, 1266, 1243, 991, 911, 772, 748 cm^{-1}; **HRMS** (ESI) *m/z* calc'd for $C_{15}H_{20}O_2N$ $[M + H]^+$: 246.1489, found: 246.1490.

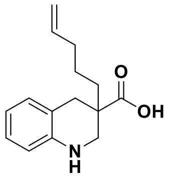

3-(Cyanomethyl)-1,2,3,4-tetrahydroquinoline-3-carboxylic acid (2h): Prepared by the general procedure F from **1h** (21.6 mg, 0.1 mmol) and isolated as a colorless liquid (12.5 mg, 58% yield); **^{1}H NMR** (600 MHz, CD_3OD): δ 6.96 – 6.91 (m, 2H), 6.59 – 6.55 (m, 2H), 3.45 (d, *J* = 11.4 Hz, 1H), 3.34 (s, 1H), 3.24 (d, *J* = 16.2 Hz, 1H), 2.76 (dd, *J* = 16.6, 2.2 Hz, 1H), 2.73 – 2.66 (m, 2H); **^{13}C NMR** (151 MHz, CD_3OD): δ 176.53, 144.31, 130.81, 128.34, 119.14, 118.40, 118.13, 115.25, 47.39, 43.18, 35.84, 22.50; **IR** (thin film): 3406, 3019, 2962, 2928, 2854, 2251, 1715, 1607, 1498, 1284, 1219, 1096, 1032, 855, 770 cm^{-1}; **HRMS** (ESI) *m/z* calc'd for $C_{12}H_{13}O_2N_2$ $[M + H]^+$: 217.0972, found: 217.0973.

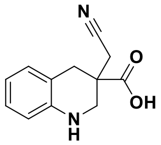

5-Methylene-1',4,4',5-tetrahydro-2H,2'H-spiro[furan-3,3'-quinolin]-2-one (5): Prepared by the general procedure F from **1i** (21.6 mg, 0.1 mmol) and isolated as a colorless liquid (15.0 mg, 70% yield). **^{1}H NMR** (400 MHz, $CDCl_3$): δ 7.04 (t, *J* = 7.6 Hz, 1H), 6.99 (d, *J* = 7.2 Hz, 1H), 6.68 (td, *J* = 7.4, 1.1 Hz, 1H), 6.56 (d, *J* = 8.0 Hz, 1H), 4.82 (dd, *J* = 4.5, 2.1 Hz, 1H), 4.34 (dd, *J* = 4.4, 2.0 Hz, 1H), 3.44 (d, *J* = 11.6 Hz, 1H), 3.23 (d, *J* = 15.6 Hz, 1H), 3.17 (dd, *J* = 11.4, 2.8 Hz, 1H), 2.91 (dt, *J* = 16.6, 1.7 Hz, 1H), 2.67 (dd, *J* = 15.9, 2.7 Hz, 1H), 2.61 (dd, *J* = 16.6, 0.8 Hz, 1H); **^{13}C NMR** (101 MHz, $CDCl_3$): δ 177.75, 153.47, 142.44, 131.50, 130.14, 127.64, 117.96, 117.26, 114.24, 89.94, 47.04, 41.78, 35.98, 35.41; **IR** (thin film): 3405, 2921, 2848, 1786, 1671, 1607, 1497, 1361, 1294, 1165, 1013, 772 cm^{-1}; **HRMS** (ESI) *m/z* calc'd for $C_{13}H_{14}O_2N$ $[M + H]^+$: 216.1019, found: 216.1021.

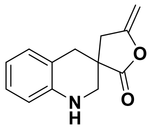

3,7-Dimethyl-1,2,3,4-tetrahydroquinoline-3-carboxylic acid (2j): Prepared by the general procedure F from **1j** (20.5 mg, 0.1 mmol) and isolated as a white solid (18.4 mg, 90% yield). **m.p.** 195–197 °C; **^{1}H NMR** (400 MHz, $CDCl_3$): δ 6.88 (d, *J* = 7.6 Hz, 1H), 6.52 (dd, *J* = 7.6, 0.8 Hz, 1H), 6.38 (s, 1H), 3.49 (dd, *J* = 11.5, 1.7 Hz, 1H), 3.16-3.08 (m, 2H), 2.65 (d, *J* = 16.4 Hz, 1H), 2.22 (s, 3H), 1.31 (s, 3H); **^{13}C NMR** (101 MHz, $CDCl_3$): δ 180.52, 142.32, 136.78, 129.59, 119.47, 116.79, 115.23, 49.06, 40.28, 36.21, 22.67, 21.11; **IR** (thin film): 3415, 2962, 2922, 1688, 1618, 1580, 1498, 1242, 1078, 917, 791, 745 cm^{-1}; **HRMS** (ESI) *m/z* calc'd for $C_{12}H_{16}O_2N$ $[M + H]^+$: 206.1176, found: 206.1175.

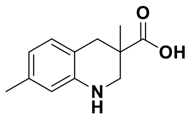

3,5-Dimethyl-1,2,3,4-tetrahydroquinoline-3-carboxylic acid (2k): Prepared by the general procedure F from

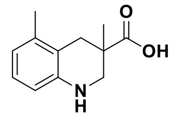

1k (20.5 mg, 0.1 mmol) and isolated as a white solid (16.6 mg, 81% yield). **m.p.** 155–157 °C; **^{1}H NMR** (400 MHz, $CDCl_3$): δ 6.93 (t, *J* = 7.7 Hz, 1H), 6.62 (d, *J* = 7.2 Hz, 1H), 6.47 (d, *J* = 8.0 Hz, 1H), 3.48 (dd, *J* = 11.2, 1.6 Hz, 1H), 3.10-3.05 (m, 2H), 2.52 (d, *J* = 17.2 Hz, 1H), 2.19 (s, 3H), 1.36 (s, 3H); **^{13}C NMR** (101 MHz, $CDCl_3$): δ 179.42, 142.23, 137.52, 126.52, 120.79, 118.97, 113.08, 48.76, 40.44, 34.34, 23.29, 19.33; **IR** (thin film): 3404, 2965, 2927, 2873, 1701, 1590, 1482, 1468, 1282, 1230, 1091, 879, 769 cm^{-1}; **HRMS** (ESI) *m/z* calc'd for $C_{12}H_{16}O_2N$ $[M + H]^+$: 206.1176, found: 206.1179.

7-Bromo-3-methyl-1,2,3,4-tetrahydroquinoline-3-carboxylic acid (2l): Prepared by the general procedure F

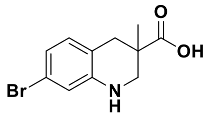

from **1l** (26.9 mg, 0.1 mmol) and isolated as a white solid (18.8 mg, 70% yield). **m.p.** 208–210 °C; **^{1}H NMR** (400 MHz, CD_3OD): δ 6.78 (d, *J* = 8.0 Hz, 1H), 6.63 – 6.58 (m, 2H), 3.44 (dd, *J* = 11.6, 1.5 Hz, 1H), 3.06 – 3.01 (m, 2H), 2.55 (d, *J* = 16.0 Hz, 1H), 1.23 (s, 3H); **^{13}C NMR** (101 MHz, CD_3OD): δ 182.27, 149.24, 134.45, 123.50, 122.46, 121.81, 119.38, 43.29, 39.84, 25.17; **IR** (thin film): 3412, 2968, 2929, 1698, 1598, 1494, 1283, 1238, 1068, 902, 861, 841, 789 cm^{-1}; **HRMS** (ESI) *m/z* calc'd for $C_{11}H_{13}O_2NBr$ $[M+H]^+$: 270.0124, found: 270.0127.

5-Chloro-3-methyl-1,2,3,4-tetrahydroquinoline-3-carboxylic acid (2m): Prepared by the general procedure F

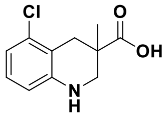

from **1m** (22.5 mg, 0.1 mmol) and isolated as a white solid (9.2 mg, 40% yield). **m.p.** 170–172 °C; **^{1}H NMR** (400 MHz, $CDCl_3$): δ 6.92 (t, *J* = 8.0 Hz, 1H), 6.75 (dd, *J* = 7.9, 1.2 Hz, 1H), 6.44 (dd, *J* = 8.0, 1.2 Hz, 1H), 3.51 (dd, *J* = 11.6, 1.6 Hz, 1H), 3.27 (dd, *J* = 17.2, 1.6 Hz, 1H), 3.09 (dd, *J* = 11.2, 0.8 Hz, 1H), 2.64 (d, *J* = 17.2 Hz, 1H), 1.35 (s, 3H); **^{13}C NMR** (101 MHz, $CDCl_3$): δ 179.75, 144.39, 134.97, 127.37, 118.63, 117.67, 112.77, 48.65, 40.45, 34.19, 22.84; **IR** (thin film): 3408, 2960, 2917, 2849, 1693, 1603, 1494, 1463, 1260, 1089, 1028, 772 cm^{-1}; **HRMS** (ESI) *m/z* calc'd for $C_{11}H_{13}O_2NCl$ $[M + H]^+$: 226.0629, found: 226.0629.

3,6-Dimethyl-1,2,3,4-tetrahydroquinoline-3-carboxylic acid (2n): Prepared by the general procedure F from

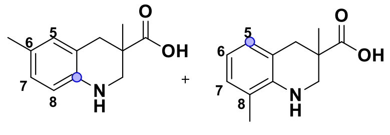

1n (20.5 mg, 0.1 mmol) and isolated as a white solid (19.0 mg, 93% yield). The ratio of C6-isomer and C8-isomer was 65:35. **m.p.** 170–172 °C; **^{1}H NMR** (400 MHz, $CDCl_3$) for the mixture of isomer: δ 6.89 (t, *J* = 8.0 Hz, 0.77H), 6.82 (d, *J* = 7.2 Hz, 1.29H), 6.61 (t, *J* = 7.4 Hz, 0.36 H), 6.48 (d, *J* = 8.4 Hz, 0.65H), 3.57 (dd, *J* = 11.6, 1.7 Hz, 0.35H), 3.48 (dd, *J* = 11.5, 1.7 Hz, 0.65H), 3.15-3.06 (m, 2H), 2.70 (d, *J* = 16.4 Hz, 0.35H), 2.65 (d, *J* = 16.4 Hz, 0.66H), 2.21 (s, 1.95 H), 2.09 (s, 1.05H), 1.32 (s, 3H); **^{13}C NMR** (101 MHz, $CDCl_3$) for the mixture of isomer: δ 181.26, 180.98, 140.73, 140.04, 130.16, 128.13, 127.84, 127.66, 127.59, 121.71, 119.91, 118.94, 117.55, 114.95, 49.27, 49.17, 40.33, 40.02, 36.64, 36.48, 22.72, 22.47, 20.45, 17.11; **IR** (thin film): 3418, 2965, 2930, 1697, 1598, 1511, 1469, 1288, 1252, 1218, 897, 810, 754 cm^{-1}; **HRMS** (ESI) *m/z* calc'd for $C_{12}H_{16}O_2N$ $[M + H]^+$: 206.1176, found: 206.1179.

6-Methoxy-3-methyl-1,2,3,4-tetrahydroquinoline-3-carboxylic acid (2oa): Prepared by the general procedure F from **1o** (22.1 mg, 0.1 mmol) and isolated as a white solid (18.3 mg, 85% yield). The ratio of C6-isomer and C8-isomer was 40:60. **m.p.** 130–132 °C; **^{1}H NMR** (400 MHz, $CDCl_3$): δ 6.64 (dd, *J* = 8.6, 2.8 Hz, 1H), 6.57 (dd, *J* = 10.3, 5.7 Hz, 2H), 3.73 (s, 3H), 3.46 (dd, *J* = 11.5, 1.8 Hz, 1H), 3.15 (d, *J* = 16.6 Hz, 1H), 3.07 (d, *J* = 11.5 Hz, 1H), 2.69 (d, *J* = 16.8 Hz, 1H), 1.32 (s, 3H); **^{13}C NMR** (101 MHz, $CDCl_3$): δ 179.40, 153.20, 135.87, 121.73, 116.55, 114.57, 113.45, 55.68, 49.64, 40.16, 37.06, 22.93; **IR** (thin film): 3394, 2932, 1702, 1506, 1464, 1238, 1194, 1037, 808, 755 cm^{-1}; **HRMS** (ESI) *m/z* calc'd for $C_{12}H_{16}O_3N$ $[M + H]^+$: 222.1125, found: 222.1127.

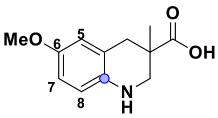

8-Methoxy-3-methyl-1,2,3,4-tetrahydroquinoline-3-carboxylic acid (2ob): m.p. 150–152 °C; **^{1}H NMR** (400 MHz, $CDCl_3$): δ 6.66 – 6.61 (m, 3H), 3.82 (s, 3H), 3.54 (dd, *J* = 11.4, 1.6 Hz, 1H), 3.18 (dd, *J* = 16.5, 1.4 Hz, 1H), 3.13 (dd, *J* = 11.4, 0.8 Hz, 1H), 2.70 (d, *J* = 16.5 Hz, 1H), 1.32 (s, 3H); **^{13}C NMR** (101 MHz, $CDCl_3$): δ 180.74, 146.56, 132.33, 121.70, 119.83, 117.40, 107.53, 55.38, 48.61, 40.10, 36.22, 22.68; **IR** (thin film): 3438, 2936, 1698, 1587, 1503, 1452, 1302, 1250, 1090, 943, 760, 728 cm^{-1}; **HRMS** (ESI) *m/z* calc'd for $C_{12}H_{16}O_3N$ $[M + H]^+$: 222.1125, found: 222.1128.

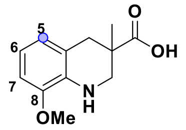

6-Fluoro-3-methyl-1,2,3,4-tetrahydroquinoline-3-carboxylic acid (2p): Prepared by the general procedure F from **1p** (20.9 mg, 0.1 mmol) and isolated as a white solid (17.3 mg, 83% yield). The ratio of C6-isomer and C8-isomer was 78:22. **m.p.** 128–130 °C; **^{1}H NMR** (400 MHz, $CDCl_3$) for the mixture of isomer: δ 6.83 – 6.76 (m, 0.41H), 6.74 – 6.70 (m, 1.55H), 6.57 (dt, *J* = 7.8, 3.9 Hz, 0.20H), 6.48 (dd, *J* = 9.5, 4.8 Hz, 0.79H), 3.57 (dd, *J* = 11.5, 1.6 Hz, 0.20H), 3.50 (dd, *J* = 11.6, 1.8 Hz, 0.80H), 3.22-3.07 (m, 2H), 2.71-2.63 (m, 1H), 1.33 (s, 0.60H), 1.32 (s, 2.42H); **^{13}C NMR** (101 MHz, $CDCl_3$) for the mixture of isomer: δ 180.97, 180.82, 156.1 (d, *J* = 237.0 Hz), 138.7 (d, *J* = 1.4 Hz), 124.69 (d, *J* = 2.9 Hz) , 121.60 (d, *J* = 3.7 Hz, 0.2), 121.15 (d, *J* = 7.1 Hz), 116.73 (d, *J* = 7.3 Hz) , 115.74 (d, *J* = 22.1 Hz), 115.54 (d, *J* = 7.6 Hz), 113.74 (d, *J* = 22.6 Hz), 112.47 (d, *J* = 17.9 Hz), 49.33, 48.35, 40.19, 36.41, 35.85 (d, *J* = 2.8 Hz), 22.60, 22.50; **^{19}F NMR** (376 MHz, $CDCl_3$): δ -126.6, -137.88; for the mixture of isomers; **IR** (thin film): 3402, 2965, 2930, 1702, 1505, 1469, 1239, 1185, 900, 807, 762 cm^{-1}; **HRMS** (ESI) *m/z* calc'd for $C_{11}H_{13}O_2NF$ $[M + H]^+$: 210.0925, found: 210.0926.

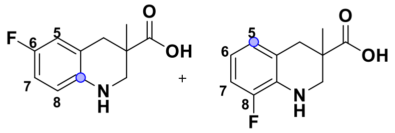

3-Methyl-7-(4,4,5,5-tetramethyl-1,3-dioxolan-2-yl)-1,2,3,4-tetrahydroquinoline-3-carboxylic acid (2q): Prepared by the general procedure F from **1q** (31.9 mg, 0.1 mmol) and isolated as a white solid (17.5 mg, 55% yield). **m.p.** 148–150 °C; **^{1}H NMR** (400 MHz, $CDCl_3$): δ 6.97 (d, *J* = 7.6 Hz, 1H), 6.81 (dd, *J* = 7.8, 1.5 Hz, 1H), 6.67 (d, *J* = 1.4 Hz, 1H), 5.85 (s, 1H), 3.49 (dd, *J* = 11.5, 1.6 Hz, 1H), 3.15 (d, *J* = 16.4, 1H), 3.11 (d, *J* = 11.2, 1H), 2.67 (d, *J* = 16.4 Hz, 1H), 1.30 (s, 3H), 1.29 (s, 6H), 1.26 (s, 6H); **^{13}C NMR** (101 MHz, $CDCl_3$): δ 180.03, 142.43, 138.59, 129.71, 120.15, 116.36, 112.36, 99.83, 82.51, 49.03, 40.09, 36.42, 24.40, 22.53, 22.18; **IR** (thin film): 3401, 2977, 2929, 2854, 1702, 1618, 1587, 1495, 1388, 1288, 1157, 1075, 988, 879, 756 cm^{-1}; **HRMS** (ESI) *m/z* calc'd for $C_{18}H_{26}O_4N$ $[M + H]^+$: 320.1856, found: 320.1855.

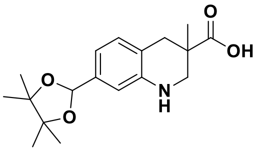

3-Methyl-7-((triisopropylsilyl)ethynyl)-1,2,3,4-tetrahydroquinoline-3-carboxylic acid (2r): Prepared by the general procedure F from **1r** (37.1 mg, 0.1 mmol) and isolated as a white solid (16.6 mg, 45% Yield); **m.p.** 78–80 °C; **^{1}H NMR** (400 MHz, $CDCl_3$): δ 6.90 (d, *J* = 7.6 Hz, 1H), 6.79 (dd, *J* = 7.7, 1.5 Hz, 1H), 6.65 (d, *J* = 1.4 Hz, 1H), 3.51 (dd, *J* = 11.6, 1.5 Hz, 1H), 3.15 (d, *J* = 16.4, 2H), 3.10 (d, *J* = 11.6, 2H), 2.65 (d, *J* = 16.4 Hz, 1H), 1.30 (s, 3H), 1.10 (s, 21H); **^{13}C NMR** (101 MHz, $CDCl_3$): δ 180.11, 142.42, 129.55, 121.94, 121.85, 120.18, 117.53, 107.44, 89.07, 48.97, 40.11, 36.44, 31.58, 22.65, 22.51, 18.66, 14.11, 11.32; **IR** (thin film): 3412, 2941, 2864, 2153, 1698, 1608, 1568, 1463, 1219, 996, 882, 772 cm^{-1}; **HRMS** (ESI) *m/z* calc'd for $C_{22}H_{34}O_2NSi$ $[M + H]^+$: 372.2353, found: 372.2354.

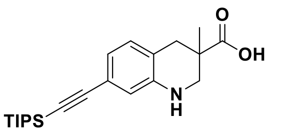

1,2,3,4-Tetrahydrobenzo[g]quinoline-3-carboxylic acid (2s): Prepared by the general procedure F from **1s** (22.7 mg, 0.1 mmol) and isolated as a light yellow solid (15.8 mg, 70% yield); **^{1}H NMR** (400 MHz, $CDCl_3$): δ 7.76 – 7.69 (m, 2H), 7.43 – 7.40 (m, 2H), 7.23 (brd, *J* = 8.3 Hz, 1H), 7.15 (brd, *J* = 8.4 Hz, 1H), 3.76 – 3.72 (m, 1H), 3.60-3.57 (m, 1H), 3.25 – 3.14 (m, 2H), 3.08-3.03 (m, 1H); **^{13}C NMR** (101 MHz, $CDCl_3$): δ 178.14, 137.88, 133.09, 128.56, 128.15, 125.32, 125.13, 123.36, 119.55, 118.11, 114.19, 43.69, 37.91, 29.78. Spectroscopic data matched those reported.[2]

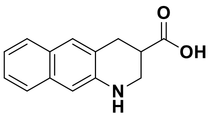

2-(1,2,3,4-Tetrahydroquinolin-2-yl)acetic acid (7): Prepared by the general procedure F from **6** (19.1 mg, 0.1 mmol) and isolated as a brown oil (11.4 mg, 60% yield). **^{1}H NMR** (400 MHz, $CDCl_3$): δ 7.02–6.97 (m, 2H), 6.69 (td, *J* = 7.4, 1.0 Hz, 1H), 6.58 (d, *J* = 7.7 Hz, 1H), 3.77- 3.71 (m, 1H), 2.90–2.71 (m, 2H), 2.62 (d, *J* = 5.8 Hz, 2H), 2.04 –1.97 (m, 1H), 1.84–1.74 (m, 1H); **^{13}C NMR** (101 MHz, $CDCl_3$): δ 175.70, 142.48, 129.39, 127.01, 121.91, 118.94, 115.77, 48.04, 39.92, 27.55, 25.44. Spectroscopic data matched those reported.[2]

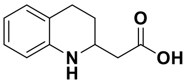

3-Benzyl-2-phenyl-1,2,3,4-tetrahydroquinoline-3-carboxylic acid (14): Prepared by the general procedure F from **13** (34.3 mg, 0.1 mmol) and isolated as a white solid (10.2 mg, 30% yield). The relative configuration was determined by NOE analyses. **m.p.** 213–215 °C; **^{1}H NMR** (400 MHz, $CDCl_3$): δ 7.24 – 7.21 (m, 8H), 7.14 (t, *J* = 7.3 Hz, 1H), 7.05 (d, *J* = 7.4 Hz, 1H), 7.03- 6.98 (m, 2H), 6.77 (td, *J* = 7.4, 1.0 Hz, 1H), 6.65 – 6.63 (m, 1H), 4.64 (d, *J* = 1.4 Hz, 1H), 3.26 (d, *J* = 13.3 Hz, 1H), 3.02 (d, *J* = 13.4 Hz, 1H), 2.93 (d, *J* = 17.0 Hz, 1H), 2.59 (d, *J* = 16.2 Hz, 1H); **^{13}C NMR** (101 MHz, $CDCl_3$): δ 178.82, 142.73, 142.41, 136.84, 130.31, 130.13, 128.30, 128.22, 127.80, 127.56, 127.43, 126.80, 117.84, 117.61, 113.24, 60.89, 49.93, 41.32, 26.62; **IR** (thin film): 3439, 3019, 2927, 1706, 1608, 1493, 1215, 928, 771 cm^{-1}; **HRMS** (ESI) *m/z* calc'd for $C_{23}H_{21}O_2NNa$ $[M + Na]^+$: 366.1465, found: 366.1470.

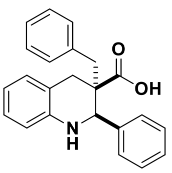

3-Methyl-8-oxo-1-azaspiro[4.5]deca-6,9-dien-1-ium-3-carboxylate (16): Prepared by the general procedure F from **15** (20.7 mg, 0.1 mmol) and isolated as a light pink powder (19.6 mg, 95% yield); Two isomers were obtained and the ratio of spiro-isomer and six membered-isomer was 85:15. **m.p.** 180–182 °C; **^{1}H NMR** (400 MHz, D_2O): δ 7.22 (dd, *J* = 10.0, 3.1 Hz, 1H), 7.16 (dd, *J* = 10.0, 3.1 Hz, 1H), 6.50 – 6.44 (m, 2H), 4.11 (d, *J* = 12.4 Hz, 1H), 3.45 (d, *J* = 12.0 Hz, 1H), 2.73 (d, *J* = 14.4 Hz, 1H),

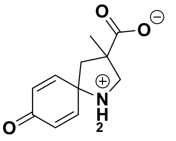

2.32 (d, *J* = 14.4 Hz, 1H), 1.47 (s, 3H); **^{13}C NMR** (151 MHz, DMSO-d_6): δ 185.30, 178.25, 154.45, 154.06, 125.06, 124.65, 61.00, 56.78, 48.59, 46.62, 39.52, 24.06; **IR** (KBr): ν 3445, 2878, 2757, 1686, 1638, 1548, 1393, 1264, 855 cm-1; **HRMS** (ESI) *m/z* calc'd for $C_{11}H_{14}O_3N$ $[M + H]^+$: 208.0968, found: 208.0971.

7-Hydroxy-3-methyl-1,2,3,4-tetrahydroquinoline-3-carboxylic acid (17): m.p. 148–150 °C; **^{1}H NMR** (600 MHz, CD_3OD): δ 6.71 (d, *J* = 8.4 Hz, 1H), 6.04 (dd, *J* = 8.4, 2.3 Hz, 1H), 5.99 (d, *J* = 1.8 Hz, 1H), 3.39 (d, *J* = 11.4 Hz, 1H), 3.01 (dd, *J* = 13.3, 8.2 Hz, 2H), 2.51 (d, *J* = 15.6 Hz, 1H), 1.22 (s, 3H); **^{13}C NMR** (151 MHz, CD_3OD): δ 180.46, 157.22, 145.83, 131.25, 112.29, 105.68, 101.58, 50.04, 41.32, 37.27, 22.60; **IR** (thin film): 3411, 2965, 2932, 2841, 1704, 1618, 1495, 1290, 1260, 1097, 1022, 958, 897, 799 cm^{-1}; **HRMS** (ESI) *m/z* calc'd for $C_{11}H_{14}O_3N$ $[M + H]^+$: 208.0968, found: 208.0971.

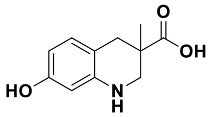

5-(4-hydroxyphenyl)-3-methylpyrrolidine-3-carboxylic acid (19): Prepared by a slight modification of the general procedure E, where $Rh_2(esp)_2$ (1 mol%) was employed as a catalyst instead of CuOTf/**L4**, and the reaction was conducted at 0 °C in HFIP. The title compound was isolated from **16** (22.1 mg, 0.1 mmol) as a white solid (21.1 mg, 95% yield, dr >20:1). **m.p.** 260–262 °C (decomp.); **^{1}H NMR** (400 MHz, D_2O): δ 7.42 – 7.39 (m, 2H), 6.99 – 6.96 (m, 2H), 4.71 (dd, *J* = 12.3, 6.3 Hz, 1H), 3.92 (d, *J* = 11.9 Hz, 1H), 3.24 (d, *J* = 11.9 Hz, 1H), 2.81 (dd, *J* = 13.5, 6.3 Hz, 1H), 2.21 (dd, *J* = 13.5, 12.4 Hz, 1H), 1.47 (s, 3H); **^{13}C NMR** (101 MHz, D_2O): δ 182.3, 156.8, 129.3, 125.7, 116.0, 62.5, 53.8, 50.8, 41.9, 22.7; **IR** (thin film): 3504, 3162, 2930, 2343, 1615, 1519, 1401, 1369, 1281, 1101, 880, 833, 685 cm^{-1}; **HRMS (ESI)** *m/z* calc'd for $C_{12}H_{16}O_3N$ $[M + H]^+$: 222.1125, found: 222.1127.

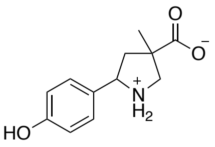

3-methyl-9-oxo-1-azaspiro[5.5]undeca-7,10-dien-1-ium-3-carboxylate (20): Prepared by the general procedure F from **16** (22.1 mg, 0.1 mmol) and isolated as a white solid (8.0 mg, 36% yield). **m.p.** 200-202 °C (decomp.); **^{1}H NMR** (400 MHz, D_2O): δ 7.70 (dd, *J* = 10.5, 3.3 Hz, 1H), 7.16 (dd, *J* = 10.2, 3.3 Hz, 1H), 6.57 (ddd, *J* = 20.7, 10.3, 2.0 Hz, 2H), 3.56 (dd, *J* = 13.3, 1.7 Hz, 1H), 3.25 (d, *J* = 13.3 Hz, 1H), 2.14-2.06 (m, 2H), 1.88-1.81 (m, 2H), 1.27 (s, 3H); **^{13}C NMR** (101 MHz, D_2O): δ 186.5, 182.2, 147.7, 142.5, 131.0, 130.5, 53.8, 47.2, 40.8, 30.6, 29.1, 22.7; **IR** (KBr): 3421, 2929, 2873, 1670, 1638, 1569, 1456, 1393, 1261, 1100, 860 cm^{-1}; **HRMS (ESI)** *m/z* calc'd for $C_{12}H_{16}O_3N$ $[M + H]^+$: 222.1125, found: 222.1126.

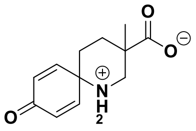

4-2. Mechanistic investigation

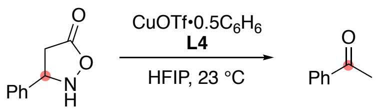

Procedure for Figure 1: Prior to the experiment, an NMR tube was dried under vacuum for 1 h. In a similar manner to the general procedure F, ^{13}C-**12** (8.2 mg, 0.05 mmol), $CuOTf•0.5C_6H_6$ (12 mg, 0.05 mmol), and **L4** (9.8 mg, 0.05 mmol) were mixed under an argon atmosphere to prepare a HFIP (500 μL) solution. The immediate consumption of the substrate was detected by a TLC analysis. A part of the HFIP solution (300 μL) was

transferred to the dried NMR tube by a syringe under an argon atmosphere, followed by the addition of THF-d_8 (300 μL), whose ^{13}C spectrum was recorded.

^{13}C-Acetophenone: **^{1}H NMR** (400 MHz, $CDCl_3$) δ 7.98 – 7.94 (m, 2H), 7.58 – 7.54 (m, 1H), 7.47 (t, J = 7.8 Hz, 2H), 2.61 (d, J = 6.0 Hz, 3H); **^{13}C NMR** (101 MHz, $CDCl_3$) δ 198.16; **HRMS** (ESI) *m/z* calc'd for $C_7{}^{13}CH_9O$ [M + H]$^+$: 122.0681, found: 122.0680.

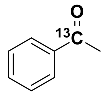

4-3. Kinetic study

The kinetic study was conducted under synthetically relevant conditions (0.1 M initial substrate concentration, 2.50–6.25 mol% catalyst loadings) to evaluate the copper dependency on the reaction rate. The reaction progression with varied copper concentrations were monitored over the course of the reaction. Only substrate **1a** and product **2a** were observed, and no intermediate was detected in ^{1}H-NMR analyses.

Procedure: Solutions of substrate **1a** (0.5 M in HFIP) and copper complex (0.05 M in HFIP, 1:1 CuOTf•0.5C_6H_6/**L4**) were prepared. To a flame-dried 10 mL test tube equipped with a magnetic stirring bar were added a solution of **1a** and HFIP, followed by the addition of a solution of the copper at an ambient temperature. The reaction mixture was quenched with aq EDTA and water at the indicated time. The aqueous layer was extracted with EtOAc (3x). The combined organic layers were concentrated under reduced pressure. The yields were determined by crude ^{1}H-NMR analysis using 1,1,2,2-tetrachloroethane as an internal standard.

catalyst loading	**1a** (0.5 M in HFIP)		Cu/**L4** (0.05 M in HFIP)		HFIP	Total volume
6.25 mol%	200 μL	0.1 M	125 μL	0.0625 M	675 μL	1000 μL
5.0 mol%	200 μL	0.1 M	100 μL	0.050 M	700 μL	1000 μL
3.75 mol%	200 μL	0.1 M	75 μL	0.0375 M	725 μL	1000 μL
2.5 mol%	200 μL	0.1 M	50 μL	0.025 M	750 μL	1000 μL

The following plots show the consumption of **1a** and the evolution of **2a** over the reaction.

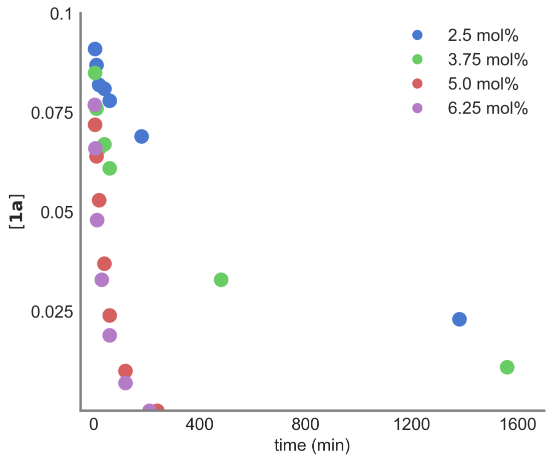

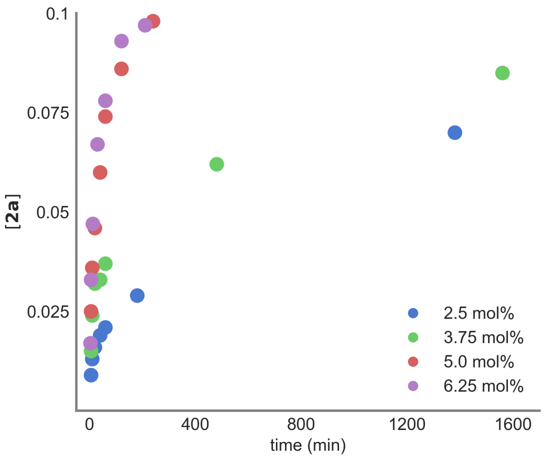

To evaluate the order of copper on the rate, the normalized time scale method developed by Burés was used.[8] Although it is not trivial to determine the exact order, the visual inspection of the plots suggests that more than one copper complex is involved in the catalytic cycle.

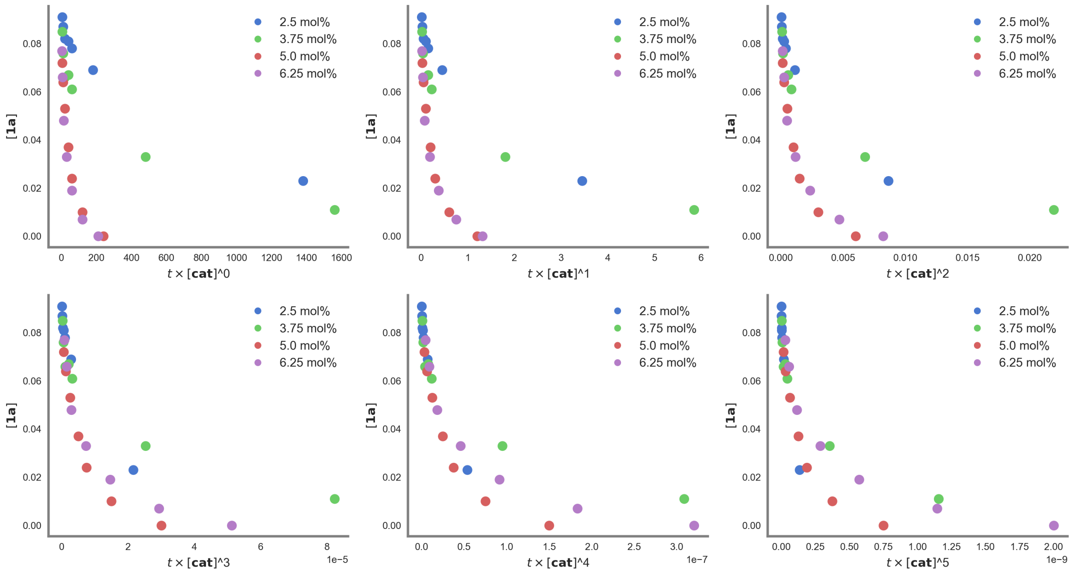

5. Catalytic asymmetric desymmetrization of 8

5-1. Catalyst screening

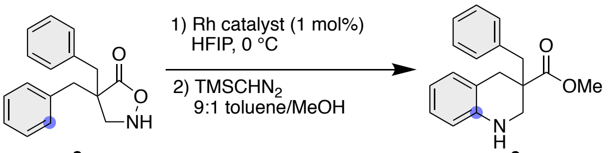

8 9

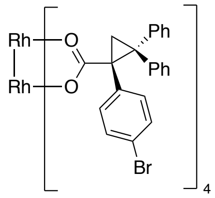

72 h, 86% yield, –3% ee

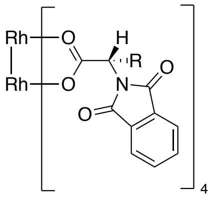

R = tert-butyl
72 h, 25% yield, –2% ee
R = 1-adamantyl
96 h, 22% yield, –7% ee

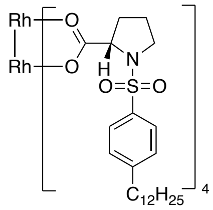

24 h, 92% yield, –11% ee

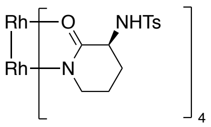

48 h, 0% yield, ND

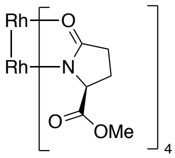

72 h, 0% yield, ND

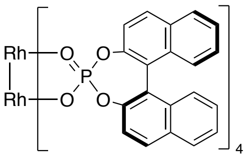

12 h, NR

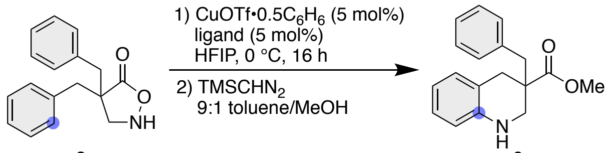

8 9

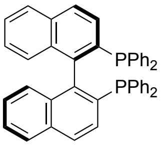

23% yield, 5% ee

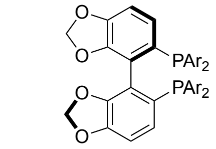

Ar = Ph: 36% yield, 11% ee
Ar = 3,5-*t*Bu-4-MeO-C_6H_2: trace

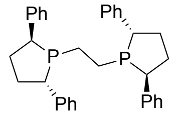

41% yield, –3% ee

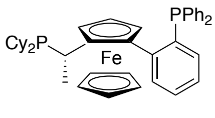

NR

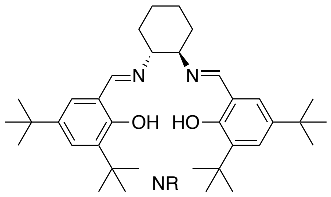

NR

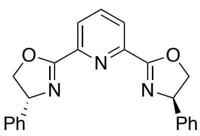

46% yield, –15% ee

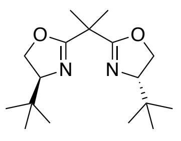

93% yield, 92% ee

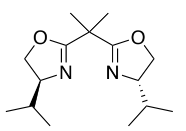

71% yield, 45% ee

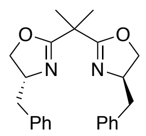

87% yield, –50% ee

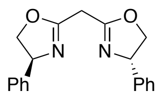

93% yield, 82% ee

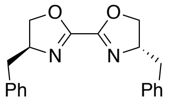

56% yield, –7% ee

5-2. Copper catalyzed asymmetric desymmetrization

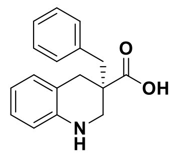

(*R*)-3-Benzyl-1,2,3,4-tetrahydroquinoline-3-carboxylic acid (S5): To a flame-dried 10 mL test tube equipped with a magnetic stirring bar were added (*S*)-*t*Bu-BOX (2.9 mg, 10 mol%) and Cu(OTf)•0.5C_6H_6 (90%, 1.4 mg, 5 mol%) in a globe box. After it was taken out, HFIP (1.0 mL, 0.1 M) was added and the mixture was stirred for 10 min at an ambient temperature. The solution was cooled to 0 °C, and **8** (26.7 mg, 0.1 mmol) was added. The reaction mixture was stirred for 18 h at the same temperature, and quenched with aq EDTA and water. The aqueous layer was extracted with EtOAc (3x). The combined organic layers were concentrated under reduced pressure. The material was purified and isolated as a white solid (24.5 mg, 92% yield). **m.p.** 98–100 °C; **^{1}H NMR** (400 MHz, $CDCl_3$): δ 7.29-7.27 (m, 1H), 7.25 – 7.24 (m, 2H), 7.18 – 7.16 (m, 2H), 7.01 (dd, *J* = 13.4, 7.0 Hz, 2H), 6.69 (td, *J* = 7.4, 1.1 Hz, 1H), 6.56 (dd, *J* = 7.9, 0.7 Hz, 1H), 3.49 (dd, *J* = 11.6, 1.4 Hz, 1H), 3.20 (dd, *J* = 11.6, 1.1 Hz, 1H), 3.10 (d, *J* = 16.4 Hz, 1H), 3.00 (s, 2H), 2.83 (d, *J* = 16.4 Hz, 1H); **^{13}C NMR** (101 MHz, $CDCl_3$): δ 179.41, 142.84, 136.47, 129.96, 129.89, 128.28, 127.12, 126.91, 119.16, 118.21, 114.44, 46.91, 45.43, 41.46, 34.90; **IR** (thin film): 3412, 3026, 2926, 2851, 1700, 1606, 1587, 1496, 1281, 1185, 939, 804, 746 cm^{-1}; **HRMS** (ESI) *m/z* calc'd for $C_{17}H_{18}O_2N$ [M + H]$^+$: 268.1332, found: 268.1335; $[\alpha]_D^{27}$ +52.79 (c 1.5, $CHCl_3$, 93% ee sample).

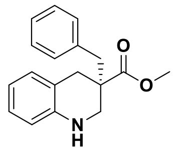

Methyl (*R*)-3-benzyl-1,2,3,4-tetrahydroquinoline-3-carboxylate (9): Isolated **S5** (24.5 mg, 0.09 mmol) was dissolved in 9:1 toluene/MeOH solution (0.90 mL, 0.1 M) and the reaction was cooled with an ice bath. TMS-diazomethane (2.0M in Et_2O, 1.5 equiv). After being stirred for 15 min, the reaction was quenched with aq AcOH, followed by the addition of aq $NaHCO_3$. The resulting aqueous layer was extracted with EtOAc (3x). The combined organic layers were concentrated under reduced pressure to give the crude product, which was purified by silica gel column chromatography to afford product **9** as a white solid (24.4 mg, 95% yield). **m.p.** 73–75 °C; **^{1}H NMR** (400 MHz, $CDCl_3$): δ 7.28 – 7.26 (m, 1H), 7.25 – 7.20 (m, 2H), 7.10 – 7.07 (m, 2H), 7.02 - 6.97 (m, 2H), 6.65 (td, *J* = 7.4, 1.1 Hz, 1H), 6.53 (d, *J* = 7.9 Hz, 1H), 3.63 (s, 3H), 3.48 (dd, *J* = 11.6, 1.1 Hz, 1H), 3.22 (dd, *J* = 11.6, 1.6 Hz, 1H), 3.08 (d, *J* = 16.2 Hz, 1H), 2.96 (dd, *J* = 42.8, 13.4 Hz, 2H), 2.81 (d, *J* = 16.2 Hz, 1H); **^{13}C NMR** (101 MHz, $CDCl_3$): δ 175.15, 143.20, 136.98, 129.90, 129.80, 128.18, 127.01, 126.73, 119.04, 117.63, 114.06, 51.76, 46.94, 45.70, 41.32, 34.85; **IR** (thin film): 3407, 3026, 2949, 2879, 1727, 1607, 1587, 1496, 1439, 1366, 1282, 1092, 748 cm^{-1}; **HRMS** (ESI) *m/z* calc'd for $C_{18}H_{20}O_2N$ [M + H]$^+$: 282.1489, found: 282.1491; $[\alpha]_D^{27}$ +112.01 (c 1.0, $CHCl_3$, 93% ee sample). Enantiomeric excess of the product was determined to be 93% ee by chiral stationary phase HPLC analysis (CHIRALPAK IA (ϕ 0.46 cm x 25 cm), *n*-hexane/2-propanol = 8/2, flow rate 1.0 mL/min, detection at 254 nm, t_R = 6.19 min (major), 10.45 min (minor).

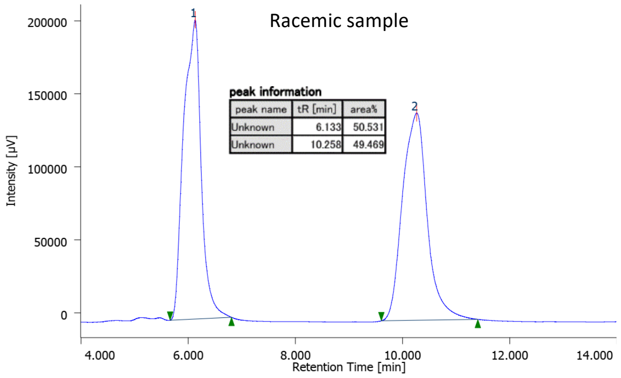

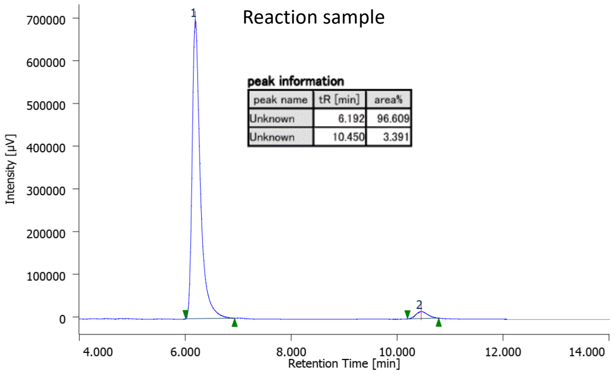

Single crystals of **9** were obtained by slow diffusion of the solution of **9** in $CHCl_3$ at 23 °C. A suitable crystal was selected and the sample was measured on a Rigaku R-AXIS RAPID diffractometer using graphite monochromated Cu-Ka radiation. The data were collected at 93.15 K. Refined structure and crystallographic parameters are summarized in Fig. S1 and Table S1. CCDC 2049408 contains the supplementary crystallographic data for **9**.

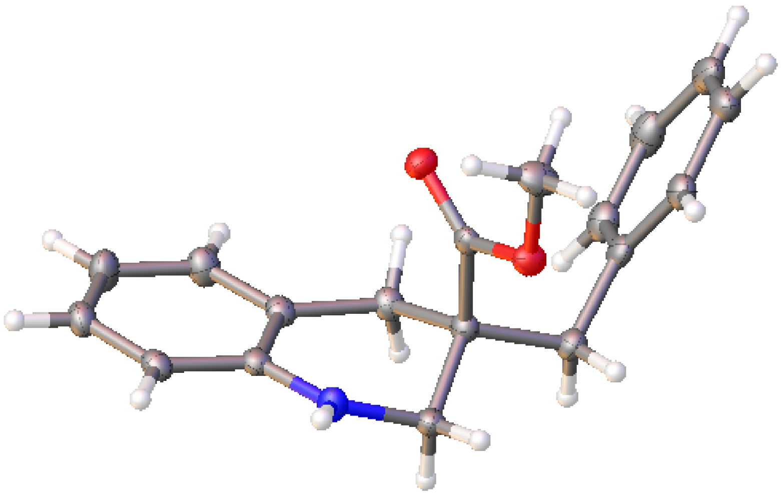

Fig. S1. ORTEP diagram of **9**.

Table S1. Crystal data and structure refinement for **9**.

Empirical formula	$C_{36}H_{38}N_2O_4$	Volume/Å^3	1489.33(2)
Formula weight	562.68	Z	2
Temperature/K	93.15	ρ_{calc}g/cm^3	1.255
Crystal system	monoclinic	μ/mm^{-1}	0.648
Space group	$P2_1$	F(000)	600.0
a/Å	10.47410(10)	Crystal size/mm^3	0.3 × 0.1 × 0.05
b/Å	10.48660(10)	Radiation	CuKα (λ = 1.54184)
c/Å	13.73150(10)	Flack parameter	-0.02(6)
α/°	90		
β/°	99.0810(10)		
γ/°	90		

5-3. Nonlinear effect

The experiments were performed by a slight modification of the procedure described above. (*R*)- and (*S*)-*t*Bu-BOX ligands were weighted to prepare target ee ligand (20–80% ee). The weighted ligand was dissolved in HFIP before complexation with a copper, and an aliquot was removed to determine the accurate ee value of the mixed ligand by a chiral HPLC. Two series of experiments were conducted, and both results showed a positive nonlinear effect.

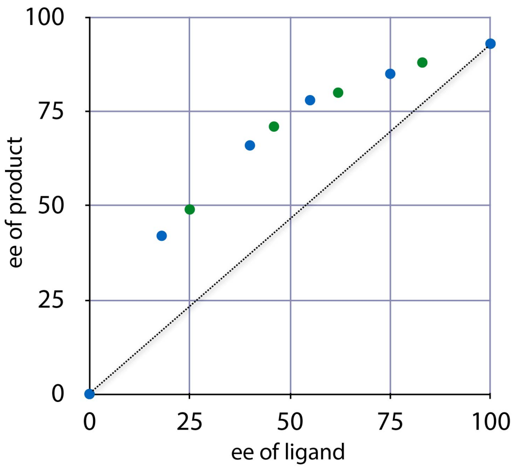

6. Computational study

6-1. Computational details

All quantum chemical calculations were performed using the Gaussian 16 program.[9] Density functional theory (DFT) calculations employed an ultrafine integration grid (99 radial shells, 590 angular points). Structural optimizations were conducted in the gas phase at the B3LYP-D3(BJ)[10]/6-31G(d)(SDD for Cu) level of theory. Frequency calculations confirmed the identity of geometry minima (no imaginary frequencies) and transition state (one imaginary frequency). All transition state structures were verified to connect the reactant and the product of interest by performing IRC calculations. Single point energies on the optimized structures were evaluated using Truhlar's M06 functional[11] with the 6-311++G(2d,p) basis set for C, H, N, and O atoms and the SDD effective core potential for Cu. Single point solvation energies were obtained by using SMD[12] solvation model (2-methyl-1-propanol) to model HFIP. Gibbs free energies are given relative to a dicopper triplet nitrene. Zero-point energies and thermal corrections were obtained at 298K and are unscaled. Computed structures were visualized by CYLview 2.0.[13]

6-2. Evaluation of interaction energies

The interaction energies between two copper complexes were estimated by computing counterpoise corrected energies on the truncated structures.

^{3}NTR_D

Counterpoise corrected energy = -1687.322851787333
BSSE energy = -0.002216308473
sum of fragments = -1687.256769441415
complexation energy = -40.08 kcal/mole (raw)
complexation energy = -41.47 kcal/mole (corrected)

^{3}TS2_D

Counterpoise corrected energy = -1687.315509706308
BSSE energy = 0.015924594258
sum of fragments = -1687.225498624053
complexation energy = -66.48 kcal/mole (raw)
complexation energy = -56.48 kcal/mole (corrected)

7. References

(1) Fulmer, G. R.; Miller, A. J. M.; Sherden, N. H.; Gottlieb, H. E.; Nudelman, A.; Stoltz, B. M.; Bercaw, J. E.; Goldberg, K. I. *Organometallics*, **2010**, *29*, 2176.

(2) Yu, J.-S.; Espinosa, M.; Noda, H.; Shibasaki, M. *J. Am. Chem. Soc*. **2019**, *141*, 10530–10537.

(3) Espinosa, M.; Noda, H.; Shibasaki, M. *Org. Lett*. **2019**, *21*, 9296−9299.

(4) Singh, R. P.; Das, J.; Yousufuddin, M.; Gout, D.; Lovely, C. J. *Org. Lett.* **2017**, *19*, 4110–4113.

(5) Baldwin, J. E.; Harwood, L. M.; Jombard, M. J. *Tetrahedron* **1984**, *40*, 4363–4370.

(6) Noda, H.; Shibasaki, M. *Chem. Pharm. Bull.* **2019**, *67*, 1248–1249.

(7) Lakshmana, M. K.; Tine, F. A.; Khandaker, T. A.; Basava, V.; Agyemang, N. B.; Benavidez, M. S. A.; Gaši, M.; Guerrera, L.; Zajc, B. *Synlett* **2017**, 381–385.

(8) Burés, J. *Angew. Chem. Int. Ed.* **2016**, *55*, 2028–2031.

(9) Gaussian 16, Revision A.03, M. J. Frisch, G. W. Trucks, H. B. Schlegel, G. E. Scuseria, M. A. Robb, J. R. Cheeseman, G. Scalmani, V. Barone, G. A. Petersson, H. Nakatsuji, X. Li, M. Caricato, A. V. Marenich, J. Bloino, B. G. Janesko, R. Gomperts, B. Mennucci, H. P. Hratchian, J. V. Ortiz, A. F. Izmaylov, J. L. Sonnenberg, D. Williams-Young, F. Ding, F. Lipparini, F. Egidi, J. Goings, B. Peng, A. Petrone, T. Henderson, D. Ranasinghe, V. G. Zakrzewski, J. Gao, N. Rega, G. Zheng, W. Liang, M. Hada, M. Ehara, K. Toyota, R. Fukuda, J. Hasegawa, M. Ishida, T. Nakajima, Y. Honda, O. Kitao, H. Nakai, T. Vreven, K. Throssell, J. A. Montgomery, Jr., J. E. Peralta, F. Ogliaro, M. J. Bearpark, J. J. Heyd, E. N. Brothers, K. N. Kudin, V. N. Staroverov, T. A. Keith, R. Kobayashi, J. Normand, K. Raghavachari, A. P. Rendell, J. C. Burant, S. S. Iyengar, J. Tomasi, M. Cossi, J. M. Millam, M. Klene, C. Adamo, R. Cammi, J. W. Ochterski, R. L. Martin, K. Morokuma, O. Farkas, J. B. Foresman, and D. J. Fox, Gaussian, Inc., Wallingford CT, 2016.

(10) Grimme, S.; Ehrlich, S.; Goerigk, L. *J. Comp. Chem.* **2011**, *32*, 1456–1465.

(11) Zhao, Y.; Truhlar, D. G. *Theor. Chem. Acc.* **2007**, *120*, 215–241.

(12) Marenich, A. V.; Cramer, C. J.; Truhlar, D. G. *J. Phys. Chem. B* **2009**, *113*, 6378–6396.

(13) CYLview20; Legault, C. Y., Université de Sherbrooke, 2020 (http://www.cylview.org). Accessed on 16-Dec-2020.

8. Optimized coordinates

^{3}NTR_M

Zero-point correction= 0.401071
(Hartree/Particle)
Thermal correction to Energy= 0.427785
Thermal correction to Enthalpy= 0.428729
Thermal correction to Gibbs Free Energy= 0.339202

C	-3.82523700	1.98193500	-0.62036300
C	-2.48165300	1.80552400	-0.25390000
C	-1.62565800	2.90205300	-0.12945300
C	-2.18353700	4.14998300	-0.39695200
C	-3.53438700	4.30388200	-0.77036300
C	-4.39782300	3.21298800	-0.88980600
C	-3.44387100	-0.12799200	-0.31541200
H	-0.59462900	2.78338000	0.18422700
H	-1.56094800	5.03436100	-0.31061700
H	-3.91774700	5.29966600	-0.96702800
H	-5.43867500	3.31990900	-1.17117700
N	-2.28443600	0.43437200	-0.07173500
O	-4.42318700	0.72854400	-0.65107500
C	-3.65696600	-1.56500400	-0.23307300
C	-4.87719500	-2.19203000	-0.46436300
C	-2.59499900	-3.57617500	0.20817500
C	-4.93342400	-3.58224600	-0.34599600
H	-5.74951900	-1.60483200	-0.72729700
C	-3.77959200	-4.28513700	-0.00492400
H	-1.67213600	-4.08116700	0.47306500
H	-5.86785800	-4.10603600	-0.51854900
H	-3.78658000	-5.36450100	0.09578300
N	-2.53126300	-2.24509000	0.09761400
Cu	-0.88422300	-0.97270000	0.32112300
O	2.06754700	0.82177700	2.96596300
C	1.80089800	1.33775100	1.74662800
C	2.58543600	0.63634500	0.64028700
C	2.23255600	-0.88834100	0.69935100
H	2.78101600	-1.40327100	-0.10817200
H	2.58418600	-1.31868500	1.65137900
O	1.01896800	2.25178200	1.58775500
N	0.86251900	-1.12786100	0.57213200
C	4.11297900	0.81610600	0.92939000
H	4.31720000	1.89324300	0.91098300
H	4.31359300	0.47047700	1.94756900
C	5.01716300	0.10250500	-0.04705300
C	5.46054300	-1.20136600	0.21746700
C	5.42674000	0.72019800	-1.23678000
C	6.27450900	-1.87872500	-0.69113500
H	5.18215300	-1.68251400	1.15229200
C	6.24149000	0.04699300	-2.14648600
H	5.11553000	1.74046400	-1.44472600
C	6.66333800	-1.25683700	-1.87831400
H	6.61427200	-2.88523200	-0.46564600
H	6.55465900	0.54361100	-3.06001100
H	7.30211700	-1.77923200	-2.58390600
H	1.54899300	1.33651900	3.61399300
C	2.18771800	1.21615800	-0.71892000
H	2.42076900	2.28327000	-0.76339000
H	2.73236300	0.70793800	-1.51831900
H	1.11516700	1.10276400	-0.89828400

^{3}TS1_M

Zero-point correction= 0.399497
(Hartree/Particle)
Thermal correction to Energy= 0.425728
Thermal correction to Enthalpy= 0.426672
Thermal correction to Gibbs Free Energy= 0.338173

C	-3.67823200	-1.77235700	-0.34038800
C	-2.27694500	-1.81708100	-0.40115700
C	-1.60400300	-3.04023200	-0.39216600
C	-2.39340700	-4.18544100	-0.32178700
C	-3.80048600	-4.11577400	-0.26373000
C	-4.48292700	-2.89721100	-0.27202400
C	-2.89645500	0.24605900	-0.42291600
H	-0.52159700	-3.08500000	-0.43917400
H	-1.91671800	-5.16004500	-0.31356100
H	-4.37189900	-5.03651200	-0.21153900
H	-5.56384300	-2.83547700	-0.22780700
N	-1.82290700	-0.50137500	-0.45691000
O	-4.05677200	-0.43704800	-0.35653700
C	-2.83771700	1.70006300	-0.42619700
C	-3.95373000	2.53128400	-0.42428300
C	-1.37647500	3.49364100	-0.42362600
C	-3.74341300	3.91162000	-0.42286400
H	-4.95051000	2.10520300	-0.42520800
C	-2.43894300	4.40135000	-0.42291700
H	-0.34467200	3.82908600	-0.42165200
H	-4.58875000	4.59184800	-0.42250300
H	-2.23775400	5.46670000	-0.42212000
N	-1.56626900	2.17095200	-0.42437100

Cu	-0.13049600	0.62826200	-0.41621600
O	5.64076700	1.12638800	-0.70179700
C	5.29015600	-0.14076700	-1.03717100
C	3.87930300	-0.48720600	-0.56584100
C	2.84845000	0.55570700	-1.07379800
H	3.25603200	1.57167000	-0.95402700
H	2.62126000	0.41075400	-2.13908500
O	6.02660200	-0.88720300	-1.63674700
N	1.66170900	0.43004300	-0.28775800
C	3.78150500	-0.35299700	0.97971300
H	4.20567900	-1.23810600	1.46375500
H	4.36981700	0.50951400	1.30195800
C	2.33102600	-0.15625700	1.41424700
C	2.00326300	1.02003900	2.19566500
C	1.50934400	-1.32554900	1.66334600
C	0.80980800	1.10683300	2.88307300
H	2.68839700	1.86214600	2.16772700
C	0.32011700	-1.22152100	2.35258300
H	1.82567900	-2.27954100	1.25616300
C	-0.06419100	0.00358200	2.93417700
H	0.55641000	2.01936500	3.41450700
H	-0.31198300	-2.09598300	2.47551500
H	-0.99776900	0.07459700	3.48334800
H	6.55077800	1.25946400	-1.03004500
C	3.51476800	-1.88860600	-1.06854500
H	3.65721300	-1.95473400	-2.15032900
H	4.15207700	-2.64756700	-0.60726200
H	2.46912100	-2.11649500	-0.84484200

^{3}INT1_M

Zero-point correction= (Hartree/Particle)	0.400817
Thermal correction to Energy=	0.427127
Thermal correction to Enthalpy=	0.428071
Thermal correction to Gibbs Free Energy=	0.339480

C	-3.82430700	-1.89861800	0.06545800
C	-2.42620100	-2.01818100	0.05219200
C	-1.81766800	-3.25675700	0.26287300
C	-2.66649600	-4.33887700	0.48127300
C	-4.06893300	-4.19416900	0.48960900
C	-4.68736000	-2.95973600	0.27994100
C	-2.94251000	0.04409700	-0.30950400
H	-0.73816900	-3.36322800	0.25437200
H	-2.24044100	-5.32234100	0.64943200
H	-4.68777900	-5.06802700	0.66385100
H	-5.76424200	-2.83976800	0.28424000
N	-1.90526800	-0.74810400	-0.19135800
O	-4.13412800	-0.56583900	-0.16876900
C	-2.81323300	1.47244800	-0.55837100
C	-3.88177800	2.35762600	-0.65896200
C	-1.25414800	3.15666500	-0.86927300
C	-3.59463600	3.70682600	-0.87860200
H	-4.89999400	1.99786700	-0.56480500
C	-2.26607700	4.11417700	-0.98186900
H	-0.20417000	3.42163100	-0.93194100
H	-4.40100000	4.42778900	-0.96431800
H	-2.00850400	5.15413700	-1.14808700
N	-1.52092100	1.86384700	-0.66837500
Cu	-0.17444100	0.28086200	-0.42027500
O	5.46742100	-0.26045400	-1.49894700
C	4.99548300	-1.37499400	-0.88520400
C	3.64713300	-1.13068800	-0.21838100
C	2.61719800	-0.53344100	-1.21236500
H	3.10410900	0.20323000	-1.86854900
H	2.13547300	-1.28589500	-1.84281400
O	5.59270700	-2.42490800	-0.88109700
N	1.64518500	0.13562100	-0.37176000
C	3.73546300	-0.00005200	0.84410500
H	3.94867200	-0.39981800	1.83832100
H	4.52984800	0.70536100	0.59214400
C	2.35224800	0.73112100	0.81027600
C	2.50123600	2.20942900	0.53175600
C	1.49340000	0.46851300	2.02136400
C	1.81725900	3.16392800	1.22838100
H	3.17792100	2.48111000	-0.27367800
C	0.82093600	1.46255100	2.68084400
H	1.41438600	-0.56549100	2.34375900
C	0.94670800	2.81919100	2.29546200
H	1.96530600	4.21241800	0.98214500
H	0.20112900	1.20873500	3.53656700
H	0.41871800	3.59311100	2.84175200
H	6.33030200	-0.50077300	-1.88764100
C	3.12484200	-2.44117200	0.38005800
H	3.03425400	-3.21141300	-0.39112600
H	3.80955800	-2.81794500	1.14374400
H	2.14107900	-2.28492300	0.83517900

^{3}TS2_M

Zero-point correction= 0.400537
(Hartree/Particle)
Thermal correction to Energy= 0.426414
Thermal correction to Enthalpy= 0.427358
Thermal correction to Gibbs Free Energy= 0.341119

C	3.06855900	2.21963400	-0.53391600
C	1.70331300	1.90948200	-0.43590400
C	0.74601400	2.92599300	-0.42641500
C	1.22108800	4.23163100	-0.51873200
C	2.59808600	4.51795000	-0.61683100
C	3.56375200	3.50946700	-0.62700400
C	2.82824600	0.07125100	-0.40827900
H	-0.31021100	2.70107900	-0.34617900
H	0.51414800	5.05474600	-0.51778300
H	2.91822300	5.55210700	-0.68781200
H	4.62412400	3.71916600	-0.70283500
N	1.59925000	0.52072100	-0.36077600
O	3.77373800	1.02484000	-0.51488000
C	3.15205000	-1.34553000	-0.33619000
C	4.44240800	-1.86038400	-0.41471400
C	2.21853900	-3.45074300	-0.10432400
C	4.60245100	-3.24493800	-0.33142400
H	5.28819100	-1.19378100	-0.53843400
C	3.47765200	-4.05230300	-0.17484500
H	1.31524800	-4.03915600	0.01764800
H	5.59319600	-3.68350500	-0.38947800
H	3.56383400	-5.13091300	-0.10739400
N	2.05450000	-2.12630700	-0.18163400
Cu	0.27969500	-1.01532700	-0.11324800
O	-5.53629500	-1.43835200	-0.82978100
C	-4.86879200	-0.40858800	-1.40463200
C	-3.56044000	-0.09924200	-0.67637800
C	-2.71393700	-1.41343400	-0.59974900
H	-3.32174000	-2.20137700	-0.13282400
H	-2.47574000	-1.74443300	-1.62041900
O	-5.28334400	0.18603800	-2.37126000
N	-1.49647300	-1.23913700	0.13307600
C	-3.90942000	0.38449900	0.77300000
H	-4.41211200	1.35477300	0.70523100
H	-4.61450500	-0.32577500	1.21717900
C	-2.66778000	0.47949800	1.60891900
C	-2.01345200	-0.76156800	1.94661500
C	-2.02239100	1.67515100	1.88116200
C	-0.80665100	-0.71696700	2.72155200
H	-2.63815000	-1.64701300	2.04856300
C	-0.78389600	1.69565700	2.54345900
H	-2.48846000	2.61061400	1.58292700
C	-0.18564800	0.49412800	2.96687200
H	-0.38215200	-1.64582100	3.09013700
H	-0.29963300	2.64409700	2.75232500
H	0.74531700	0.52507100	3.52537900
H	-6.35980800	-1.55968500	-1.34063400
C	-2.80080700	0.97958300	-1.45303500
H	-2.58277500	0.64381600	-2.47090600
H	-3.39768500	1.89192600	-1.52985800
H	-1.85788800	1.21003600	-0.95318900

^{3}INT2_M

Zero-point correction= 0.402084
(Hartree/Particle)
Thermal correction to Energy= 0.427925
Thermal correction to Enthalpy= 0.428870
Thermal correction to Gibbs Free Energy= 0.342863

C	2.95007000	2.21143200	-0.73076100
C	1.61606000	1.89377600	-0.43112200
C	0.64827100	2.89688400	-0.34893700
C	1.08084700	4.19883800	-0.58710900
C	2.42530600	4.49330900	-0.89293500
C	3.40177700	3.49801200	-0.97113000
C	2.77163600	0.07352700	-0.43572400
H	-0.38004300	2.66520400	-0.09961500
H	0.36565600	5.01318100	-0.53605800
H	2.71200100	5.52436400	-1.07117500
H	4.43792700	3.71544500	-1.20173500
N	1.55101700	0.51216500	-0.25737700
O	3.67563200	1.02795900	-0.72780400
C	3.12532500	-1.33170500	-0.30568200
C	4.40578100	-1.84072900	-0.49817100
C	2.26196000	-3.42262400	0.19006000
C	4.59853100	-3.21364300	-0.33242800
H	5.21967100	-1.17795100	-0.76894500
C	3.51437100	-4.01659600	0.01606400
H	1.39015900	-4.00831400	0.46187300
H	5.58317400	-3.64673900	-0.47446800
H	3.62671900	-5.08610600	0.15327900
N	2.06656900	-2.10902100	0.03424800
Cu	0.29702900	-1.02311200	0.20794300
O	-5.34756900	-1.58155500	-0.98163600

C	-4.56260100	-0.71102400	-1.66142100
C	-3.35341900	-0.26886700	-0.83809500
C	-2.56946900	-1.54324300	-0.38164800
H	-3.26792300	-2.22759100	0.11807100
H	-2.15181000	-2.05259500	-1.25427800
O	-4.81350500	-0.33705900	-2.78274800
N	-1.49835700	-1.19914200	0.53281800
C	-3.84981900	0.48115500	0.43852400
H	-4.29810200	1.43749000	0.14934900
H	-4.63585300	-0.12290500	0.91015000
C	-2.72105200	0.68729700	1.39995000
C	-1.99408000	-0.58729000	1.78737600
C	-2.27777800	1.90345600	1.83955400
C	-0.85869500	-0.38008700	2.74521100
H	-2.73011000	-1.29778300	2.21277500
C	-1.16115300	2.02799900	2.70540200
H	-2.80464400	2.80105300	1.52282500
C	-0.47559700	0.87061300	3.14868600
H	-0.36535200	-1.26999100	3.12742500
H	-0.84522200	3.00918000	3.04216100
H	0.35633400	0.98052700	3.83889400
H	-6.09830300	-1.79935300	-1.56710000
C	-2.46611800	0.63409200	-1.69970800
H	-2.11166300	0.10049000	-2.58653300
H	-3.02748900	1.50574400	-2.04530300
H	-1.60096800	0.97663200	-1.12798000

^{3}TS3_M

Zero-point correction= 0.400303 (Hartree/Particle)

Thermal correction to Energy= 0.425497

Thermal correction to Enthalpy= 0.426442

Thermal correction to Gibbs Free Energy= 0.343365

C	-3.88577200	-0.52820800	0.41017700
C	-2.68389000	-1.16758600	0.06845600
C	-2.62913300	-2.55838800	-0.04296900
C	-3.81277600	-3.25172100	0.19820900
C	-5.00806000	-2.58685600	0.53847500
C	-5.07342600	-1.19663800	0.65417600
C	-2.33613600	0.95964800	0.13334600
H	-1.70844300	-3.06722100	-0.30377200
H	-3.81862000	-4.33402600	0.12227900
H	-5.90470000	-3.17124000	0.71602400
H	-5.98748700	-0.67664600	0.91550500
N	-1.72321100	-0.17325800	-0.10323400
O	-3.64079500	0.83698300	0.44772800
C	-1.66169200	2.24545300	0.05231500
C	-2.27688600	3.47962200	0.24207600
C	0.38909400	3.21914300	-0.39648300
C	-1.49424400	4.62733100	0.10113100
H	-3.33172600	3.53336800	0.48602700
C	-0.14539500	4.49924600	-0.22716400
H	1.42947900	3.05883800	-0.66014400
H	-1.93681300	5.60818300	0.24051500
H	0.48727000	5.37067900	-0.35320700
N	-0.34952800	2.11898800	-0.25167800
Cu	0.27382900	0.09285400	-0.40155800
O	2.21947100	-1.34671900	3.29352800
C	2.04345800	-0.59415600	2.20161100
C	3.30482700	-0.46160700	1.35422600
C	3.14811100	0.75265500	0.41168700
H	2.72352000	1.62556200	0.91393500
H	4.13287400	1.03725100	0.01997200
O	0.95152200	-0.11567000	1.92183000
N	2.25328500	0.33201900	-0.67975500
C	3.30593000	-1.64972300	0.33754200
H	4.33328700	-1.82255400	0.00100400
H	2.94718100	-2.58114000	0.78238500
C	2.41599600	-1.17966800	-0.81253000
C	1.05021400	-1.77435900	-0.99435200
C	3.01453400	-0.51216300	-1.98566400
C	0.49836600	-1.78977600	-2.33340800
H	0.76230800	-2.56687700	-0.30794700
C	2.36746700	-0.52218000	-3.25213100
H	4.06498900	-0.24473900	-1.92340700
C	1.11401800	-1.15718600	-3.39991900
H	-0.44671600	-2.30268900	-2.49064600
H	2.84438300	-0.03140300	-4.09297500
H	0.63746700	-1.16872100	-4.37497200
H	1.35832100	-1.40669000	3.75191400
C	4.56993200	-0.40667700	2.21680100
H	4.54721500	0.44625800	2.90288000
H	4.68468500	-1.31441000	2.81284300
H	5.44785000	-0.30278400	1.57131200

^{3}NTR_D

Zero-point correction= 0.581506 (Hartree/Particle)

Thermal correction to Energy= 0.621282
Thermal correction to Enthalpy= 0.622226
Thermal correction to Gibbs Free Energy= 0.502733

C	2.74553400	0.23419500	2.23033500
C	1.50940000	-0.32380700	1.86838000
C	1.25774400	-1.68211800	2.06965200
C	2.29875400	-2.43604400	2.60886100
C	3.54057100	-1.85842600	2.94074800
C	3.79407000	-0.49491600	2.76431800
C	1.47520500	1.78965900	1.41644700
H	0.29746500	-2.11923700	1.82238400
H	2.14947100	-3.49597600	2.78572200
H	4.31836700	-2.48512100	3.36406900
H	4.73839000	-0.03875300	3.03700600
N	0.73317200	0.70971800	1.33958100
O	2.69374900	1.59313700	1.94445900
C	1.00936100	3.08338400	0.94737800
C	1.68312800	4.28493400	1.12969400
C	-0.74366400	4.11408500	-0.16778100
C	1.09085000	5.44915400	0.63272300
H	2.63540400	4.30351700	1.64702600
C	-0.13712300	5.36467100	-0.02152500
H	-1.69735500	3.99096200	-0.66775300
H	1.58199800	6.40818400	0.76096300
H	-0.62597600	6.24896100	-0.41465900
N	-0.18115300	2.99636000	0.30362000
Cu	-0.86253200	1.04093100	0.09084600
O	-4.54757500	1.23619900	-1.66621300
C	-3.55209500	0.85353700	-0.90099600
C	-3.56523700	-0.58360800	-0.42508700
C	-2.29359600	-1.25058400	-1.05447700
H	-2.31094500	-2.30412600	-0.73555300
H	-2.39926100	-1.26933200	-2.15232900
O	-2.63460300	1.65705100	-0.63641600
N	-1.02121700	-0.68525400	-0.71305200
C	-4.80866500	-1.38769200	-0.91153700
H	-4.56699400	-2.44237300	-0.73250200
H	-4.89845400	-1.27155700	-1.99656200
C	-6.13178700	-1.07507900	-0.24101200
C	-7.06575400	-0.21764900	-0.83629900
C	-6.46438700	-1.68354800	0.97823800
C	-8.28597900	0.04915800	-0.21472400
H	-6.84692100	0.22895300	-1.80051600
C	-7.68038100	-1.41473700	1.60500900
H	-5.77584900	-2.39116900	1.43352000
C	-8.59320800	-0.54113500	1.01165100
H	-9.00242400	0.70795400	-0.69604900
H	-7.92157100	-1.89959100	2.54602700
H	-9.54479700	-0.33739400	1.49255200
H	-4.42799000	2.17383400	-1.92176500
C	-3.45126100	-0.59592200	1.11288500
H	-4.31638300	-0.11929500	1.57727800
H	-3.41317500	-1.63355300	1.45782600
H	-2.54458500	-0.09394500	1.46741700
Cu	0.66877800	-1.26354200	-1.11361000
C	1.39023200	-4.28150100	-0.70115400
C	3.12691500	-2.75497000	-0.76390200
C	2.27582100	-5.32677400	-0.42495700
H	0.32333600	-4.45684400	-0.79234200
C	4.07707700	-3.73214000	-0.48662100
C	3.63645800	-5.04621400	-0.31336500
H	1.89776500	-6.33579800	-0.30389200
H	5.12637300	-3.46876700	-0.41709300
H	4.34794800	-5.83741000	-0.10092700
C	3.20631100	0.75768000	-1.36935400
C	3.43833900	-1.34853100	-0.96851800
C	4.53972400	0.51842300	-1.00251500
C	2.78021800	2.04803900	-1.68945400
C	5.50529900	1.50705600	-0.91555400
C	3.73460700	3.06043200	-1.60636300
H	1.76063000	2.24220900	-2.00548300
C	5.06581600	2.79694600	-1.22511900
H	6.53099100	1.29448800	-0.63767300
H	3.45283100	4.07792100	-1.85657300
H	5.77592300	3.61612300	-1.18493000
N	1.80075300	-3.01963200	-0.86356800
N	2.53871600	-0.46637200	-1.33151900
O	4.66755200	-0.84096700	-0.75639100

^{3}TS1_D

Zero-point correction= 0.579639
(Hartree/Particle)
Thermal correction to Energy= 0.618924
Thermal correction to Enthalpy= 0.619868
Thermal correction to Gibbs Free Energy= 0.504636

C	-2.58445600	-0.13700800	-2.26899200
C	-1.33714100	-0.77726400	-2.18423800
C	-1.24121700	-2.15873300	-2.37274000

C	-2.42663200	-2.84342600	-2.62845500
C	-3.66798200	-2.17754000	-2.69704700
C	-3.77735100	-0.79603200	-2.51993500
C	-1.03626000	1.31751200	-1.82819400
H	-0.28445200	-2.66710700	-2.32877800
H	-2.39772100	-3.91582200	-2.78965400
H	-4.56378300	-2.75166700	-2.90966800
H	-4.72530200	-0.27516900	-2.58753300
N	-0.37432400	0.19323500	-1.89729300
O	-2.37320000	1.21233700	-2.04552700
C	-0.45378300	2.60790900	-1.48872000
C	-1.11631300	3.81632200	-1.69350400
C	1.40900100	3.68529600	-0.62264700
C	-0.46669800	4.99897900	-1.34349000
H	-2.11166400	3.81895500	-2.12182000
C	0.81816800	4.93448200	-0.80373800
H	2.40402000	3.58698300	-0.20219300
H	-0.95353400	5.95639500	-1.49744400
H	1.35950200	5.83232900	-0.52736400
N	0.78851000	2.54138500	-0.94798600
Cu	1.54126800	0.71189200	-0.61221200
O	5.50619200	-0.06037100	-1.85352700
C	4.28187100	-0.21122700	-1.37599500
C	4.10093200	-1.49375500	-0.56791400
C	2.61204800	-1.89585500	-0.54474200
H	2.18400100	-1.94657500	-1.55514400
H	2.54283500	-2.90136100	-0.10404400
O	3.42151600	0.65516400	-1.57290900
N	1.89998600	-0.92946700	0.25650300
C	4.40052800	-1.18223700	0.93390900
H	5.30176000	-0.57102500	1.03922300
H	4.61033100	-2.13914100	1.42147200
C	3.20669300	-0.51260300	1.61068100
C	2.55343700	-1.19293700	2.71124200
C	3.16633500	0.93828900	1.65679400
C	1.67683300	-0.51829000	3.54282600
H	2.70668300	-2.26231900	2.82331900
C	2.30011300	1.59671800	2.50665000
H	3.82978200	1.49941100	1.00544500
C	1.51016900	0.87499400	3.42444200
H	1.14819600	-1.05871100	4.32211500
H	2.26020700	2.68222200	2.50389200
H	0.83364300	1.39898500	4.09131300
H	5.55882700	0.78464900	-2.34508300
C	5.00242100	-2.61440400	-1.10350800
H	6.05867600	-2.35443400	-1.01077000
H	4.82224000	-3.52984400	-0.53201900
H	4.79769300	-2.82100800	-2.15844300
Cu	0.13877800	-0.87347400	0.87371200
C	-0.86109300	-3.91970300	0.72262700
C	-2.43803000	-2.24407800	0.92417800
C	-1.85922200	-4.89008500	0.59207500
H	0.19062500	-4.18732100	0.69783300
C	-3.49608200	-3.13825800	0.79575800
C	-3.19376200	-4.49158800	0.62841100
H	-1.58690900	-5.93254700	0.46934900
H	-4.51942300	-2.78266300	0.82990900
H	-3.99099300	-5.22117700	0.53125000
C	-2.13989000	1.28104800	1.41075000
C	-2.60219600	-0.81040500	1.11045800
C	-3.53384900	1.13448700	1.35370500
C	-1.56481100	2.53639400	1.61286700
C	-4.42493500	2.18700500	1.47465600
C	-2.44268000	3.61122400	1.73811200
H	-0.48936300	2.65621800	1.67396900
C	-3.84028500	3.44156300	1.66776800
H	-5.49869900	2.04708100	1.43258500
H	-2.04380800	4.60673700	1.90271600
H	-4.48370500	4.30802800	1.77770100
N	-1.13779900	-2.62450800	0.88677900
N	-1.58644200	0.01125800	1.24266900
O	-3.80623100	-0.21442100	1.16388300

^{3}INT1_D

Zero-point correction= 0.580964
(Hartree/Particle)
Thermal correction to Energy= 0.620295
Thermal correction to Enthalpy= 0.621239
Thermal correction to Gibbs Free Energy= 0.505977

C	-2.57821900	-0.38470200	-2.27270400
C	-1.30048900	-0.95230200	-2.14057800
C	-1.12699000	-2.33180300	-2.28407600
C	-2.26741800	-3.08714700	-2.54463300
C	-3.54100000	-2.49240700	-2.66132400
C	-3.72798400	-1.11438100	-2.52925600
C	-1.12153800	1.16717600	-1.84985500
H	-0.14637200	-2.78721500	-2.20328800
H	-2.17712100	-4.16067700	-2.67205600

H	-4.39923100	-3.12050600	-2.87618800
H	-4.70052700	-0.64778400	-2.63398600
N	-0.39789800	0.07884000	-1.86552900
O	-2.44554000	0.98079400	-2.08914900
C	-0.62222600	2.50219000	-1.55252200
C	-1.36046700	3.65837400	-1.79500300
C	1.16578000	3.72397300	-0.71841300
C	-0.78888700	4.89059900	-1.48119400
H	-2.35321800	3.58385100	-2.22275900
C	0.49548800	4.92615900	-0.93809100
H	2.16305800	3.70275300	-0.29231500
H	-1.33603900	5.80948400	-1.66447100
H	0.97675000	5.86503100	-0.68821600
N	0.62112300	2.53405300	-1.01154100
Cu	1.48757300	0.77775800	-0.61224400
O	5.39765600	0.01377400	-2.07789600
C	4.21536700	-0.12350200	-1.50381800
C	4.09464500	-1.37751500	-0.64240700
C	2.61301600	-1.79152200	-0.55032500
H	2.09636100	-1.81675000	-1.51390600
H	2.56446600	-2.79822200	-0.11100200
O	3.33598700	0.73347900	-1.66191000
N	2.00073500	-0.80397600	0.33956600
C	4.37088800	-0.99135900	0.84443900
H	5.19239500	-0.27983200	0.95712600
H	4.66100300	-1.90217600	1.37665900
C	3.03401100	-0.43328600	1.41313600
C	2.55215400	-1.11488500	2.66671800
C	3.04266600	1.06990000	1.55714300
C	1.90356900	-0.43384800	3.66336600
H	2.66789700	-2.19452800	2.71527700
C	2.40762600	1.70877600	2.59120900
H	3.61746000	1.63653300	0.82951100
C	1.78133900	0.98006400	3.63073800
H	1.51249800	-0.97908200	4.51754200
H	2.42674800	2.79424000	2.64315900
H	1.28104300	1.50060700	4.43965700
H	5.41056000	0.84415300	-2.59650800
C	5.01065300	-2.50000600	-1.14286000
H	6.06138300	-2.20792600	-1.09317100
H	4.87139600	-3.38774500	-0.51873000
H	4.78427300	-2.76840800	-2.17936400
Cu	0.20581200	-0.79618400	0.94729600
C	-0.70859600	-3.87758200	0.88017200
C	-2.33054000	-2.23713500	0.99798200
C	-1.67960500	-4.87786400	0.77121100
H	0.34974600	-4.11915300	0.88131600
C	-3.36299700	-3.16239300	0.88440000
C	-3.02386800	-4.51286200	0.77107900
H	-1.37892000	-5.91675100	0.69225300
H	-4.39547700	-2.83266900	0.89147400
H	-3.80082700	-5.26575700	0.68859800
C	-2.12489500	1.30342200	1.41776000
C	-2.53434400	-0.80347700	1.14399000
C	-3.51311300	1.12591400	1.32398800
C	-1.58004600	2.57207300	1.61892300
C	-4.42797300	2.16201600	1.40043900
C	-2.48236600	3.63053100	1.70045500
H	-0.50947200	2.71323800	1.71511800
C	-3.87352500	3.43088400	1.58956200
H	-5.49710500	1.99983800	1.33014100
H	-2.10861700	4.63588300	1.86360700
H	-4.53706100	4.28565700	1.66701600
N	-1.02002500	-2.58488500	0.99174300
N	-1.54104500	0.04326700	1.28771500
O	-3.75137000	-0.23245500	1.15263100

^{3}TS2_D

Zero-point correction= 0.580755
(Hartree/Particle)
Thermal correction to Energy= 0.619628
Thermal correction to Enthalpy= 0.620572
Thermal correction to Gibbs Free Energy= 0.506575

C	2.68161200	-0.26494900	2.19493000
C	1.49376700	-0.99103800	2.00889500
C	1.50691100	-2.38750300	2.06874200
C	2.73717200	-2.99713600	2.30109200
C	3.91760400	-2.24417400	2.47223000
C	3.91763400	-0.84797400	2.42556300
C	1.02873100	1.09583500	1.83478900
H	0.59624200	-2.96392400	1.94803800
H	2.79198500	-4.07879200	2.36424100
H	4.85146900	-2.76284300	2.66247600
H	4.81746000	-0.26224800	2.57309300
N	0.46124100	-0.07920600	1.77562400
O	2.36560200	1.07726400	2.08839200
C	0.36487900	2.37187700	1.60460300
C	0.93975700	3.58982400	1.96335400

C	-1.52873200	3.42561400	0.77308100
C	0.23047700	4.76279100	1.71134800
H	1.91640400	3.60576700	2.43228800
C	-1.02538800	4.68061300	1.10995100
H	-2.49408800	3.31119300	0.29285200
H	0.64982000	5.72501000	1.98639300
H	-1.61103600	5.56937000	0.90329800
N	-0.84988300	2.29133400	1.00643000
Cu	-1.52303800	0.49124300	0.46570700
O	-5.04701200	-0.84105000	2.29942500
C	-3.96718000	-0.77519100	1.53640300
C	-3.89376400	-1.88810900	0.49878600
C	-2.42550300	-2.15413900	0.06034200
H	-1.83687500	-2.44101300	0.94395200
H	-2.44922900	-3.02911900	-0.60643700
O	-3.16718700	0.15659500	1.67426800
N	-1.76579600	-1.04642500	-0.59836900
C	-4.74062600	-1.40318800	-0.73435900
H	-5.73355200	-1.09896100	-0.39009200
H	-4.87484100	-2.27886900	-1.38054400
C	-4.05253900	-0.30089500	-1.48220800
C	-2.84676500	-0.64953500	-2.19095300
C	-4.40190500	1.03575900	-1.36041100
C	-2.14913800	0.37459100	-2.91365500
H	-2.75969400	-1.66818300	-2.56300000
C	-3.64385200	2.03787000	-1.99030200
H	-5.28647700	1.30952600	-0.79162700
C	-2.52121700	1.69792100	-2.77178100
H	-1.33100400	0.09593100	-3.57187100
H	-3.95853300	3.07454400	-1.91822800
H	-1.98406600	2.47485500	-3.30769300
H	-5.05005900	-0.08056500	2.91569100
C	-4.49373300	-3.19054200	1.05688100
H	-5.54557000	-3.06243900	1.31822700
H	-4.42254000	-3.97800700	0.30065500
H	-3.96003900	-3.52372100	1.95272000
Cu	0.03133200	-0.78085500	-1.03523300
C	1.17223000	-3.73956500	-1.20645000
C	2.66622800	-1.97446900	-1.18841900
C	2.21716300	-4.66701800	-1.15617800
H	0.13542100	-4.05893900	-1.23712200
C	3.76539100	-2.82422700	-1.12919300
C	3.52944600	-4.20116100	-1.11550400
H	1.99701600	-5.72878800	-1.15392600
H	4.76948600	-2.41725000	-1.09894100
H	4.36128100	-4.89676900	-1.07700000
C	2.17918300	1.56004200	-1.33047500
C	2.75595000	-0.52280300	-1.22158200
C	3.57872700	1.48423400	-1.27018100
C	1.53763500	2.79521500	-1.43076500
C	4.41198000	2.58948100	-1.28577300
C	2.35654200	3.92222500	-1.44946200
H	0.45775100	2.86361800	-1.49872400
C	3.76074300	3.82265200	-1.37454500
H	5.49144200	2.50421500	-1.24385800
H	1.90522500	4.90529400	-1.53312000
H	4.35636600	4.72898200	-1.39917400
N	1.38655900	-2.42217900	-1.22432200
N	1.69640900	0.25131200	-1.28568400
O	3.92402700	0.13962500	-1.20394200

^{3}INT2_D

Zero-point correction= 0.582250 (Hartree/Particle)

Thermal correction to Energy= 0.621135

Thermal correction to Enthalpy= 0.622079

Thermal correction to Gibbs Free Energy= 0.508079

C	2.62933100	-0.34364900	2.22216600
C	1.44074100	-1.04356100	1.95773500
C	1.42884900	-2.44124100	1.98151600
C	2.63648200	-3.07798100	2.25582700
C	3.81928400	-2.35043400	2.50315800
C	3.84336400	-0.95370700	2.49549000
C	1.01862200	1.05629600	1.82089200
H	0.51695200	-3.00011100	1.80258600
H	2.67054200	-4.16171900	2.29253700
H	4.73469300	-2.88990400	2.72281400
H	4.74406300	-0.38759300	2.70229600
N	0.43639200	-0.10744400	1.69756400
O	2.33981500	1.00613900	2.13963400
C	0.39101700	2.35222000	1.59674100
C	0.98288000	3.55078900	1.99112400
C	-1.44860400	3.46444700	0.71464200
C	0.31081400	4.74399000	1.73063300
H	1.94402300	3.53743600	2.49109600
C	-0.92418100	4.70199400	1.08342500
H	-2.39927400	3.37962500	0.19850500
H	0.74412800	5.69185200	2.03257800

H	-1.47814900	5.60828000	0.86596400
N	-0.80591600	2.31194700	0.96107000
Cu	-1.52128400	0.54709200	0.39157400
O	-4.79560000	-0.94475200	2.51911800
C	-3.82471600	-0.80036400	1.63252300
C	-3.82235900	-1.87035500	0.55111100
C	-2.39339200	-2.09124700	-0.02026400
H	-1.70276900	-2.34297800	0.79021700
H	-2.45339600	-2.96011100	-0.69194600
O	-3.06359000	0.17229000	1.69981300
N	-1.85560400	-0.94607500	-0.76218200
C	-4.75106700	-1.32724100	-0.59246800
H	-5.71952500	-1.03164300	-0.17743200
H	-4.93364200	-2.17670200	-1.26586900
C	-4.09744300	-0.20895300	-1.34013000
C	-2.75041600	-0.56371300	-1.91884300
C	-4.57275600	1.07302100	-1.39938600
C	-2.11630900	0.53742100	-2.71371200
H	-2.84297200	-1.47296500	-2.54030700
C	-3.86799300	2.09599200	-2.08548700
H	-5.52483000	1.31044400	-0.93075700
C	-2.65330200	1.79643700	-2.75259800
H	-1.23192300	0.29286700	-3.29828400
H	-4.29114700	3.09285900	-2.14830000
H	-2.16571000	2.57113600	-3.33789100
H	-4.76704500	-0.19643100	3.14960700
C	-4.36428100	-3.20366500	1.09030000
H	-5.38900500	-3.09765900	1.45092100
H	-4.35962500	-3.94967600	0.29004500
H	-3.75201600	-3.57942700	1.91649800
Cu	0.00410600	-0.69899200	-1.10959600
C	1.00479100	-3.70211700	-1.32776600
C	2.58126000	-2.01250000	-1.23213200
C	2.00262700	-4.68013500	-1.28033600
H	-0.04504800	-3.97020400	-1.39012600
C	3.63678400	-2.91585100	-1.17479500
C	3.33469800	-4.27963300	-1.20150900
H	1.73173300	-5.72965000	-1.31095600
H	4.65840800	-2.55888300	-1.11452300
H	4.13114700	-5.01561100	-1.16523400
C	2.26719900	1.54387100	-1.29007200
C	2.74144200	-0.56673600	-1.22362400
C	3.66044000	1.39853100	-1.21101100
C	1.68786700	2.81067100	-1.37312900
C	4.54651900	2.46175900	-1.18933600
C	2.56081700	3.89628700	-1.35461100
H	0.61406600	2.93315600	-1.45820800
C	3.95719100	3.72676600	-1.26029100
H	5.61978900	2.32294400	-1.13384200
H	2.15912200	4.90169500	-1.42438800
H	4.59628200	4.60331700	-1.25597400
N	1.28241400	-2.39651900	-1.30540300
N	1.72159500	0.25923600	-1.28278500
O	3.93936400	0.03736200	-1.17250700

^{3}TS3_D

Zero-point correction= 0.579487 (Hartree/Particle)

Thermal correction to Energy= 0.618261

Thermal correction to Enthalpy= 0.619205

Thermal correction to Gibbs Free Energy= 0.506663

C	-1.83950800	1.34937600	2.62114100
C	-0.60104800	1.31672800	1.95997800
C	0.20076500	2.45833100	1.90071100
C	-0.29328200	3.60150700	2.52400600
C	-1.54238700	3.61144200	3.17822900
C	-2.35365200	2.47531500	3.24198000
C	-1.50464000	-0.63729500	1.81896100
H	1.15424000	2.44748400	1.38731900
H	0.29827400	4.51088600	2.51205600
H	-1.88091700	4.52441900	3.65673100
H	-3.30966500	2.47204200	3.75233100
N	-0.43341700	0.02466500	1.46139400
O	-2.40257300	0.08366000	2.51952300
C	-1.71428100	-2.03411500	1.47490800
C	-2.77349000	-2.80314700	1.94322200
C	-0.80662800	-3.81685400	0.31109800
C	-2.82462900	-4.14876500	1.57226500
H	-3.52386200	-2.36018800	2.58757100
C	-1.82453000	-4.66567700	0.75226000
H	-0.01558300	-4.17915000	-0.33600900
H	-3.63087100	-4.78227200	1.92700000
H	-1.82302200	-5.70791400	0.45330900
N	-0.74860000	-2.52771300	0.65808600
Cu	0.67612600	-1.10969900	0.10859800
O	6.45477800	-1.57712300	1.08275100
C	5.13128900	-1.43252300	1.22689600
C	4.67433300	-0.00981200	0.88574800

C	3.17914300	0.15767100	1.23718500
H	2.86752800	-0.53231700	2.02416100
H	2.99050100	1.18025700	1.58532700
O	4.39553800	-2.34093600	1.56509900
N	2.37466200	-0.03245800	0.01197700
C	4.70568900	0.20827300	-0.65521700
H	5.48393300	-0.37824500	-1.14963500
H	4.91169600	1.26391700	-0.86192300
C	3.31066500	-0.12723200	-1.17899100
C	2.87957200	0.54810200	-2.42287800
C	2.80699200	-1.49351600	-0.96086900
C	1.82005200	0.01299300	-3.19675000
H	3.40001400	1.44373300	-2.74423500
C	1.60534700	-1.92821500	-1.62580200
H	3.36677300	-2.18470800	-0.34530500
C	1.14249600	-1.16300400	-2.80083800
H	1.61087700	0.44030600	-4.17540200
H	1.38691600	-2.99131900	-1.61074400
H	0.48202800	-1.65890000	-3.50616500
H	6.68544200	-2.49867800	1.31397200
C	5.56064200	1.00772200	1.62602800
H	6.60461700	0.91111600	1.32177400
H	5.22341100	2.02500700	1.40189100
H	5.50919400	0.85991100	2.70956700
Cu	0.07810400	0.51081500	-2.11291700
C	0.49311400	3.53004900	-1.56306400
C	-1.54264600	2.59590400	-0.98045800
C	0.07622000	4.78953400	-1.12387600
H	1.48168800	3.37284500	-1.98099000
C	-2.03627000	3.81019900	-0.51605400
C	-1.20543000	4.92967300	-0.59555700
H	0.74681800	5.63768400	-1.20574600
H	-3.03861100	3.87160800	-0.10885700
H	-1.55827300	5.89623700	-0.25146600
C	-2.87615900	-0.69728900	-1.32467100
C	-2.30225700	1.35554000	-0.97948300
C	-3.92428600	-0.04888300	-0.65362700
C	-2.99541400	-2.03770300	-1.69613100
C	-5.11204600	-0.66494100	-0.29698400
C	-4.18377900	-2.67781800	-1.35293500
H	-2.20344900	-2.54551700	-2.23462800
C	-5.21706500	-2.00876200	-0.66455300
H	-5.90925900	-0.13783500	0.21388900
H	-4.32696100	-3.71654200	-1.63121700
H	-6.12885800	-2.54727200	-0.42850100
N	-0.29703000	2.45391900	-1.49945800
N	-1.85364400	0.23704600	-1.49763800
O	-3.53504900	1.26765100	-0.44319800

^{3}TS4_D

Zero-point correction=	0.574768
(Hartree/Particle)	
Thermal correction to Energy=	0.614117
Thermal correction to Enthalpy=	0.615061
Thermal correction to Gibbs Free Energy=	0.500260

C	3.17304900	-1.07031300	1.79600100
C	1.86259500	-1.56445300	1.70264400
C	1.62404200	-2.93949700	1.68739100
C	2.74036600	-3.76994900	1.75167900
C	4.04880500	-3.25044000	1.82811900
C	4.29814500	-1.87556700	1.85473100
C	1.77780900	0.59329800	1.73013600
H	0.61572200	-3.33462600	1.63415100
H	2.60326100	-4.84618000	1.75139800
H	4.88721200	-3.93676800	1.88452200
H	5.29956400	-1.46856600	1.93200300
N	1.00405600	-0.46588800	1.65197500
O	3.09913200	0.31427800	1.81392800
C	1.27867200	1.95559200	1.70003800
C	2.08141200	3.07669200	1.90277800
C	-0.62115800	3.24890700	1.41928000
C	1.47857800	4.33274500	1.86885000
H	3.14338600	2.95859400	2.08361900
C	0.10771100	4.42160700	1.62127800
H	-1.68588800	3.27301400	1.21443300
H	2.06965600	5.22725500	2.03448500
H	-0.39625100	5.38138000	1.59243200
N	-0.05739700	2.03478300	1.46226500
Cu	-0.90769800	0.13107900	1.25124000
O	-4.68958300	-1.08469600	2.49044300
C	-3.76611400	-1.04811700	1.54131300
C	-4.26163600	-1.57200800	0.20509500
C	-3.10133300	-1.94200400	-0.74772500
H	-2.35883100	-2.54615700	-0.22278600
H	-3.52749200	-2.57419300	-1.53581200
O	-2.65052900	-0.58391800	1.77732200
N	-2.35869900	-0.84325700	-1.40405600
C	-5.10980600	-0.43297800	-0.42957100

H	-5.90975000	-0.13134700	0.25146200
H	-5.59250000	-0.85459600	-1.32294600
C	-4.25108000	0.73546000	-0.81543100
C	-3.03217100	0.45129800	-1.51684800
C	-4.58086100	2.05177600	-0.57534400
C	-2.17446200	1.53446300	-1.93191400
H	-3.03397700	-0.48827800	-2.49785700
C	-3.74056100	3.11674900	-0.98708600
H	-5.51429100	2.27884200	-0.06739600
C	-2.55331200	2.84848100	-1.66631900
H	-1.34950300	1.33261800	-2.61422000
H	-4.04896300	4.14080700	-0.80634800
H	-1.94059300	3.66675800	-2.03192600
H	-4.32324700	-0.70265500	3.31347600
C	-5.13199800	-2.82750700	0.41881500
H	-5.97748000	-2.61271800	1.07408700
H	-5.52159800	-3.16968200	-0.54431800
H	-4.55292800	-3.64272200	0.86520100
Cu	-0.49724600	-0.39456300	-1.22678100
C	0.25909200	-3.46040400	-1.56516000
C	1.96844000	-1.89553400	-1.54337400
C	1.17451200	-4.50764600	-1.66592900
H	-0.80920800	-3.64823700	-1.54632100
C	2.94950100	-2.88262500	-1.62854000
C	2.53988300	-4.21228400	-1.69280900
H	0.81909800	-5.53005700	-1.72823300
H	3.99705100	-2.60576500	-1.64799700
H	3.27440600	-5.00690300	-1.76935000
C	1.90009100	1.66829400	-1.49155600
C	2.23425500	-0.47678700	-1.51362500
C	3.28160800	1.42548000	-1.44857100
C	1.41031600	2.97405200	-1.50653600
C	4.23787100	2.42520300	-1.40461000
C	2.35513400	3.99702200	-1.47079100
H	0.34685700	3.17608200	-1.52825100
C	3.73756800	3.73020000	-1.41738900
H	5.29958600	2.21018500	-1.37556300
H	2.02064200	5.02892500	-1.48526600
H	4.43607200	4.55998400	-1.39616900
N	0.63726200	-2.17924700	-1.50203500
N	1.26787100	0.42289900	-1.52752700
O	3.47246800	0.05005600	-1.46147900

Zero-point correction= 0.583909 (Hartree/Particle)
Thermal correction to Energy= 0.622607
Thermal correction to Enthalpy= 0.623551
Thermal correction to Gibbs Free Energy= 0.511528

C	3.16074100	-1.54233800	1.51424100
C	1.79711600	-1.30305700	1.73668400
C	0.97244500	-2.32223400	2.21295400
C	1.56318600	-3.56601100	2.42022600
C	2.93071200	-3.78826400	2.16528400
C	3.77019600	-2.77013000	1.70735500
C	2.73847500	0.54556700	1.08082800
H	-0.07839000	-2.15136200	2.41666800
H	0.95701100	-4.38626000	2.78817000
H	3.34838100	-4.77316600	2.34441100
H	4.82783000	-2.92432400	1.52874000
N	1.56272800	0.04111000	1.43407600
O	3.73971800	-0.35129100	1.10081300
C	2.93230100	1.93974100	0.74866900
C	4.16241600	2.50661400	0.41871700
C	1.83507100	3.96960700	0.54693200
C	4.20428800	3.87609400	0.15772700
H	5.05312700	1.89072500	0.37651300
C	3.02796200	4.62343900	0.23126600
H	0.89388000	4.50822800	0.59910800
H	5.14547500	4.35347100	-0.09421900
H	3.02756300	5.69161400	0.04575200
N	1.78067100	2.66081300	0.80166300
Cu	0.15875500	1.25700600	0.88902500
O	-3.27344800	3.36374000	0.00580900
C	-2.57980200	2.41254000	0.59296300
C	-3.38371800	1.60798700	1.60099200
C	-2.78299900	0.22607800	1.87729900
H	-1.78177300	0.28604400	2.31066800
H	-3.43317800	-0.25470600	2.61498900
O	-1.37029300	2.29230500	0.33471800
N	-2.70598700	-0.64235700	0.65818800
C	-4.81235900	1.38370500	1.07038600
H	-5.29477400	2.33535800	0.84004000
H	-5.39763200	0.92245200	1.87606000
C	-4.78766100	0.49527800	-0.15061800
C	-3.79169700	-0.47519000	-0.32215300
C	-5.76691000	0.61314700	-1.14432800
C	-3.77779300	-1.31048800	-1.44647700

^{3}PRO_D

H	-2.72962500	-1.60858900	0.98805000
C	-5.76719700	-0.21524800	-2.26384700
H	-6.54280500	1.36401100	-1.02604900
C	-4.76970900	-1.18235900	-2.41410100
H	-2.99668800	-2.05563700	-1.56534400
H	-6.54017400	-0.10671600	-3.01723400
H	-4.76087200	-1.83326000	-3.28190000
H	-2.69695200	3.88012500	-0.59351400
C	-3.38670400	2.42768400	2.91480200
H	-3.86341500	3.39884600	2.76070200
H	-3.95450200	1.88653700	3.67749900
H	-2.37088800	2.58865100	3.29137200
Cu	-0.98776400	-0.63327900	-0.34829400
C	-1.27733500	-3.73166200	-0.03953600
C	0.64556000	-2.82504900	-1.03293500
C	-0.73034900	-5.00074400	-0.03376600
H	-2.28832500	-3.56920800	0.32867400
C	1.26558100	-4.09405600	-1.04505500
C	0.57723500	-5.17880800	-0.54649500
H	-1.30732900	-5.83764200	0.34166400
H	2.26845800	-4.18796300	-1.44588500
H	1.02987100	-6.16460200	-0.55358800
C	1.41384800	0.50945000	-1.98176300
C	1.21347100	-1.62637700	-1.49861200
C	2.62590200	-0.14224300	-2.26259500
C	1.27618800	1.87188400	-2.24429000
C	3.73763800	0.49815800	-2.77628100
C	2.38886200	2.53984100	-2.76115400
H	0.34049700	2.38787200	-2.05661300
C	3.59724300	1.87138500	-3.01579700
H	4.65516500	-0.03638800	-2.99382300
H	2.31541700	3.59964200	-2.98099200
H	4.43472600	2.42278800	-3.42953100
N	-0.62928900	-2.65230000	-0.51901500
N	0.53096200	-0.45172200	-1.48367400
O	2.48831600	-1.48813900	-1.95576900

MECP

C	2.56436850	1.11296160	2.11360110
C	1.64499820	0.06735050	1.92555260
C	2.02777240	-1.25516540	2.16653870
C	3.34341950	-1.46722840	2.57217320
C	4.25000710	-0.39992170	2.73892120
C	3.87700400	0.92735260	2.51575800
C	0.66449100	1.91062050	1.44105990
H	1.32874390	-2.07509890	2.04308390
H	3.68110960	-2.47795590	2.77606010
H	5.26294190	-0.61324080	3.06329030
H	4.56263340	1.75522090	2.65259870
N	0.44161970	0.62469780	1.48830780
O	1.91452290	2.29366100	1.81179050
C	-0.29109350	2.92057710	1.00954860
C	-0.06984080	4.28587470	1.18638160
C	-2.35207250	3.31018160	0.01428820
C	-1.04742470	5.18232070	0.75772330
H	0.84622490	4.62334320	1.65590810
C	-2.21059010	4.68760710	0.16729000
H	-3.23262980	2.87918350	-0.45082640
H	-0.90478780	6.25000470	0.88838480
H	-2.99611540	5.35250940	-0.17376210
N	-1.41255740	2.44074040	0.41709870
Cu	-1.59197280	0.48924220	0.17846530
O	-4.58077320	-1.27178660	2.70832510
C	-3.55762350	-1.12356130	1.87299450
C	-3.35575360	-2.33825730	0.96971000
C	-1.95410130	-2.33792820	0.32351530
H	-1.18763670	-2.30971720	1.10365670
H	-1.85736830	-3.29687460	-0.21108290
O	-2.90614580	-0.08216930	1.84297730
N	-1.68053850	-1.24809300	-0.62166720
C	-4.40027230	-2.23352040	-0.18743780
H	-5.41281330	-2.14676630	0.21542540
H	-4.34226120	-3.18133600	-0.74257690
C	-4.06584130	-1.09781080	-1.09442170
C	-2.69423350	-1.15021990	-1.72109000
C	-4.93025370	-0.05627600	-1.33972380
C	-2.36029500	0.08697100	-2.50382340
H	-2.62885710	-2.04866250	-2.36921160
C	-4.57659530	0.99822490	-2.20478410
H	-5.91256260	-0.05634450	-0.87614470
C	-3.28050780	1.06867260	-2.76468370
H	-1.36676900	0.13289750	-2.94277660
H	-5.29938630	1.77968130	-2.41816190
H	-3.01225740	1.91420740	-3.39040590
H	-4.67412420	-0.45598440	3.24094380
C	-3.56722280	-3.64089260	1.75847420
H	-4.58161630	-3.70059830	2.15693250
H	-3.40493610	-4.49926510	1.09947240

H	-2.86750910	-3.71264490	2.59753810
Cu	0.18197220	-0.99390430	-1.04498130
C	1.41595090	-4.02356510	-0.74231340
C	2.86395670	-2.23631620	-0.92449520
C	2.48036740	-4.91736900	-0.59111880
H	0.38742660	-4.37081750	-0.73338560
C	3.98450750	-3.04923300	-0.77750950
C	3.78209490	-4.42019870	-0.60677780
H	2.28403980	-5.97683940	-0.46560120
H	4.97912650	-2.61798650	-0.79938660
H	4.62993910	-5.08786630	-0.49254470
C	2.34612980	1.26404590	-1.40894660
C	2.93132100	-0.79393390	-1.10803790
C	3.73081950	1.22145400	-1.19429060
C	1.69714380	2.47989530	-1.62803570
C	4.54066280	2.34444480	-1.16977760
C	2.49094460	3.62478220	-1.60341910
H	0.62921190	2.52090470	-1.81265840
C	3.88076690	3.55855200	-1.37724880
H	5.61033100	2.28483770	-1.00738930
H	2.03279670	4.59377520	-1.77196140
H	4.45914960	4.47620650	-1.37513740
N	1.59553820	-2.71000720	-0.90174190
N	1.87740490	-0.04787170	-1.34335260
O	4.08507400	-0.10759260	-1.01205330

^{1}INT2_D

Zero-point correction= 0.582453
(Hartree/Particle)
Thermal correction to Energy= 0.621788
Thermal correction to Enthalpy= 0.622732
Thermal correction to Gibbs Free Energy= 0.507126

C	2.53353800	1.12740000	2.08841000
C	1.61366200	0.08622000	1.87853800
C	1.98762100	-1.23948200	2.11595200
C	3.29600900	-1.45983800	2.53952800
C	4.20340500	-0.39695900	2.72901400
C	3.83886700	0.93343600	2.51060300
C	0.64673400	1.93601200	1.39416400
H	1.28850500	-2.05564000	1.97242600
H	3.62758900	-2.47383400	2.73729900
H	5.21094100	-0.61688500	3.06601700
H	4.52614400	1.75692100	2.66472200
N	0.41932400	0.65110700	1.42811700
O	1.89228800	2.31305700	1.78478700
C	-0.31097300	2.94706100	0.97202300
C	-0.09545500	4.31144400	1.16222200
C	-2.38645300	3.33888700	0.00813000
C	-1.08312900	5.20807800	0.75892000
H	0.82395400	4.64762100	1.62664200
C	-2.25267200	4.71445500	0.17988500
H	-3.27052900	2.91062600	-0.45213500
H	-0.94519000	6.27477100	0.90219600
H	-3.04864500	5.37853300	-0.13762100
N	-1.43723300	2.46886700	0.38419700
Cu	-1.58913900	0.52810400	0.11939300
O	-4.65414500	-1.19513400	2.59756200
C	-3.54282100	-1.10576200	1.86724900
C	-3.34601600	-2.32015000	0.96010900
C	-1.95018700	-2.31334000	0.31119000
H	-1.18232900	-2.27541500	1.08851100
H	-1.84859700	-3.27358100	-0.22110500
O	-2.80798700	-0.12701600	1.92852500
N	-1.67484100	-1.22639200	-0.63931800
C	-4.38916800	-2.23825100	-0.19874100
H	-5.40685400	-2.18508600	0.19569900
H	-4.29427500	-3.17367900	-0.76994700
C	-4.07787200	-1.07869900	-1.08059700
C	-2.70413200	-1.07718300	-1.71096600
C	-4.97512600	-0.06853800	-1.32242100
C	-2.36842700	0.24188400	-2.34896400
H	-2.65756200	-1.90206800	-2.45275900
C	-4.63664100	1.02188000	-2.15528500
H	-5.96839500	-0.11451500	-0.88527900
C	-3.32980500	1.18967300	-2.63620800
H	-1.35733700	0.34730300	-2.73284800
H	-5.39172800	1.76999600	-2.37970900
H	-3.06926000	2.07889500	-3.20120900
H	-4.72497900	-0.39467700	3.15537500
C	-3.54999000	-3.61574600	1.76512300
H	-4.55626400	-3.66454500	2.18518500
H	-3.40325200	-4.48311900	1.11407200
H	-2.83332700	-3.67982900	2.59047400
Cu	0.20719600	-1.07704500	-1.09407700
C	1.52094000	-4.12006400	-0.72061400
C	2.92627400	-2.30588600	-0.93060200
C	2.60522500	-4.98910200	-0.56205300
H	0.50049700	-4.49078200	-0.70239500

C	4.06626400	-3.09145000	-0.77913900
C	3.89597600	-4.46429500	-0.59005600
H	2.43287000	-6.05042300	-0.42109400
H	5.05071500	-2.63876700	-0.81282600
H	4.75888600	-5.11135000	-0.47159300
C	2.36099900	1.18429100	-1.44070200
C	2.96843500	-0.86395700	-1.12751900
C	3.74087400	1.16308300	-1.19465500
C	1.69969600	2.39234300	-1.66659700
C	4.53218800	2.29838200	-1.14510200
C	2.47452100	3.54953400	-1.61814100
H	0.63486800	2.41940200	-1.87184100
C	3.85948000	3.50388500	-1.36075700
H	5.59843800	2.25411900	-0.95678200
H	2.00514300	4.51241200	-1.79136800
H	4.42339300	4.43036800	-1.33919100
N	1.66865300	-2.80486100	-0.89680800
N	1.90923300	-0.13492400	-1.38849100
O	4.11058300	-0.15960000	-1.00570000

[1]TS4_D

Zero-point correction= 0.578050
(Hartree/Particle)
Thermal correction to Energy= 0.617532
Thermal correction to Enthalpy= 0.618476
Thermal correction to Gibbs Free Energy= 0.503765

C	2.96496300	-0.48817400	1.95637500
C	1.73878300	-1.16140200	1.84811900
C	1.69483100	-2.55669000	1.86892600
C	2.91282600	-3.22104100	1.98558600
C	4.13409700	-2.52171700	2.07932100
C	4.18956200	-1.12606000	2.06920800
C	1.35650500	0.95918700	1.78579100
H	0.75346500	-3.09034500	1.80204500
H	2.92672400	-4.30536600	2.01205300
H	5.05749700	-3.08290300	2.17691600
H	5.12189800	-0.58068500	2.15649100
N	0.73813000	-0.19563400	1.73145200
O	2.69910100	0.87184200	1.91959000
C	0.68748900	2.24601700	1.67635800
C	1.33520200	3.46298800	1.87695400
C	-1.33239400	3.28127400	1.24541100
C	0.58874500	4.63479000	1.75312600
H	2.39058400	3.48296700	2.12232000
C	-0.76492800	4.54445200	1.43178300
H	-2.38193800	3.16733600	0.99573400
H	1.05711600	5.60088600	1.90982600
H	-1.37954300	5.43254900	1.33359200
N	-0.62729500	2.14981000	1.36016700
Cu	-1.21535700	0.10390500	1.17592100
O	-4.99212200	-1.49143600	2.13822800
C	-3.86908500	-1.30454900	1.45945500
C	-3.86889000	-2.01607900	0.11236600
C	-2.48800800	-1.94193700	-0.56125000
H	-1.73010500	-2.42415200	0.06184200
H	-2.54685400	-2.52432200	-1.49048900
O	-2.96505000	-0.60531800	1.91755600
N	-1.99143200	-0.57508500	-0.81793400
C	-4.88982900	-1.30651200	-0.80984100
H	-5.87269500	-1.26212900	-0.33236800
H	-5.01173500	-1.92967500	-1.70603500
C	-4.44100900	0.07782400	-1.19600500
C	-3.04022300	0.38115700	-1.30404900
C	-5.35696000	1.08703300	-1.47273000
C	-2.63639700	1.72495800	-1.60151800
H	-2.49799600	-0.24971200	-2.16742600
C	-4.94657400	2.37772500	-1.81680300
H	-6.41762200	0.86119800	-1.41031100
C	-3.58001100	2.69177600	-1.88206700
H	-1.57296100	1.93742700	-1.64839100
H	-5.68929400	3.13751500	-2.03622800
H	-3.26354500	3.69449100	-2.15022800
H	-4.93848200	-1.01056800	2.98911600
C	-4.26134800	-3.49387600	0.30736000
H	-5.26935100	-3.58152900	0.71727800
H	-4.23537800	-4.01076000	-0.65709100
H	-3.56998000	-4.00156200	0.98803300
Cu	-0.12898800	-0.33399700	-1.18743000
C	0.90667900	-3.65663800	-1.37198100
C	2.45564100	-1.95871700	-1.41606300
C	1.91082000	-4.62937300	-1.42948700
H	-0.14066900	-3.94248600	-1.33544600
C	3.52569800	-2.85062800	-1.46896500
C	3.24020100	-4.21692000	-1.47690800
H	1.64740700	-5.68138200	-1.44102500
H	4.54407500	-2.48222700	-1.50844200
H	4.04460800	-4.94355900	-1.52428200
C	2.27260700	1.62115300	-1.42876500

C	2.63899400	-0.51409300	-1.41843100
C	3.65765200	1.40708900	-1.43956500
C	1.75715100	2.91745400	-1.43265600
C	4.59635900	2.42445800	-1.45065100
C	2.68242300	3.95936300	-1.44611200
H	0.68831300	3.10161500	-1.42141400
C	4.07082800	3.71919100	-1.45383200
H	5.66235800	2.23020900	-1.46466200
H	2.32778300	4.98469200	-1.45619000
H	4.75292000	4.56255900	-1.47059400
N	1.16215300	-2.34779300	-1.36281200
N	1.66033000	0.36407100	-1.41160300
O	3.86829600	0.03667900	-1.43494100

^{1}PRO_D

Zero-point correction= 0.585157
(Hartree/Particle)

Thermal correction to Energy= 0.624549

Thermal correction to Enthalpy= 0.625493

Thermal correction to Gibbs Free Energy= 0.509955

C	3.26018600	-0.71804900	1.80820700
C	2.02037000	-1.37039900	1.74354000
C	1.95147000	-2.76392100	1.76415300
C	3.16189900	-3.44822000	1.84137200
C	4.39725200	-2.77042900	1.89473400
C	4.47653300	-1.37561400	1.88036600
C	1.67418800	0.76336000	1.71096300
H	0.99927700	-3.28126600	1.72878500
H	3.15804600	-4.53269300	1.86878700
H	5.31348900	-3.34739600	1.96328000
H	5.42031400	-0.84605800	1.93672300
N	1.03593100	-0.38278400	1.67260300
O	3.01532600	0.64836400	1.78583700
C	1.01599700	2.06094300	1.66819800
C	1.68993200	3.26701700	1.83855700
C	-1.02299100	3.11250900	1.41698200
C	0.94287400	4.44542400	1.79678200
H	2.76199200	3.27704500	1.99677700
C	-0.43160700	4.36946900	1.58210000
H	-2.08829200	3.00836500	1.23969000
H	1.42977700	5.40549900	1.93209800
H	-1.04482400	5.26333100	1.54802000
N	-0.31955900	1.97793400	1.45959000
Cu	-0.91106300	-0.14520800	1.47308700
O	-4.83474600	-1.33478000	2.09616200
C	-3.72992100	-1.30756200	1.37114600
C	-3.87323000	-2.05197800	0.04758000
C	-2.56296900	-1.99811600	-0.74892500
H	-1.71319100	-2.17462300	-0.08667600
H	-2.57738100	-2.80503600	-1.48501900
O	-2.72551300	-0.72436100	1.78656500
N	-2.32084800	-0.71342400	-1.51789500
C	-4.97961800	-1.33705500	-0.76801900
H	-5.94592700	-1.42705800	-0.26744800
H	-5.06831000	-1.84805500	-1.73664400
C	-4.58277400	0.10695600	-0.92917700
C	-3.24649200	0.39487800	-1.23543700
C	-5.46395100	1.16767500	-0.70311100
C	-2.80388800	1.71102000	-1.33913000
H	-2.45839100	-0.94463800	-2.50435100
C	-5.03212700	2.48968600	-0.81816200
H	-6.49722900	0.95223000	-0.44708900
C	-3.70155000	2.76167500	-1.14116900
H	-1.76474500	1.90742300	-1.58577800
H	-5.73227300	3.30307400	-0.65820200
H	-3.36229000	3.78781500	-1.24309900
H	-4.69121600	-0.84775000	2.93259600
C	-4.23537000	-3.52371400	0.33885300
H	-5.13890800	-3.58778900	0.94822900
H	-4.41854400	-4.04739400	-0.60404600
H	-3.42559500	-4.03825000	0.86778400
Cu	-0.40831400	-0.24854000	-1.45401700
C	0.89747100	-3.53644300	-1.54485000
C	2.30463600	-1.72022800	-1.51415400
C	1.97704800	-4.42434200	-1.61265200
H	-0.12371200	-3.90724500	-1.53313600
C	3.44381600	-2.52226100	-1.57181200
C	3.26927600	-3.90586800	-1.62457900
H	1.79879000	-5.49314400	-1.65876200
H	4.42989400	-2.07334800	-1.58084800
H	4.13028700	-4.56383000	-1.67840300
C	1.86584500	1.84179000	-1.52183200
C	2.37840200	-0.26522500	-1.49057000
C	3.26117000	1.72431500	-1.46691400
C	1.26195000	3.09824900	-1.57344900
C	4.12620500	2.80509600	-1.43888900
C	2.11183600	4.20167100	-1.55461500
H	0.18483200	3.20784400	-1.62428800

C	3.51228800	4.05965400	-1.48365100
H	5.20260600	2.68598000	-1.40045600
H	1.68788300	5.19922200	-1.60171600
H	4.13408700	4.94862600	-1.47772900
N	1.04539200	-2.21197900	-1.49753300
N	1.34298300	0.54442900	-1.51923200
O	3.56609000	0.37162600	-1.45099900

9. NMR spectra

5-(4-((*tert*-Butyldimethylsilyl)oxy)phenethyl)-2,2-dimethyl-1,3-dioxane-4,6-dione (S1u)

^{1}H NMR (400 MHz, $CDCl_3$)

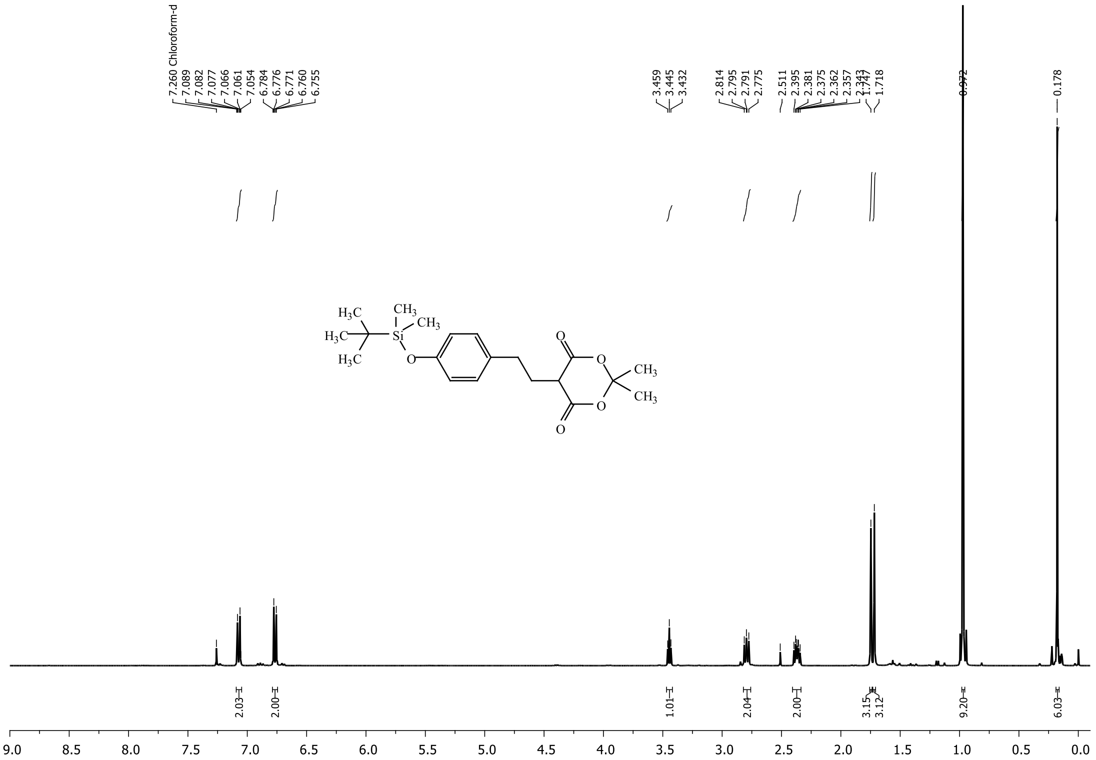

^{13}C NMR (101 MHz, $CDCl_3$)

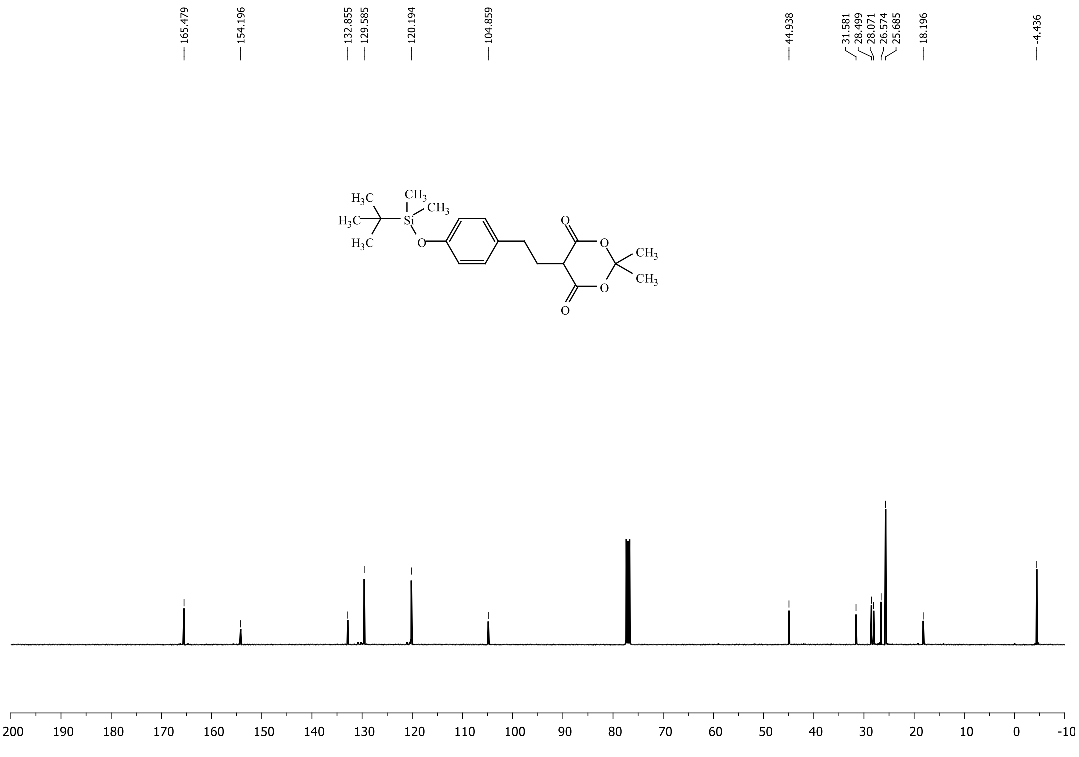

tert-Butyl 4-hexyl-5-oxoisoxazolidine-2-carboxylate (S2b)

^{1}H NMR (400 MHz, $CDCl_3$)

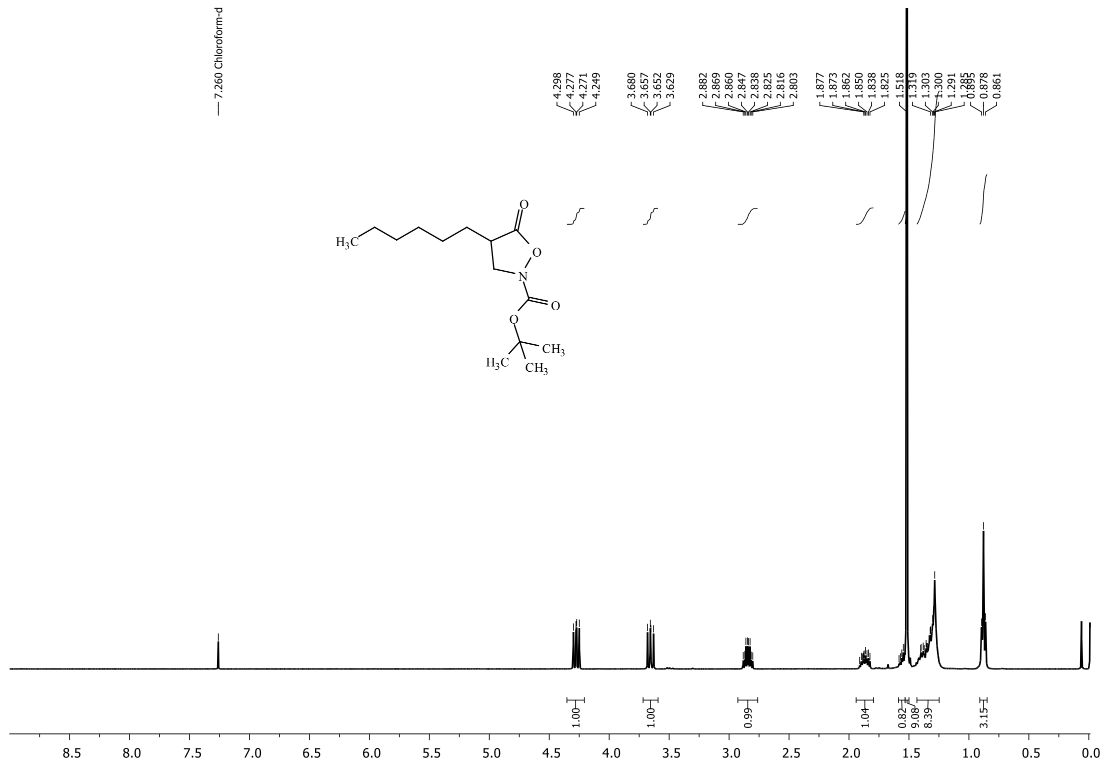

^{13}C NMR (101 MHz, $CDCl_3$)

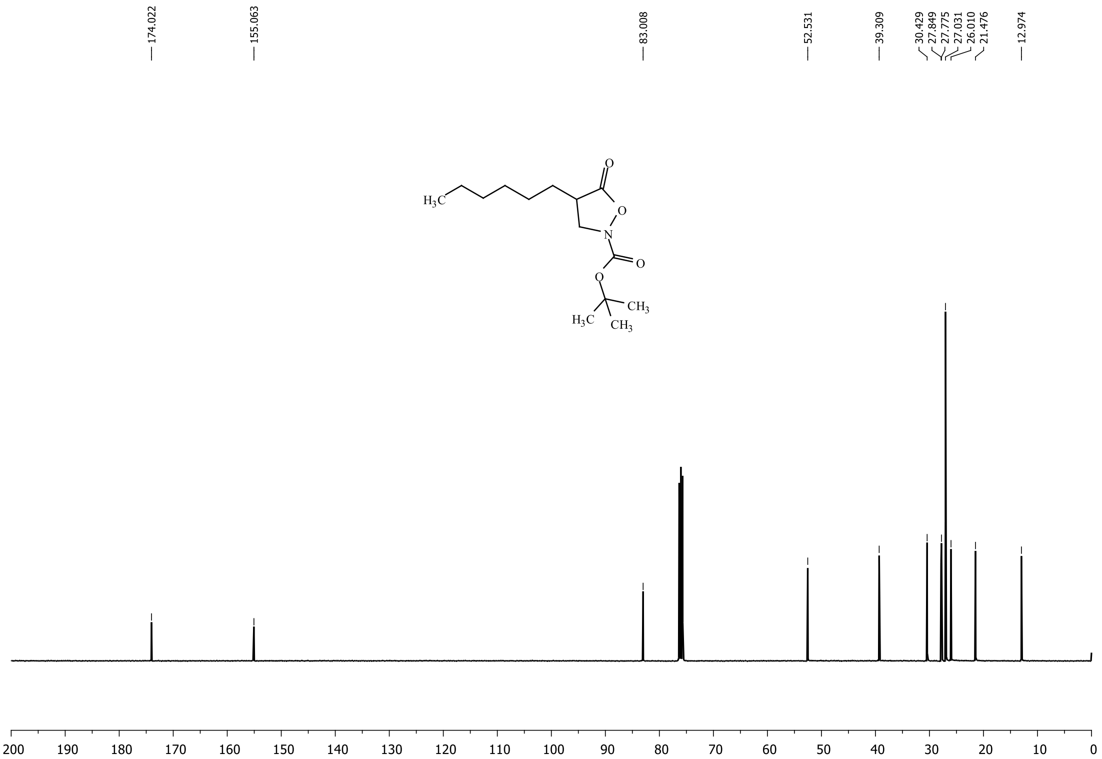

tert-Butyl 5-oxo-4-phenethylisoxazolidine-2-carboxylate (S2c)

^{1}H NMR (400 MHz, $CDCl_3$)

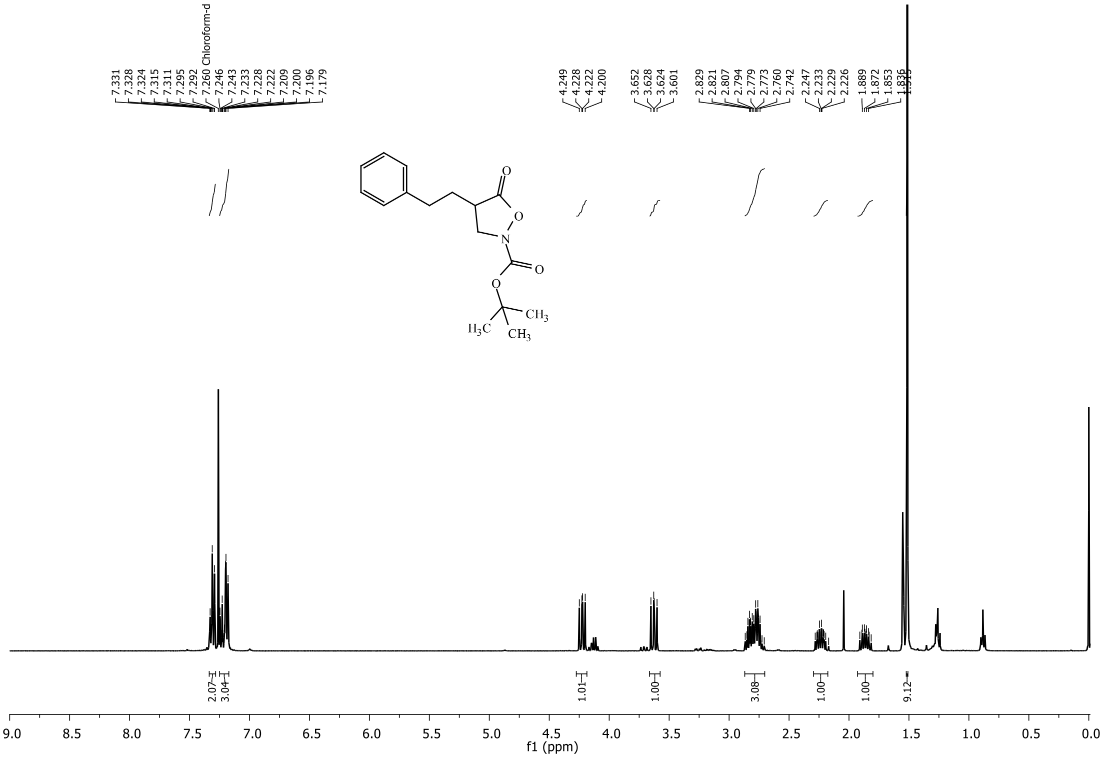

^{13}C NMR (101 MHz, $CDCl_3$)

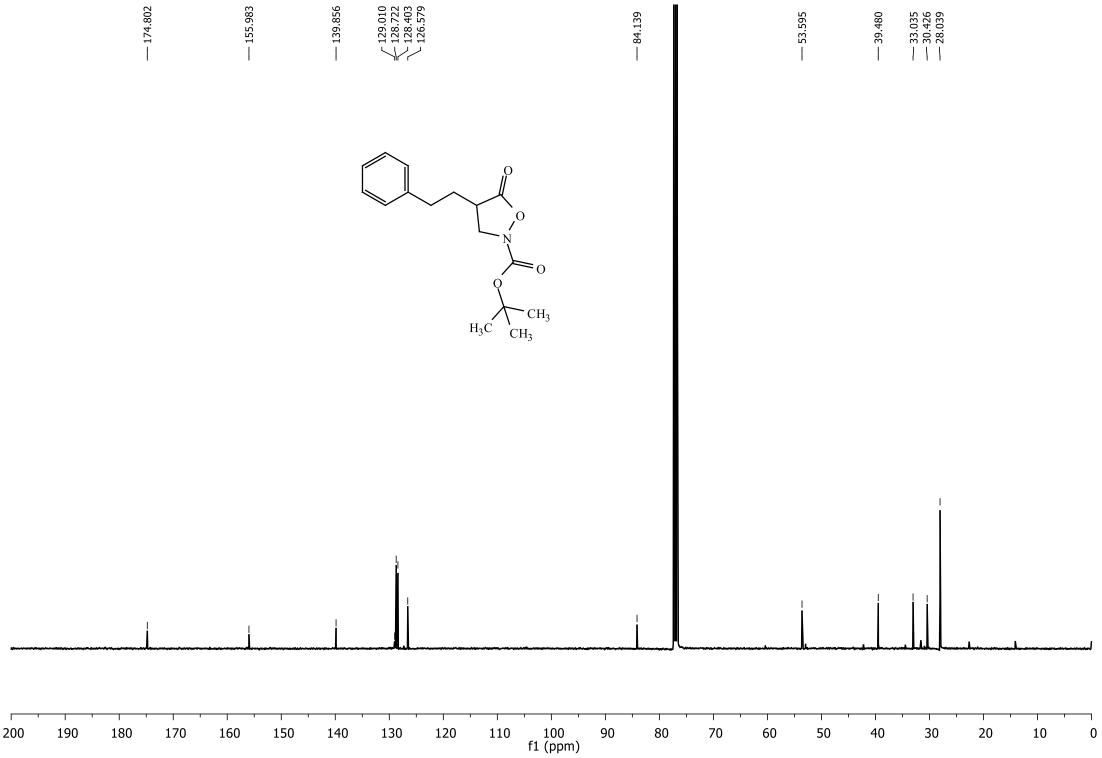

***tert*-Butyl 5-oxo-4-(pent-4-en-1-yl)isoxazolidine-2-carboxylate (S2g)**

^{1}H NMR (400 MHz, $CDCl_3$)

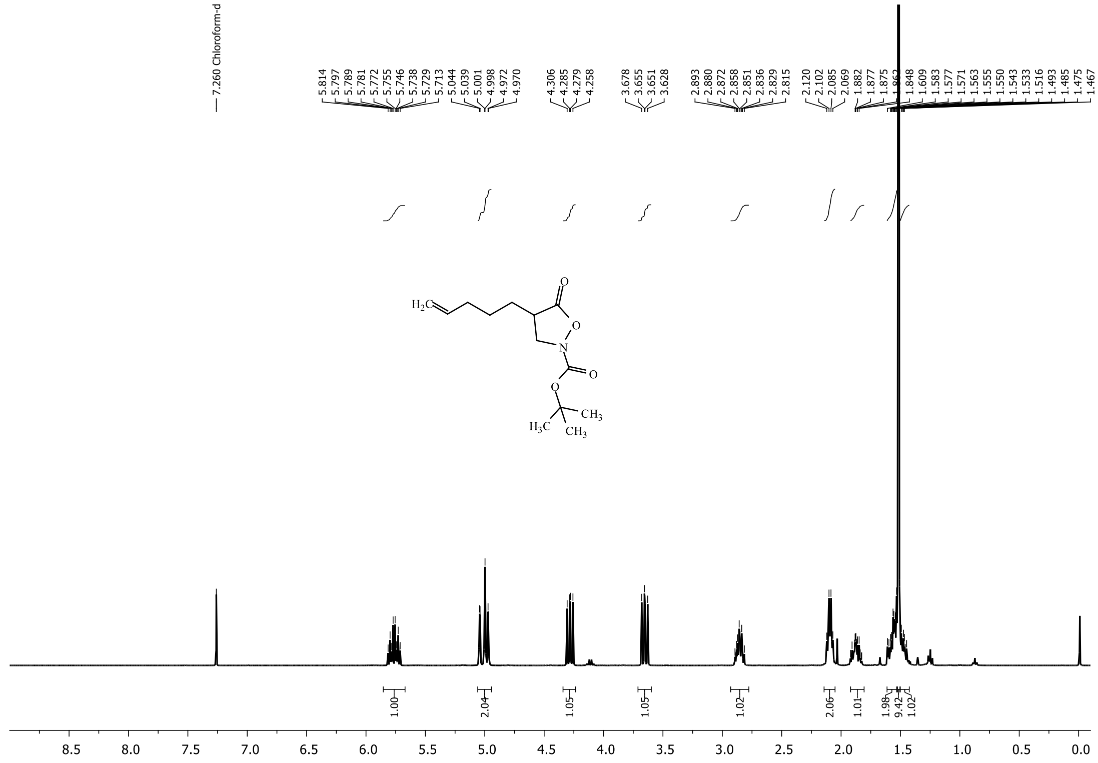

^{13}C NMR (101 MHz, $CDCl_3$)

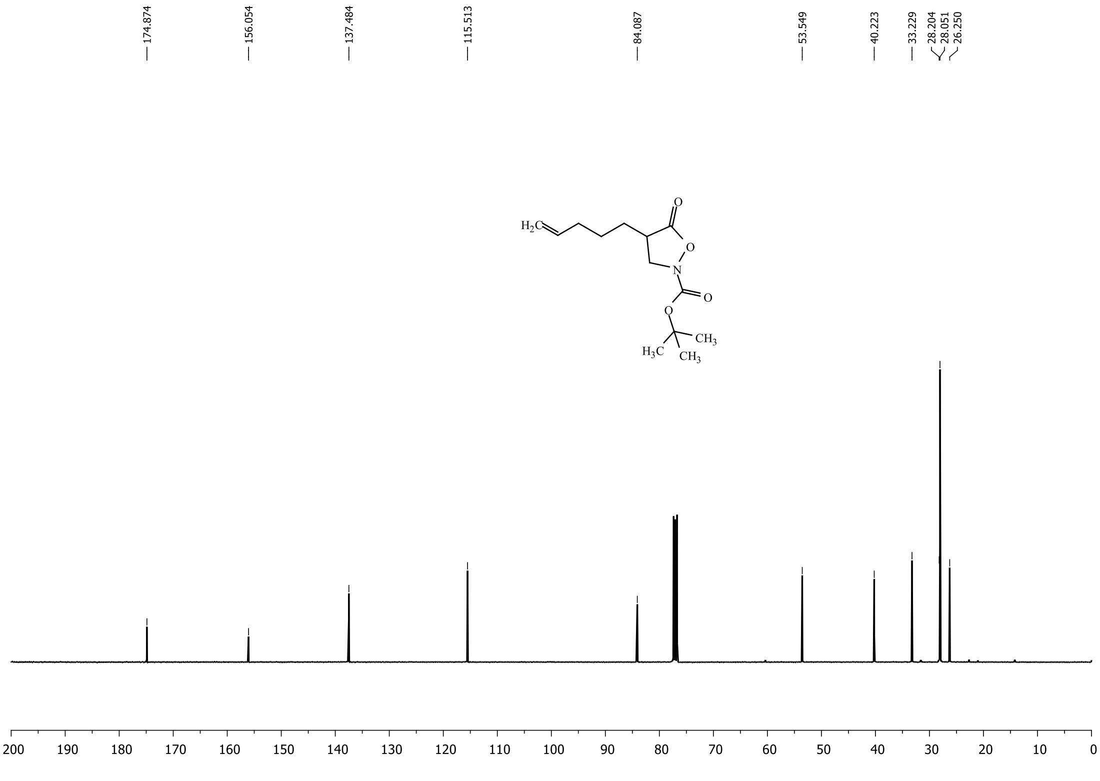

tert-Butyl 4-(4-((_tert_-butyldimethylsilyl)oxy)phenethyl)-5-oxoisoxazolidine-2-carboxylate (S2u)

^{1}H NMR (400 MHz, $CDCl_3$)

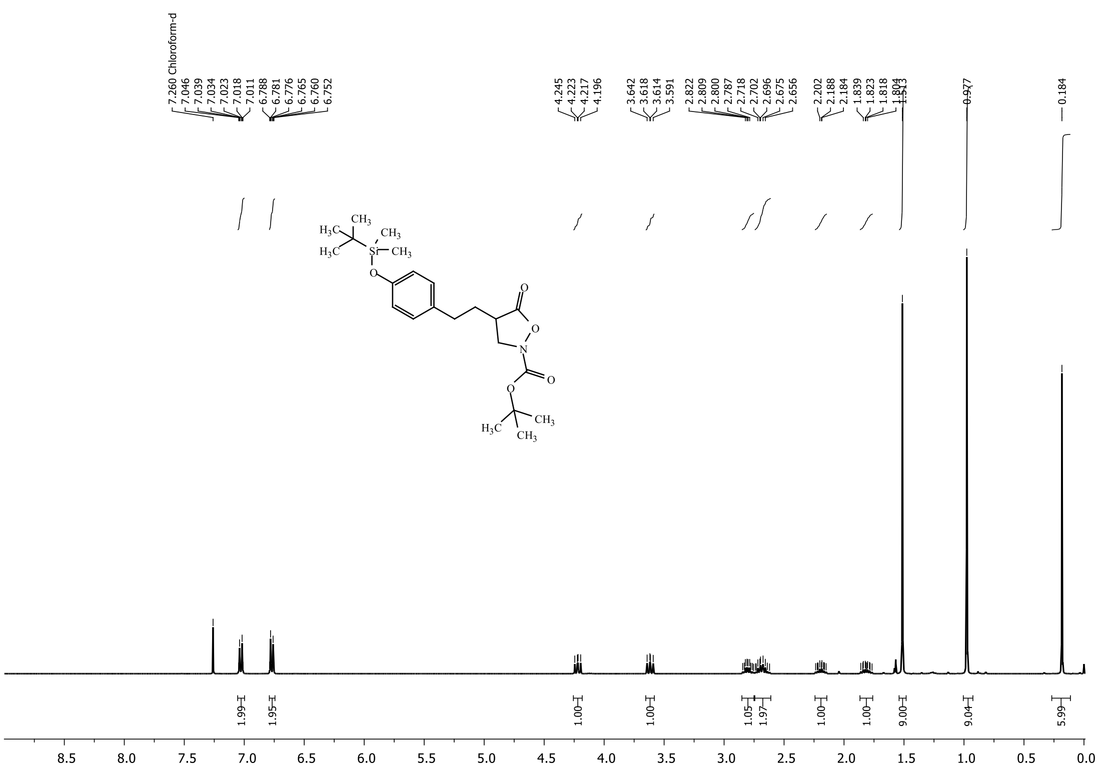

^{13}C NMR (101 MHz, $CDCl_3$)

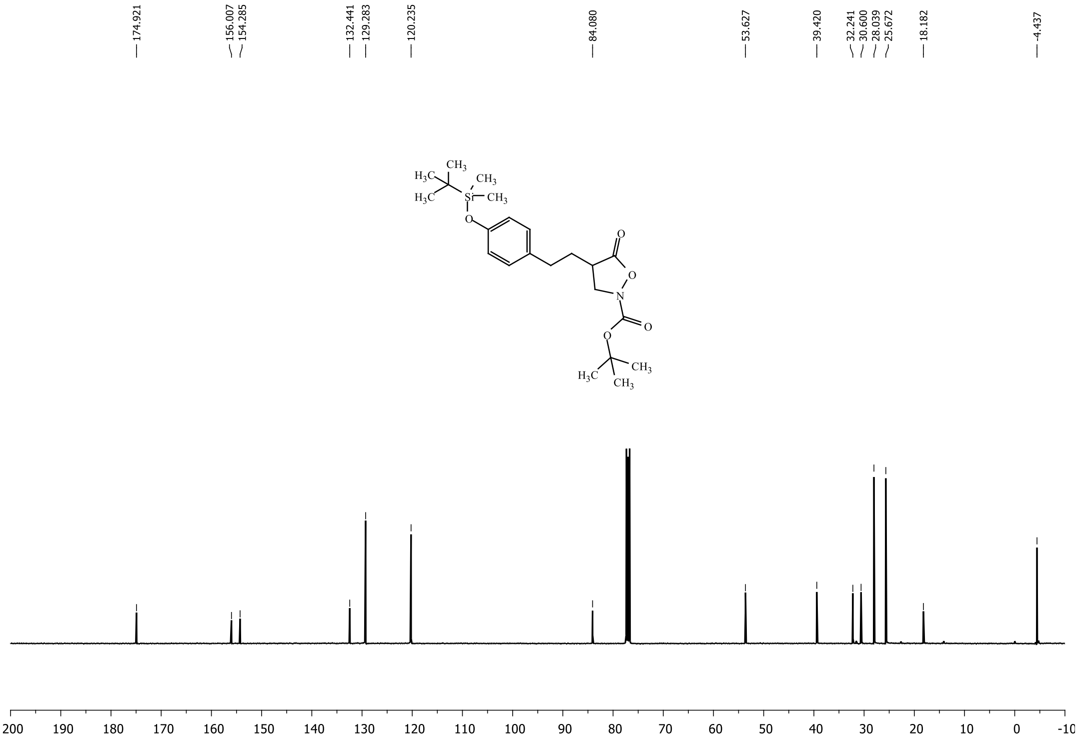

***tert*-Butyl 4-benzyl-4-methyl-5-oxoisoxazolidine-2-carboxylate (3a)**

^{1}H NMR (400 MHz, CDCl$_3$)

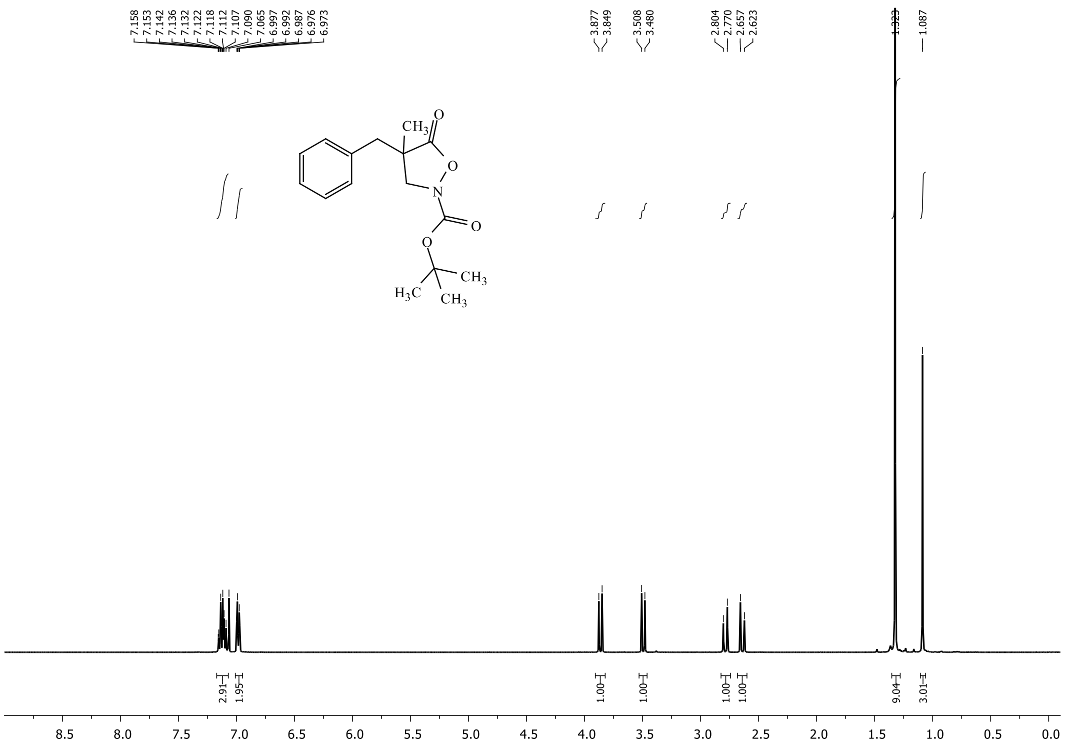

^{13}C NMR (101 MHz, CDCl$_3$)

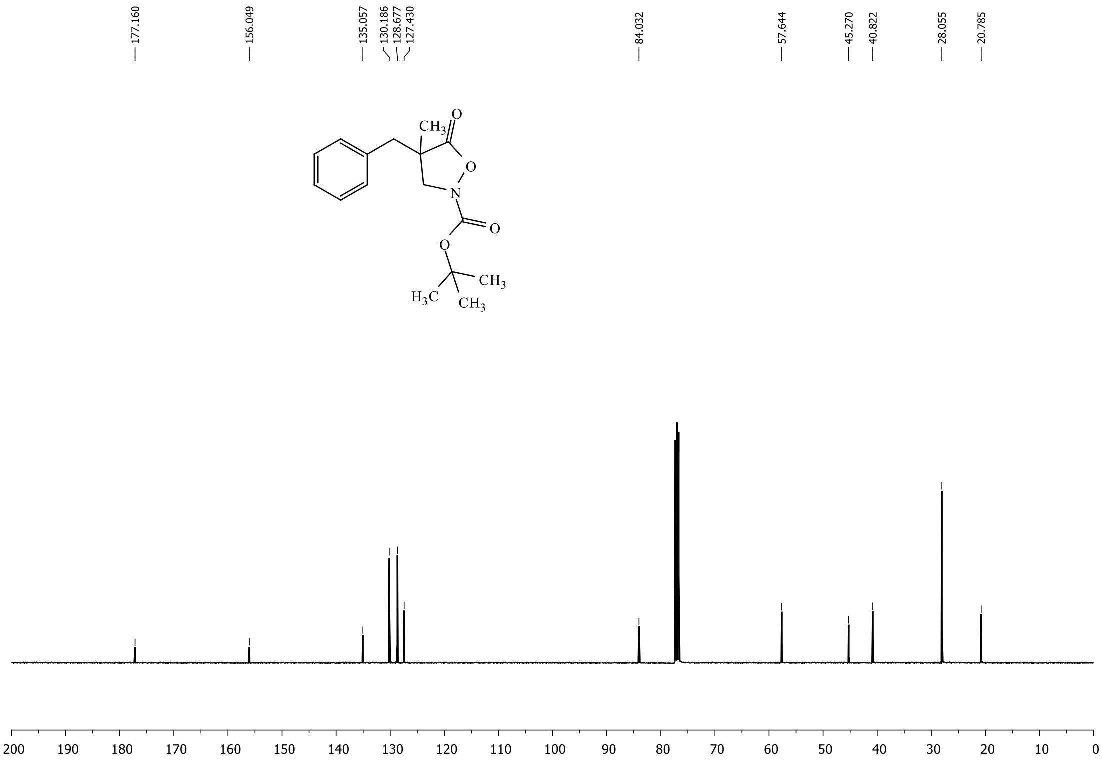

***tert*-Butyl 4-benzyl-4-hexyl-5-oxoisoxazolidine-2-carboxylate (3b)**

^{1}H NMR (400 MHz, $CDCl_3$)

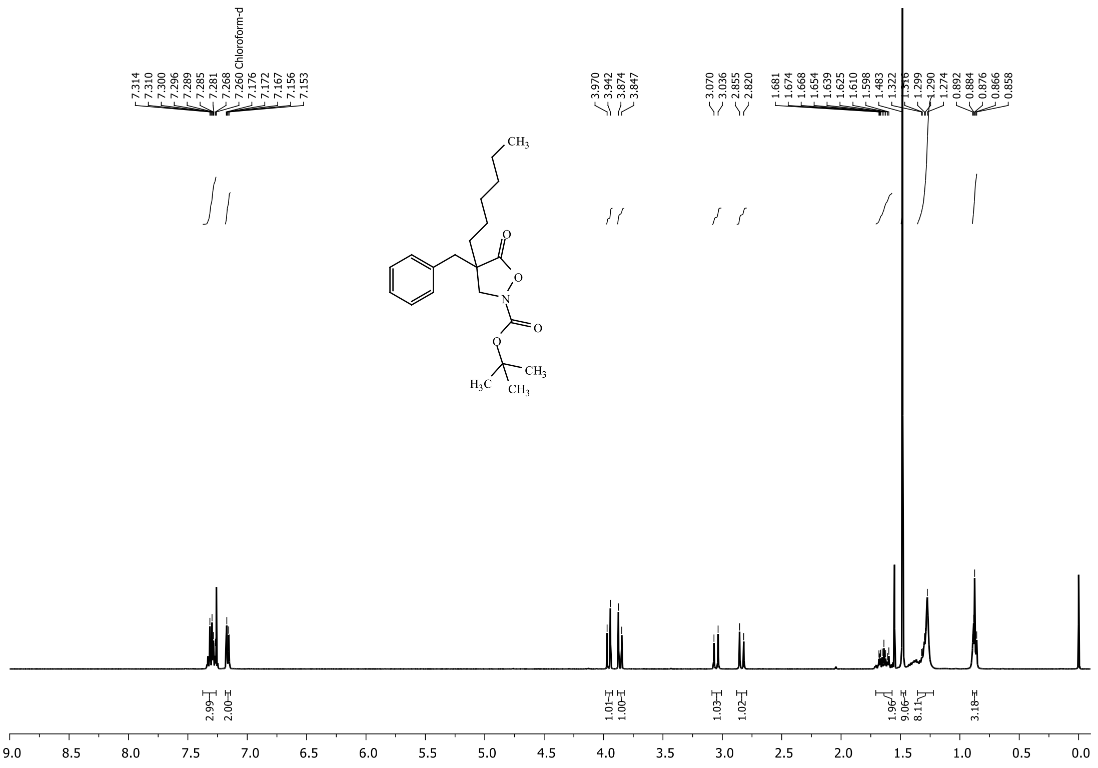

^{13}C NMR (101 MHz, $CDCl_3$)

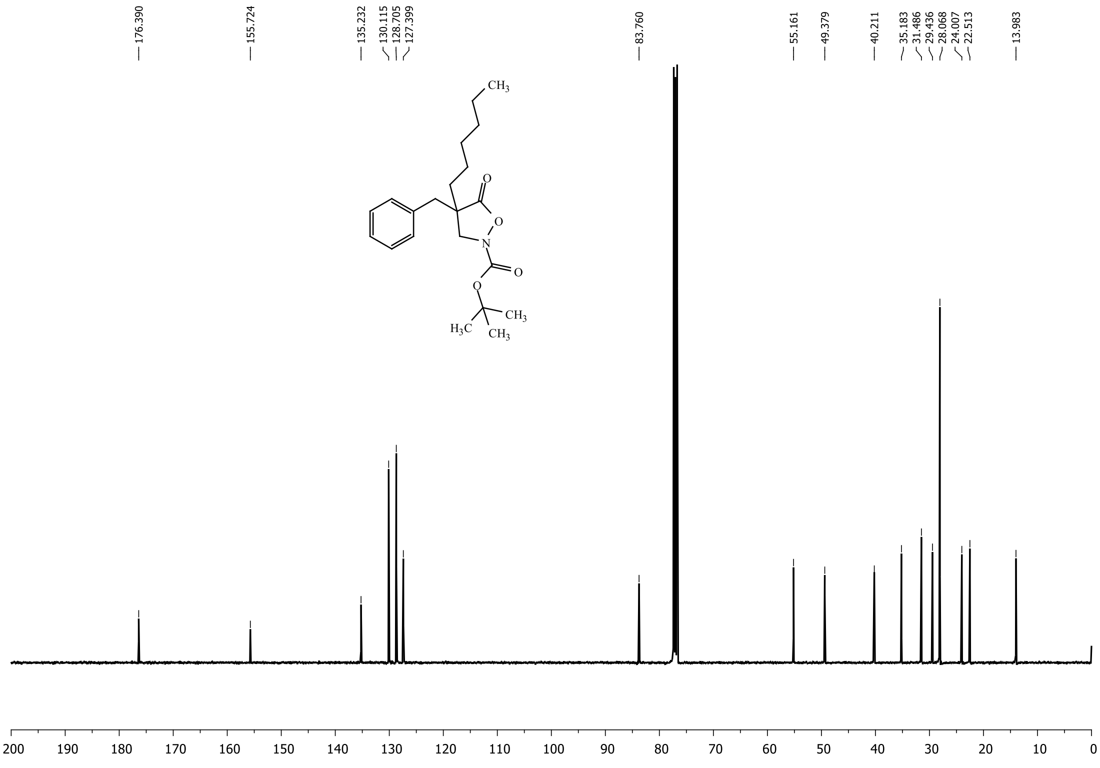

tert-Butyl 4-benzyl-5-oxo-4-phenethylisoxazolidine-2-carboxylate (3c)

^{1}H NMR (400 MHz, $CDCl_3$)

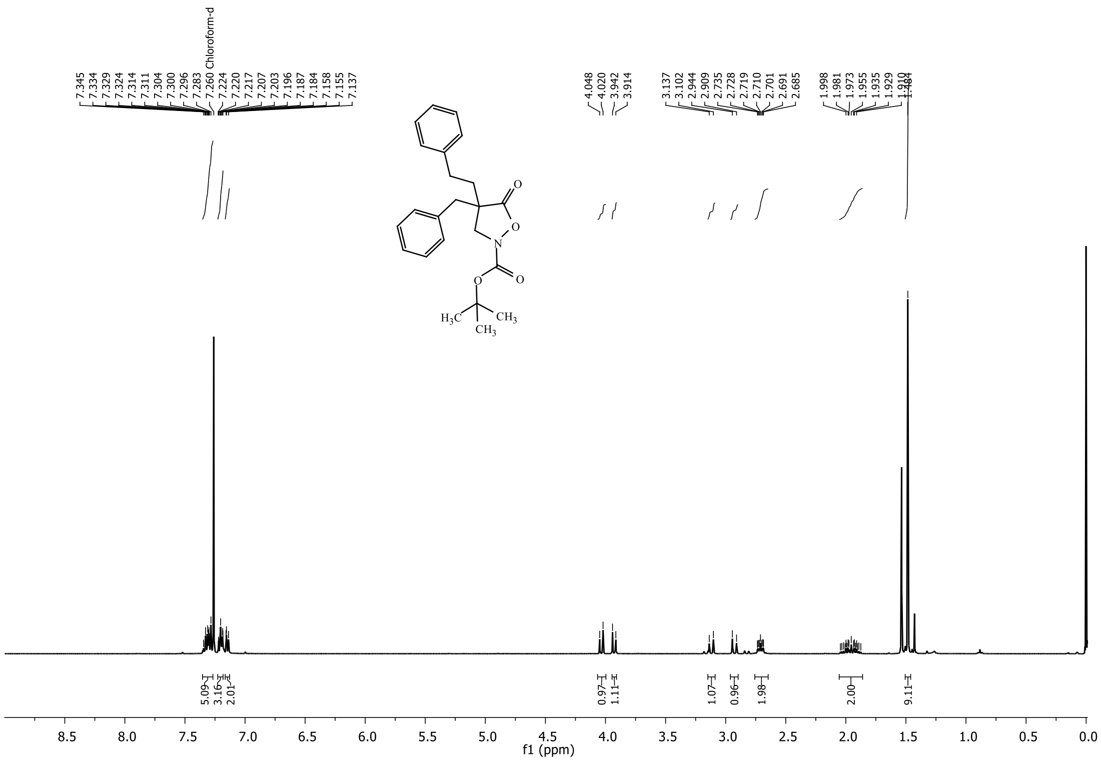

^{13}C NMR (101 MHz, $CDCl_3$)

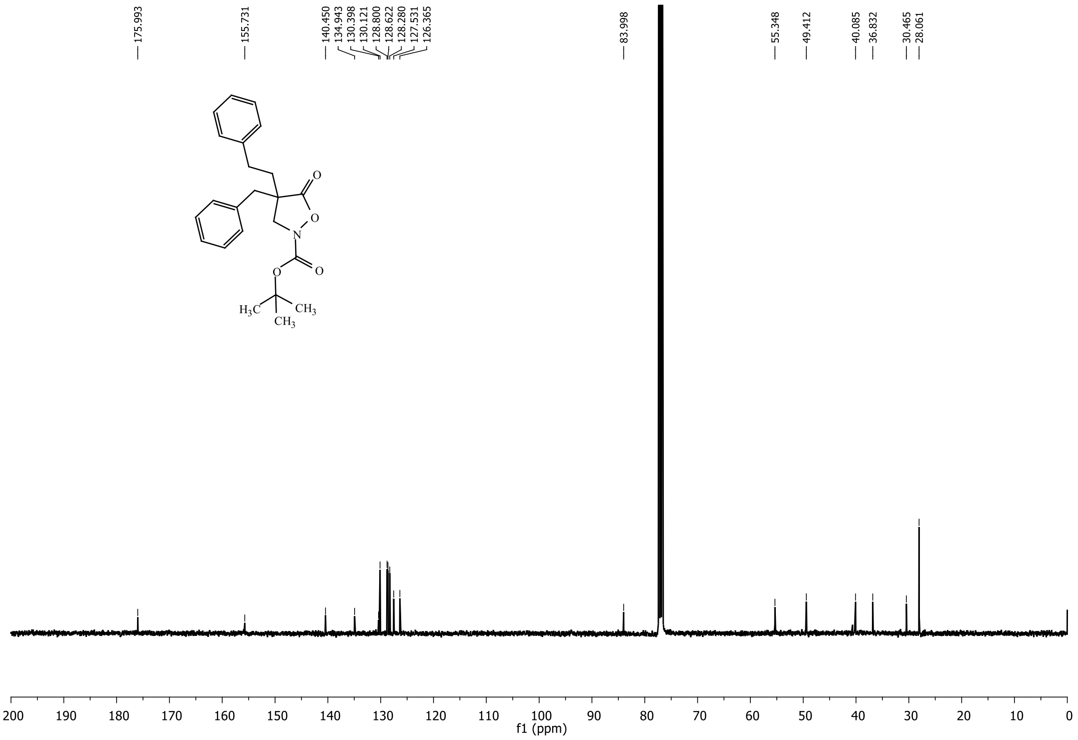

***tert*-Butyl 4-benzyl-5-oxo-4-(pent-4-en-1-yl)isoxazolidine-2-carboxylate (3g)**

^{1}H NMR (400 MHz, CDCl$_3$)

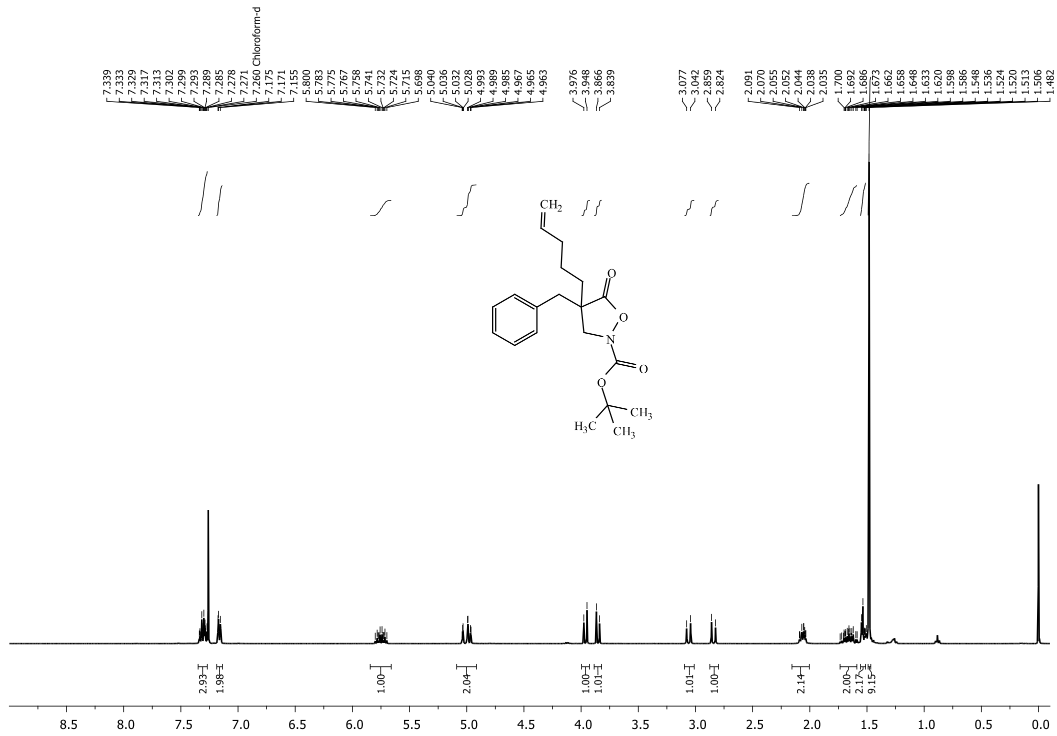

^{13}C NMR (101 MHz, CDCl$_3$)

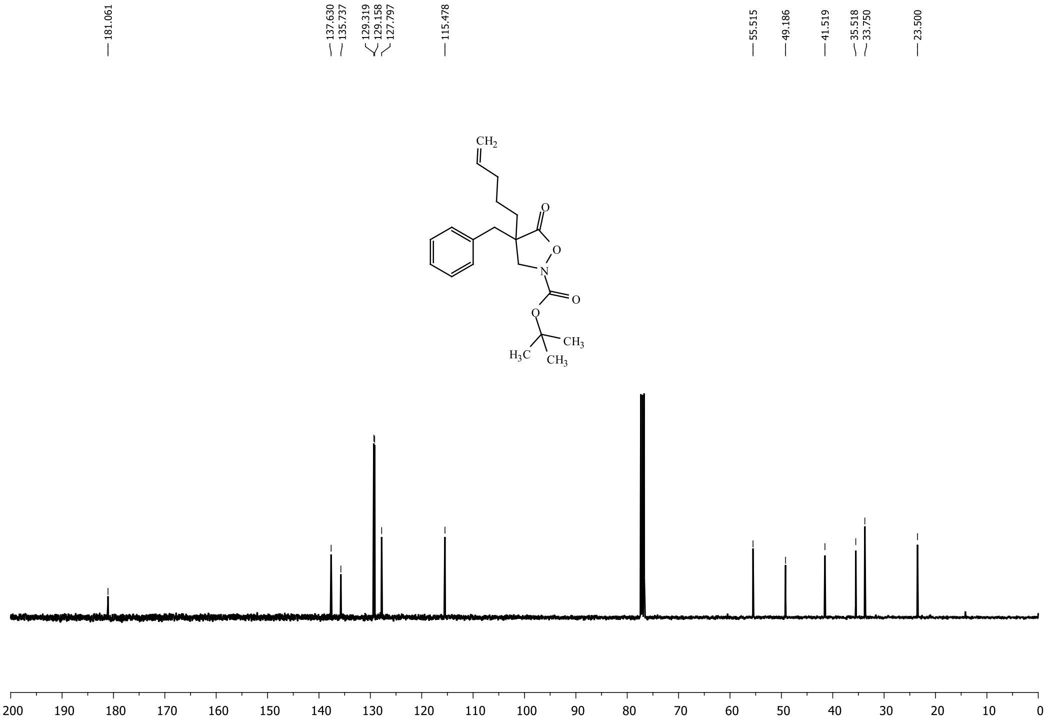

tert-Butyl 4-benzyl-4-(cyanomethyl)-5-oxoisoxazolidine-2-carboxylate (3h)

^{1}H NMR (400 MHz, $CDCl_3$)

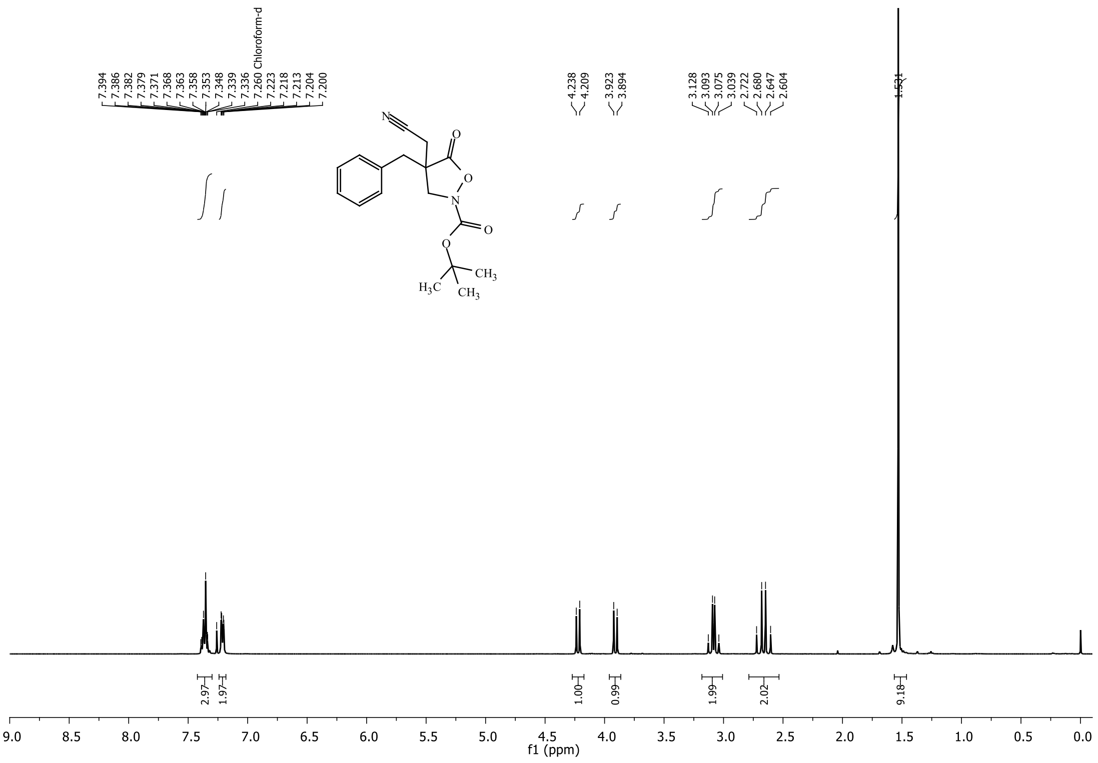

^{13}C NMR (101 MHz, $CDCl_3$)

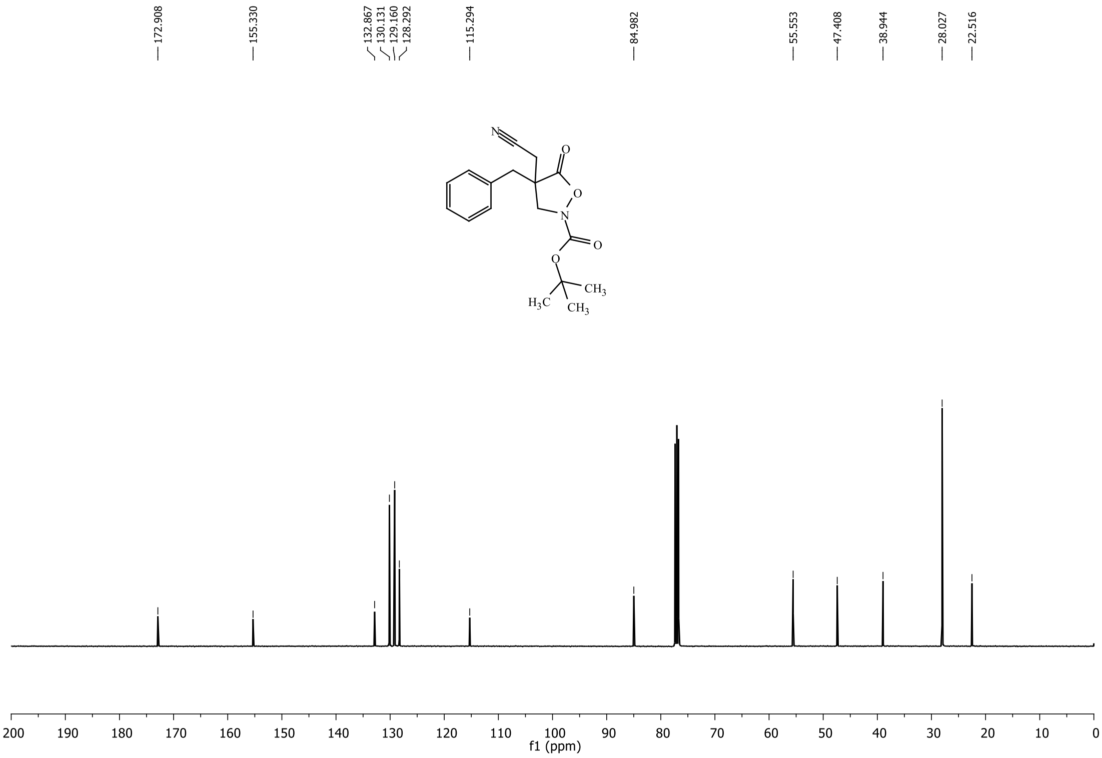

tert-Butyl 4-benzyl-5-oxo-4-(prop-2-yn-1-yl)isoxazolidine-2-carboxylate (3i)

^{1}H NMR (400 MHz, CDCl$_3$)

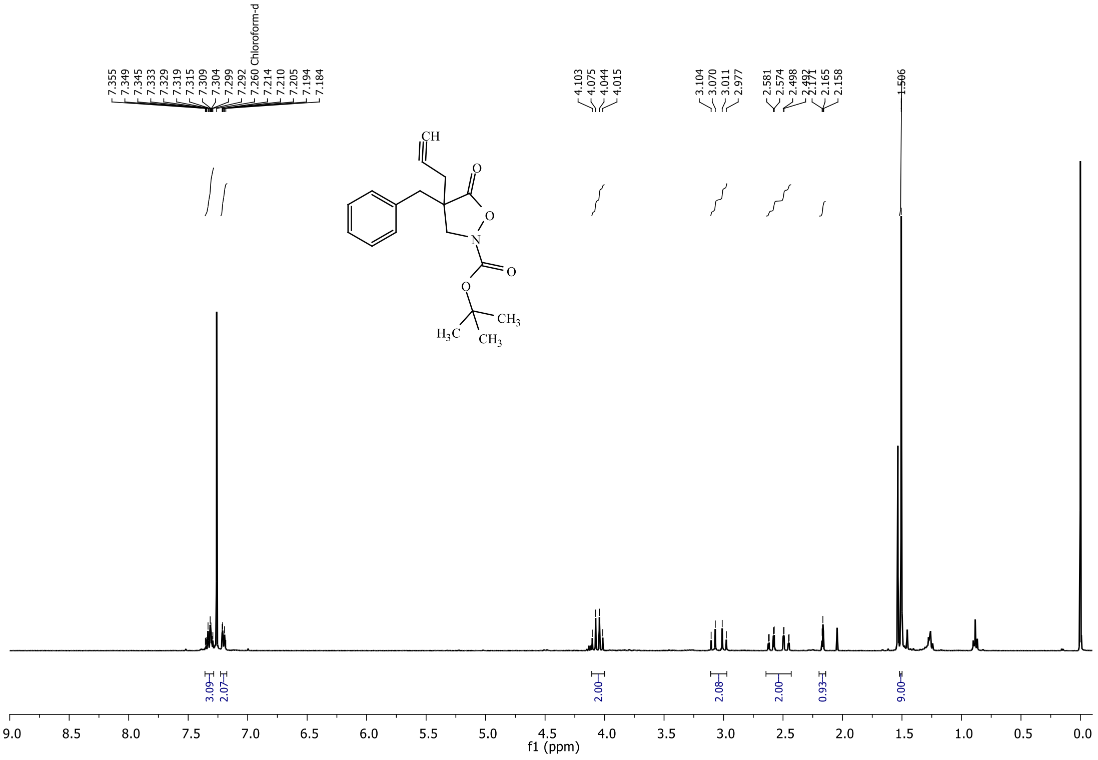

^{13}C NMR (101 MHz, CDCl$_3$)

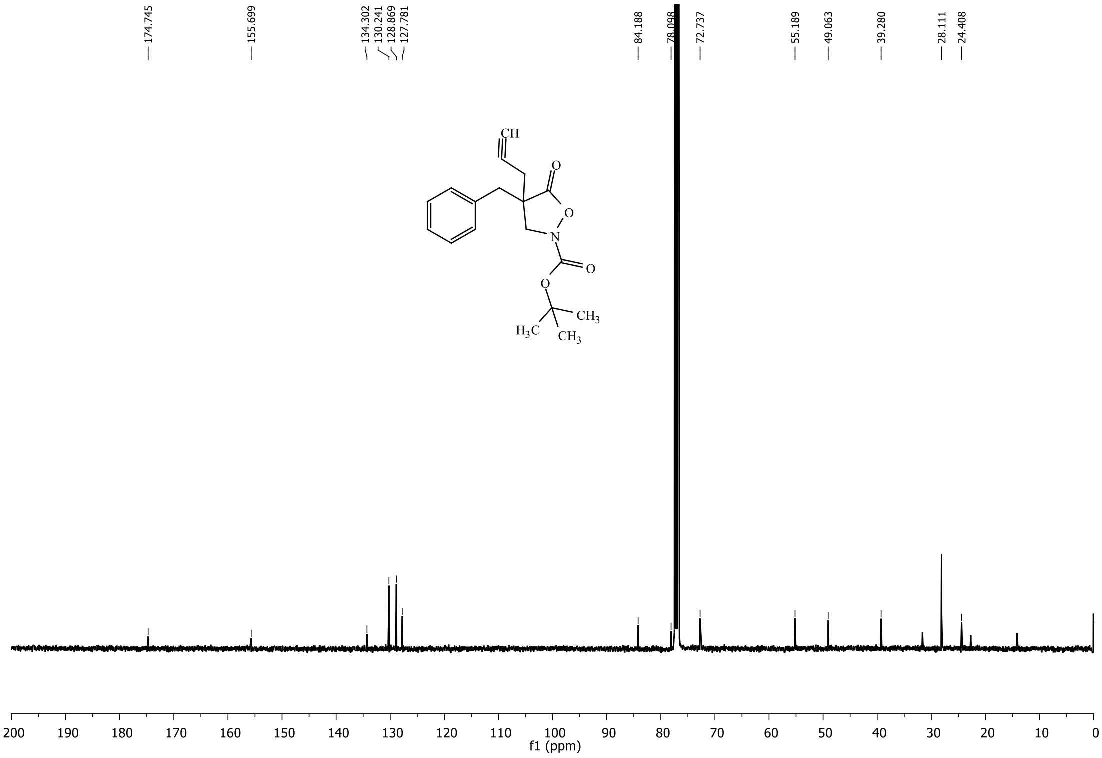

***tert*-Butyl 4-methyl-4-(4-methylbenzyl)-5-oxoisoxazolidine-2-carboxylate (3j)**

^{1}H NMR (400 MHz, $CDCl_3$)

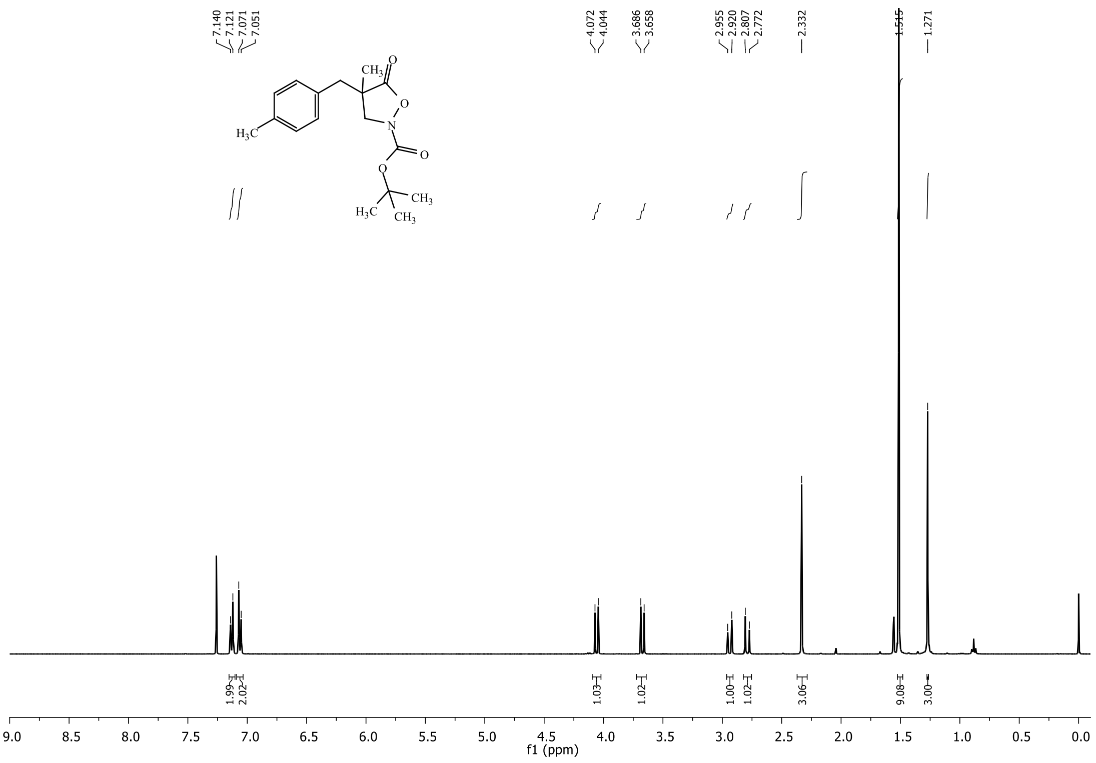

^{13}C NMR (101 MHz, $CDCl_3$)

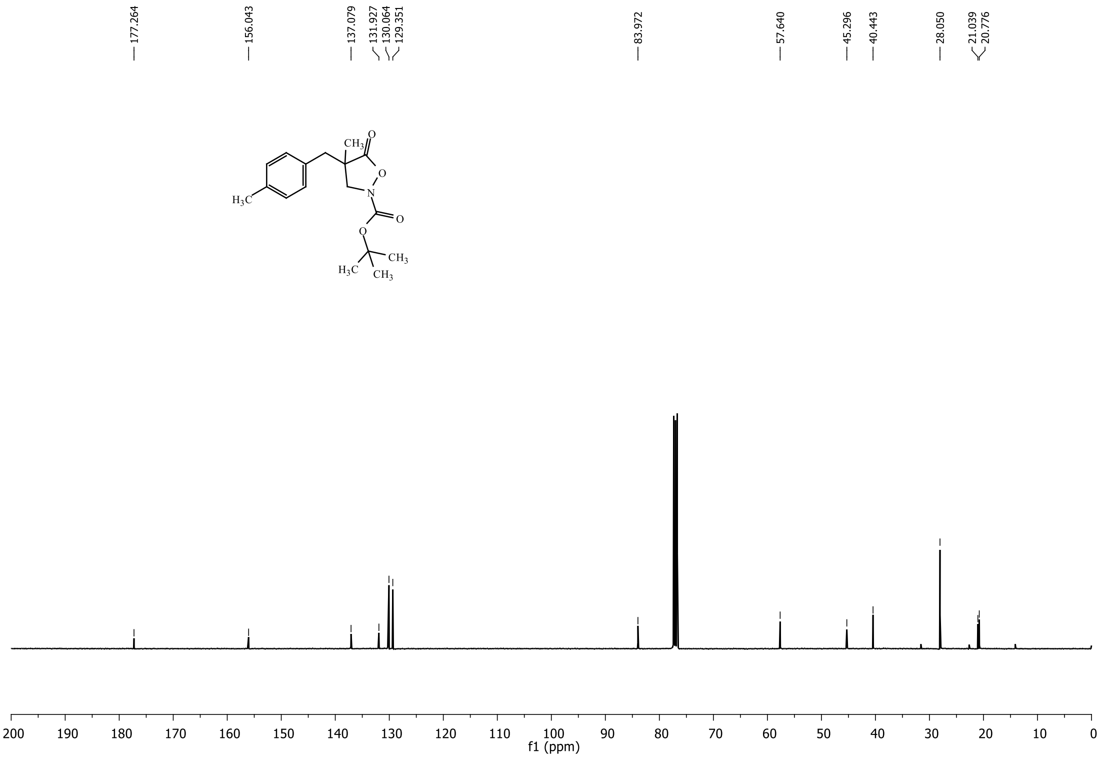

tert-Butyl 4-methyl-4-(2-methylbenzyl)-5-oxoisoxazolidine-2-carboxylate (3k)

^{1}H NMR (400 MHz, $CDCl_3$)

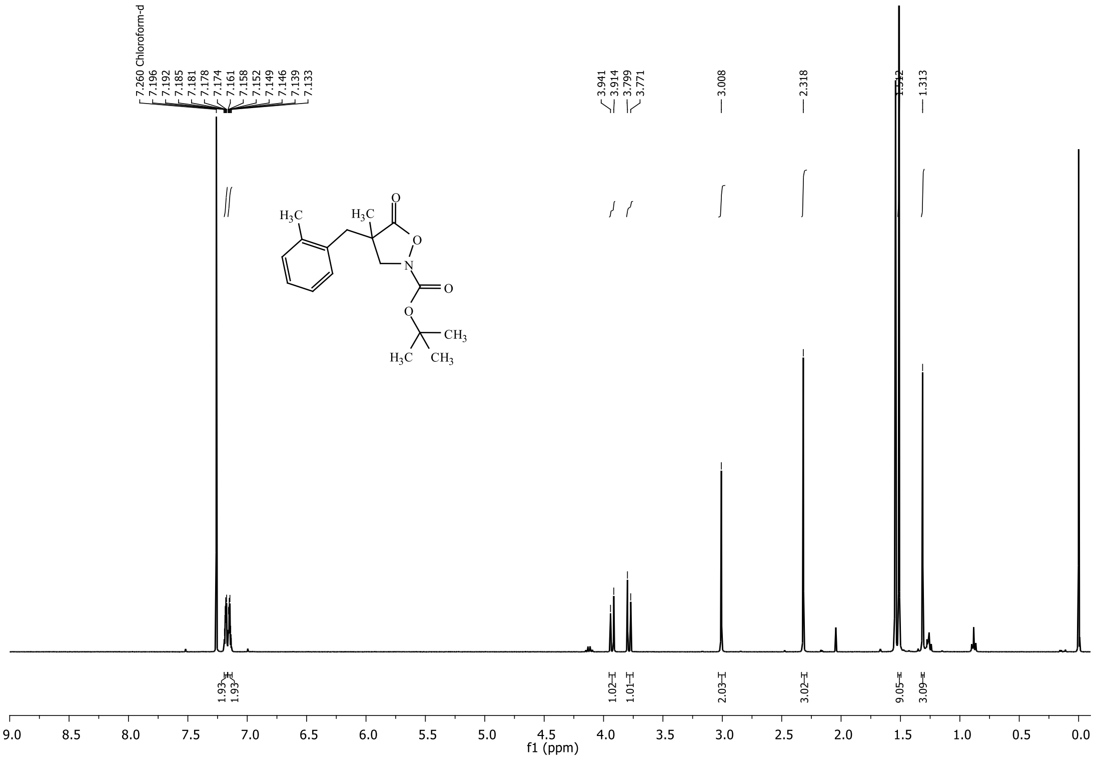

^{13}C NMR (101 MHz, $CDCl_3$)

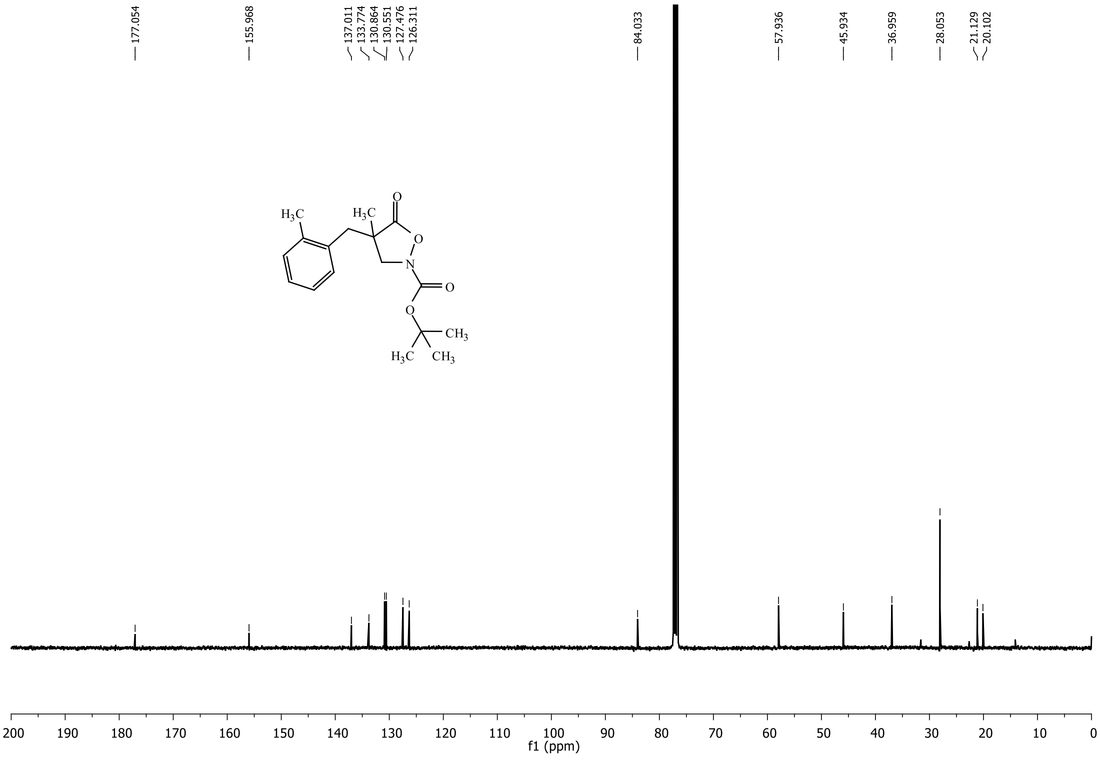

tert-Butyl 4-(4-bromobenzyl)-4-methyl-5-oxoisoxazolidine-2-carboxylate (3l)

^{1}H NMR (400 MHz, $CDCl_3$)

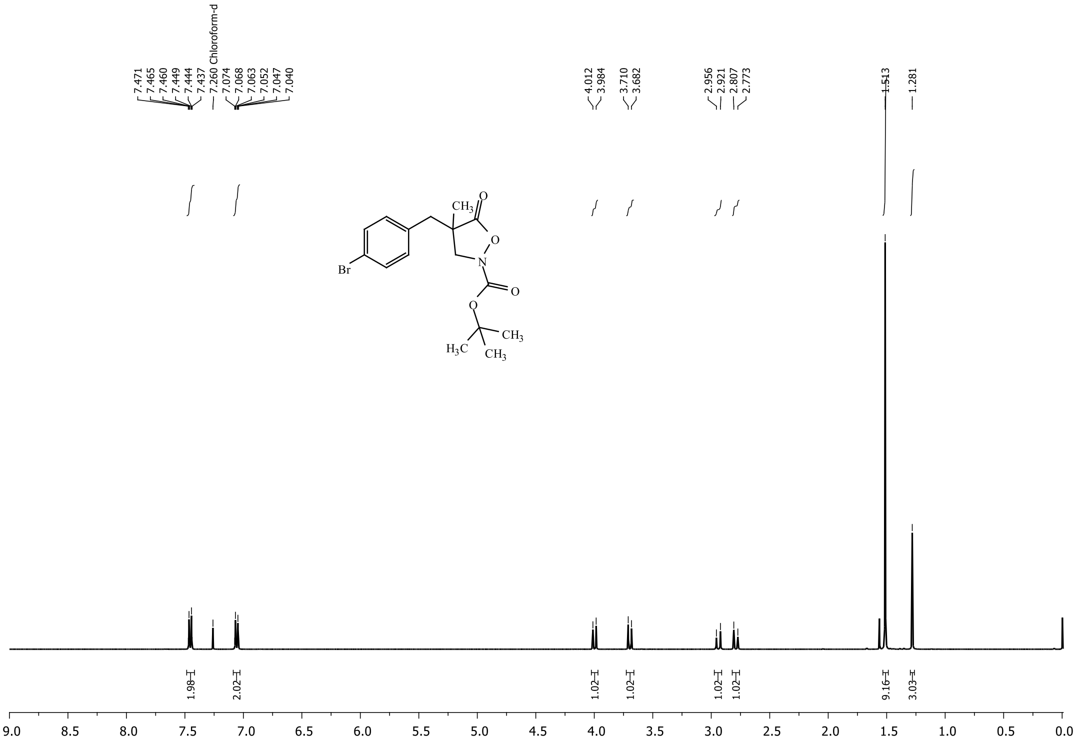

^{13}C NMR (101 MHz, $CDCl_3$)

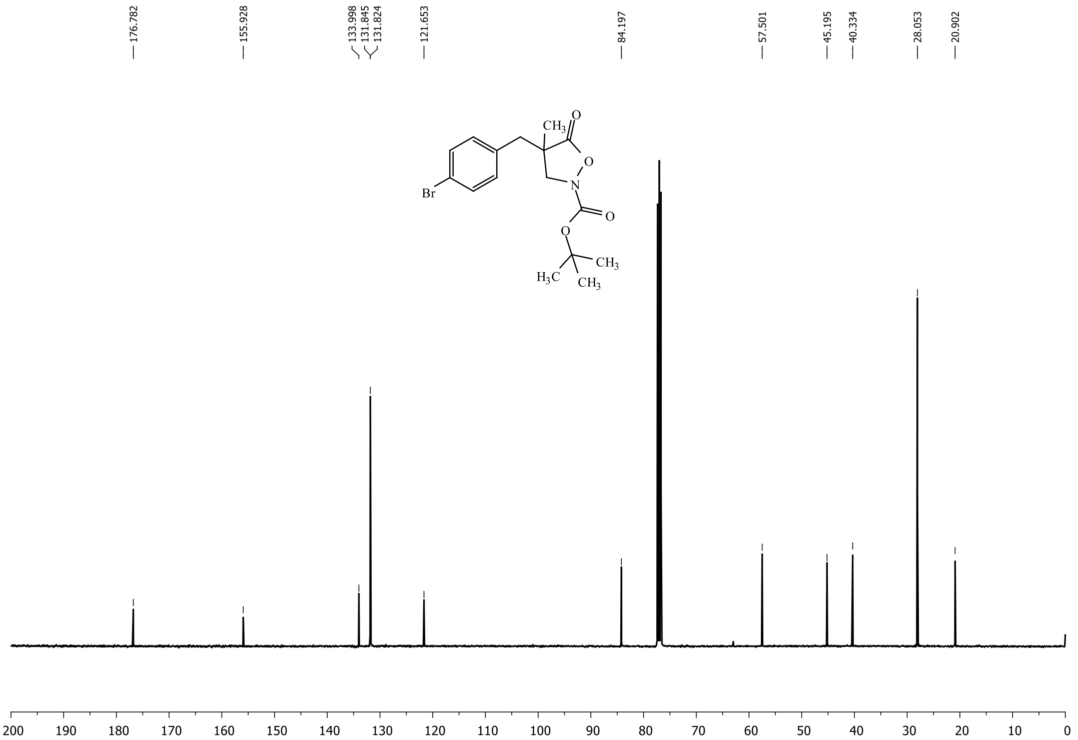

tert-Butyl 4-(2-chlorobenzyl)-4-methyl-5-oxoisoxazolidine-2-carboxylate (3m)

^{1}H NMR (400 MHz, $CDCl_3$)

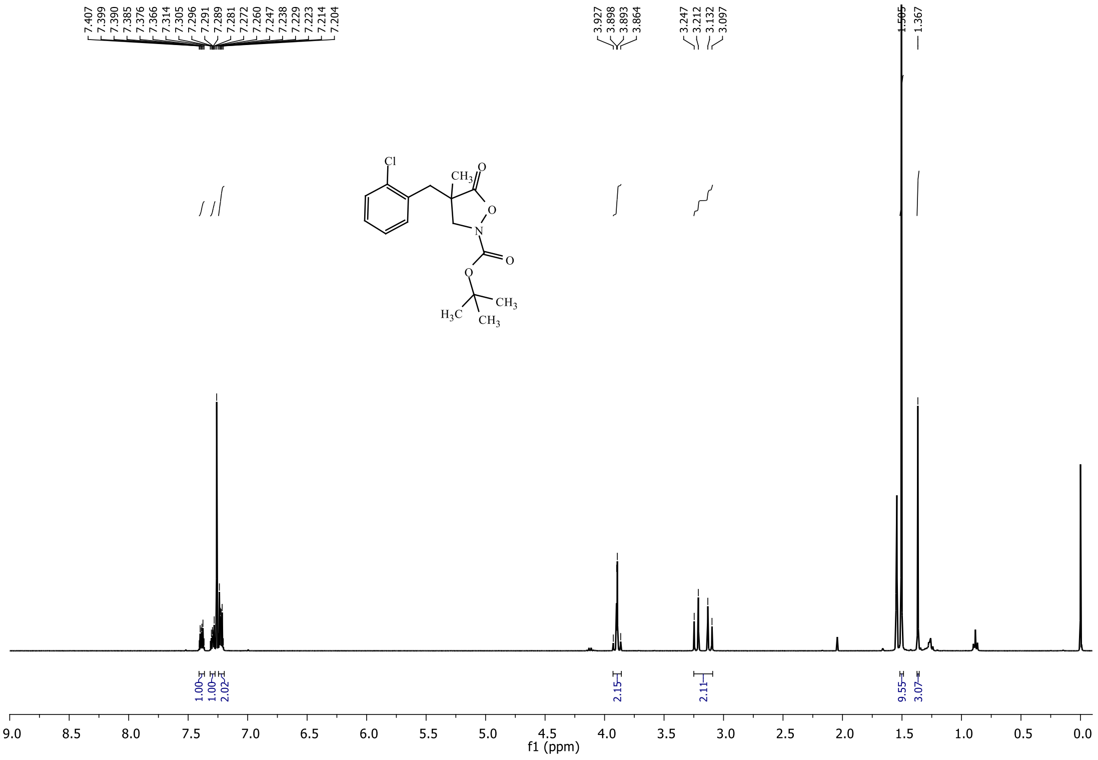

^{13}C NMR (101 MHz, $CDCl_3$)

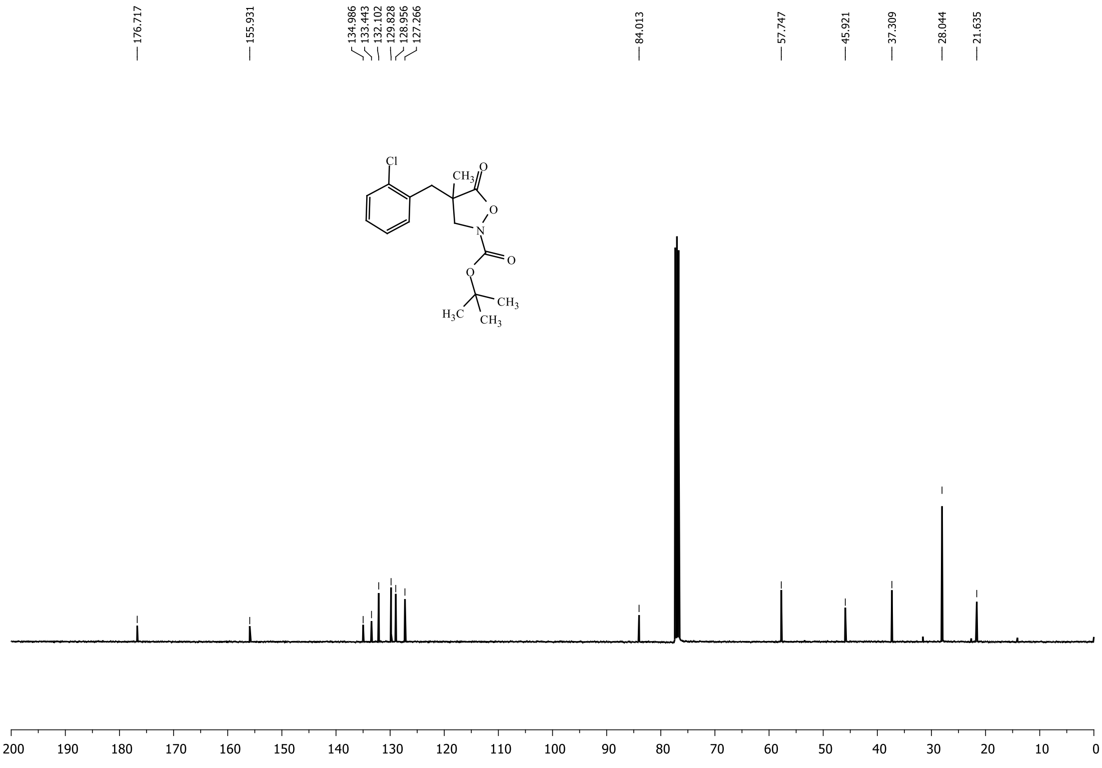

tert-Butyl 4-methyl-4-(3-methylbenzyl)-5-oxoisoxazolidine-2-carboxylate (3n)

^{1}H NMR (400 MHz, $CDCl_3$)

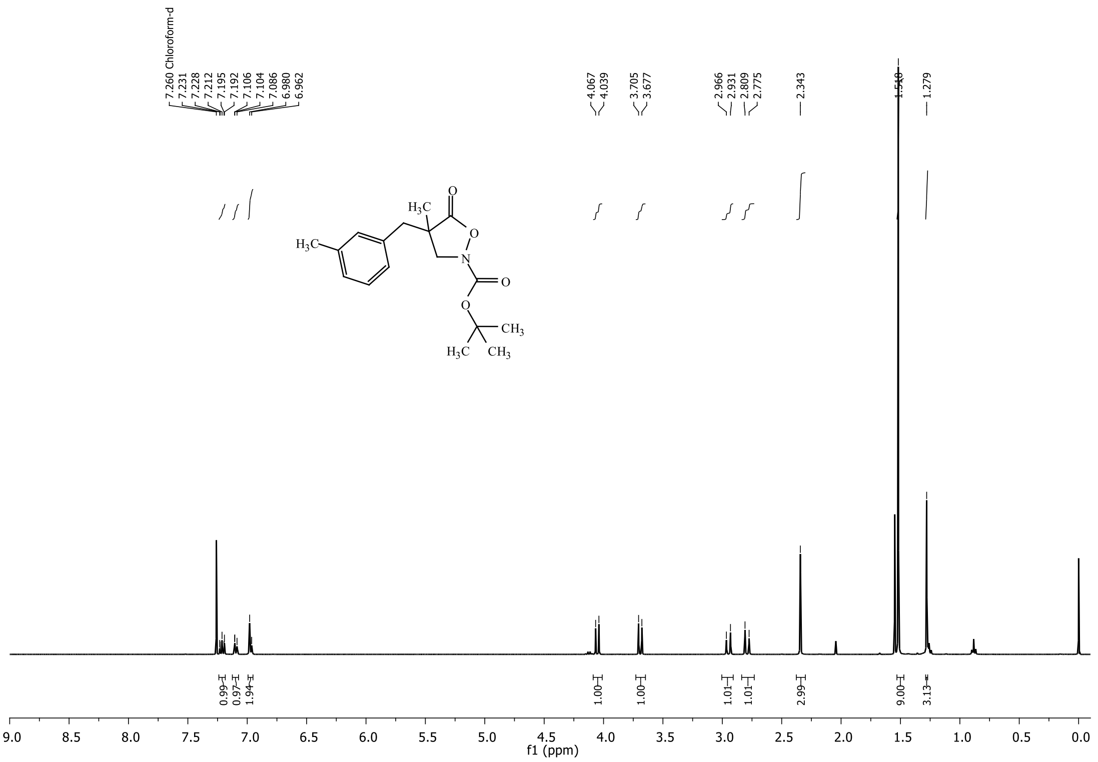

^{13}C NMR (101 MHz, $CDCl_3$)

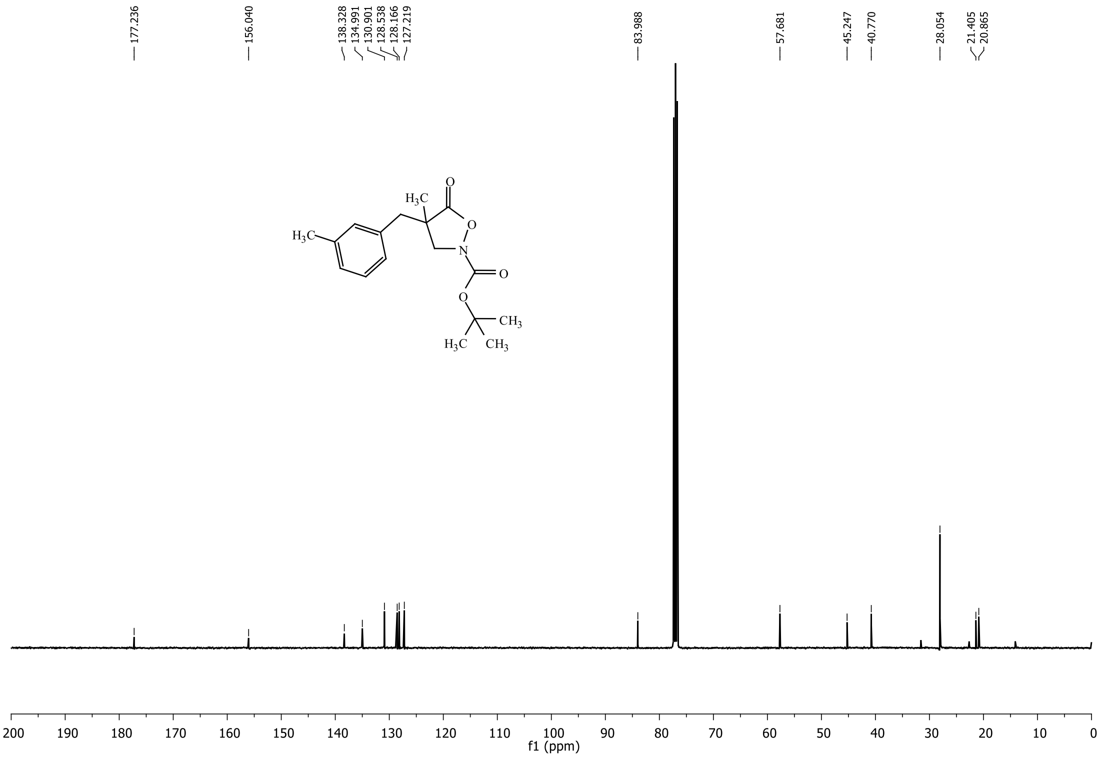

***tert*-Butyl 4-(3-methoxybenzyl)-4-methyl-5-oxoisoxazolidine-2-carboxylate (3o)**

^{1}H NMR (400 MHz, CDCl$_3$)

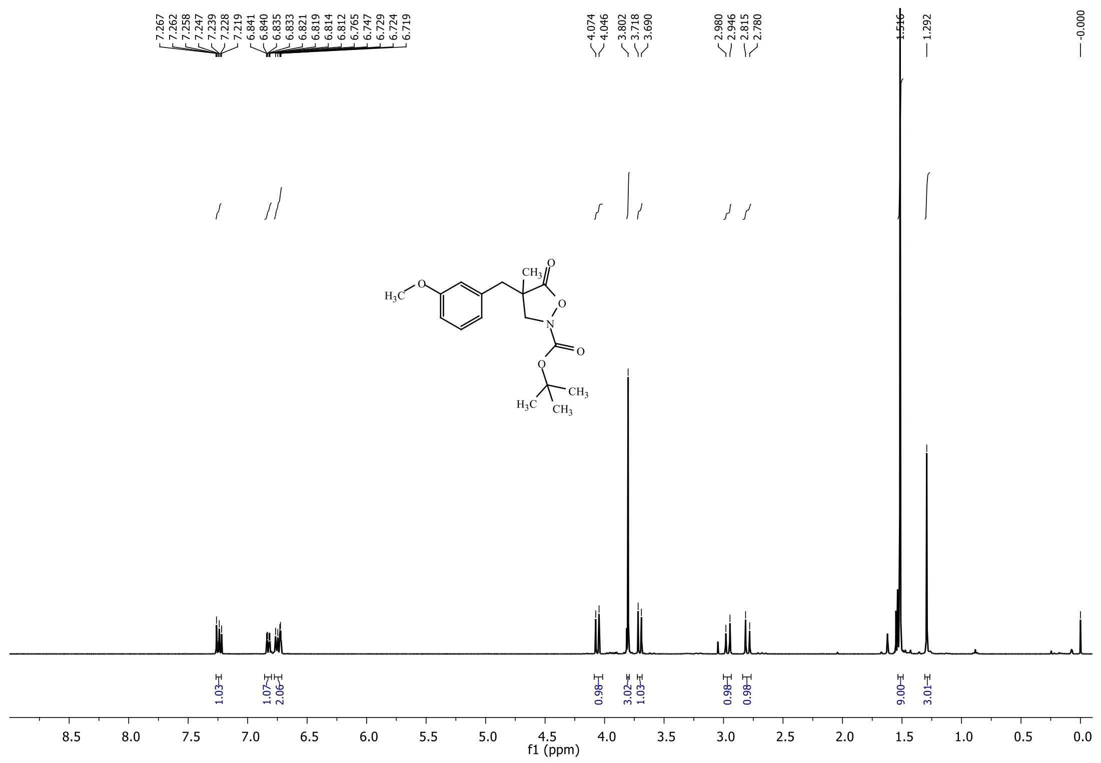

^{13}C NMR (101 MHz, CDCl$_3$)

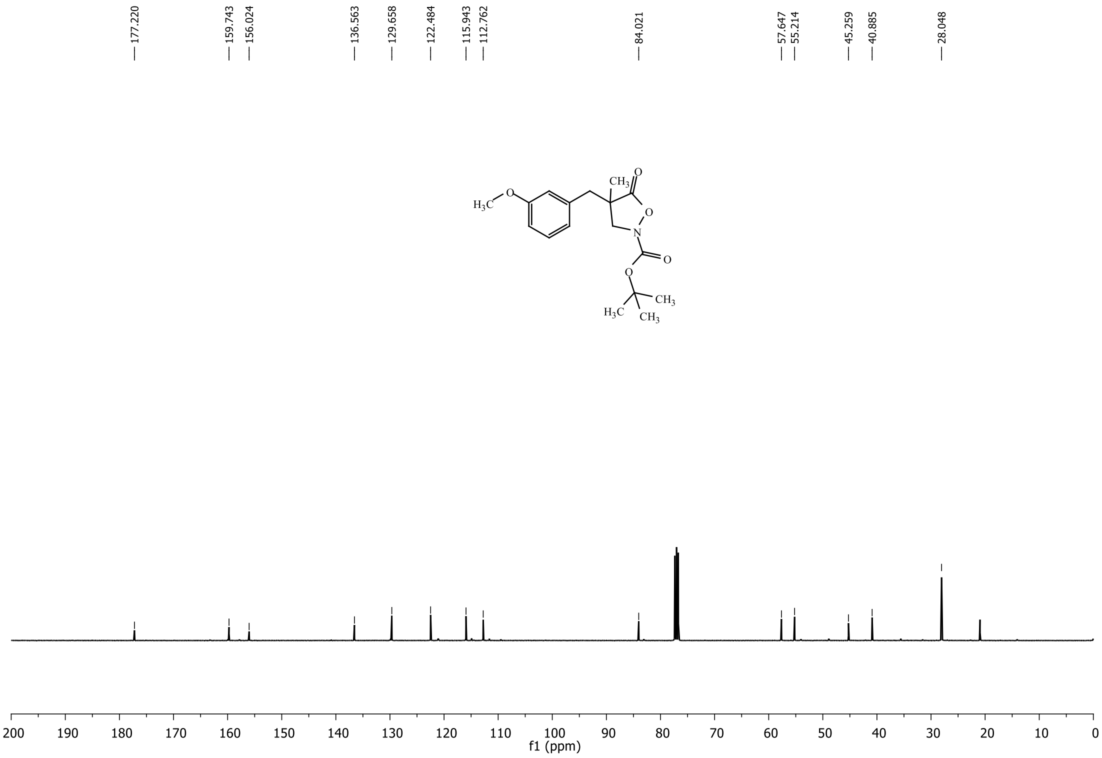

***tert*-Butyl 4-(3-fluorobenzyl)-4-methyl-5-oxoisoxazolidine-2-carboxylate (3p)**

^{1}H NMR (400 MHz, $CDCl_3$)

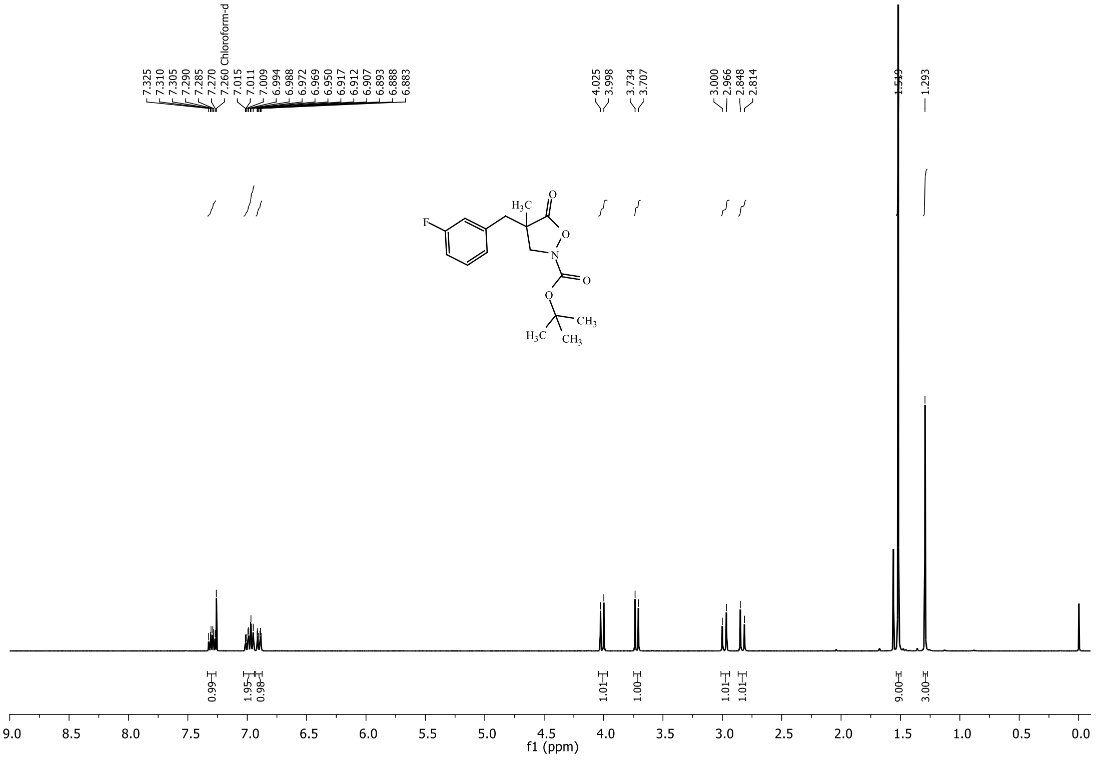

^{13}C NMR (101 MHz, $CDCl_3$)

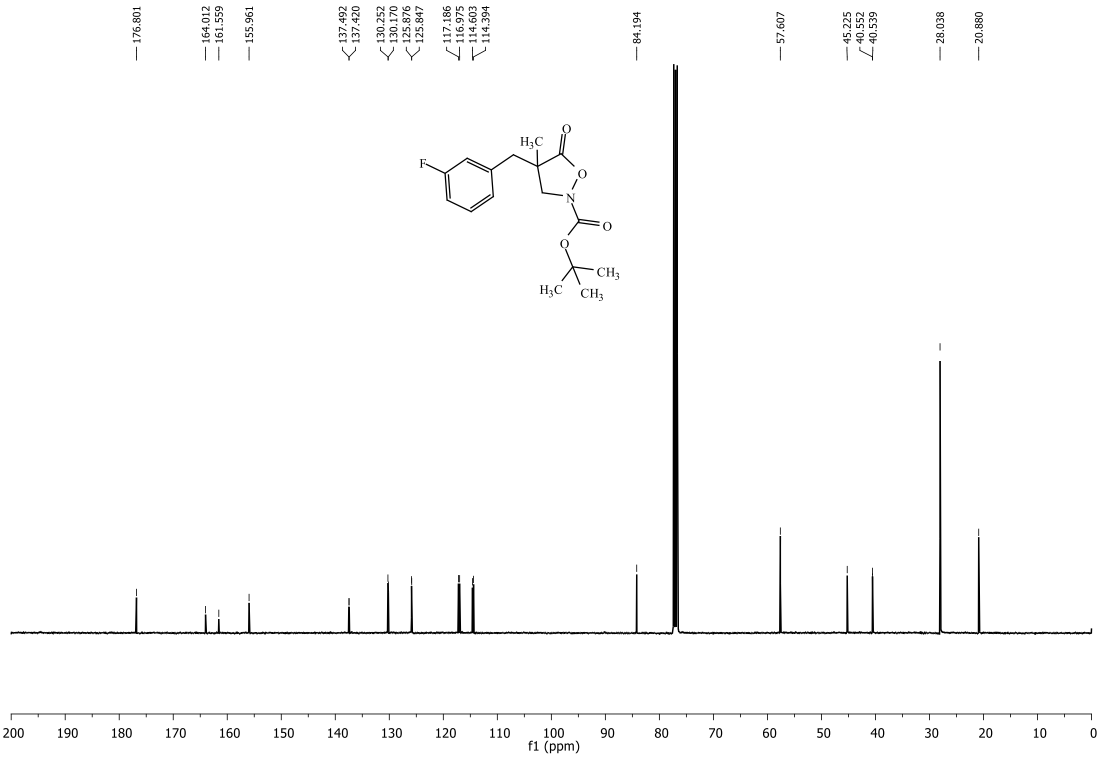

^{19}F NMR (376 MHz, $CDCl_3$)

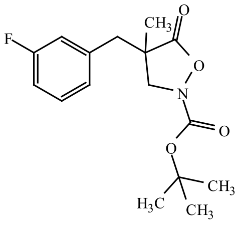

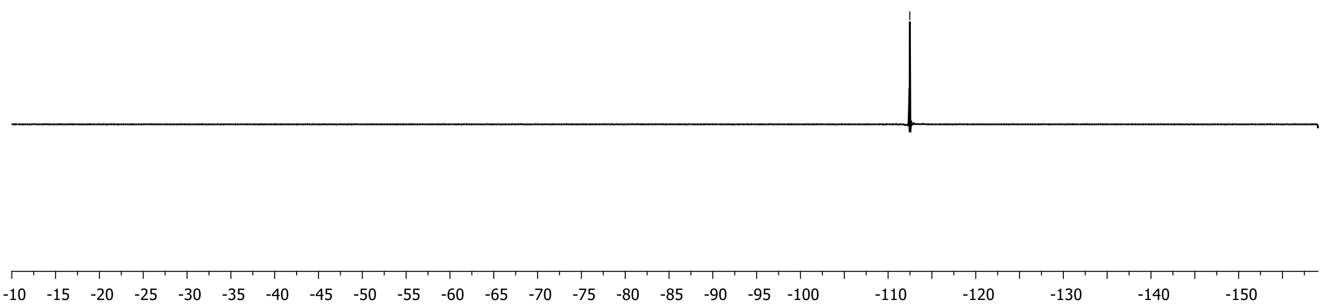

tert-Butyl 4-methyl-5-oxo-4-(4-(4,4,5,5-tetramethyl-1,3-dioxolan-2-yl)benzyl)isoxazolidine-2-carboxylate (3q)

^{1}H NMR (400 MHz, $CDCl_3$)

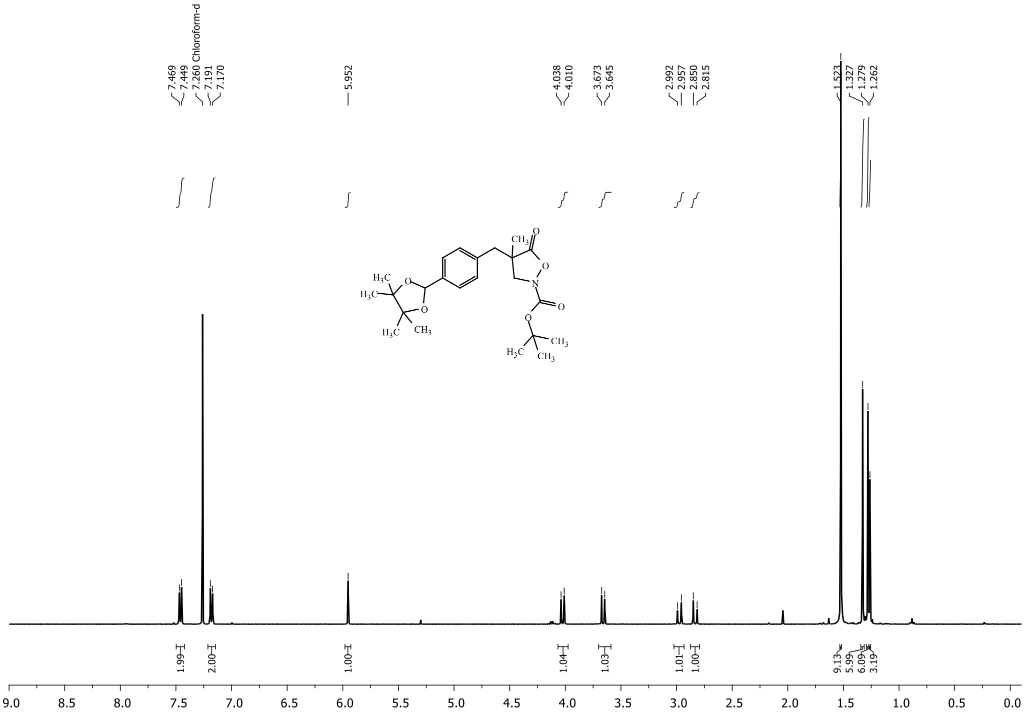

^{13}C NMR (101 MHz, $CDCl_3$)

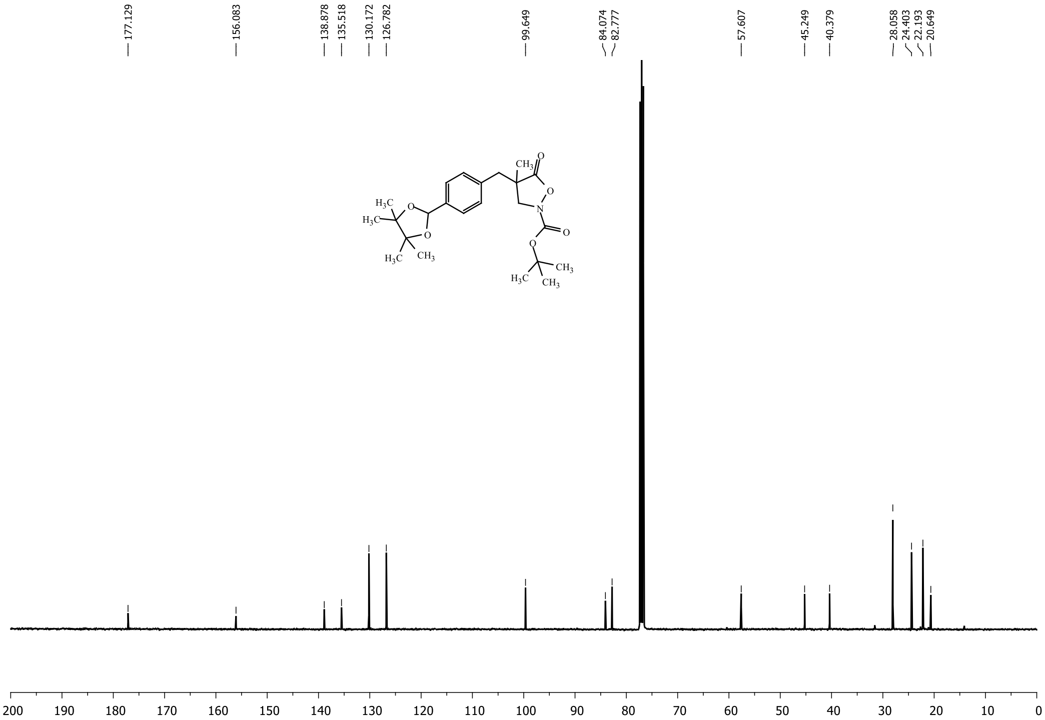

tert-Butyl 4-methyl-5-oxo-4-(4-(4,4,5,5-tetramethyl-1,3-dioxolan-2-yl)benzyl)isoxazolidine-2-carboxylate (3r)

^{1}H NMR (400 MHz, CDCl$_3$)

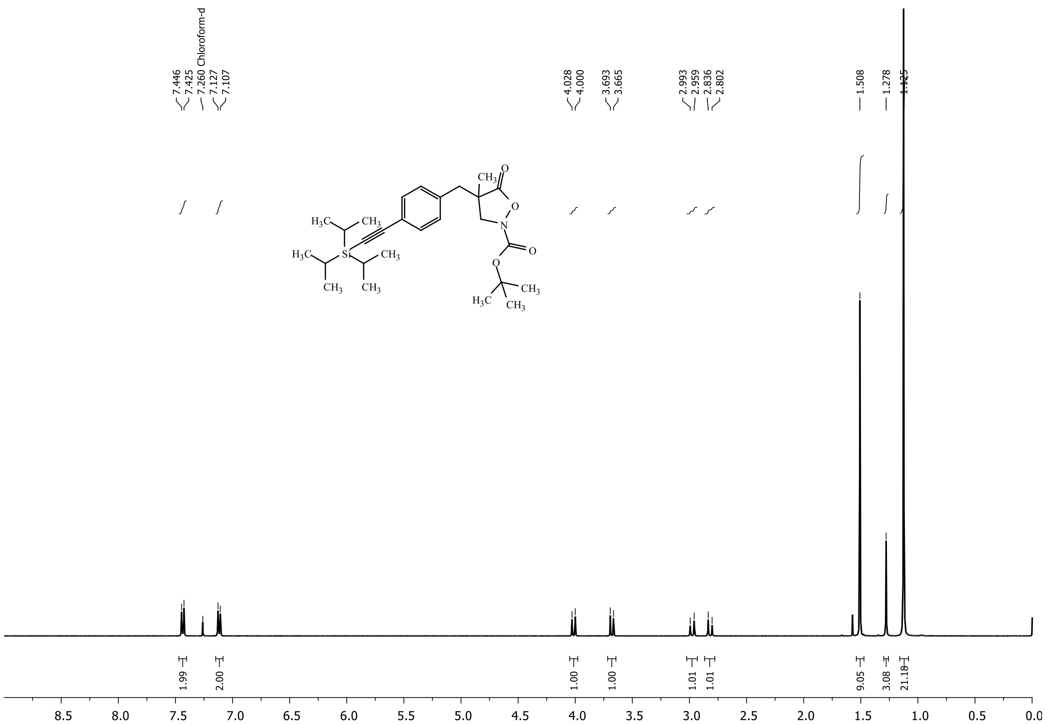

^{13}C NMR (101 MHz, CDCl$_3$)

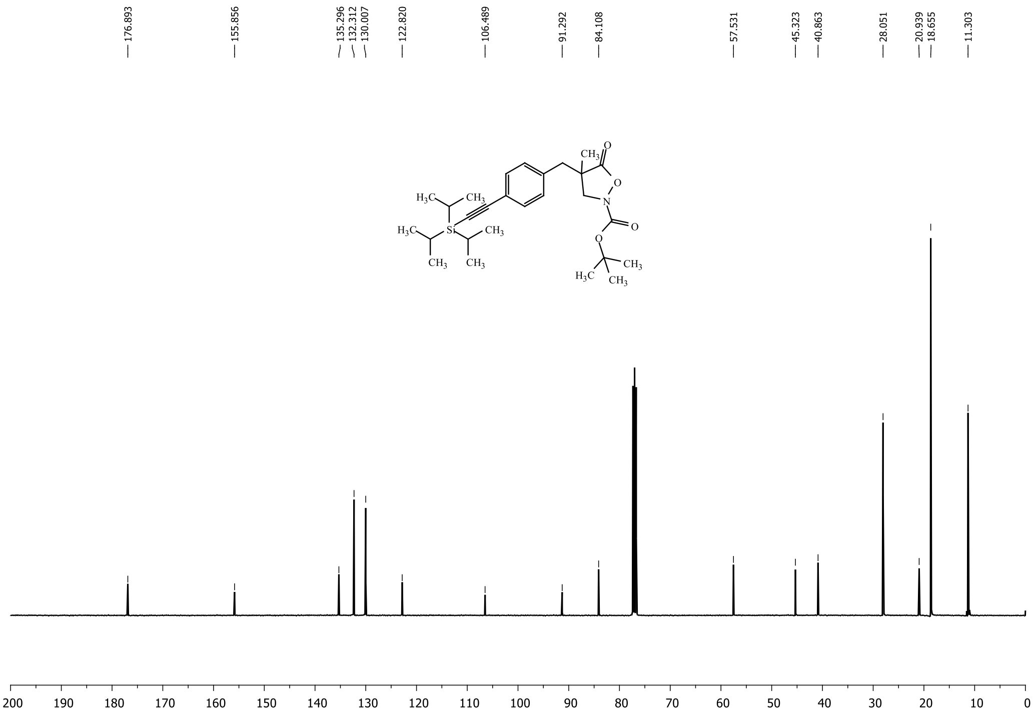

***tert*-Butyl 4-(4-(tert-butoxy)benzyl)-4-methyl-5-oxoisoxazolidine-2-carboxylate (3t)**

^{1}H NMR (400 MHz, $CDCl_3$)

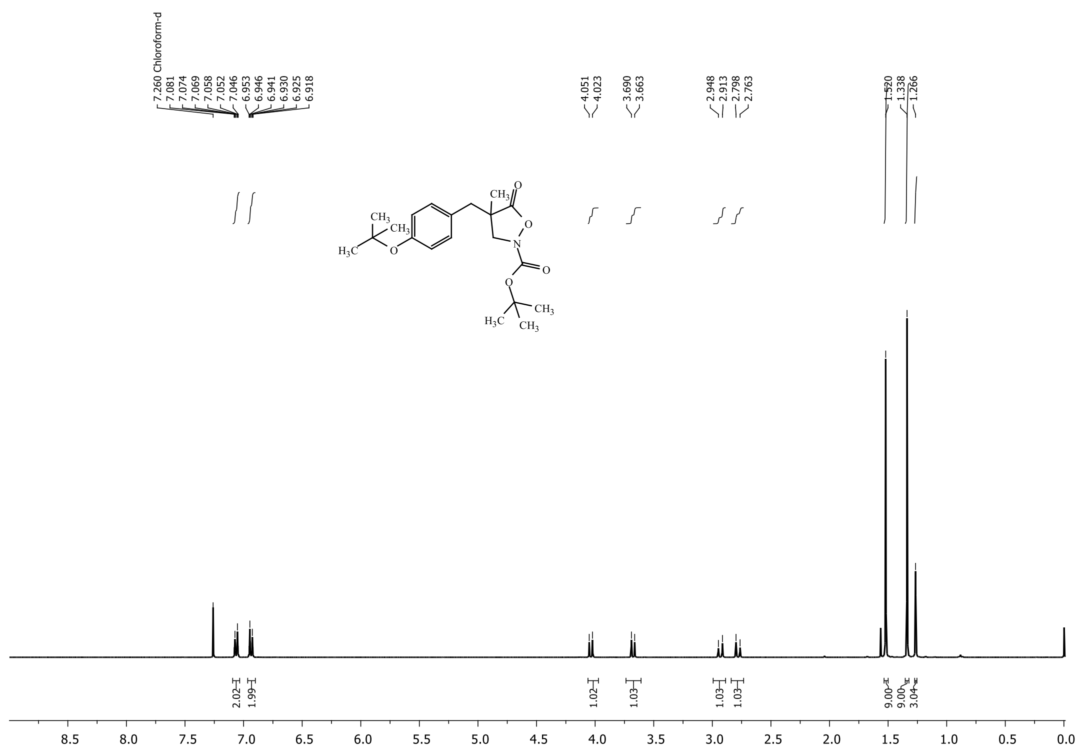

^{13}C NMR (101 MHz, $CDCl_3$)

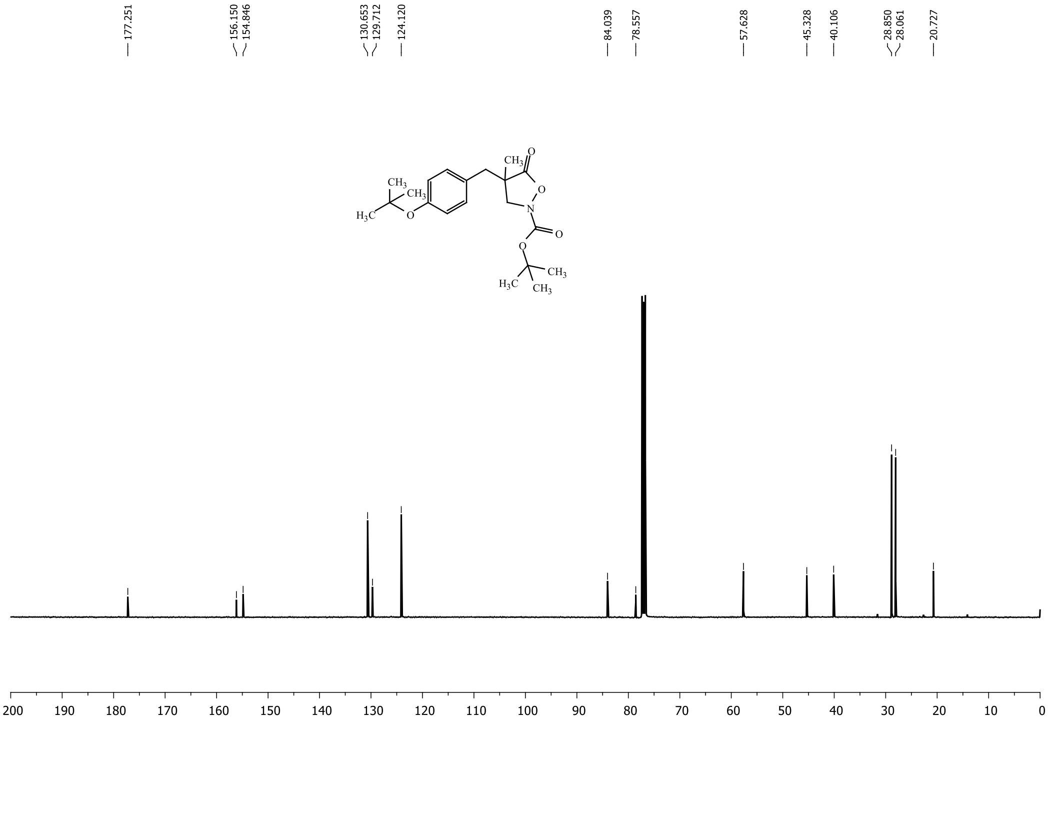

4-Benzyl-2,4-dimethylisoxazolidin-5-one (10):

^{1}H NMR (400 MHz, $CDCl_3$)

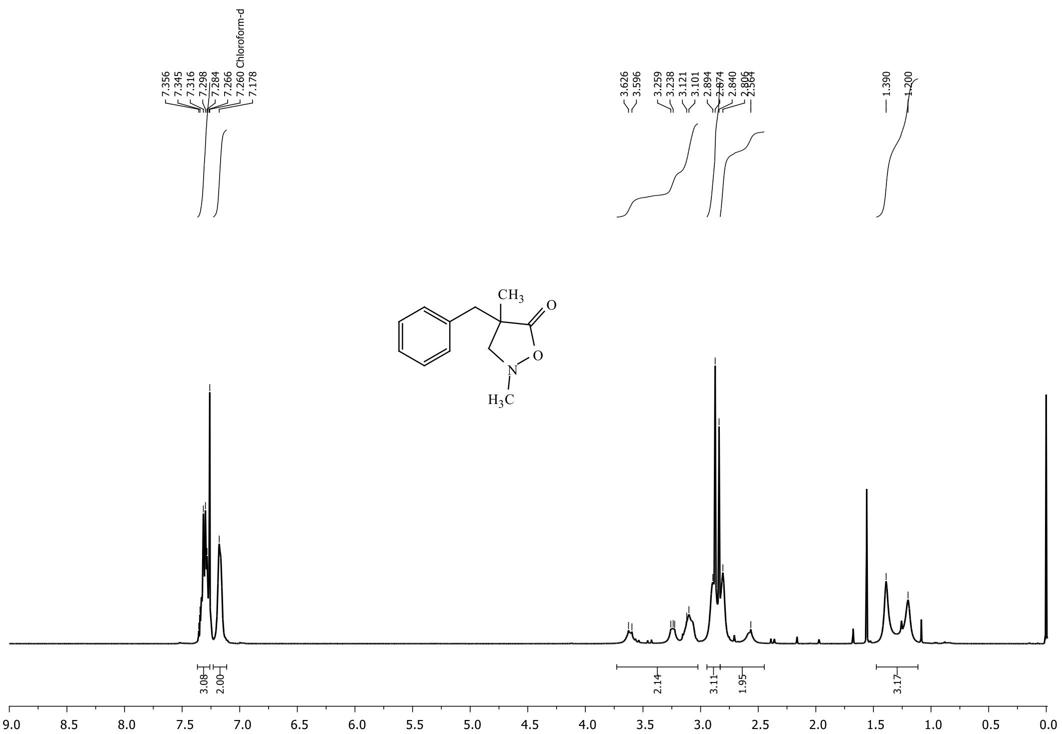

^{13}C NMR (101 MHz, $CDCl_3$)

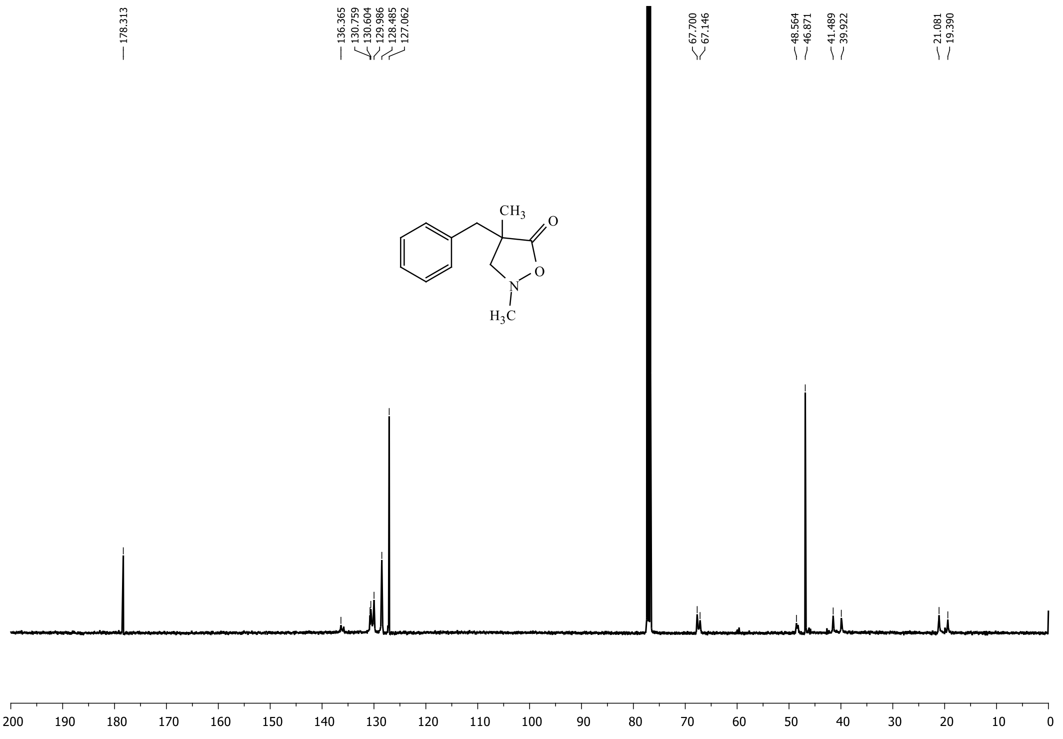

tert-Butyl4-(4-((*tert*-butyldimethylsilyl)oxy)phenethyl)-4-methyl-5-oxoisoxazolidine-2-carboxylate (3u)

^{1}H NMR (400 MHz, $CDCl_3$)

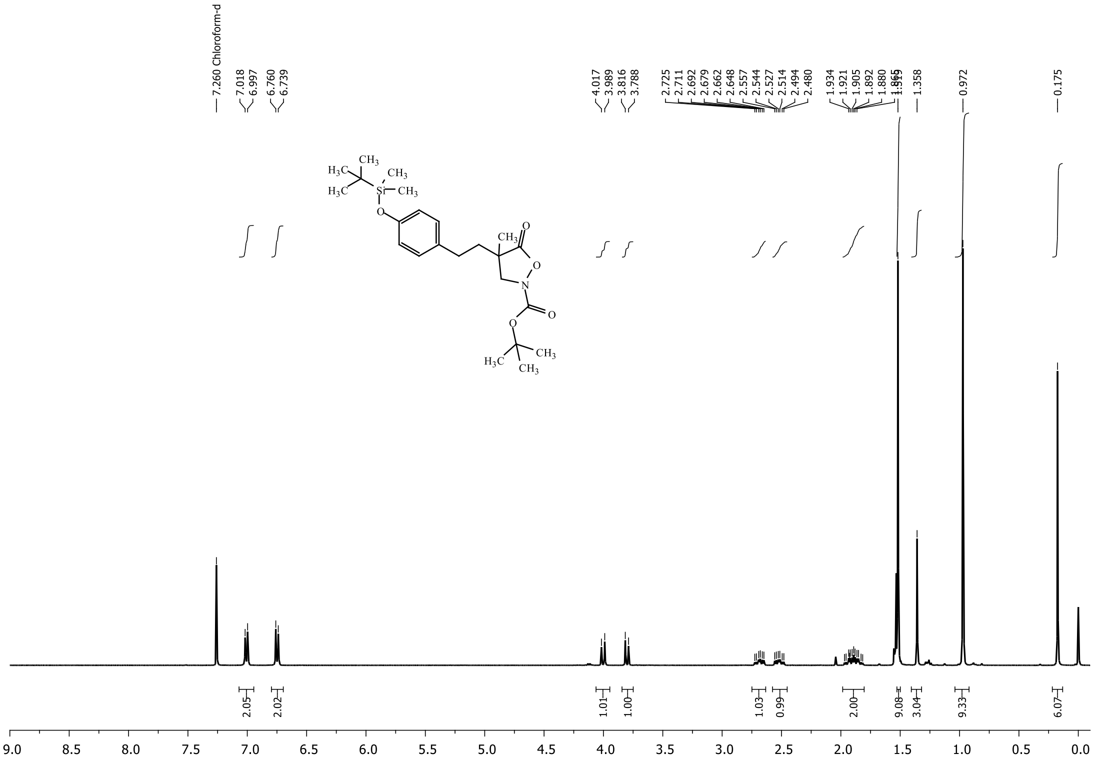

^{13}C NMR (101 MHz, $CDCl_3$)

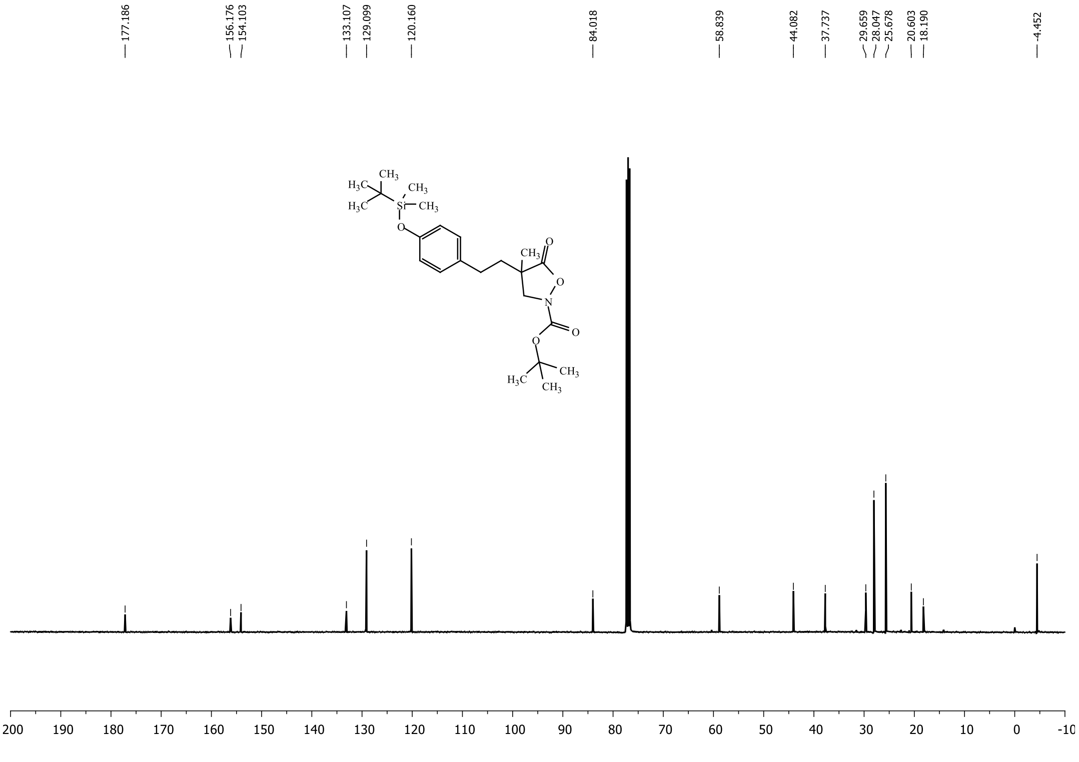

***tert*-Butyl 5-oxo-3-phenylisoxazolidine-2-carboxylate (S3)**

^{1}H NMR (400 MHz, $CDCl_3$)

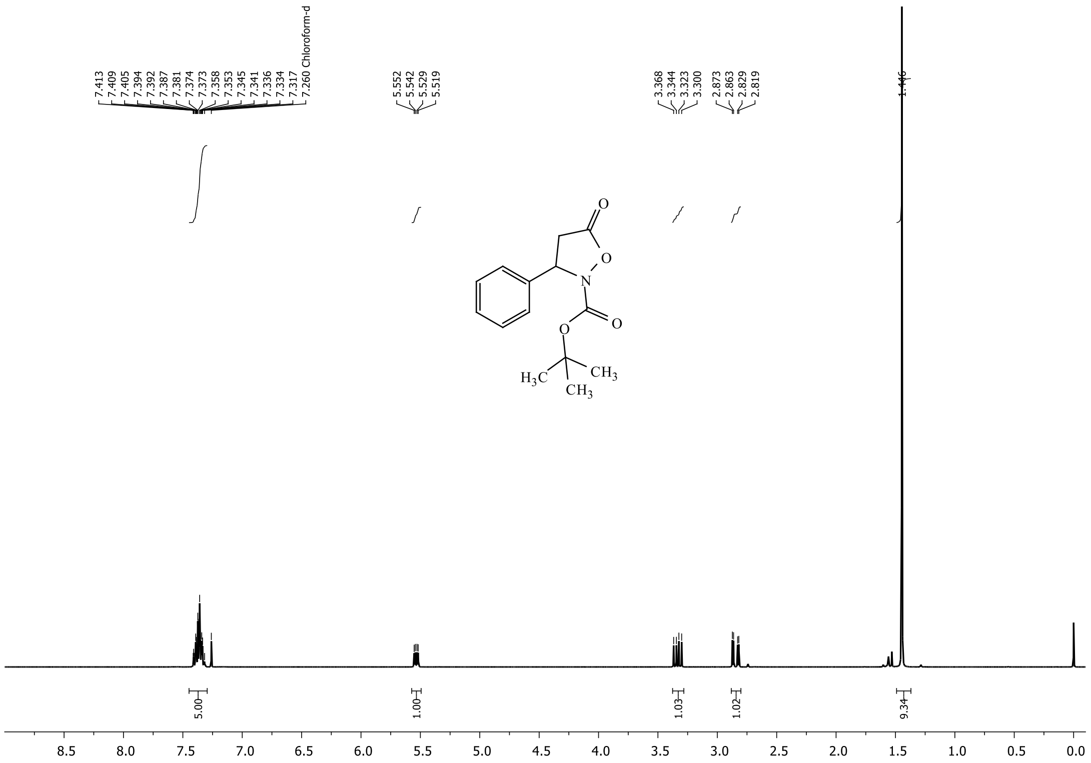

^{13}C NMR (101 MHz, $CDCl_3$)

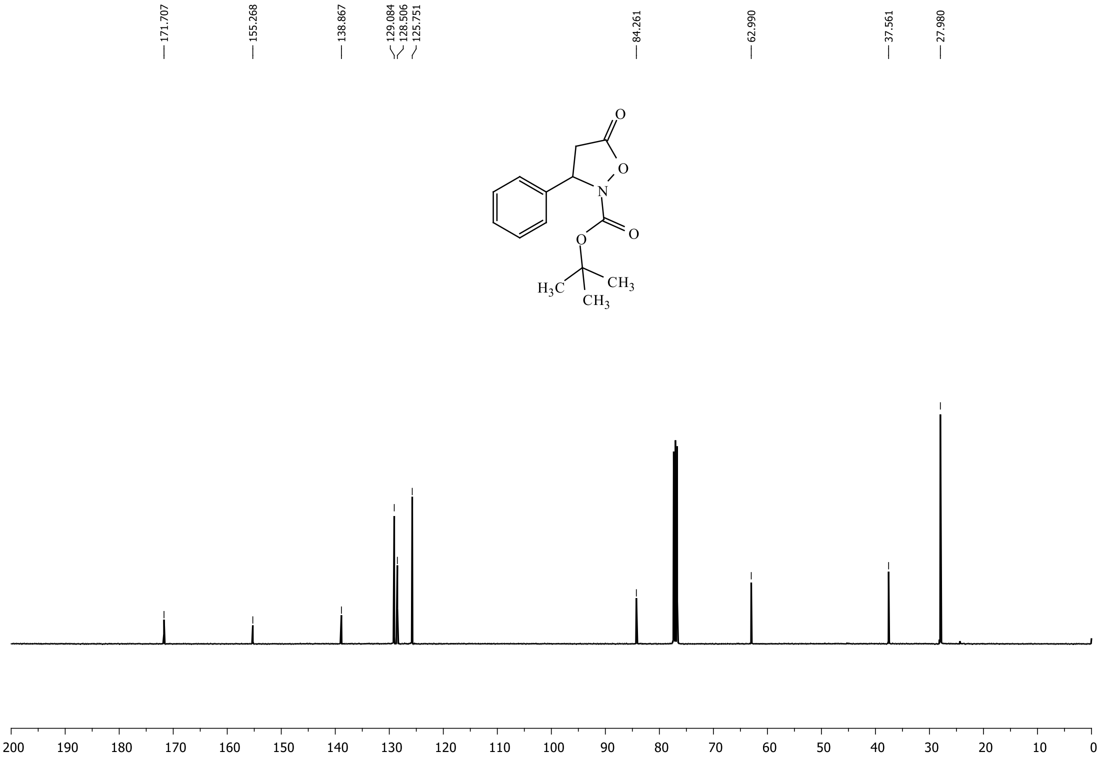

***tert*-Butyl 4,4-dibenzyl-5-oxo-3-phenylisoxazolidine-2-carboxylate (S4)**

^{1}H NMR (400 MHz, $CDCl_3$)

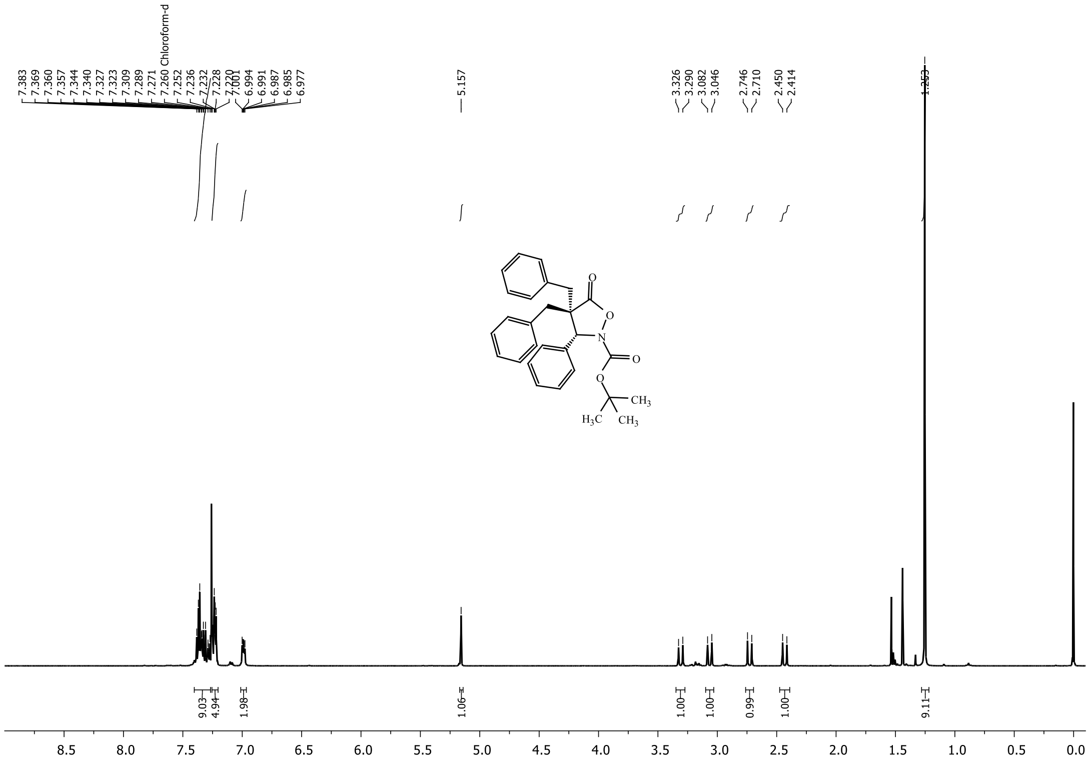

^{13}C NMR (101 MHz, $CDCl_3$)

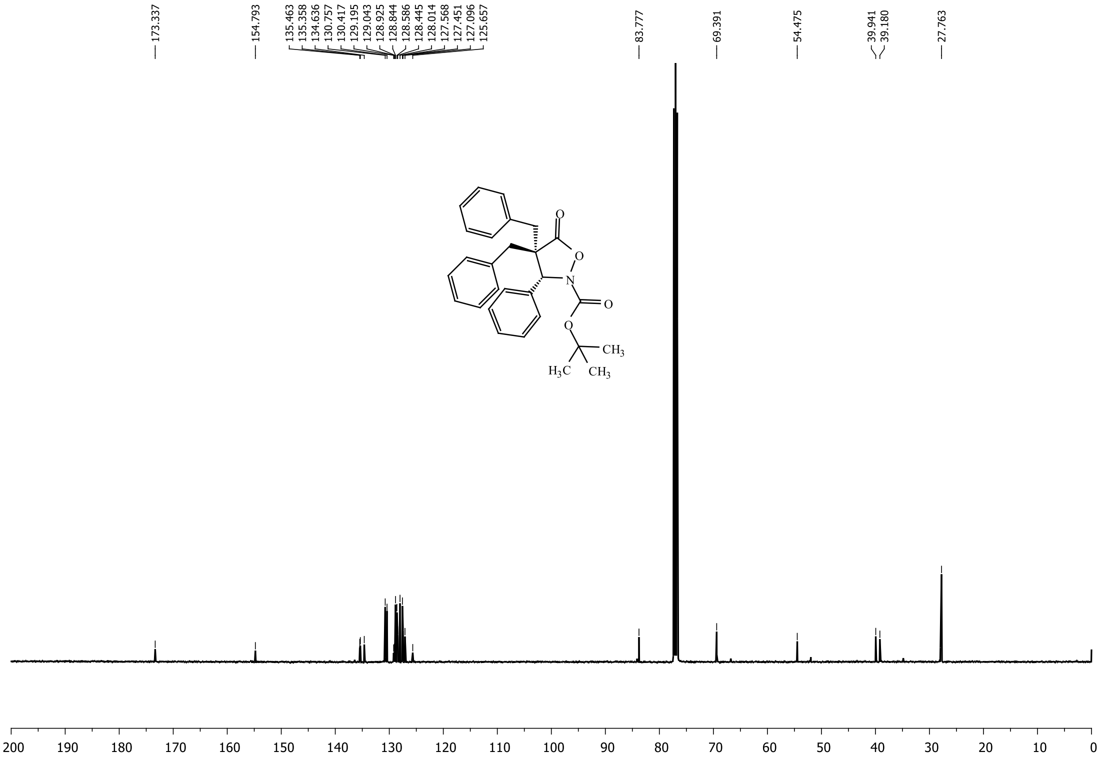

4-Benzyl-4-methylisoxazolidin-5-one (1a)

^{1}H NMR (400 MHz, CDCl₃)

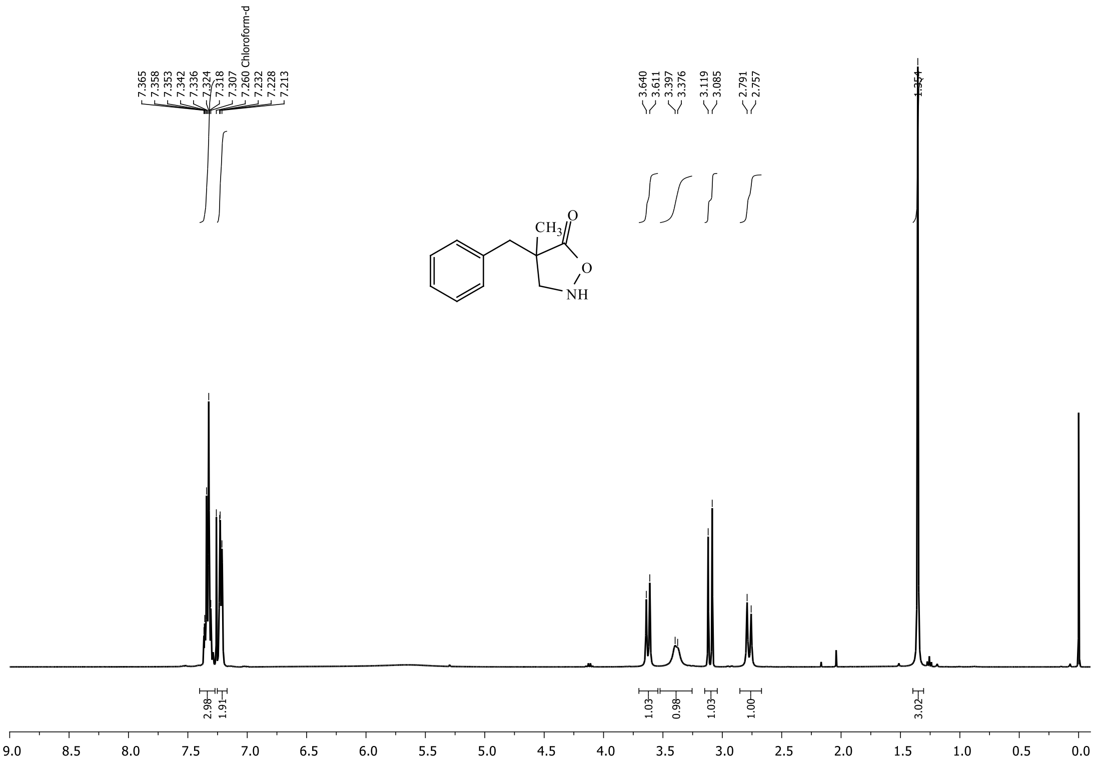

^{13}C NMR (101 MHz, CDCl₃)

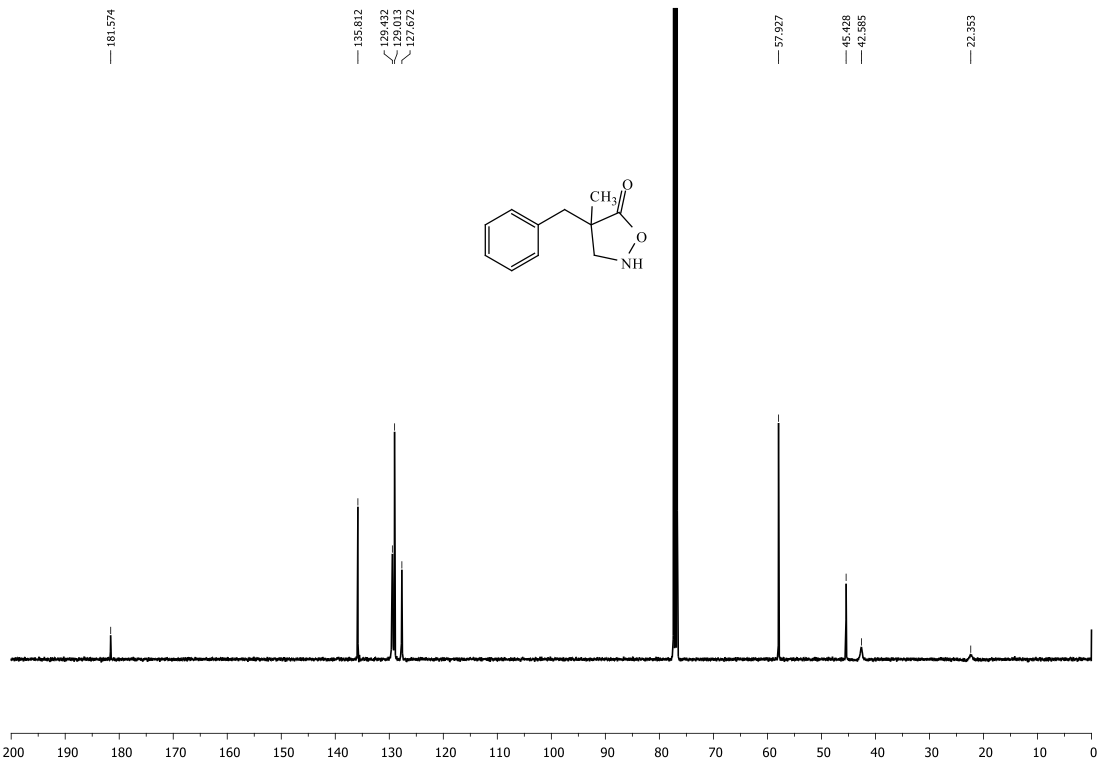

4-Benzyl-4-hexylisoxazolidin-5-one (1b)

^{1}H NMR (400 MHz, $CDCl_3$)

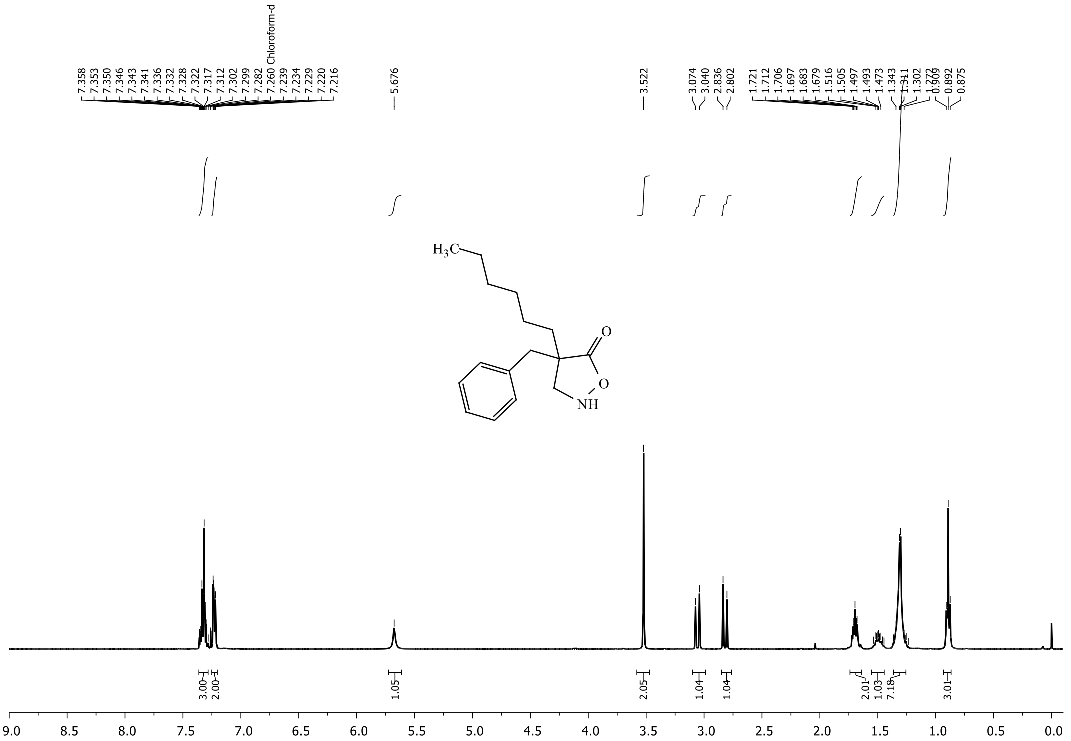

^{13}C NMR (101 MHz, $CDCl_3$)

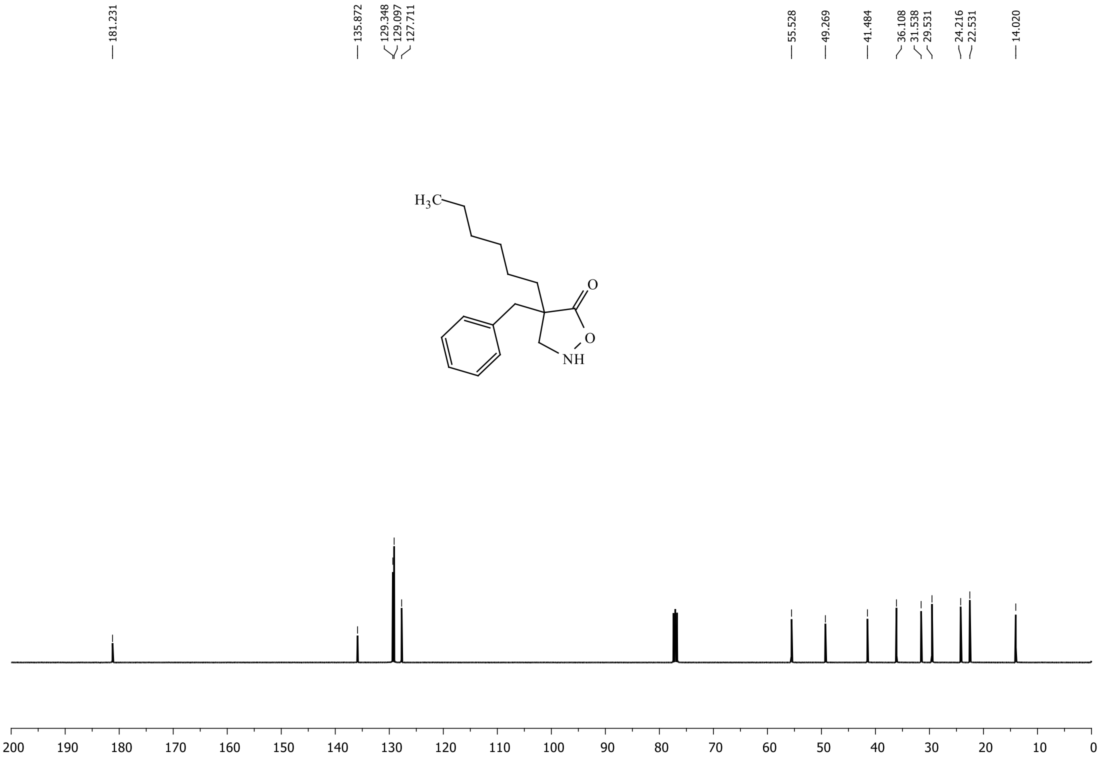

4-Benzyl-4-phenethylisoxazolidin-5-one (1c)

^{1}H NMR (400 MHz, CDCl$_3$)

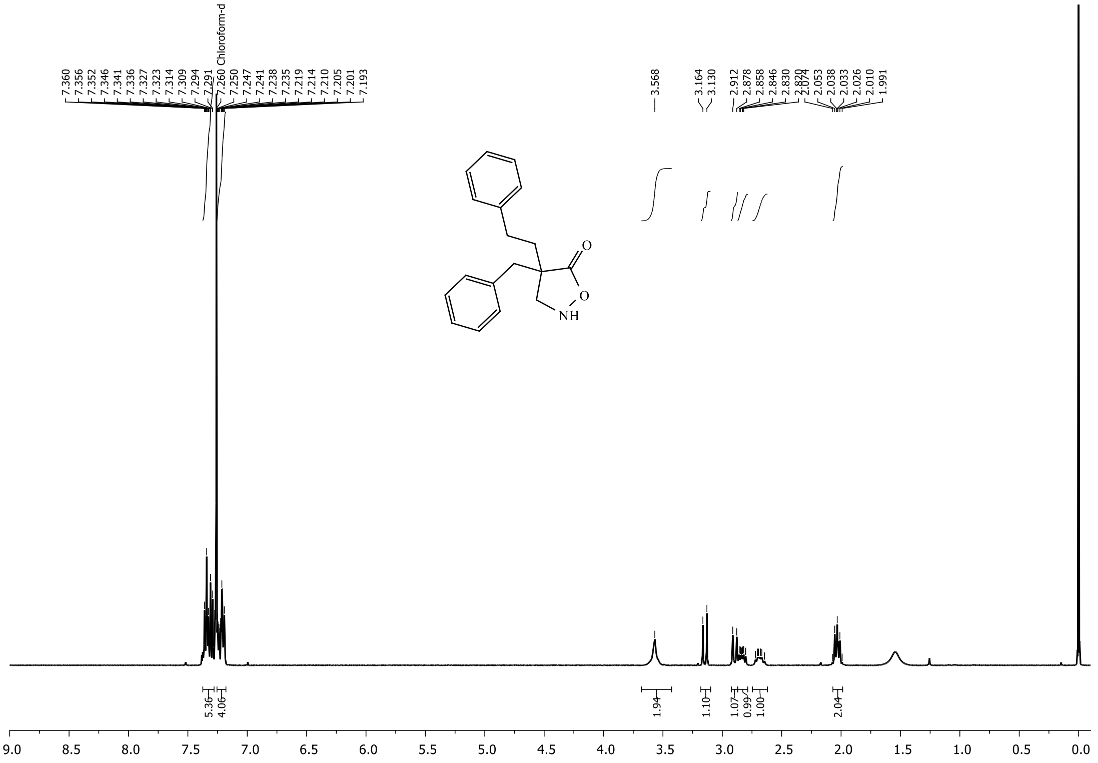

^{13}C NMR (101 MHz, CDCl$_3$)

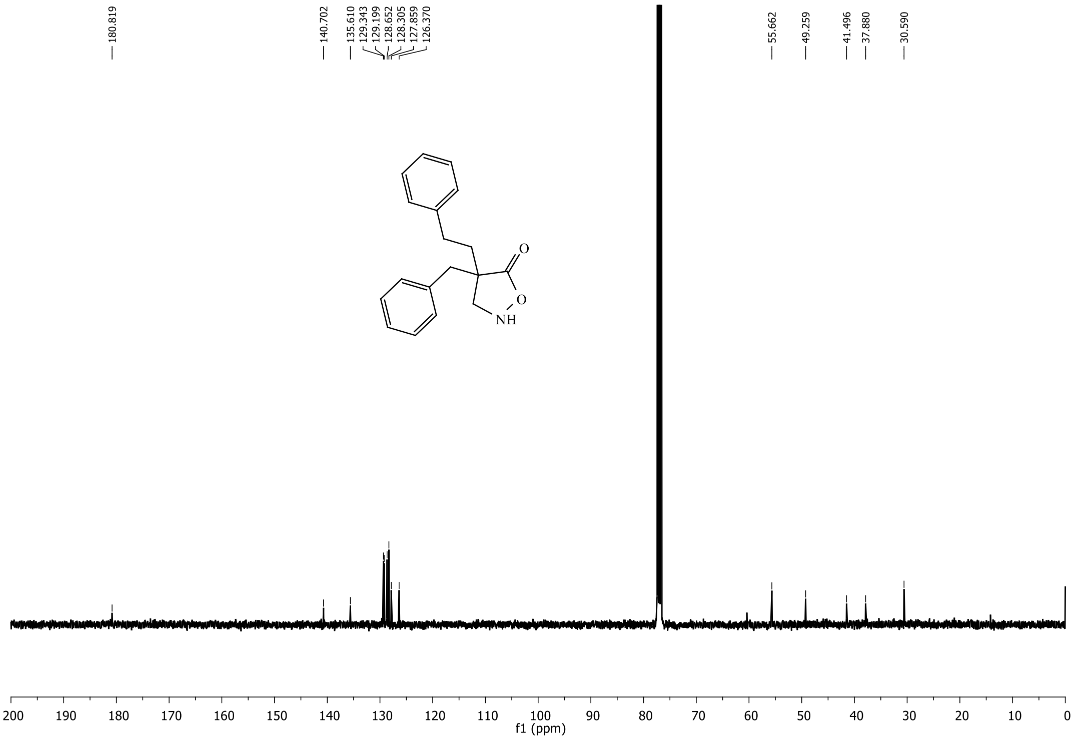

4-Benzyl-4-(pent-4-en-1-yl)isoxazolidin-5-one (1g)

^{1}H NMR (400 MHz, $CDCl_3$)

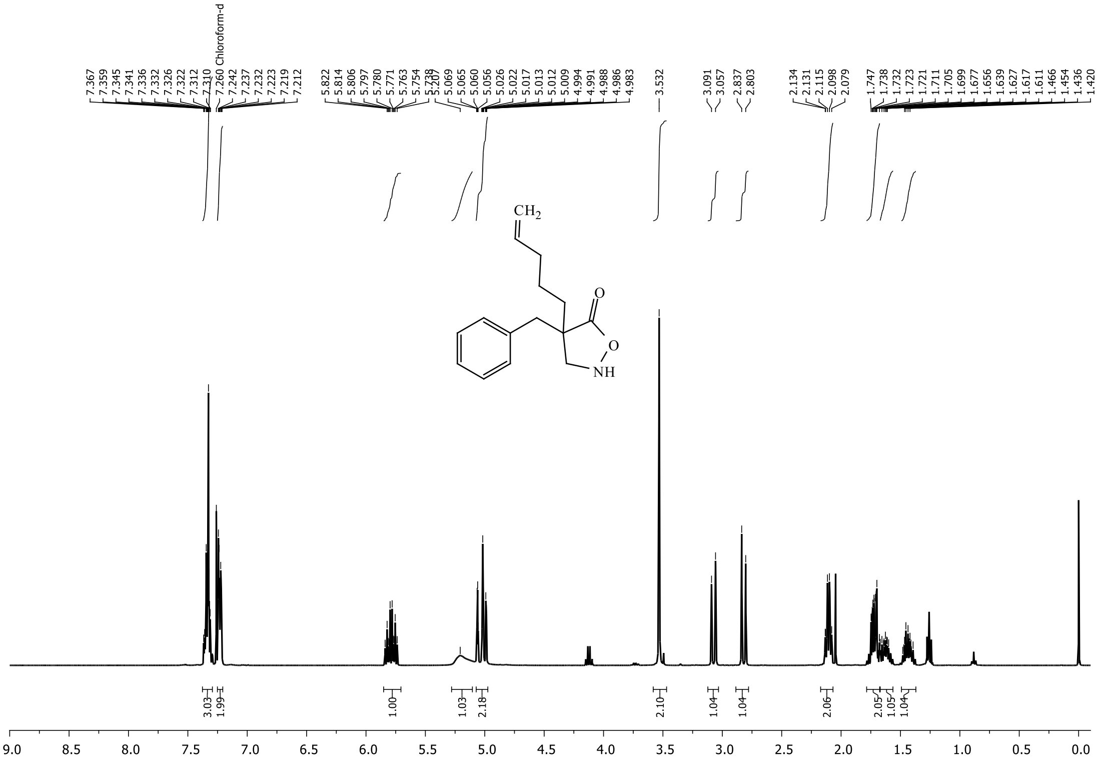

^{13}C NMR (101 MHz, $CDCl_3$)

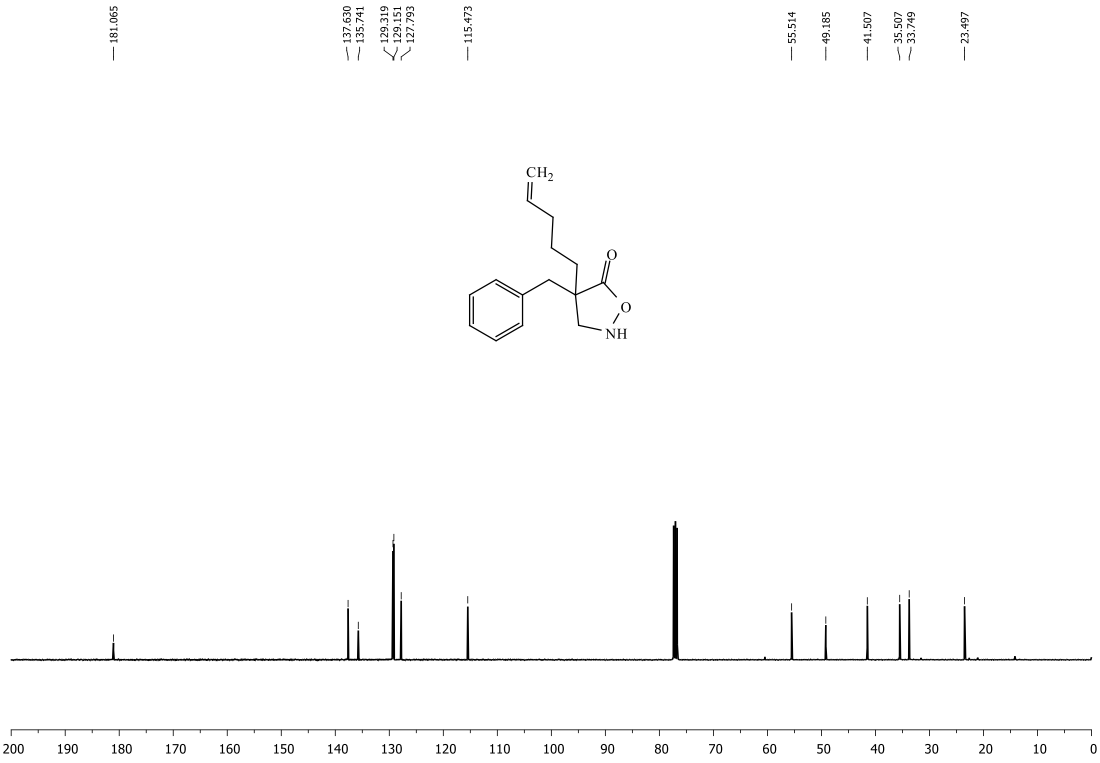

2-(4-Benzyl-5-oxoisoxazolidin-4-yl)acetonitrile (1h)

^{1}H NMR (400 MHz, $CDCl_3$)

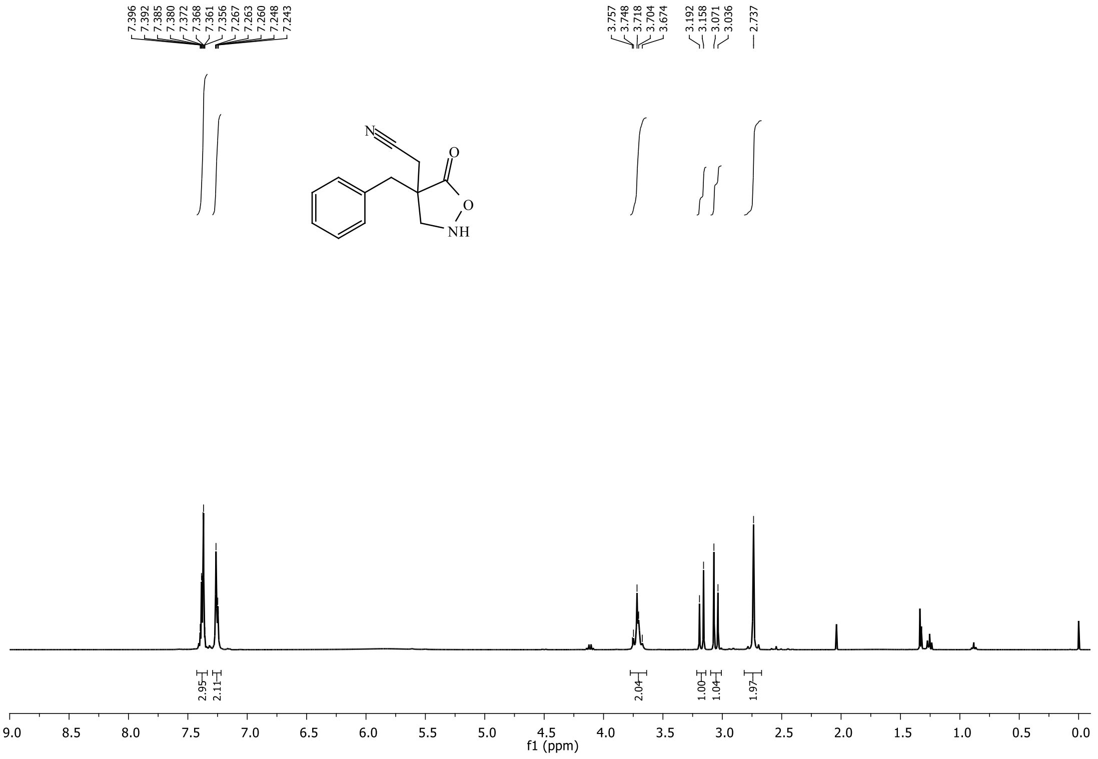

^{13}C NMR (101 MHz, $CDCl_3$)

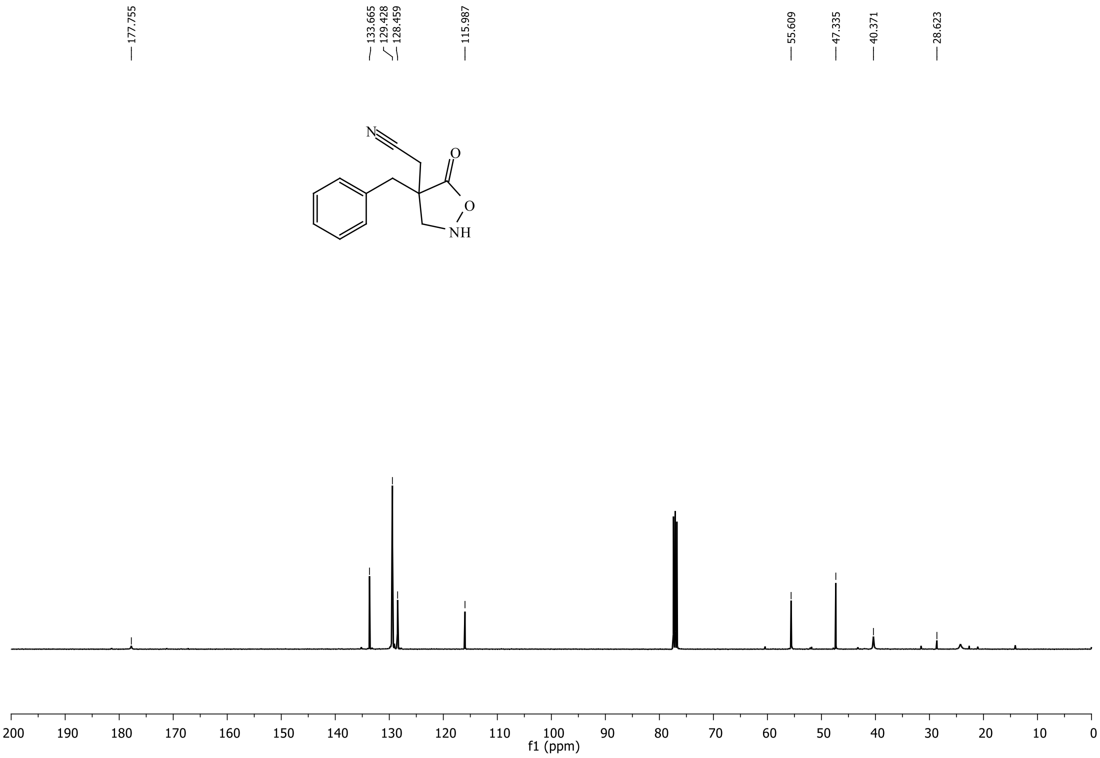

4-Benzyl-4-(prop-2-yn-1-yl)isoxazolidin-5-one (1i)

^{1}H NMR (400 MHz, $CDCl_3$)

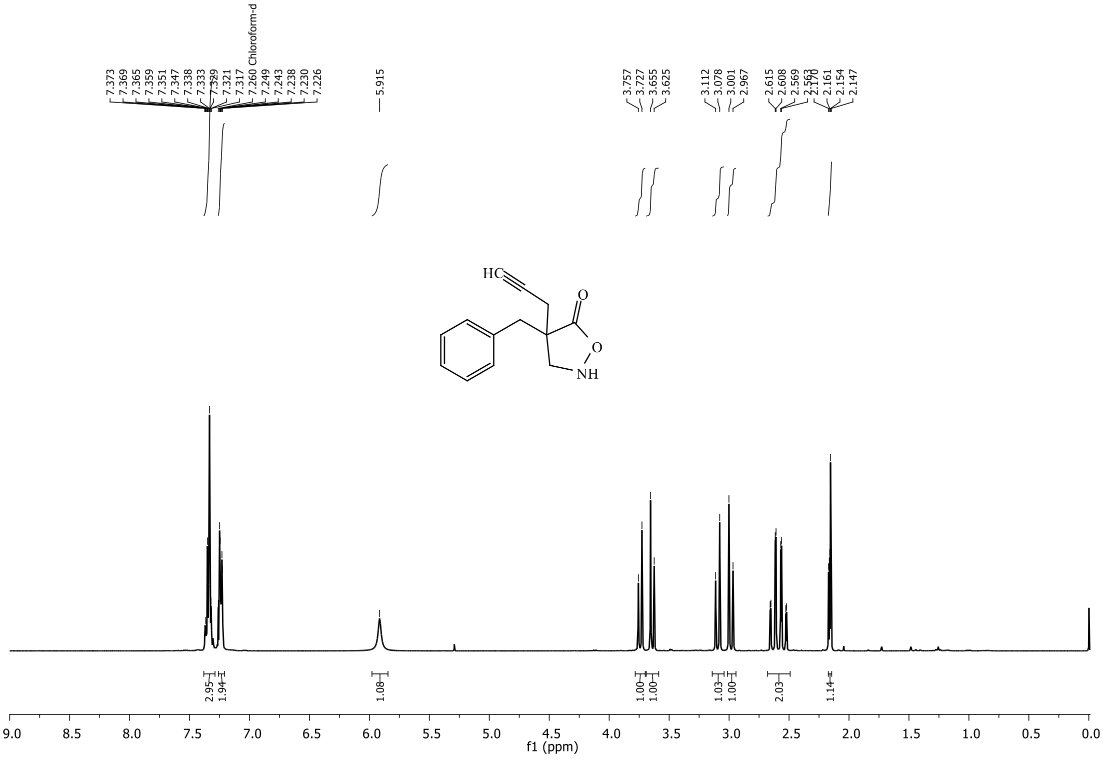

^{13}C NMR (101 MHz, $CDCl_3$)

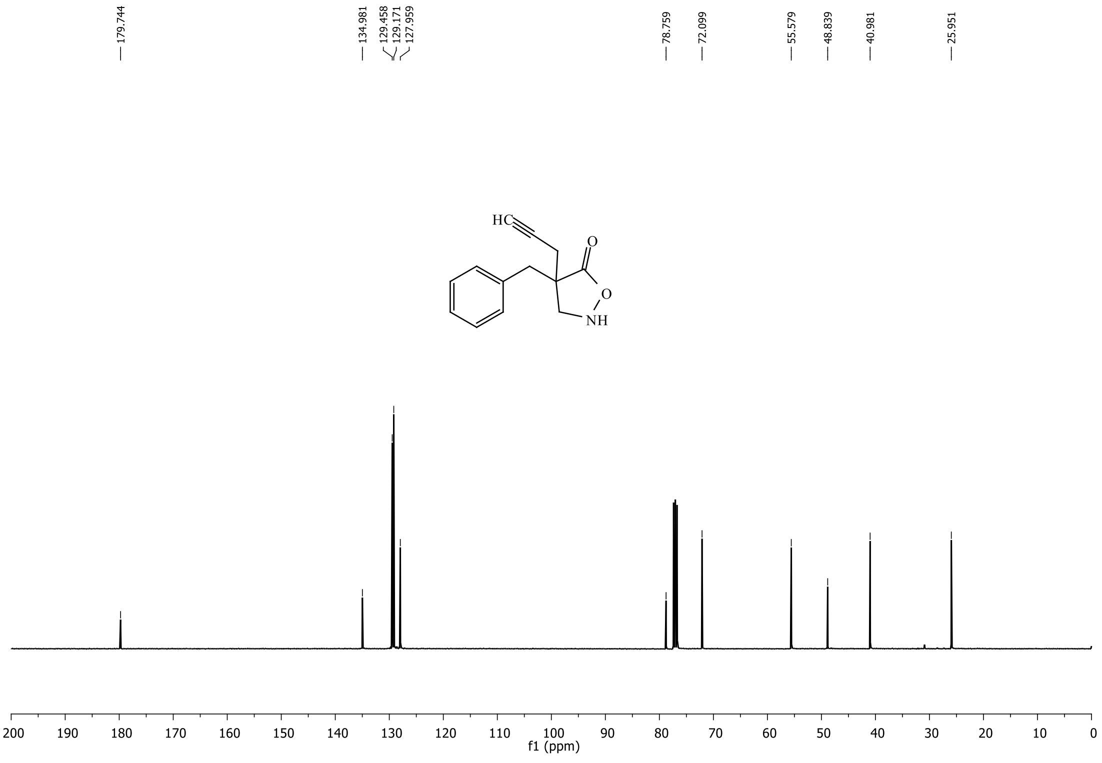

4-Methyl-4-(4-methylbenzyl)isoxazolidin-5-one (1j)

^{1}H NMR (400 MHz, $CDCl_3$)

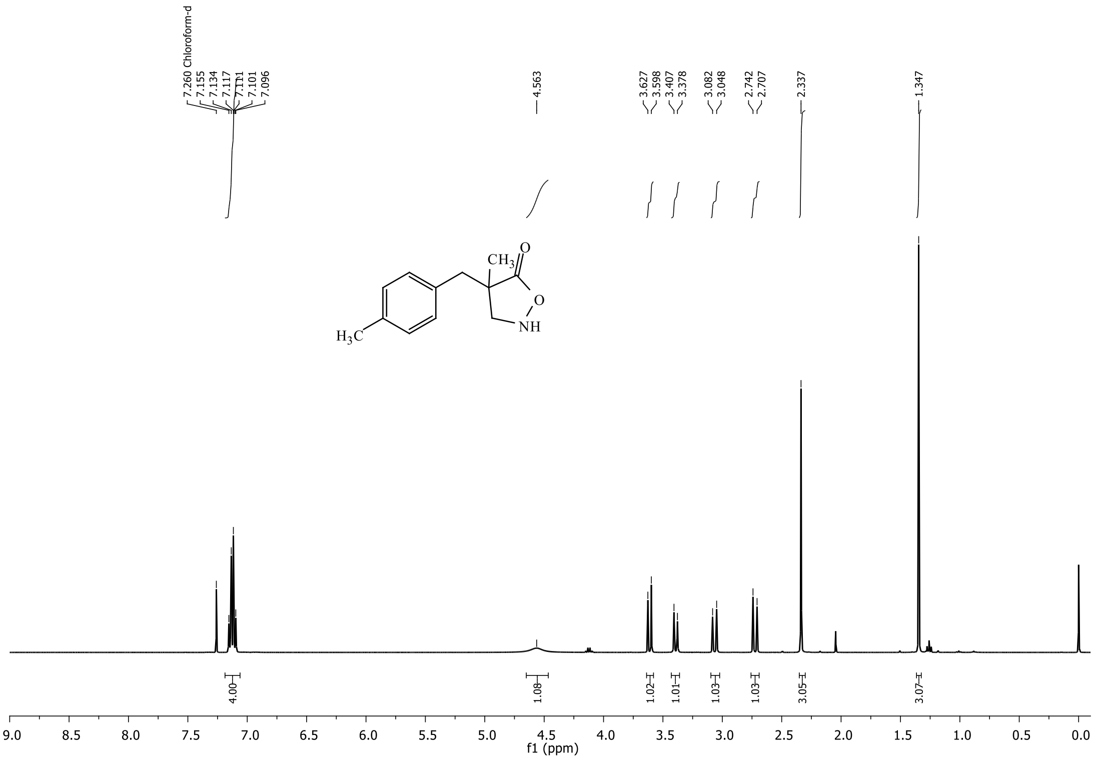

^{13}C NMR (101 MHz, $CDCl_3$)

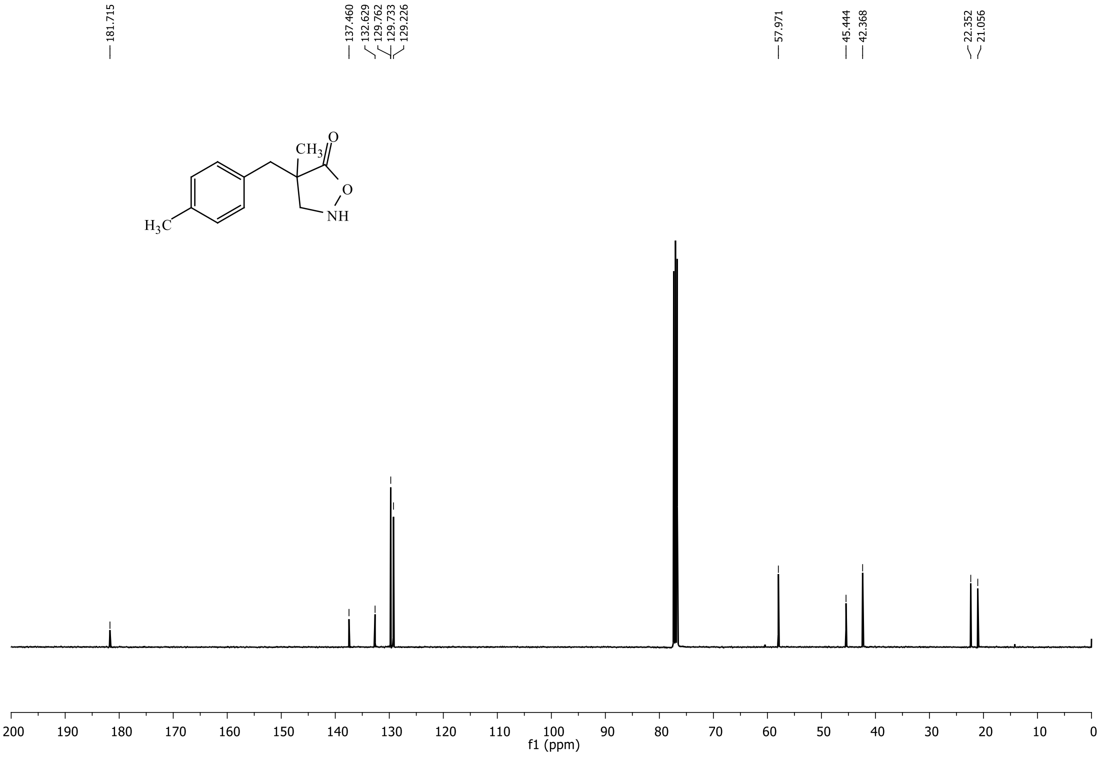

4-Methyl-4-(2-methylbenzyl)isoxazolidin-5-one (1k)

^{1}H NMR (400 MHz, $CDCl_3$)

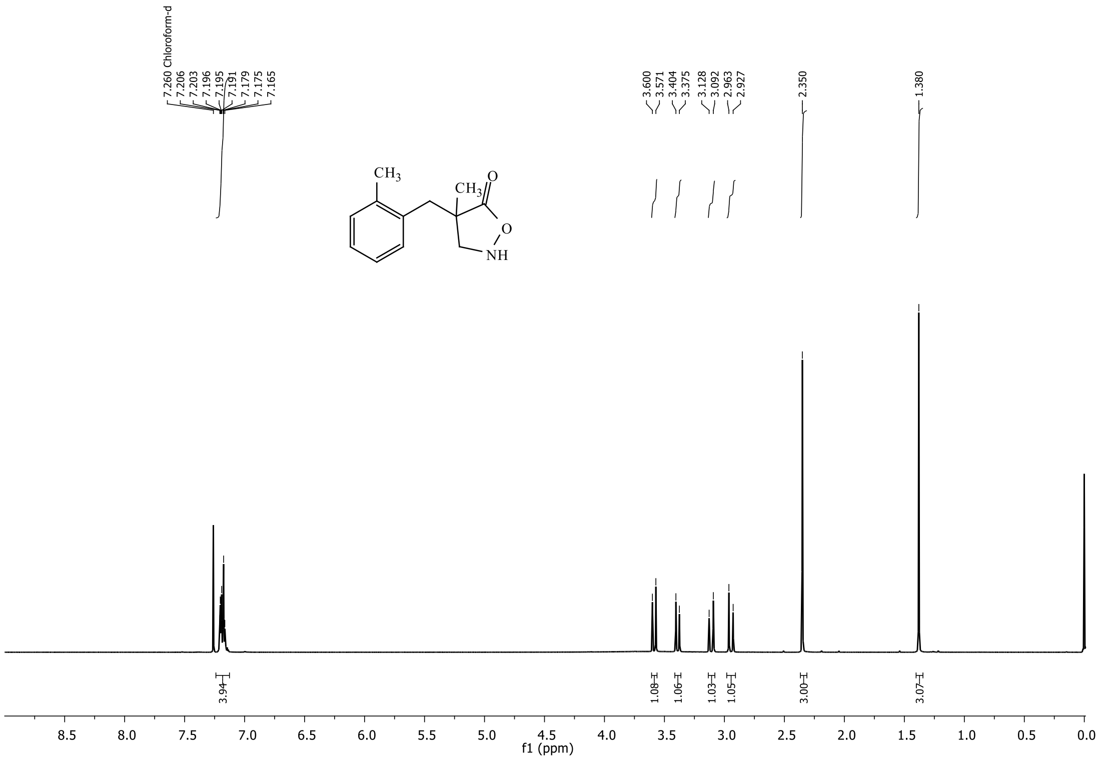

^{13}C NMR (101 MHz, $CDCl_3$)

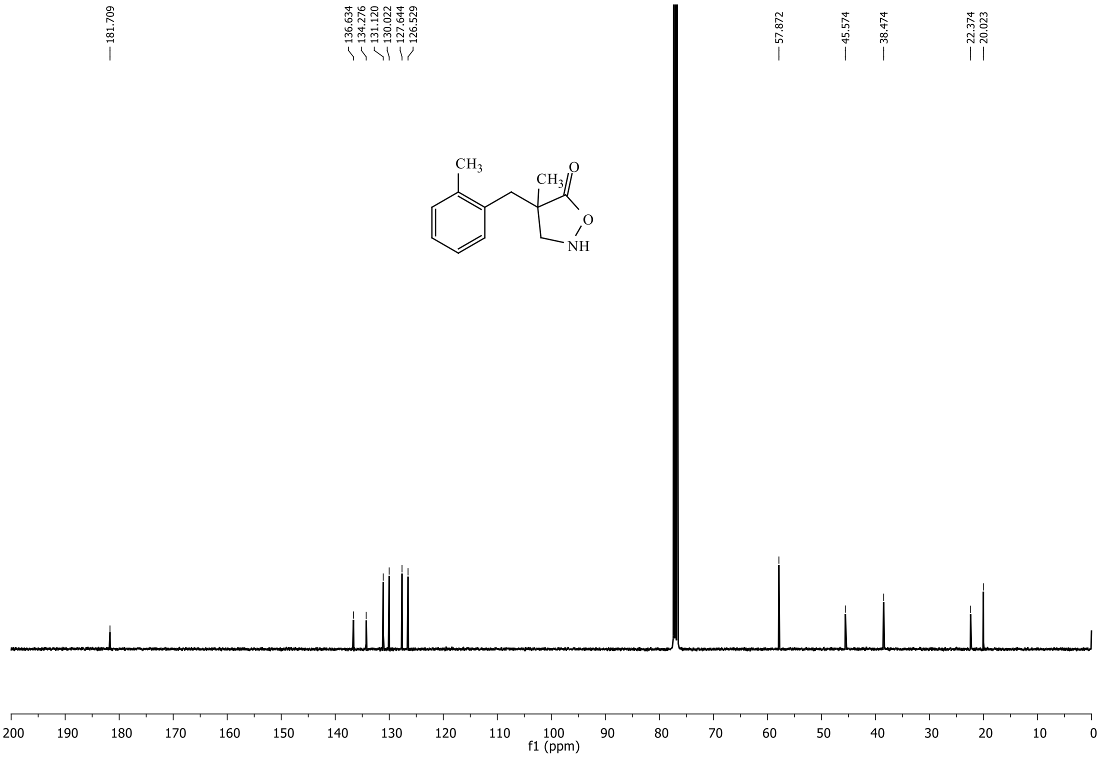

4-(4-Bromobenzyl)-4-methylisoxazolidin-5-one (1l)

^{1}H NMR (400 MHz, $CDCl_3$)

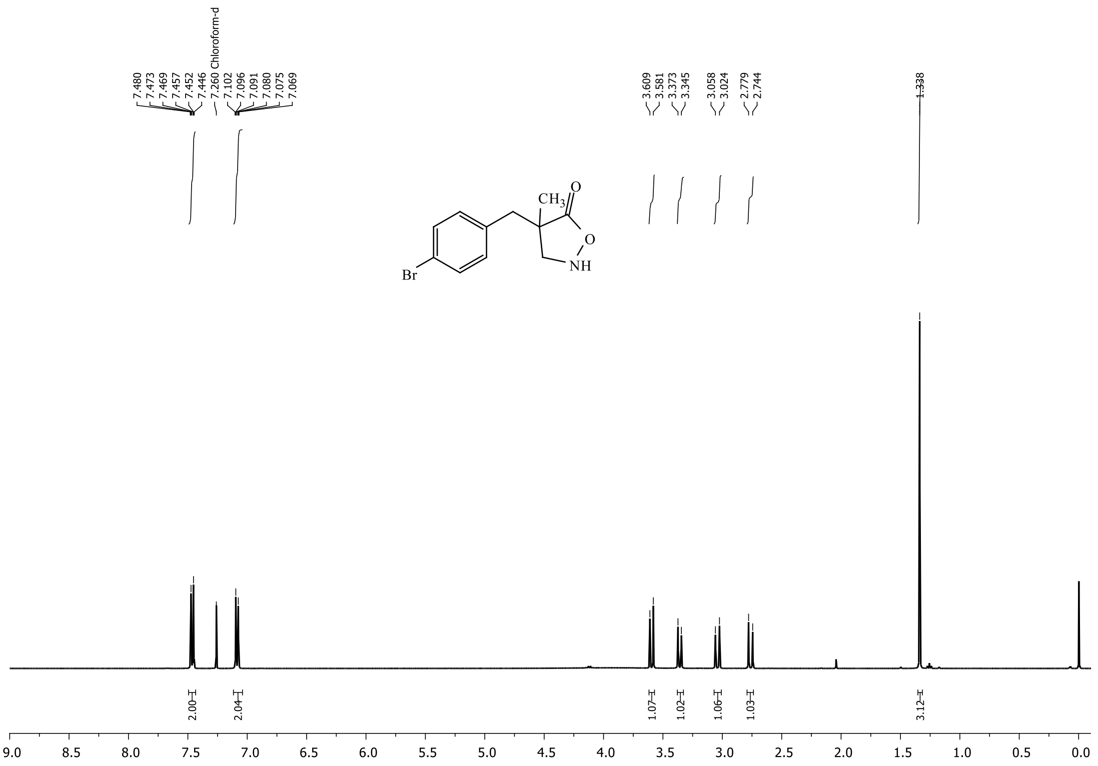

^{13}C NMR (101 MHz, $CDCl_3$)

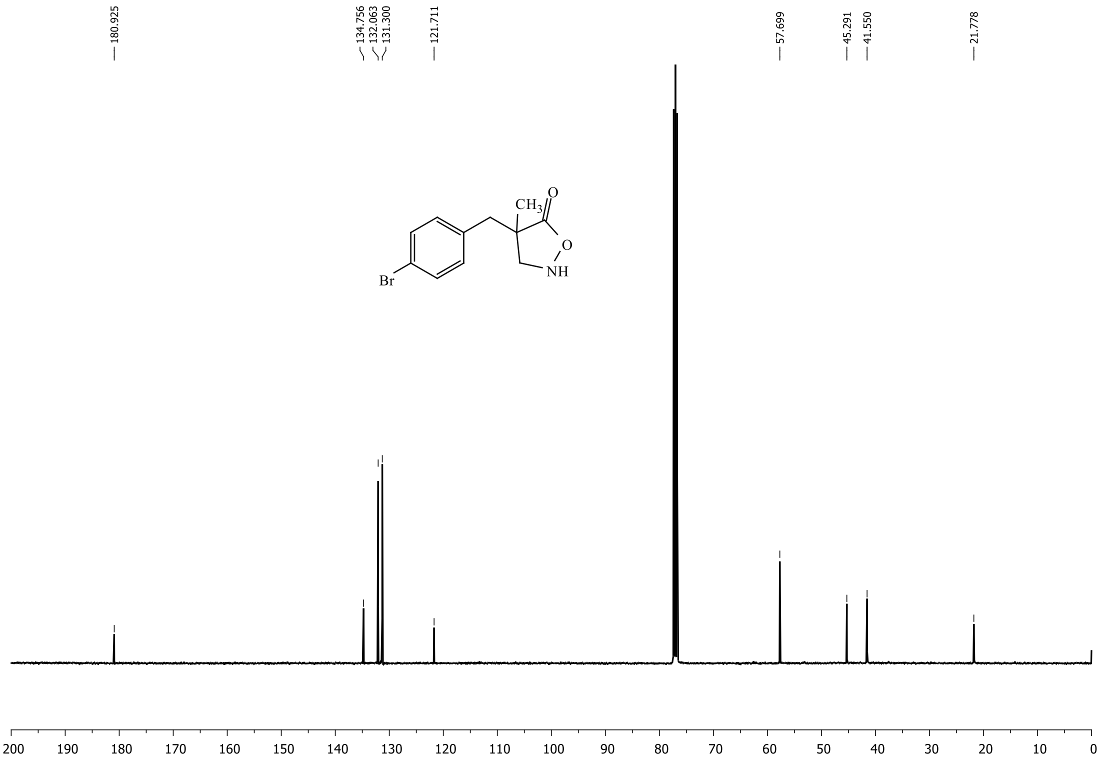

4-(2-Chlorobenzyl)-4-methylisoxazolidin-5-one (1m)

^{1}H NMR (400 MHz, $CDCl_3$)

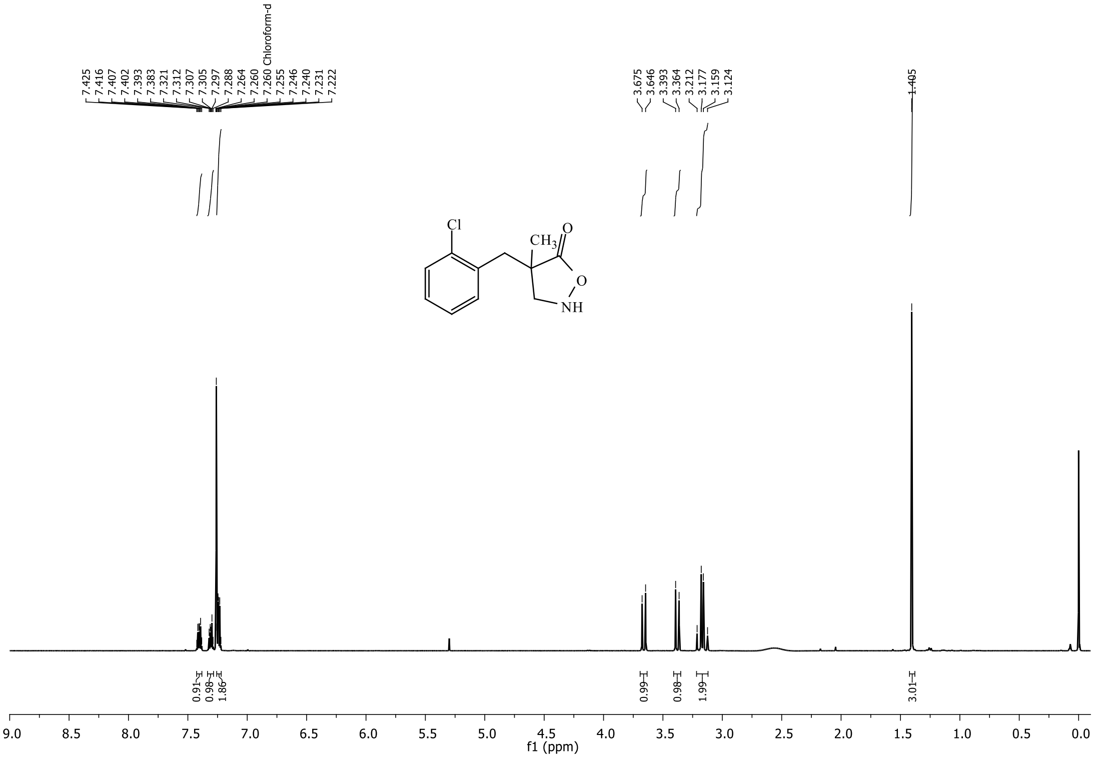

^{13}C NMR (101 MHz, $CDCl_3$)

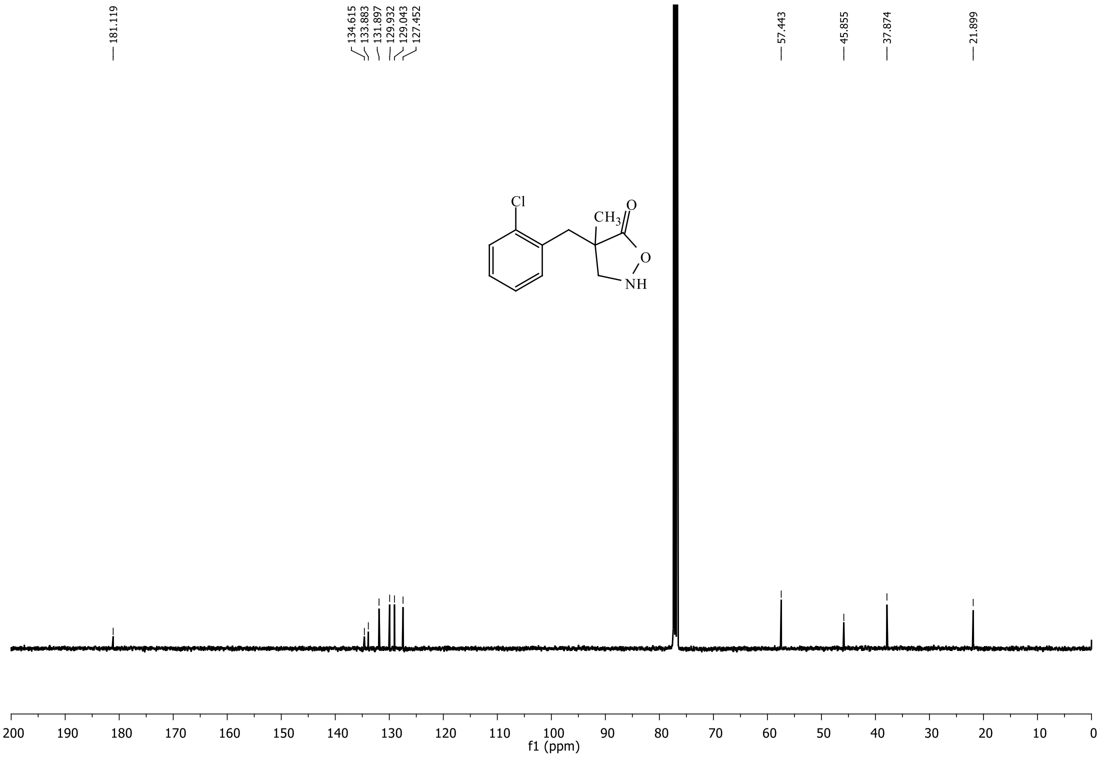

4-Methyl-4-(3-methylbenzyl)isoxazolidin-5-one (1n)

^{1}H NMR (400 MHz, CDCl$_3$)

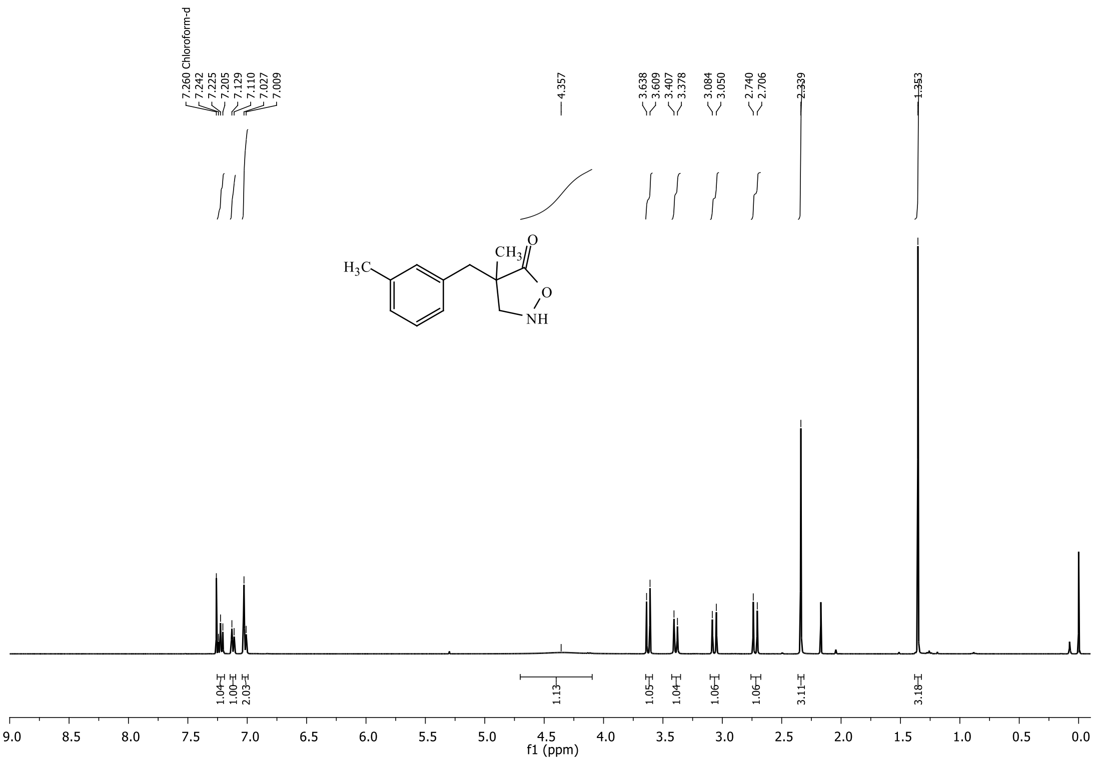

^{13}C NMR (101 MHz, CDCl$_3$)

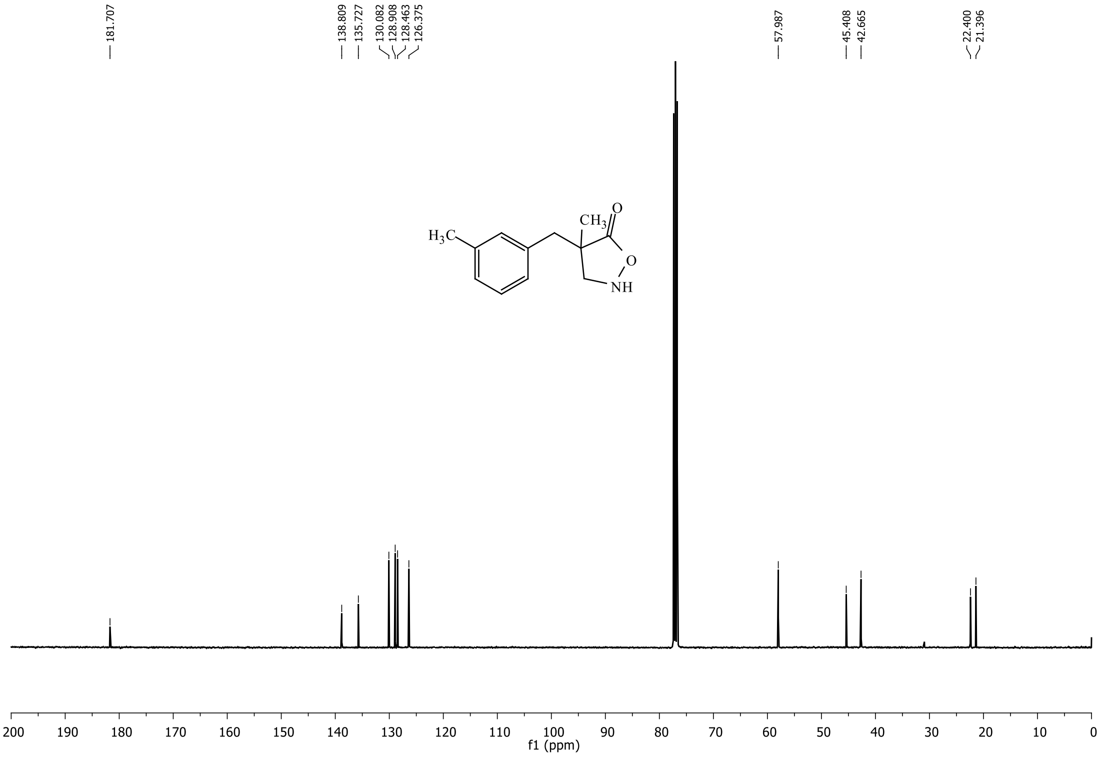

4-(3-Methoxybenzyl)-4-methylisoxazolidin-5-one (1o)

^{1}H NMR (400 MHz, $CDCl_3$)

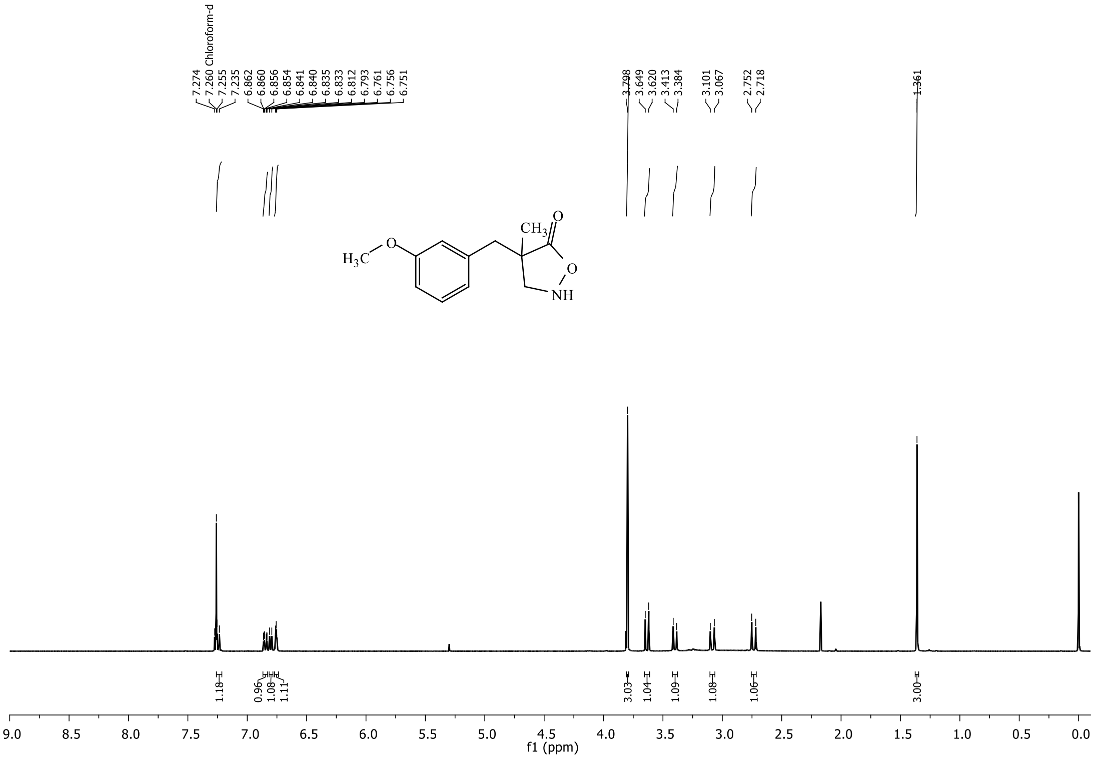

^{13}C NMR (101 MHz, $CDCl_3$)

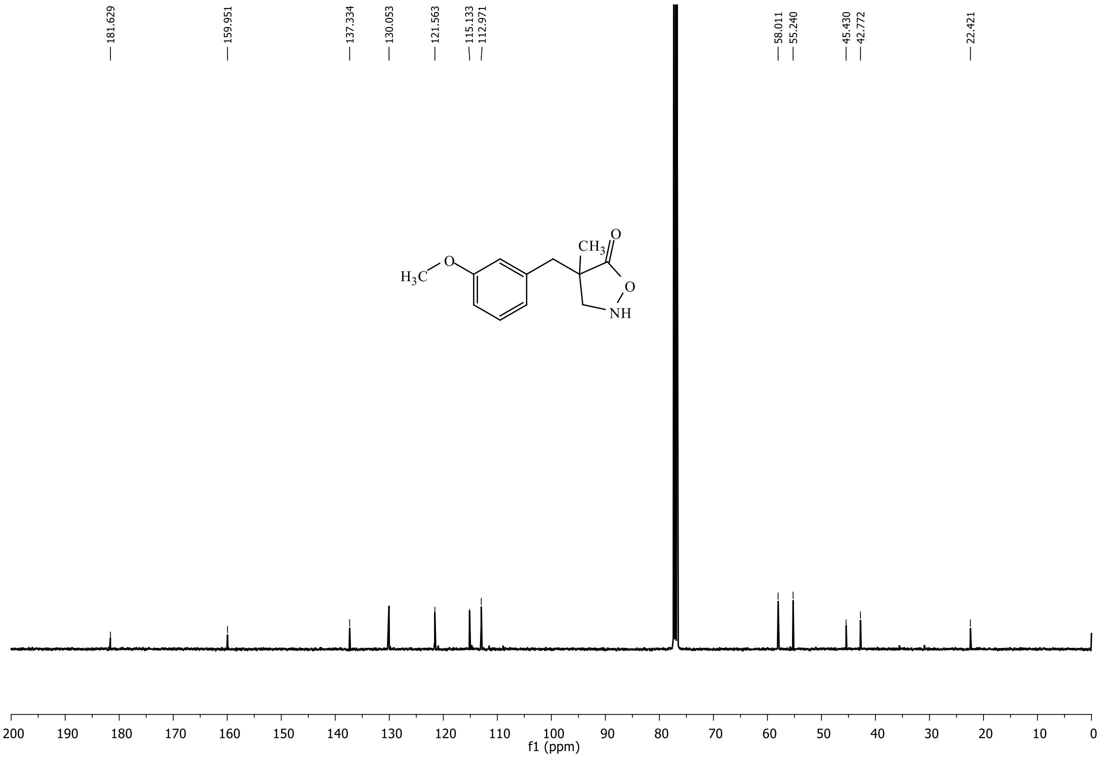

4-(3-Fluorobenzyl)-4-methylisoxazolidin-5-one (1p)

^{1}H NMR (400 MHz, $CDCl_3$)

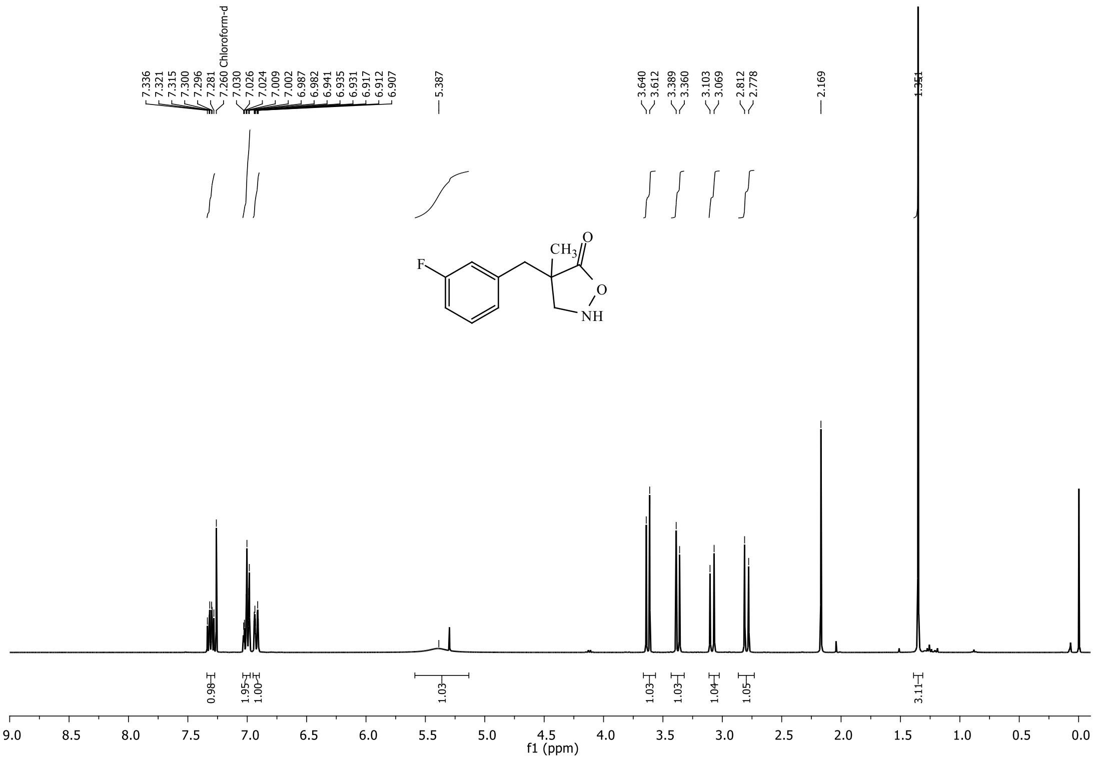

^{13}C NMR (101 MHz, $CDCl_3$)

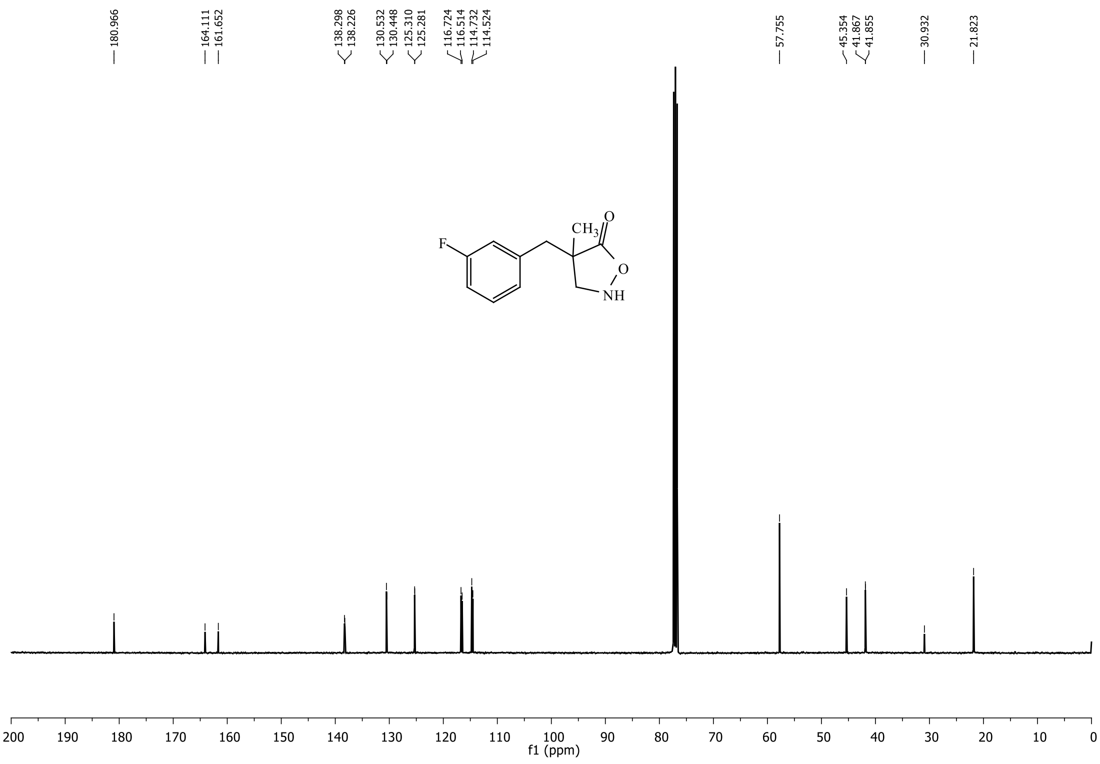

^{19}F NMR (376 MHz, CDCl$_3$)

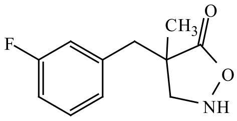

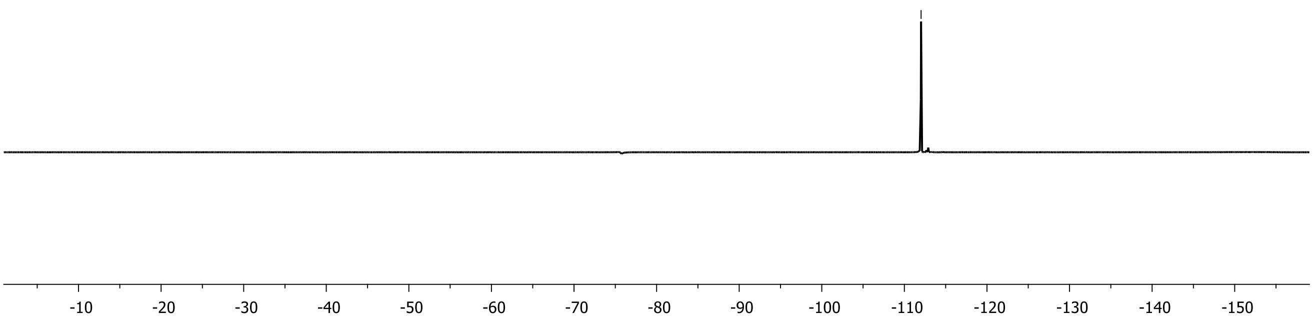

4-Methyl-4-(4-(4,4,5,5-tetramethyl-1,3-dioxolan-2-yl)benzyl)isoxazolidin-5-one (1q)

^{1}H NMR (400 MHz, CDCl$_3$)

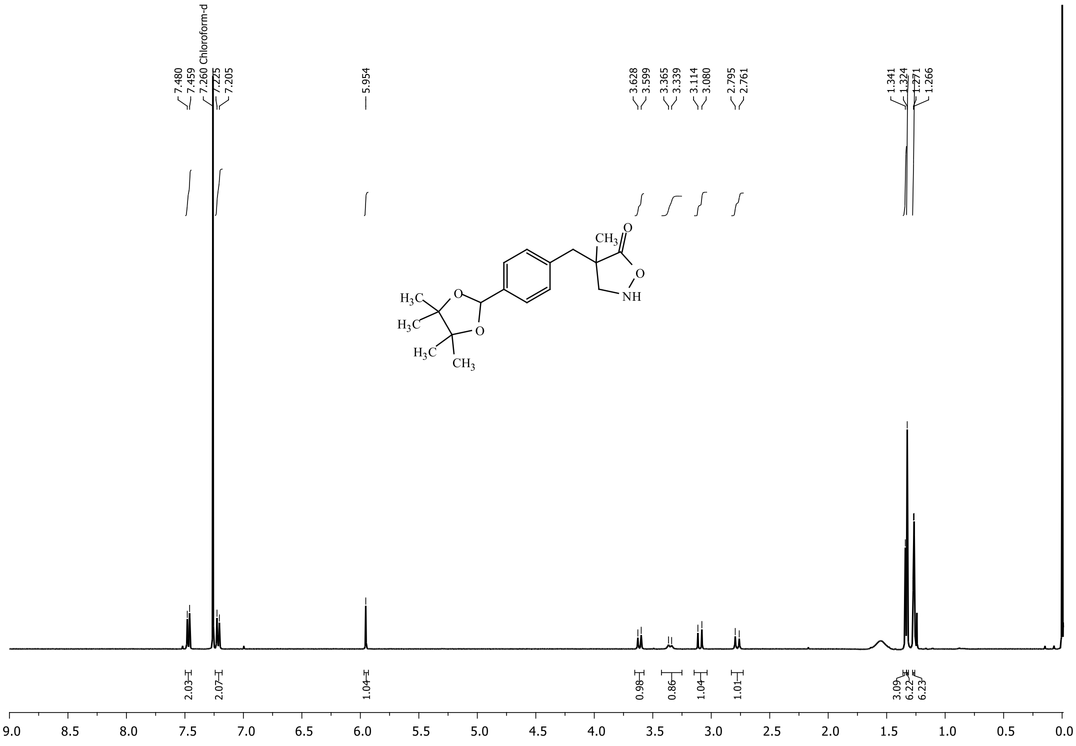

^{13}C NMR (101 MHz, CDCl$_3$)

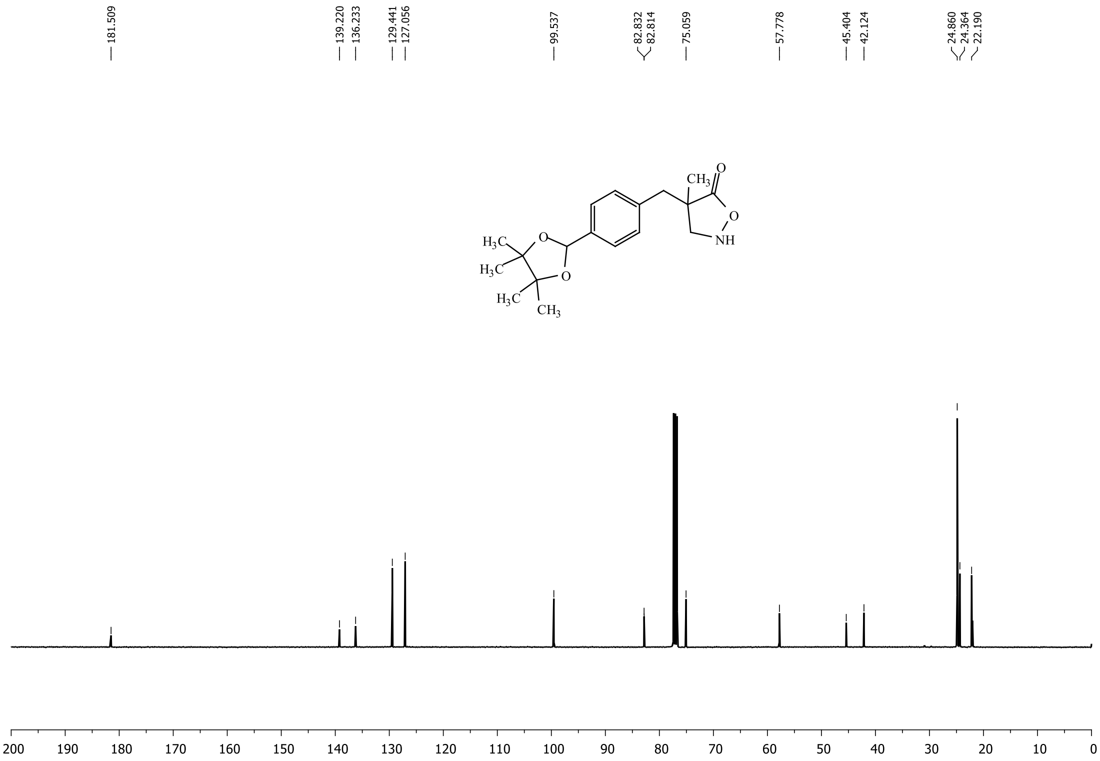

4-Methyl-4-(4-((triisopropylsilyl)ethynyl)benzyl)isoxazolidin-5-one (1r)

^{1}H NMR (400 MHz, $CDCl_3$)

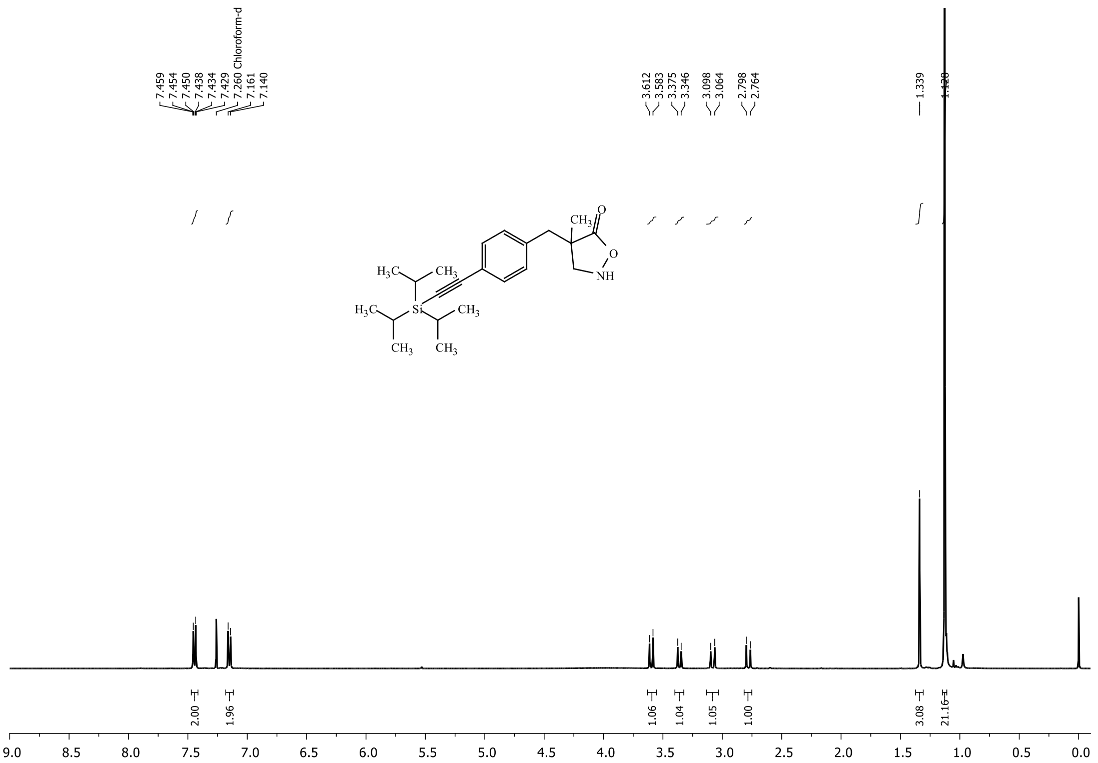

^{13}C NMR (101 MHz, $CDCl_3$)

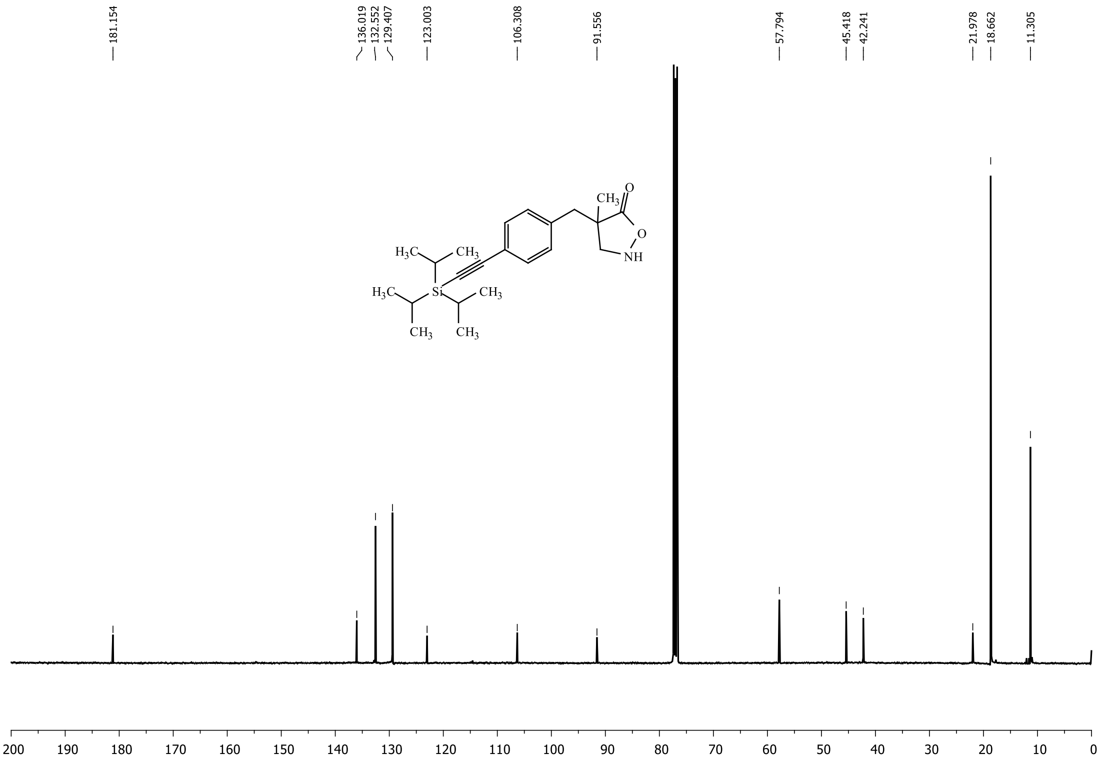

4-(4-(*tert*-Butoxy)benzyl)-4-methylisoxazolidin-5-one (15)

^{1}H NMR (400 MHz, CD$_3$OD)

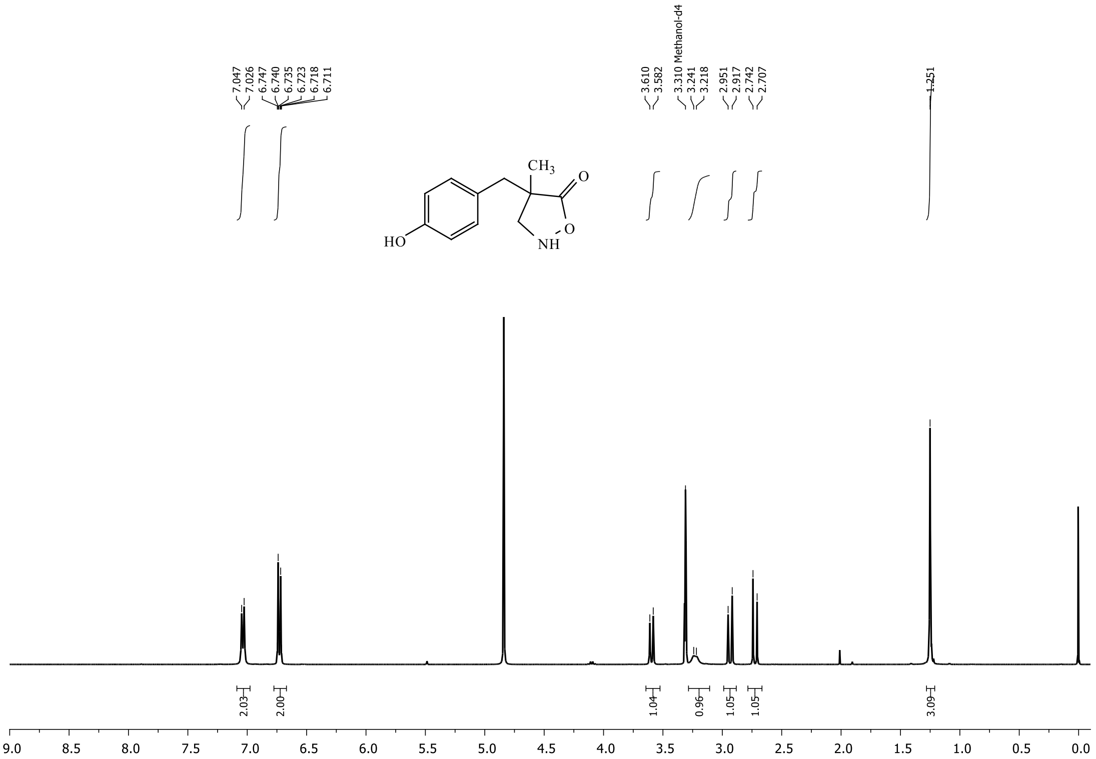

^{13}C NMR (101 MHz, CD$_3$OD)

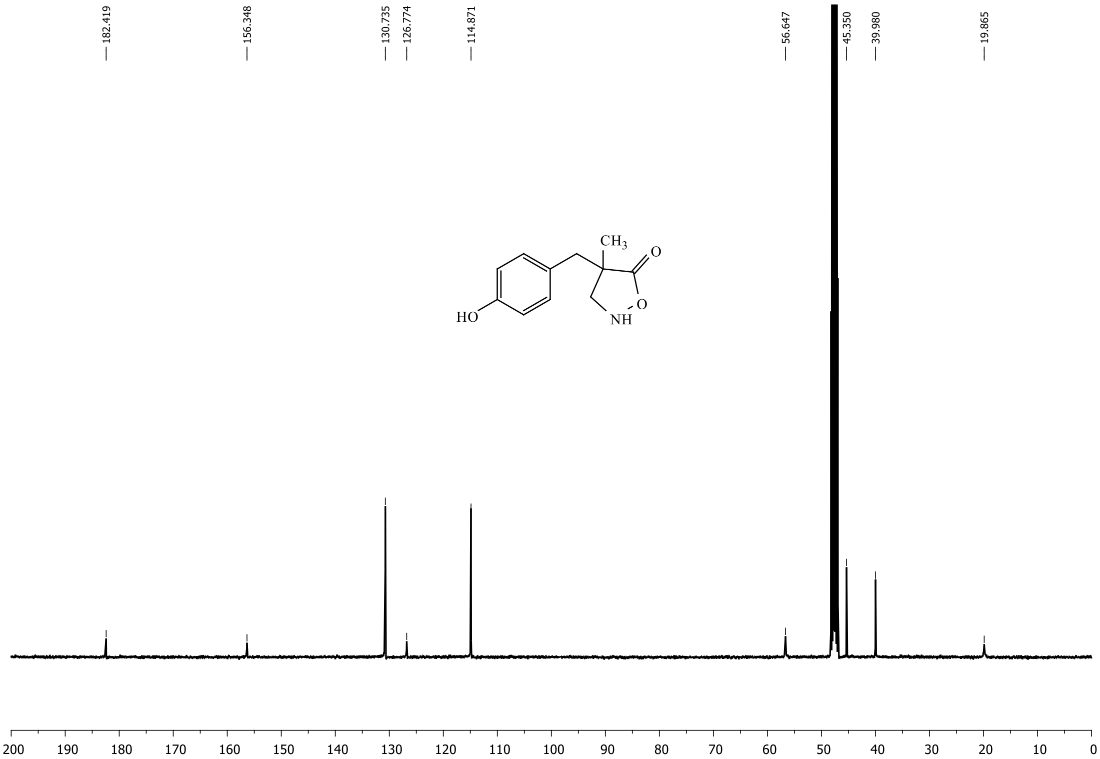

4-(4-Hydroxyphenethyl)-4-methylisoxazolidin-5-one (18)

^{1}H NMR (400 MHz, $CDCl_3$)

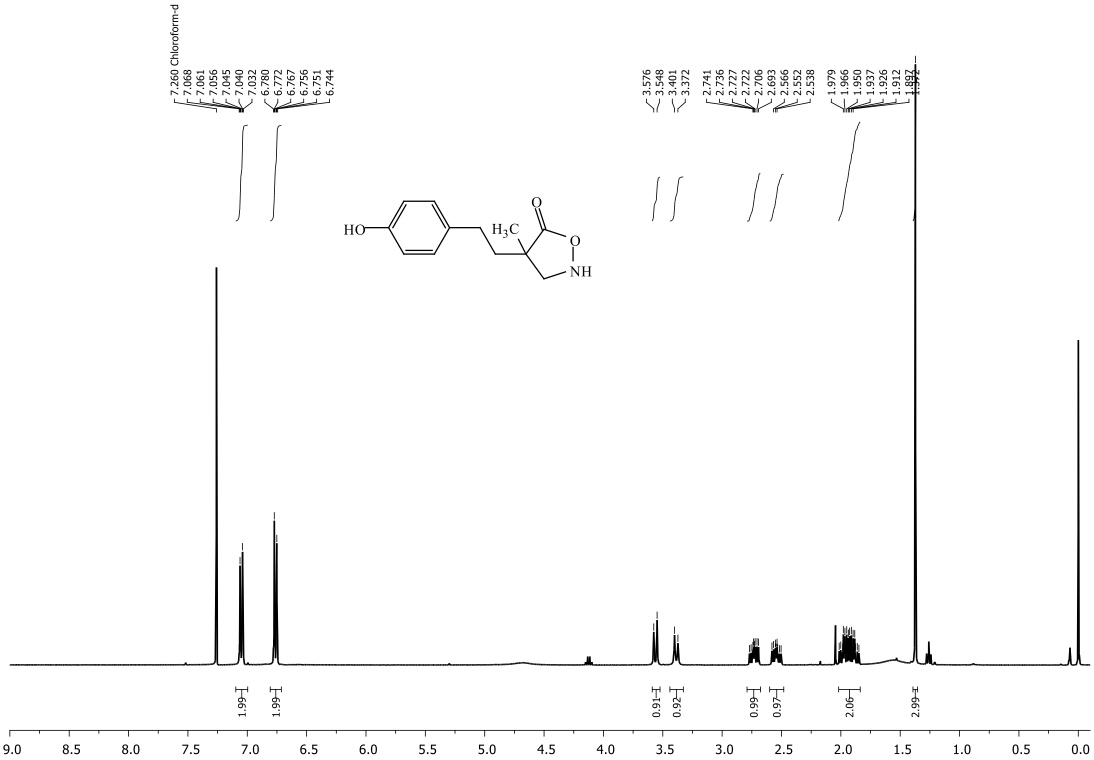

^{13}C NMR (101 MHz, $CDCl_3$)

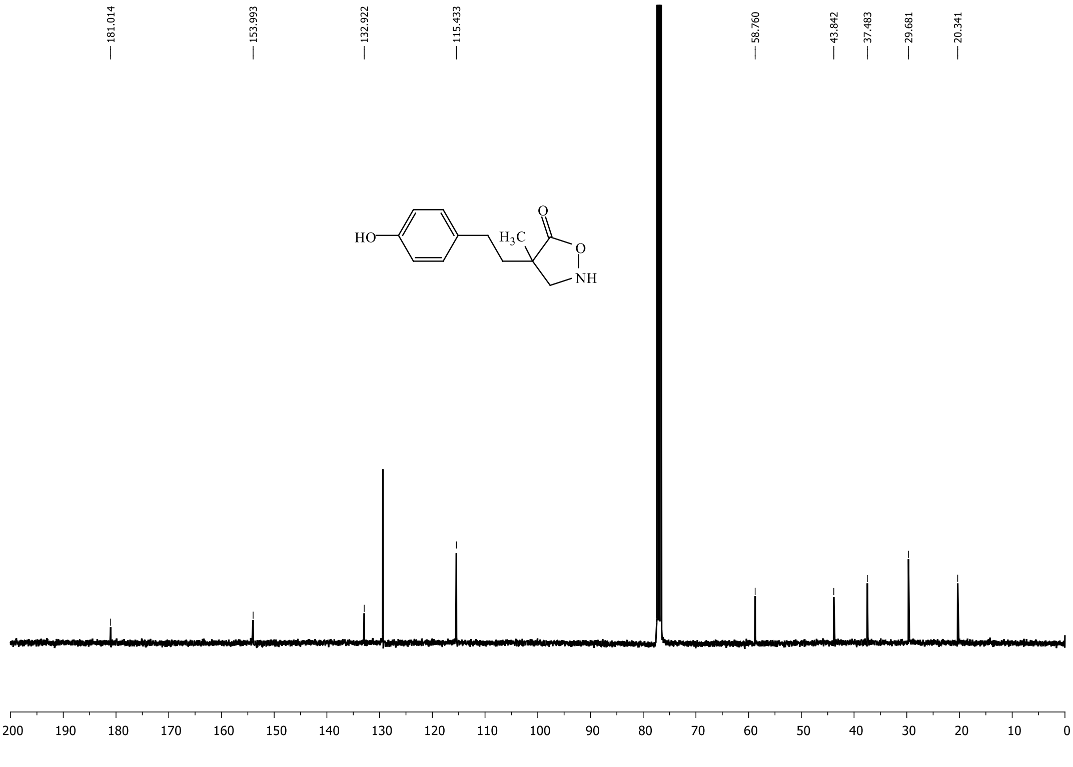

3-Phenyl-3λ^3-isoxazolidin-5-one-3-^{13}C (^{13}C-12)

^{1}H NMR (400 MHz, $CDCl_3$)

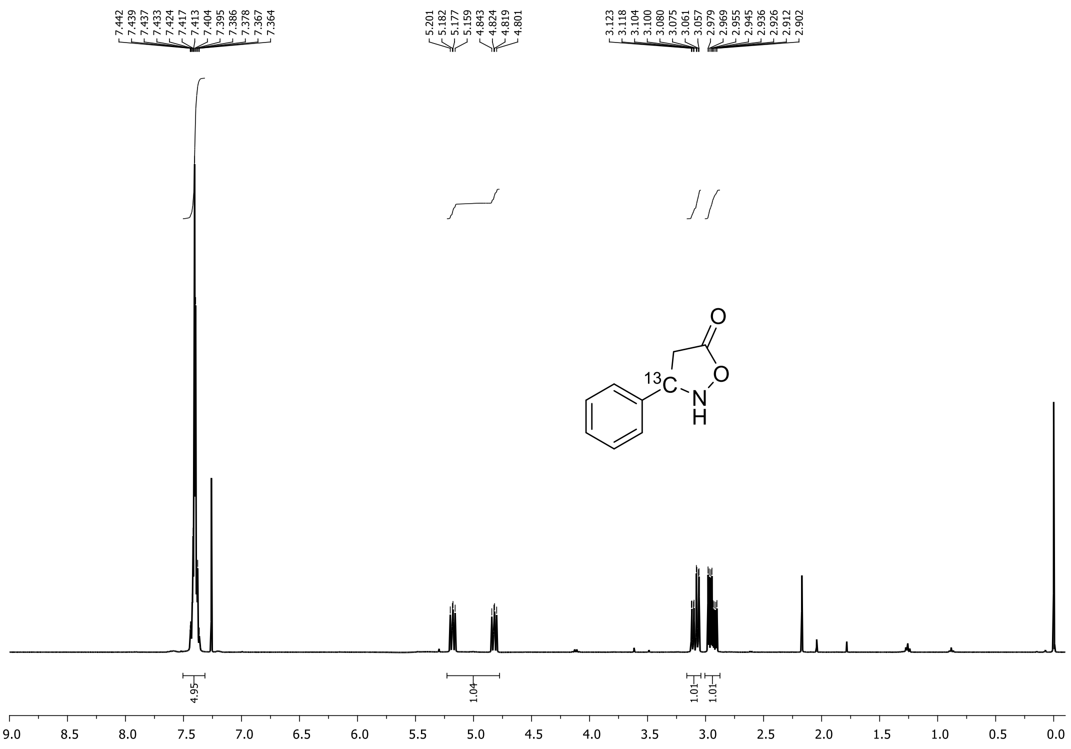

^{13}C NMR (101 MHz, $CDCl_3$)

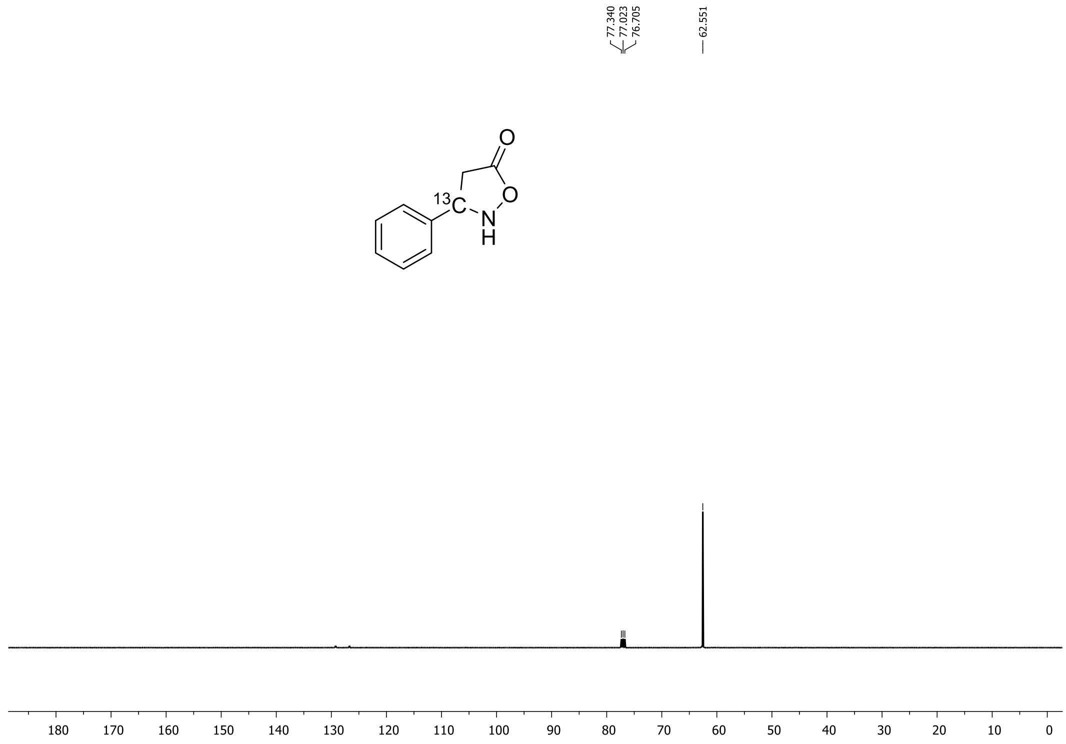

3-Phenylisoxazolidin-5-one (12)

^{1}H NMR (400 MHz, $CDCl_3$)

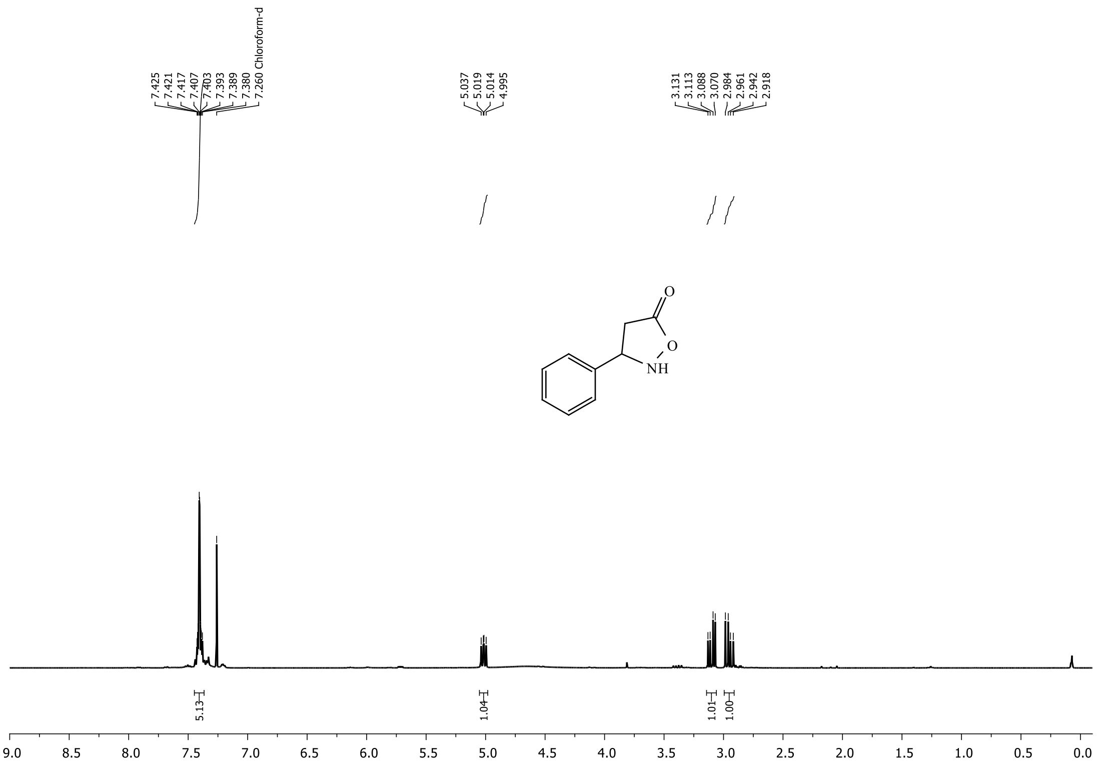

^{13}C NMR (101 MHz, $CDCl_3$)

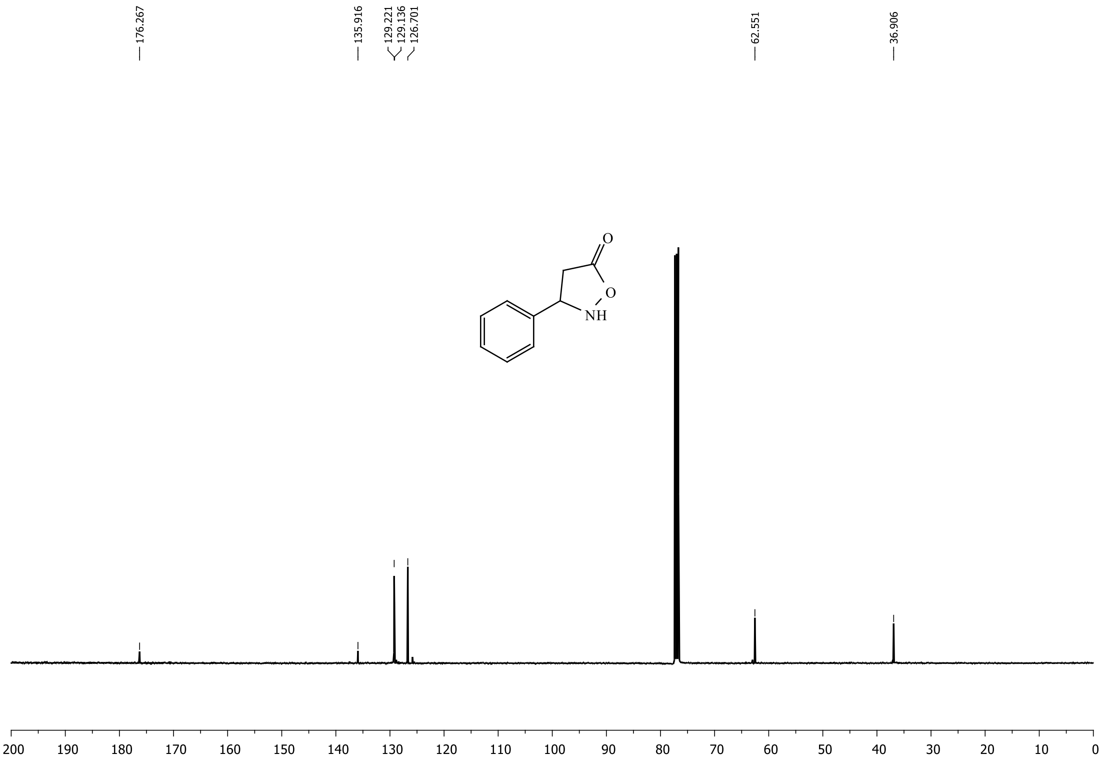

4,4-Dibenzyl-3-phenylisoxazolidin-5-one (13)

^{1}H NMR (400 MHz, $CDCl_3$)

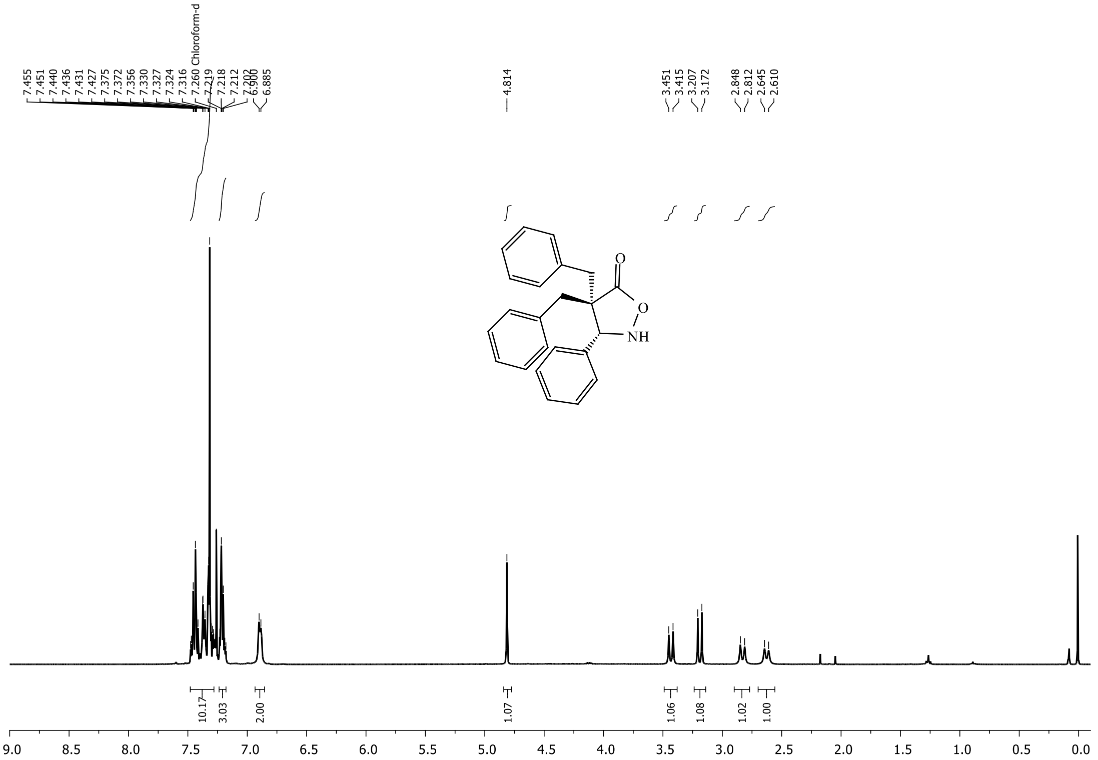

^{13}C NMR (101 MHz, $CDCl_3$)

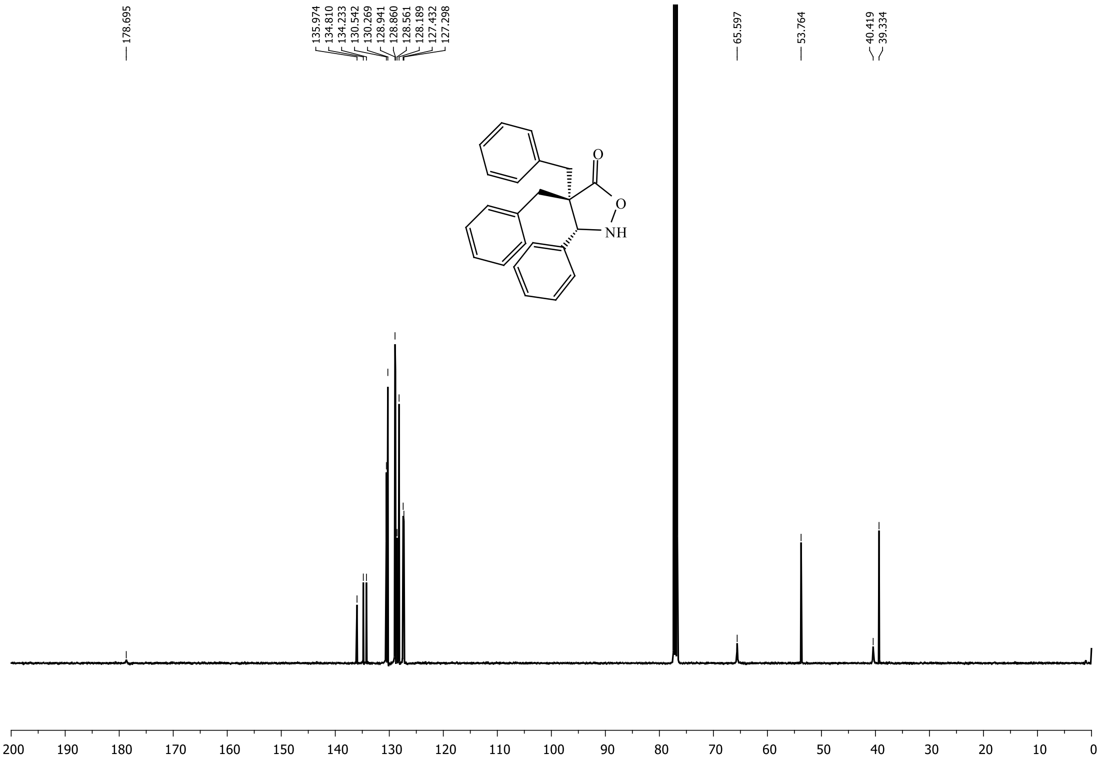

3-Methyl-1,2,3,4-tetrahydroquinoline-3-carboxylic acid (2a)

^{1}H NMR (400 MHz, $CDCl_3$)

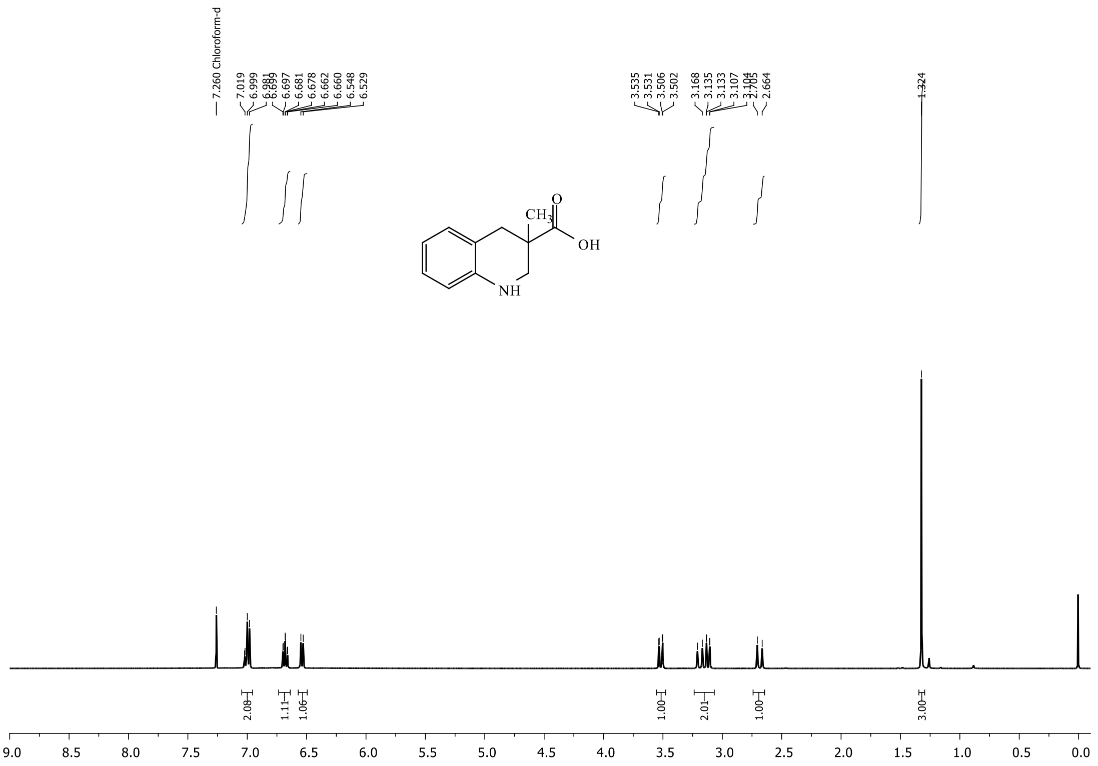

^{13}C NMR (101 MHz, $CDCl_3$)

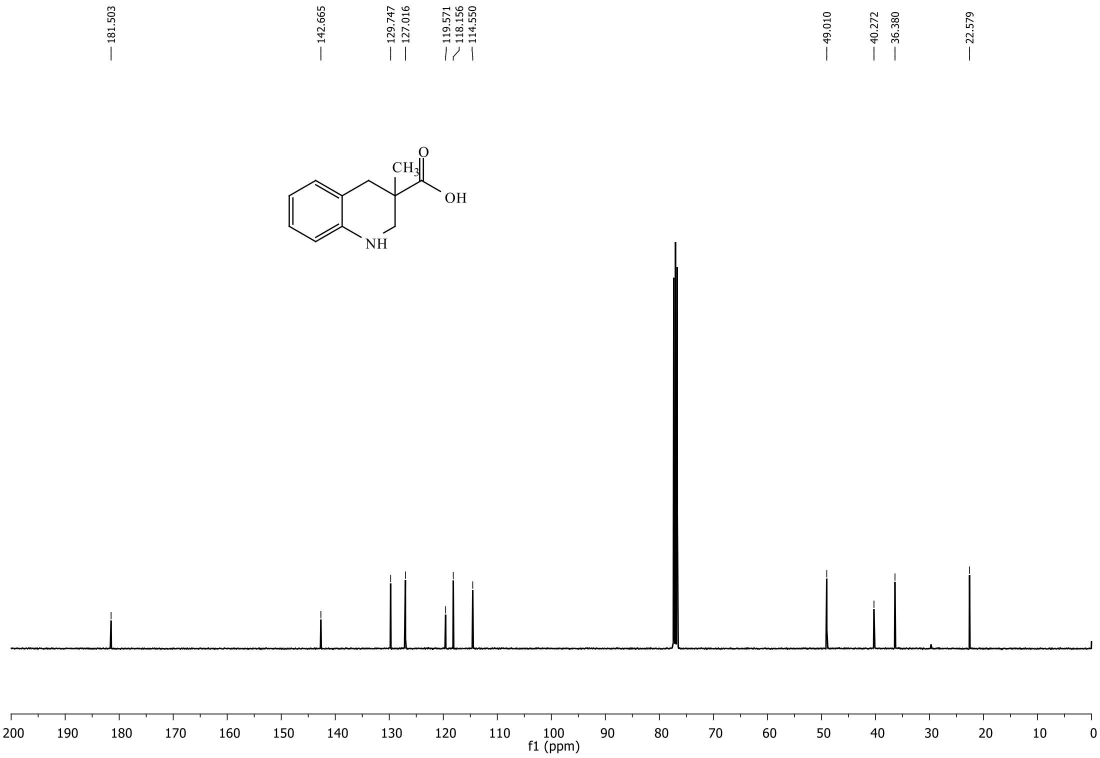

3-Hexyl-1,2,3,4-tetrahydroquinoline-3-carboxylic acid (2b)

^{1}H NMR (400 MHz, $CDCl_3$)

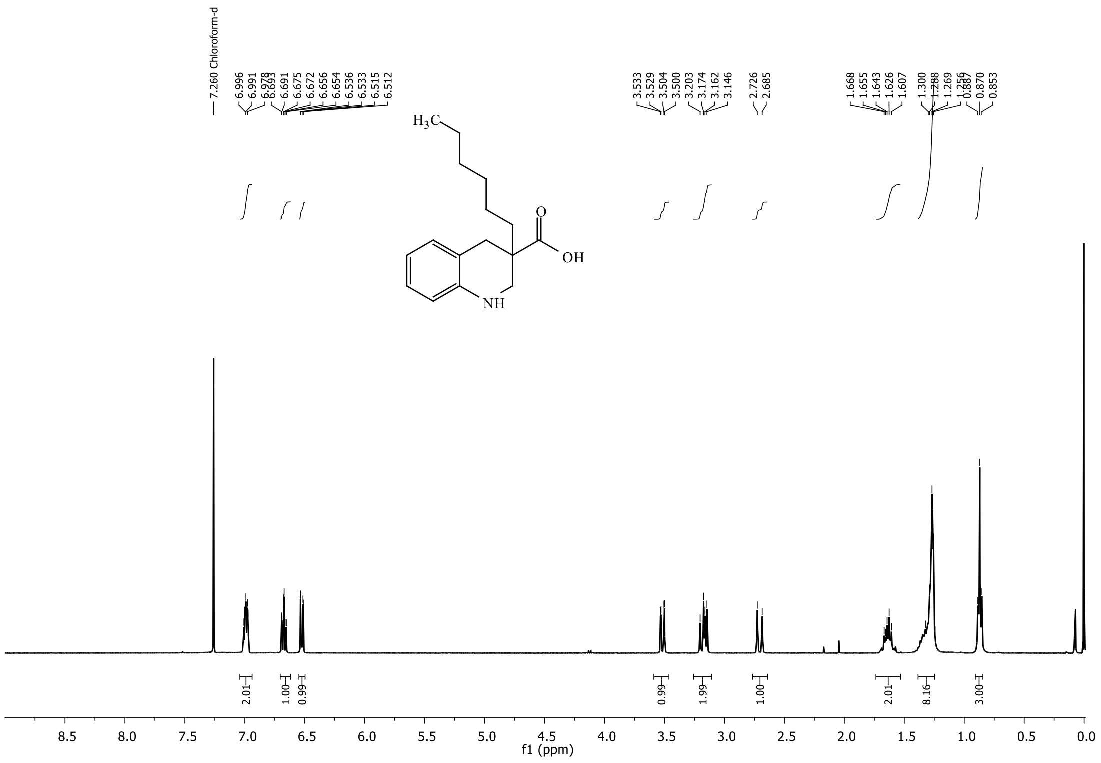

^{13}C NMR (101 MHz, $CDCl_3$)

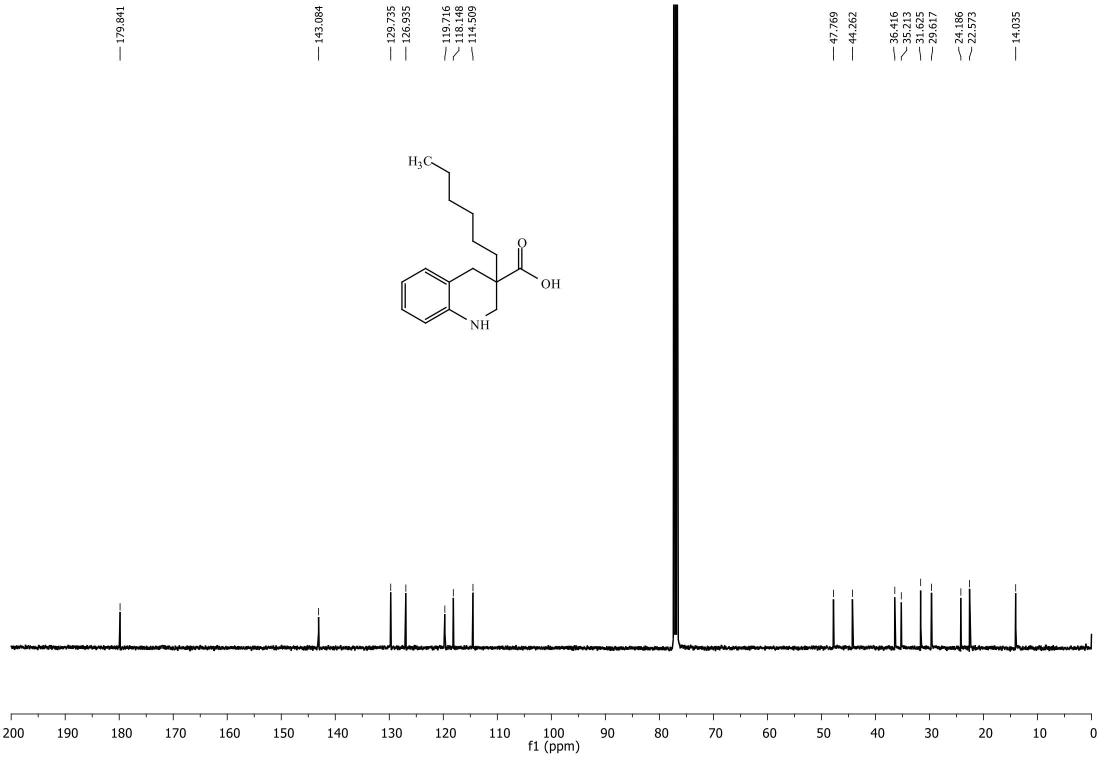

3-Phenethyl-1,2,3,4-tetrahydroquinoline-3-carboxylic acid (2c)

^{1}H NMR (400 MHz, $CDCl_3$)

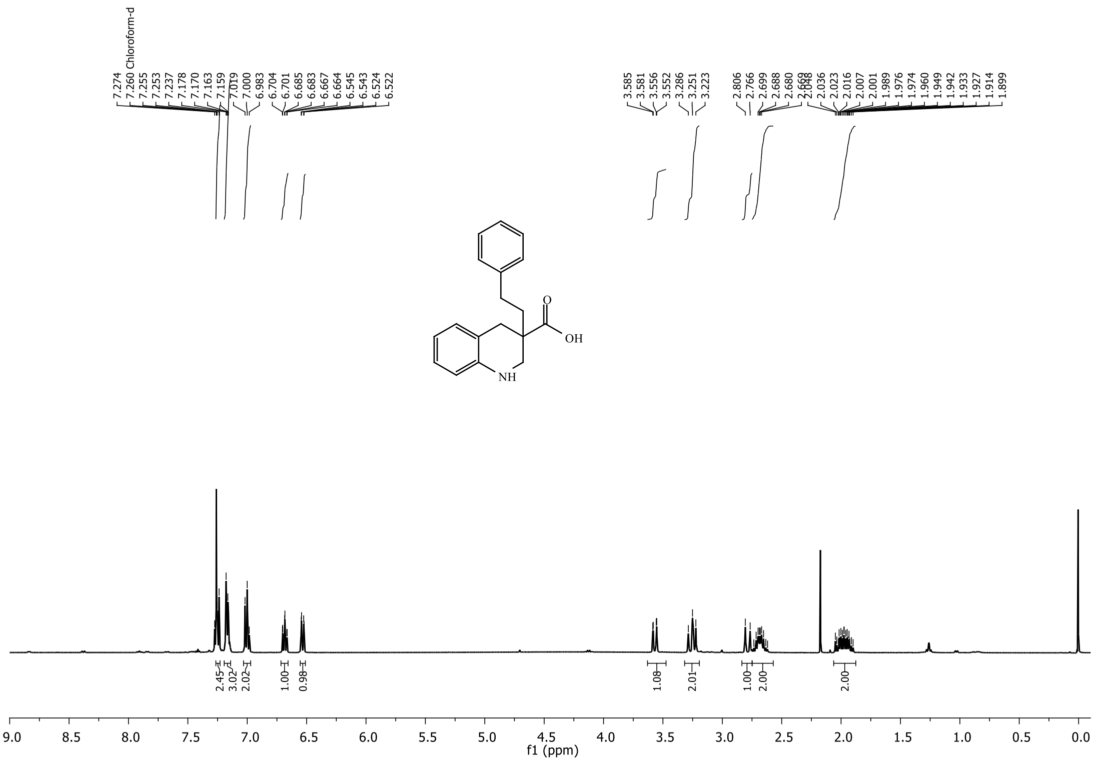

^{13}C NMR (101 MHz, $CDCl_3$)

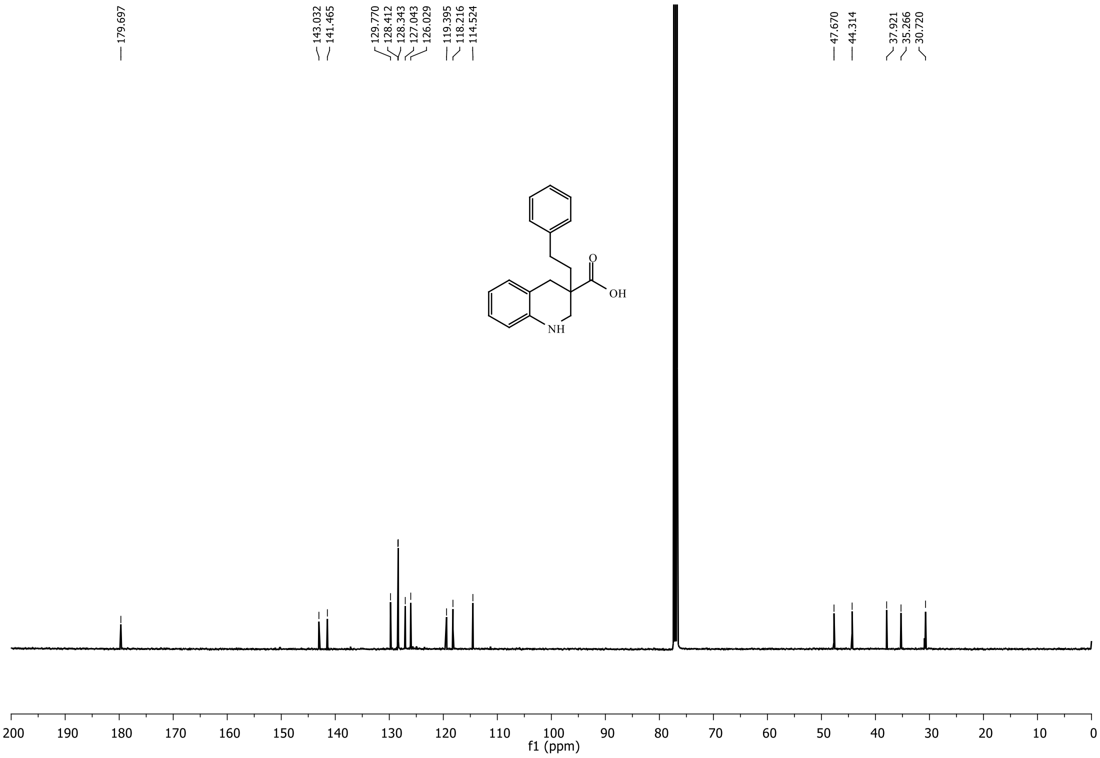

3-(Cyclohexylmethyl)-1,2,3,4-tetrahydroquinoline-3-carboxylic acid (2d)

^{1}H NMR (400 MHz, $CDCl_3$)

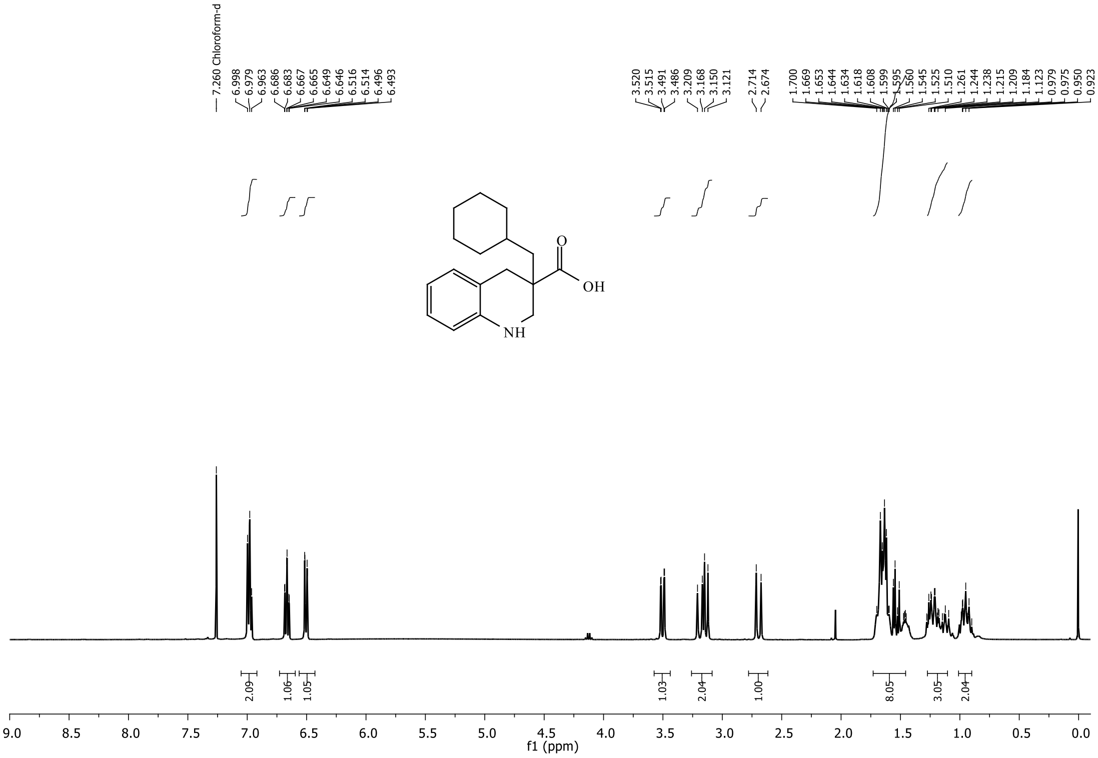

^{13}C NMR (101 MHz, $CDCl_3$)

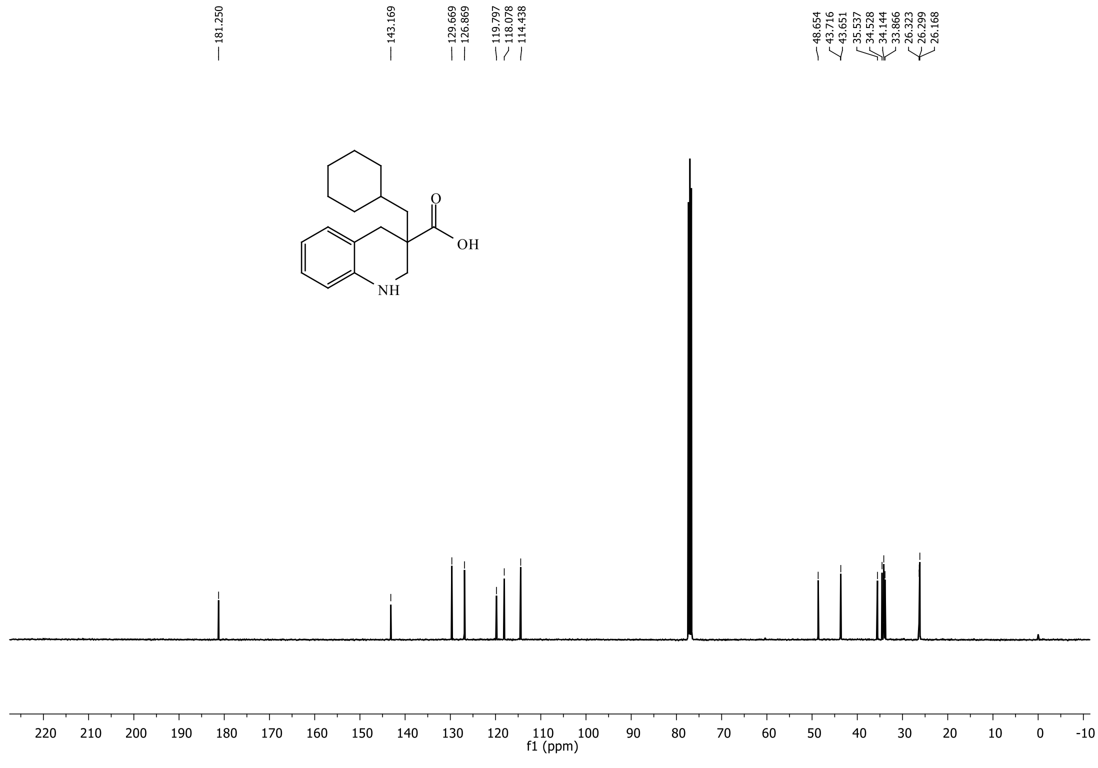

3-(3-(Benzyloxy)-3-oxopropyl)-1,2,3,4-tetrahydroquinoline-3-carboxylic acid (2e)

^{1}H NMR (400 MHz, CDCl$_3$)

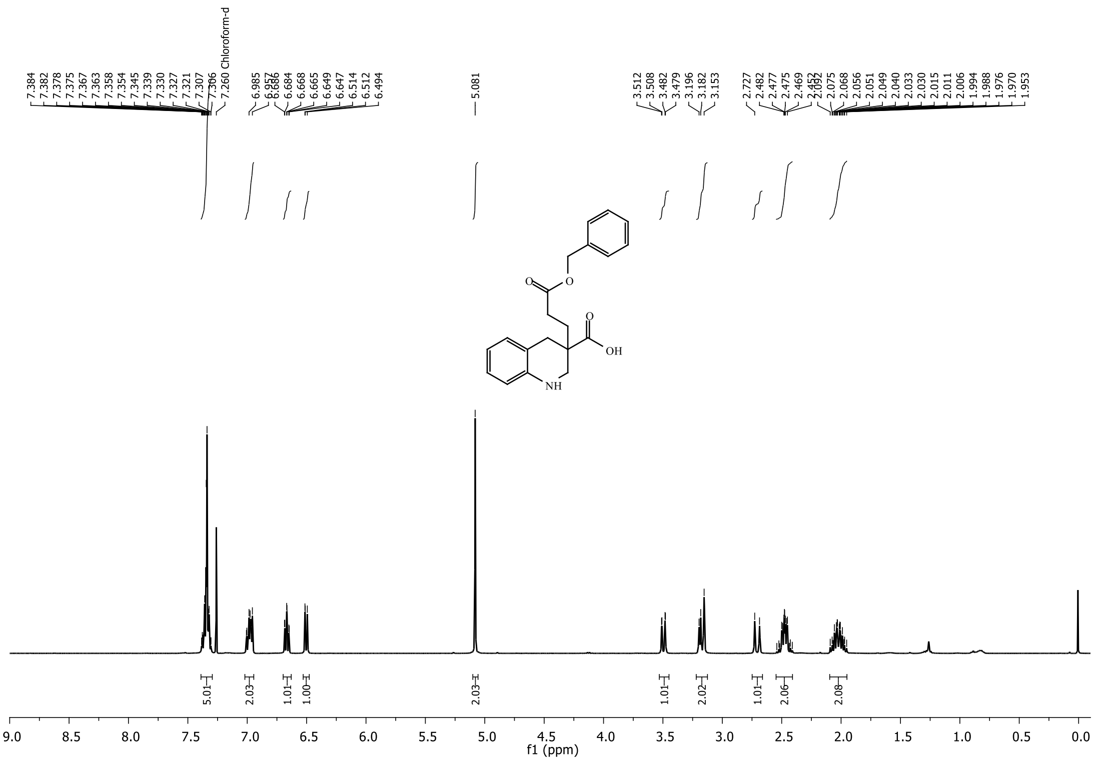

^{13}C NMR (101 MHz, CDCl$_3$)

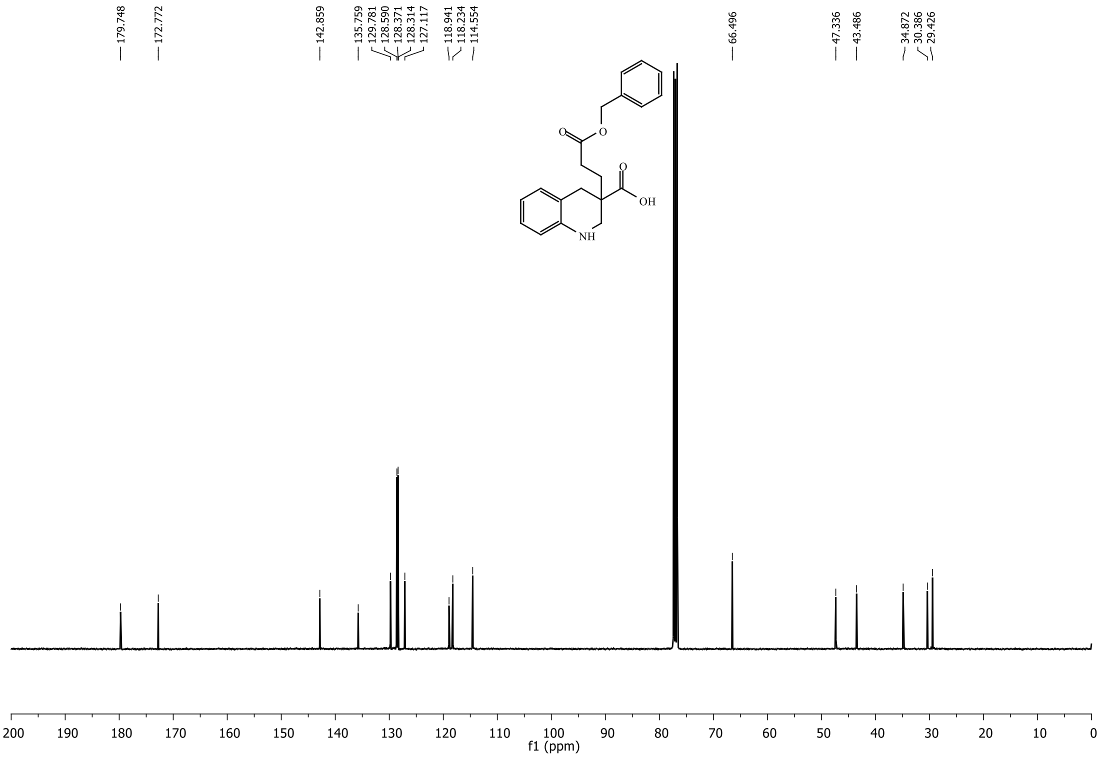

3-(2-(Phenylsulfonyl)ethyl)-1,2,3,4-tetrahydroquinoline-3-carboxylic acid (2f)

^{1}H NMR (400 MHz, CDCl$_3$)

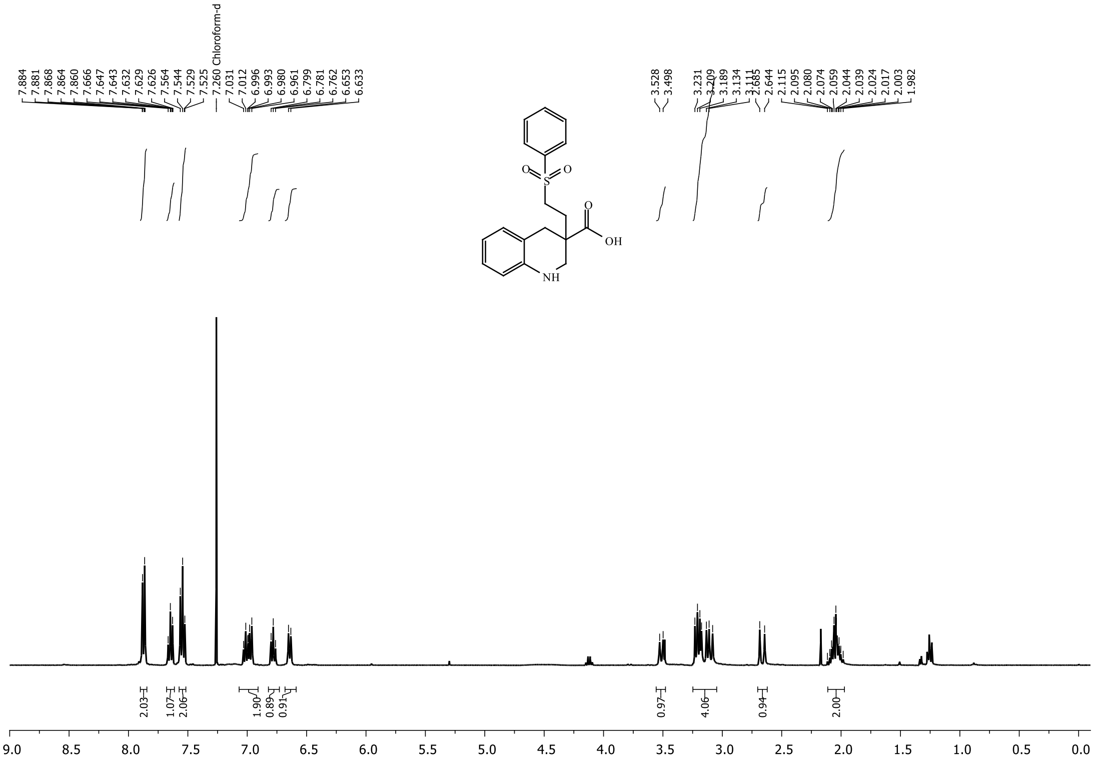

^{13}C NMR (101 MHz, CDCl$_3$)

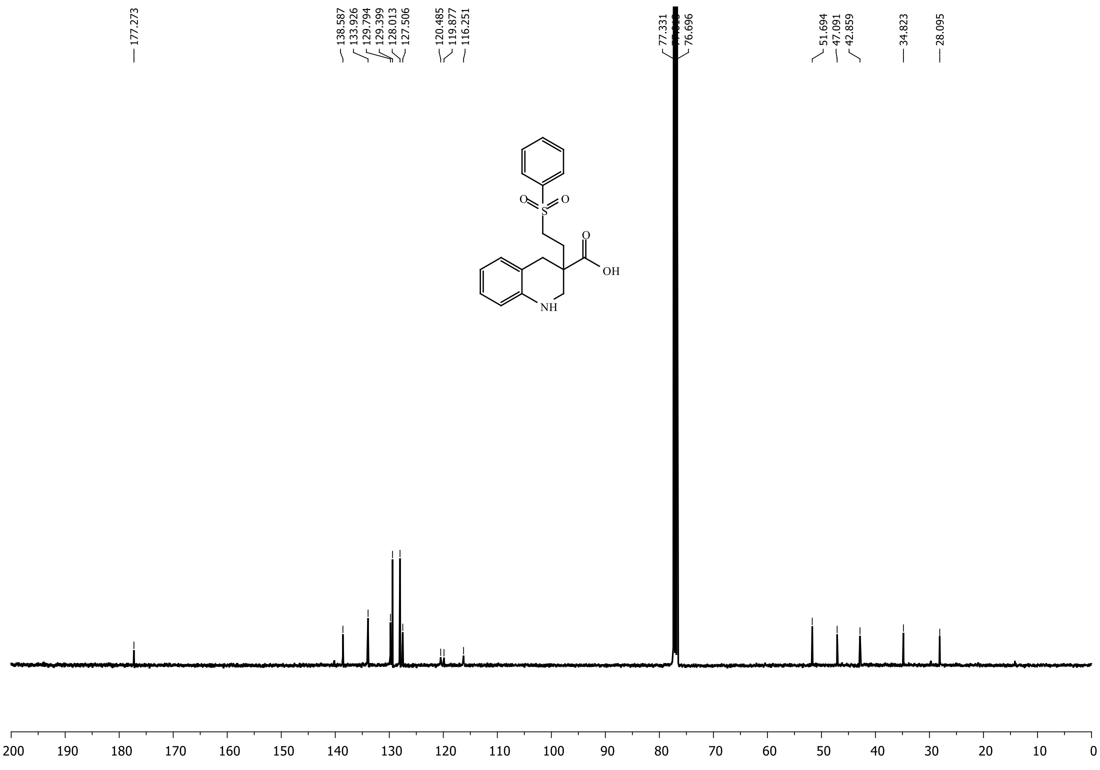

3-(Pent-4-en-1-yl)-1,2,3,4-tetrahydroquinoline-3-carboxylic acid (2g)

^{1}H NMR (400 MHz, $CDCl_3$)

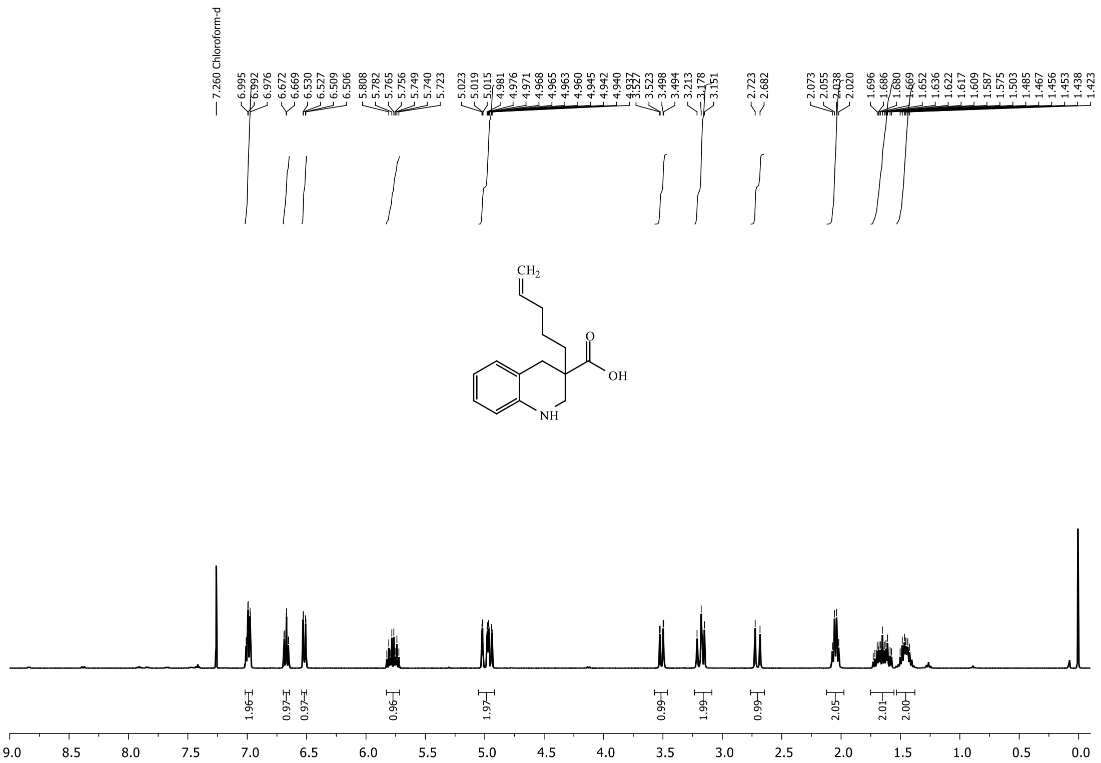

^{13}C NMR (101 MHz, $CDCl_3$)

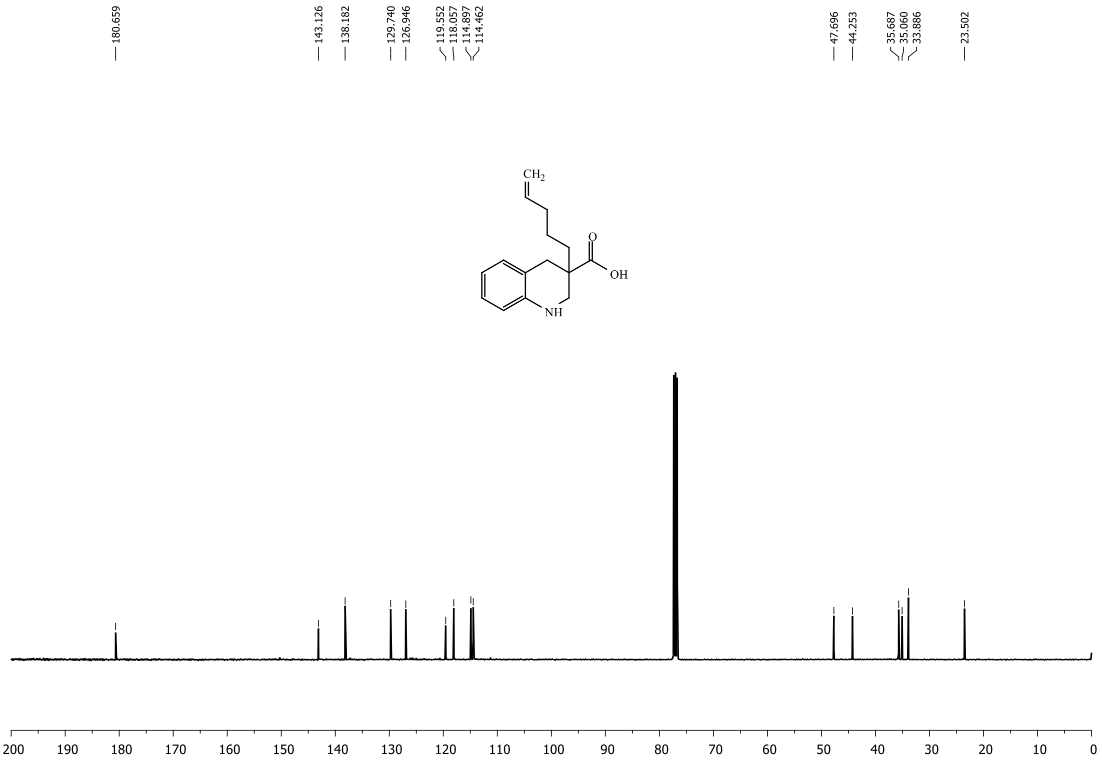

3-(Cyanomethyl)-1,2,3,4-tetrahydroquinoline-3-carboxylic acid (2h)

^{1}H NMR (400 MHz, CD$_3$OD)

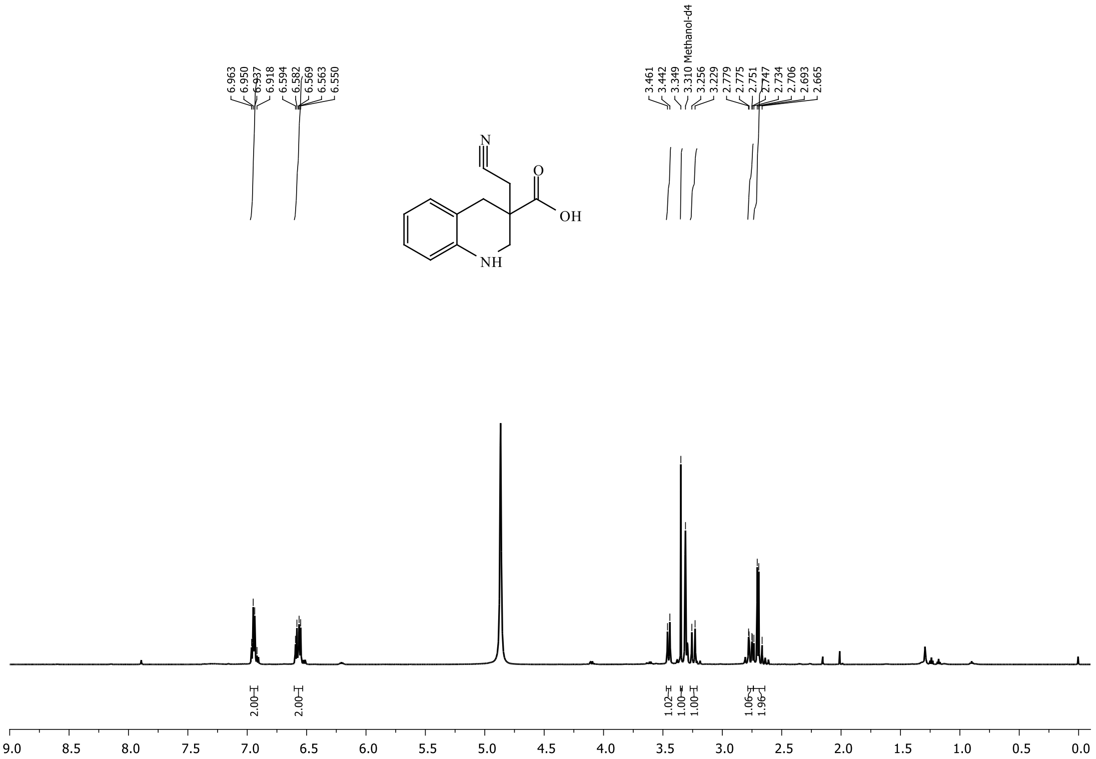

^{13}C NMR (101 MHz, CD$_3$OD)

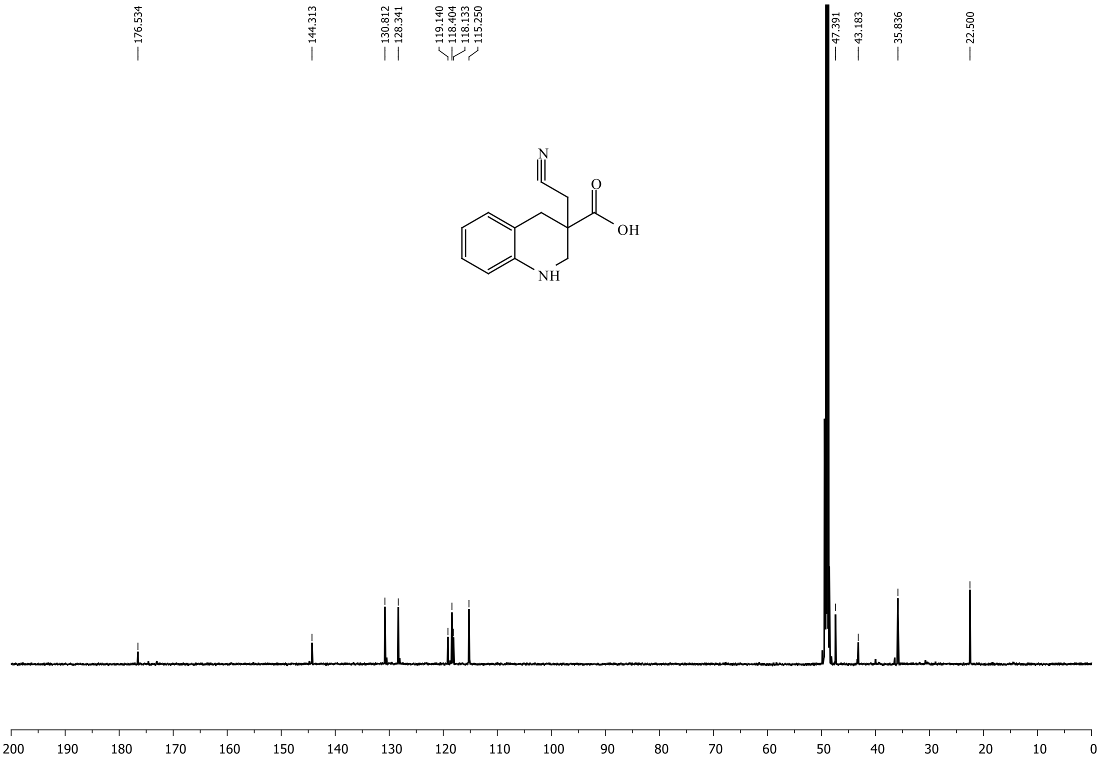

5-Methylene-1',4,4',5-tetrahydro-2H,2'H-spiro[furan-3,3'-quinolin]-2-one (5)

^{1}H NMR (400 MHz, $CDCl_3$)

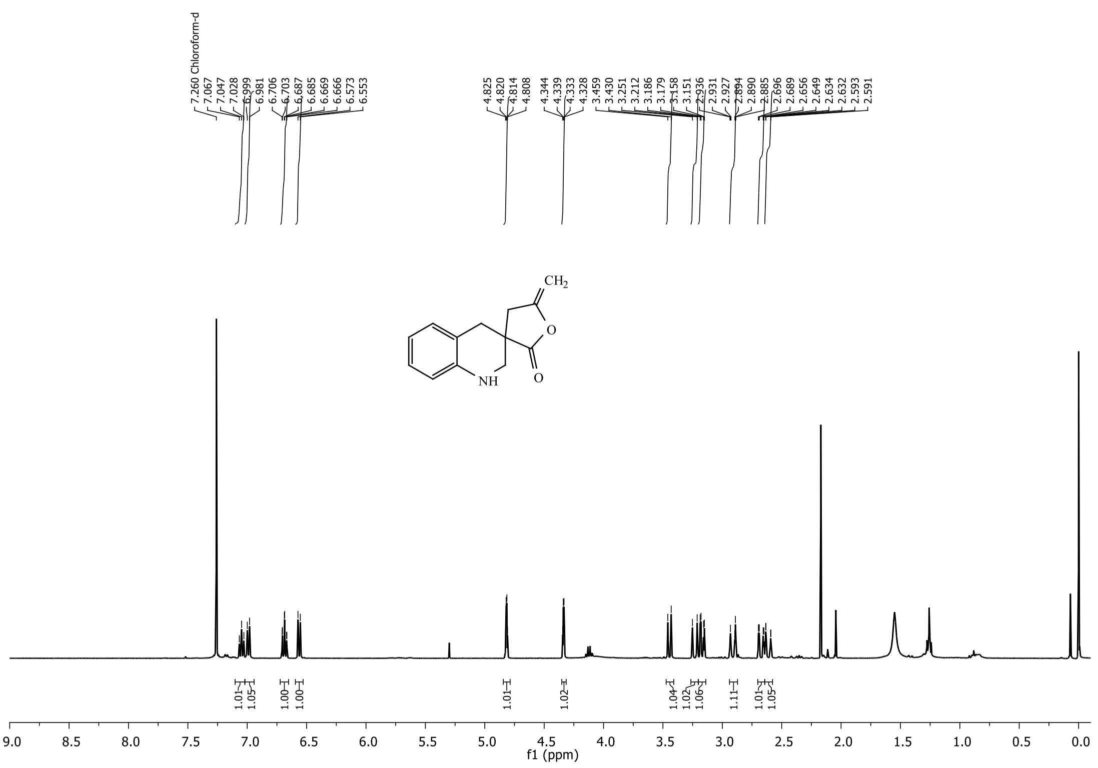

^{13}C NMR (101 MHz, $CDCl_3$)

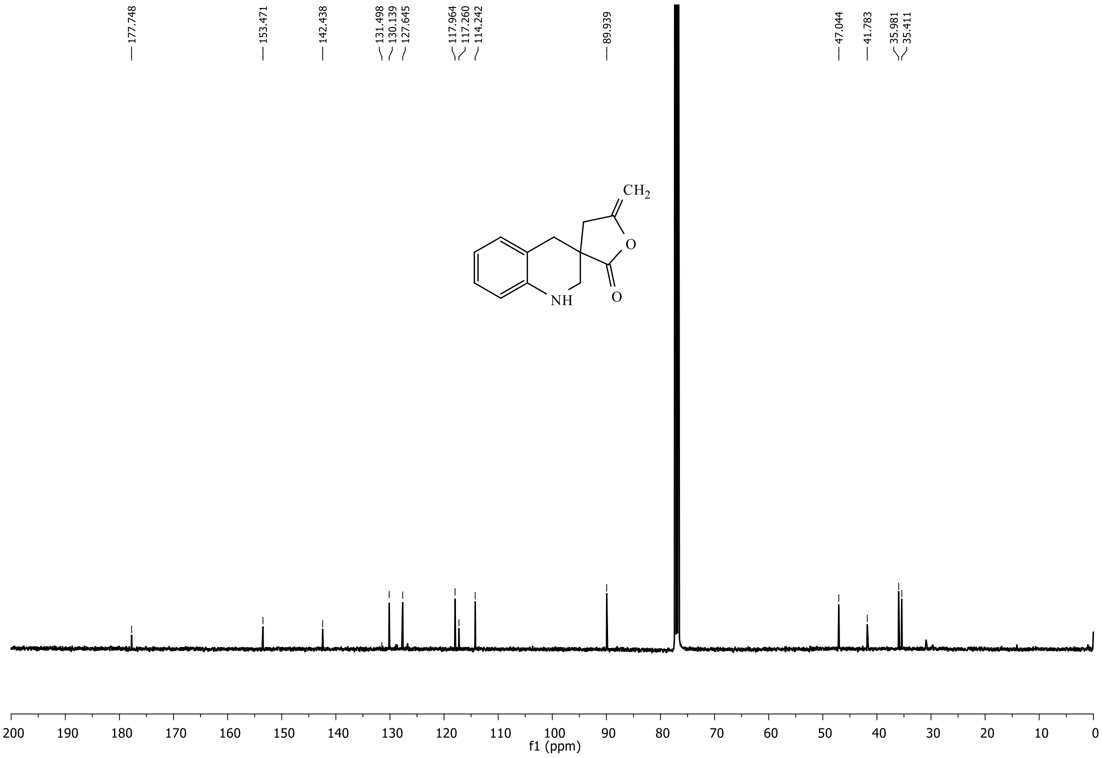

DEPT 90

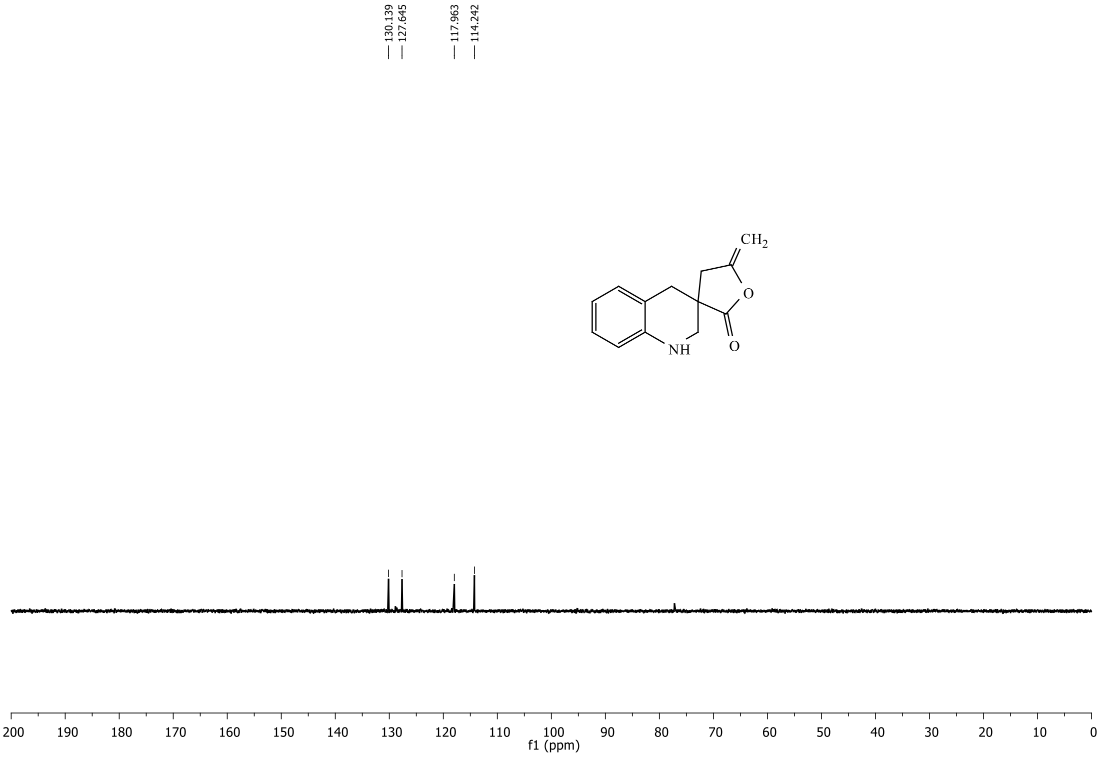

DEPT 135

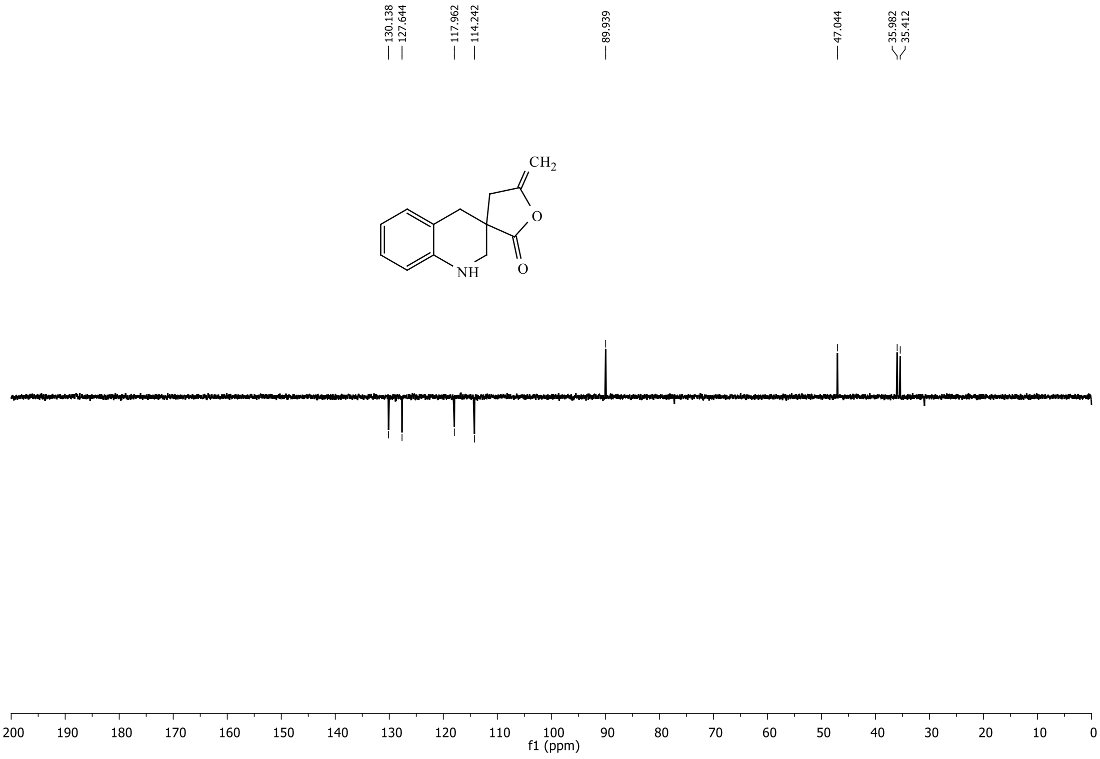

3,7-Dimethyl-1,2,3,4-tetrahydroquinoline-3-carboxylic acid (2j)

^{1}H NMR (400 MHz, CDCl₃)

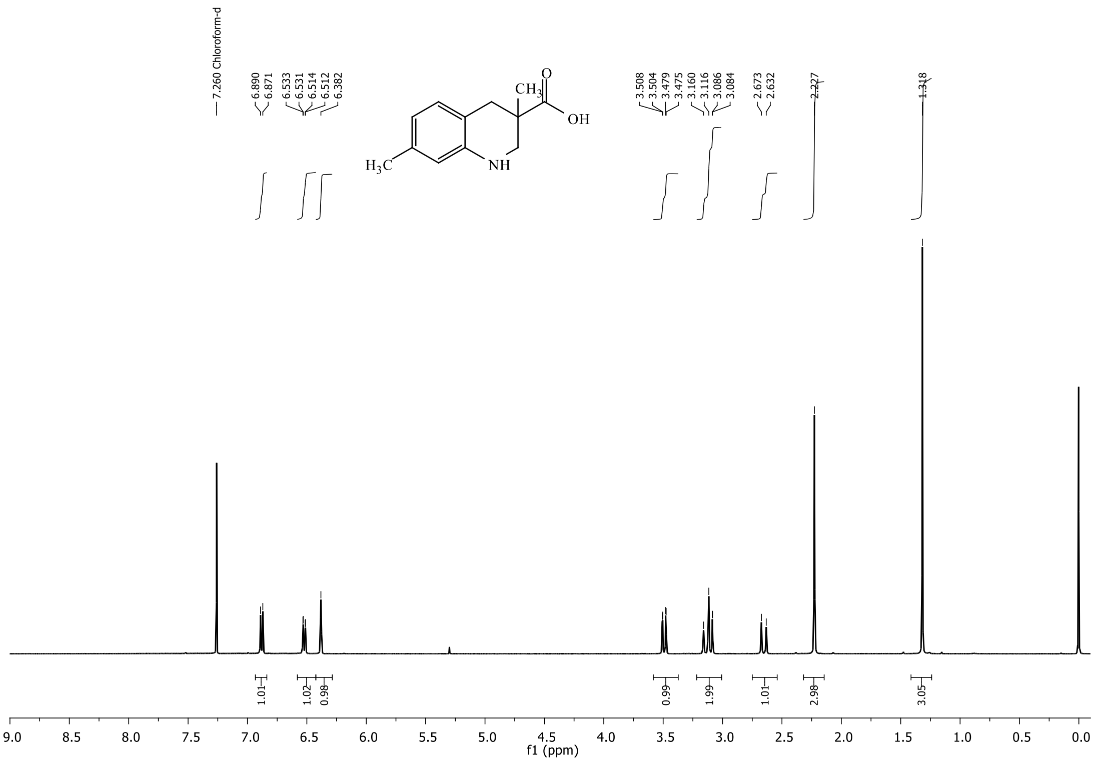

^{13}C NMR (101 MHz, CDCl₃)

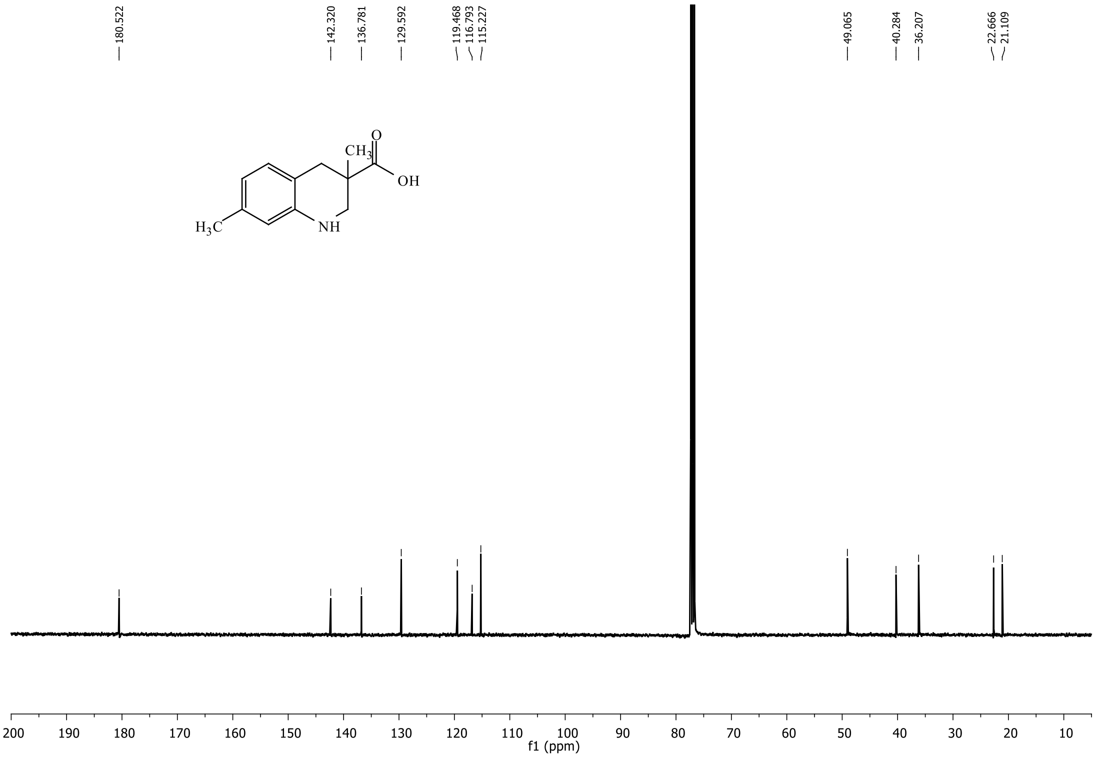

3,5-Dimethyl-1,2,3,4-tetrahydroquinoline-3-carboxylic acid (2k)

^{1}H NMR (400 MHz, $CDCl_3$)

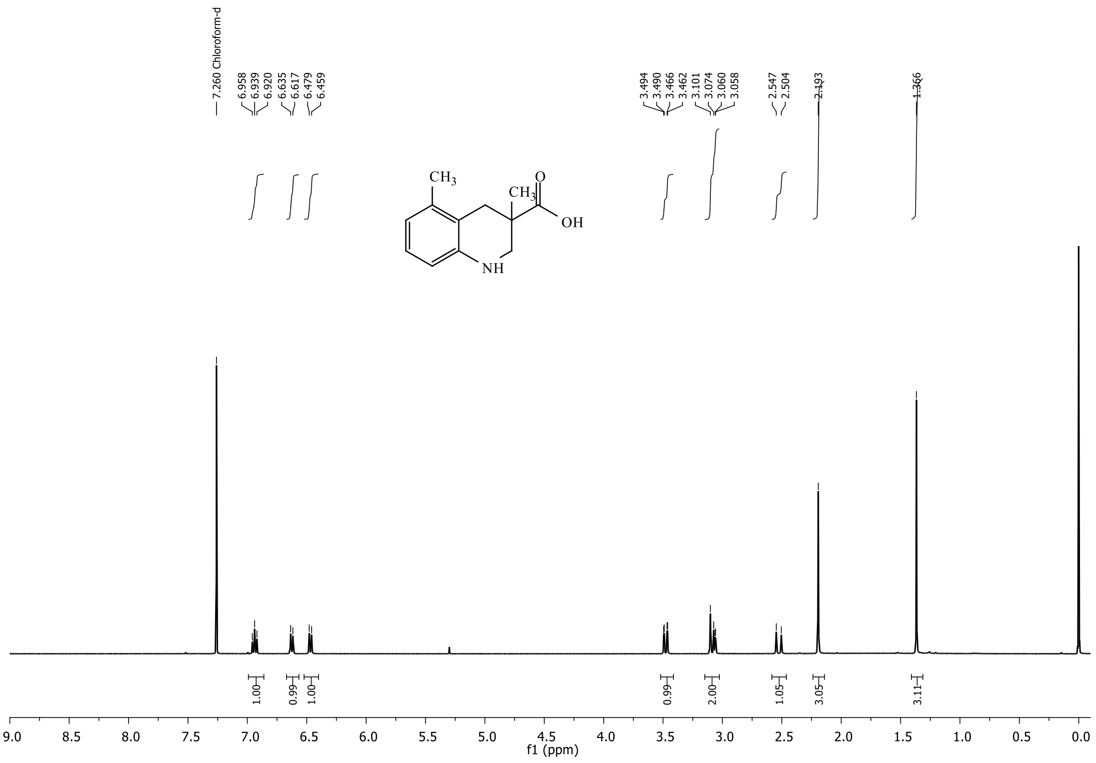

^{13}C NMR (101 MHz, $CDCl_3$)

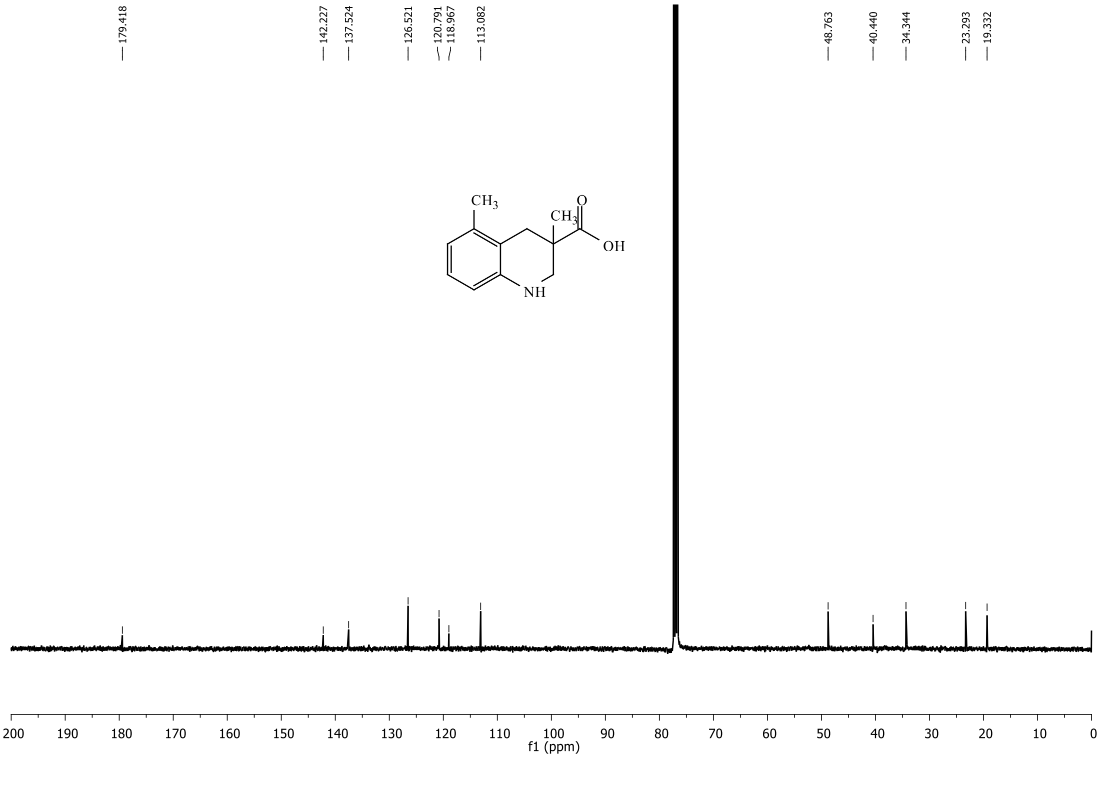

7-Bromo-3-methyl-1,2,3,4-tetrahydroquinoline-3-carboxylic acid (2l)

^{1}H NMR (400 MHz, CD_3OD)

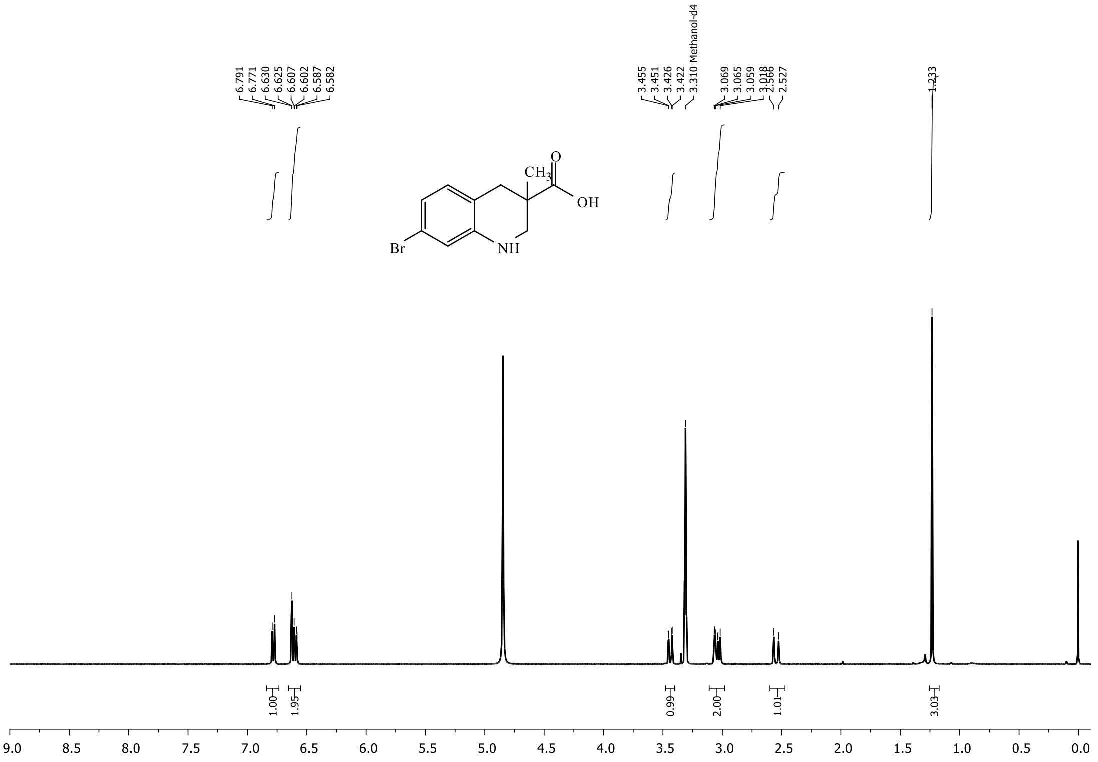

^{13}C NMR (101 MHz, CD_3OD)

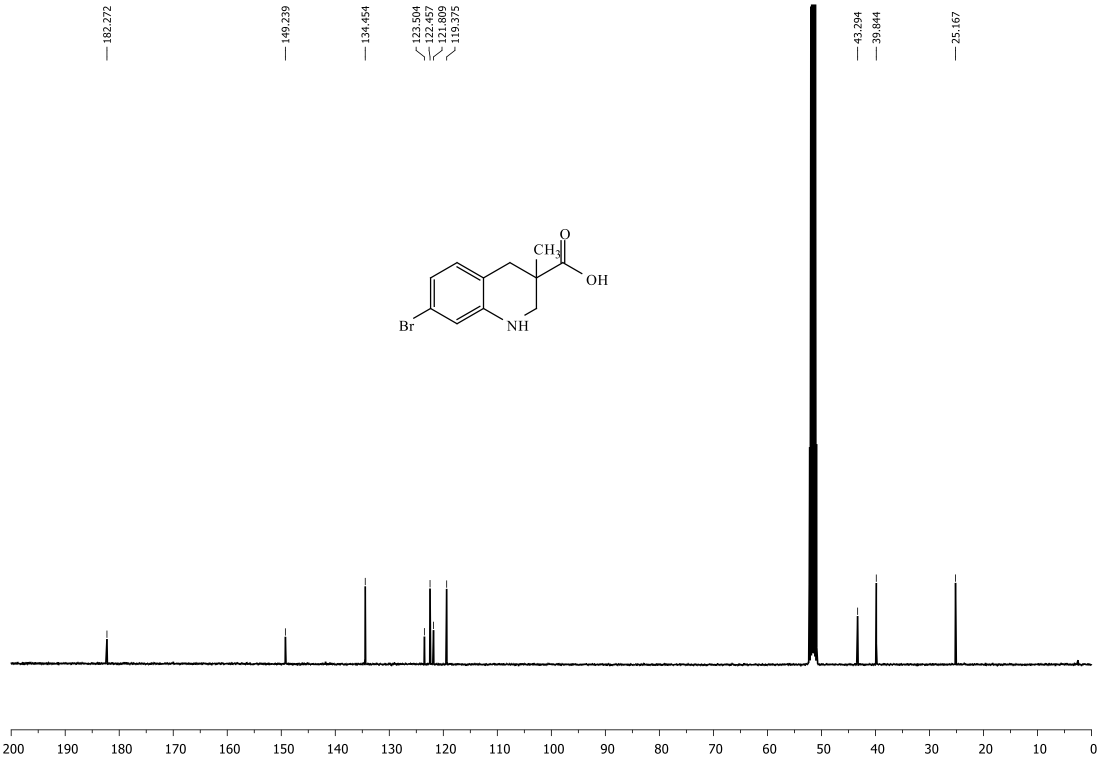

5-Chloro-3-methyl-1,2,3,4-tetrahydroquinoline-3-carboxylic acid (2m)

^{1}H NMR (400 MHz, $CDCl_3$)

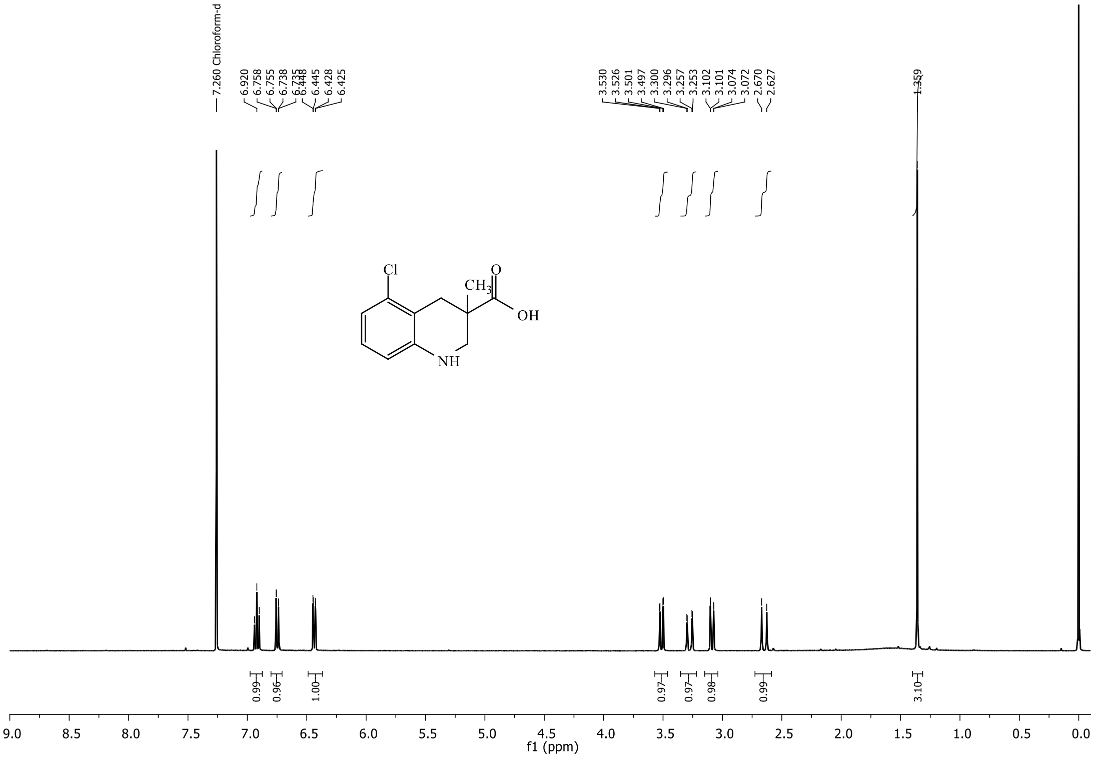

^{13}C NMR (101 MHz, $CDCl_3$)

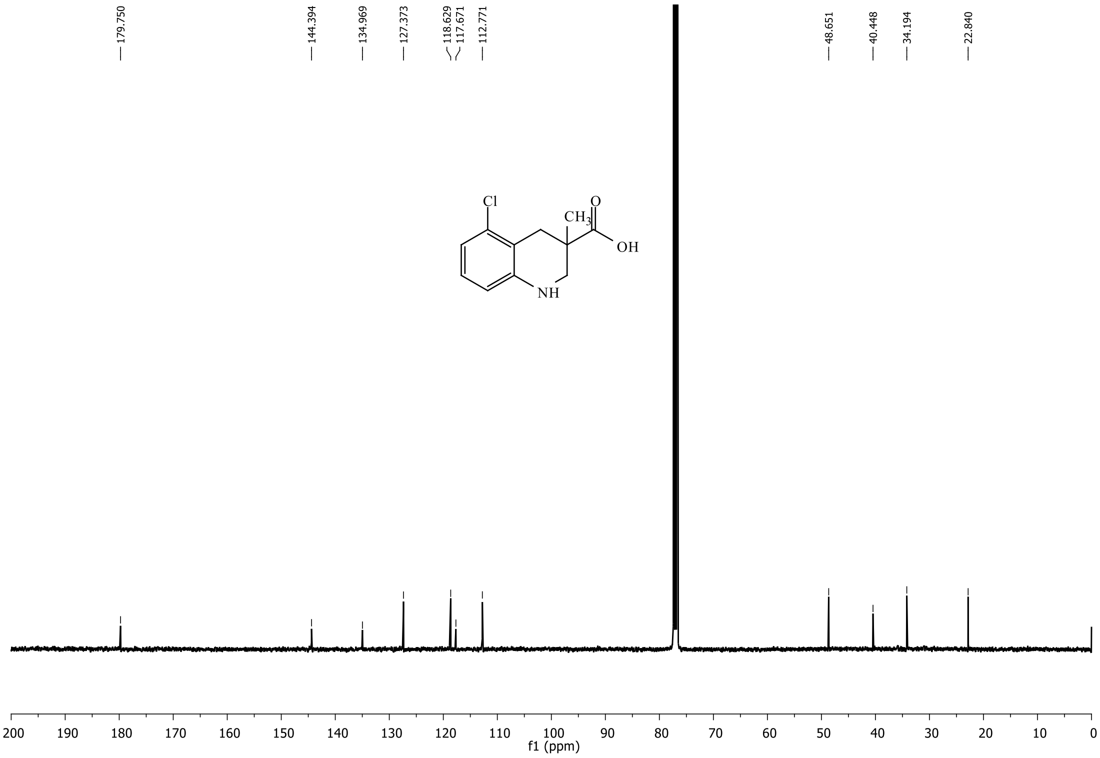

3,6-Dimethyl-1,2,3,4-tetrahydroquinoline-3-carboxylic acid (2n)

^{1}H NMR (400 MHz, $CDCl_3$)

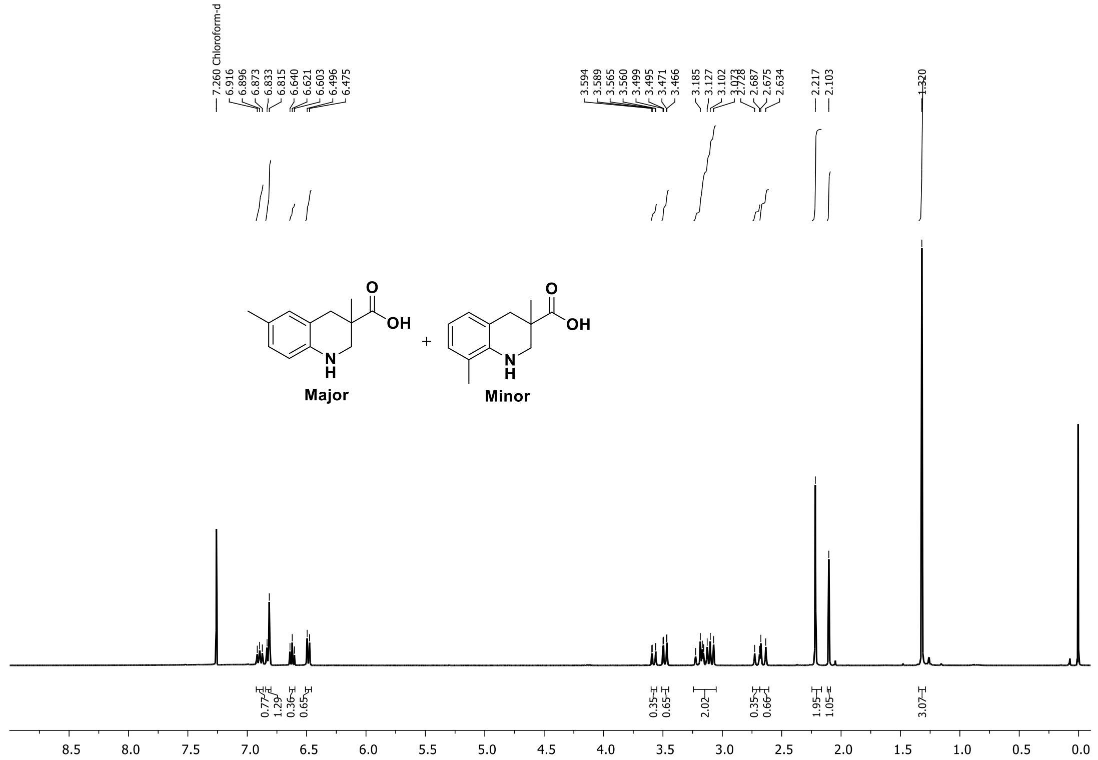

^{13}C NMR (101 MHz, $CDCl_3$)

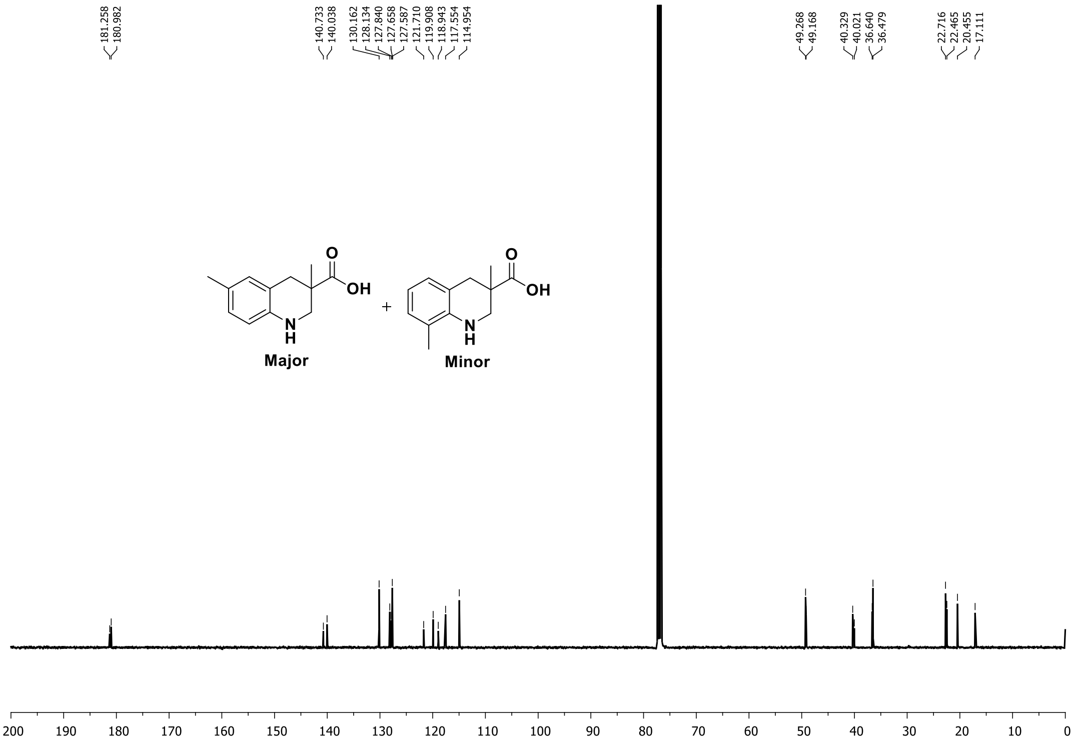

6-Methoxy-3-methyl-1,2,3,4-tetrahydroquinoline-3-carboxylic acid (2oa)

^{1}H NMR (400 MHz, $CDCl_3$)

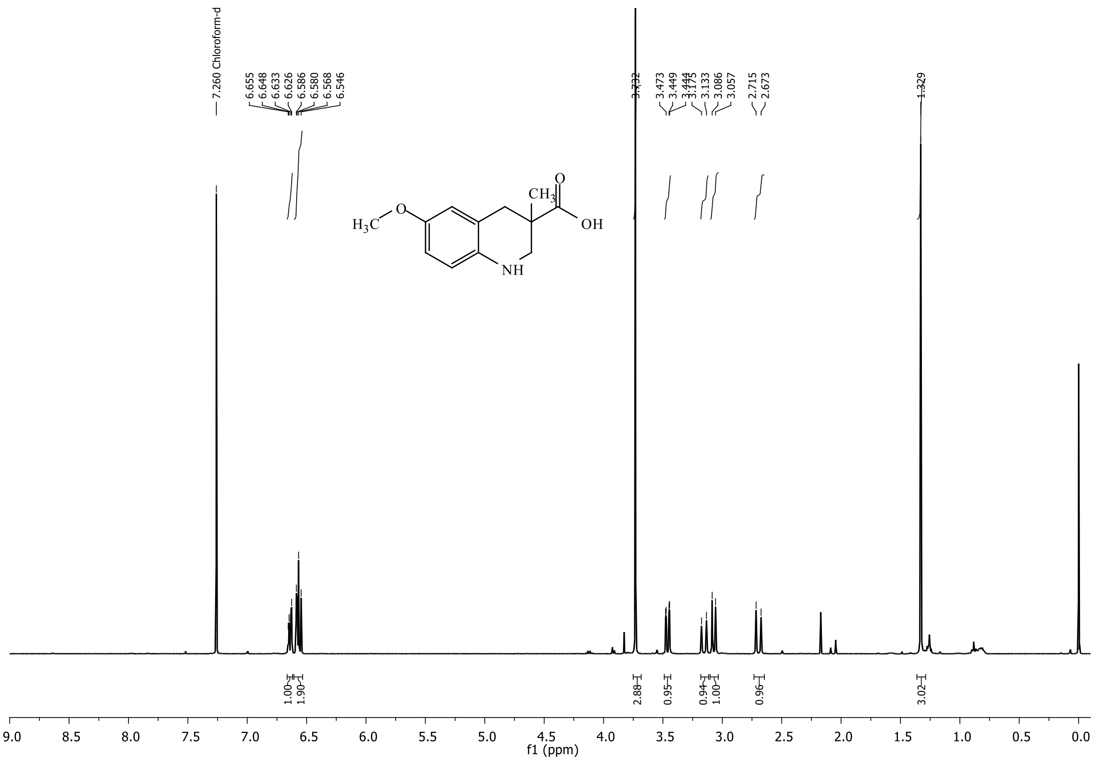

^{13}C NMR (101 MHz, $CDCl_3$)

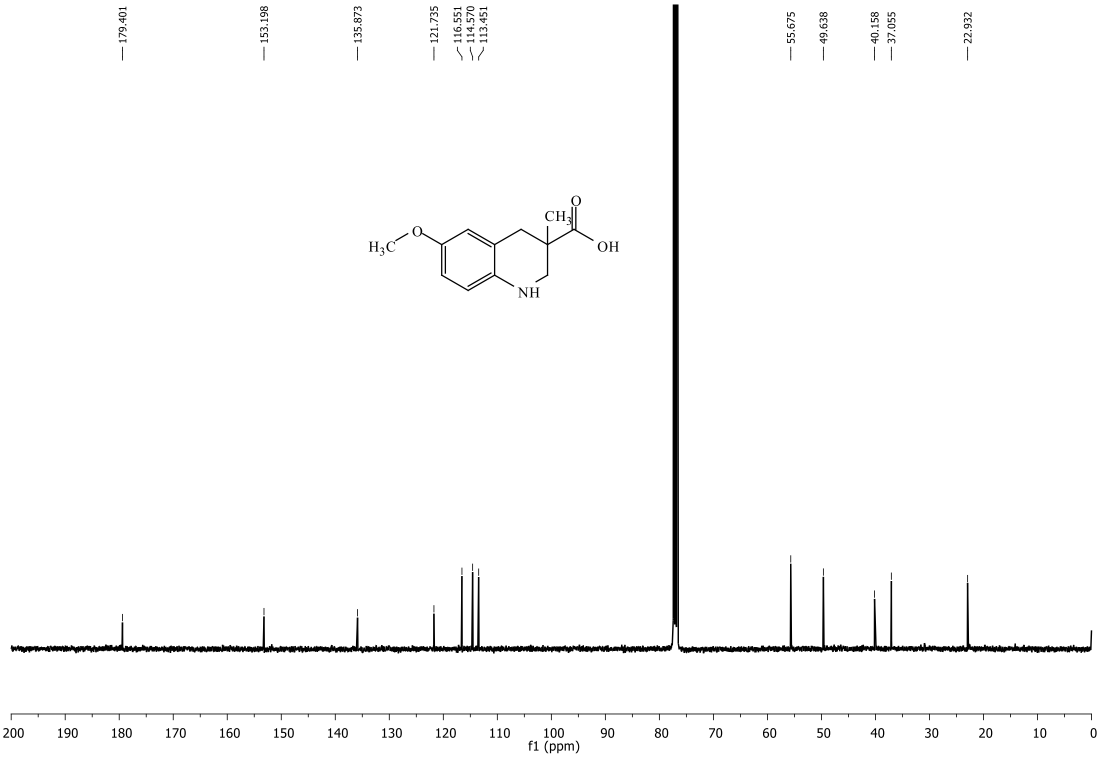

8-Methoxy-3-methyl-1,2,3,4-tetrahydroquinoline-3-carboxylic acid (2ob)

^{1}H NMR (400 MHz, $CDCl_3$)

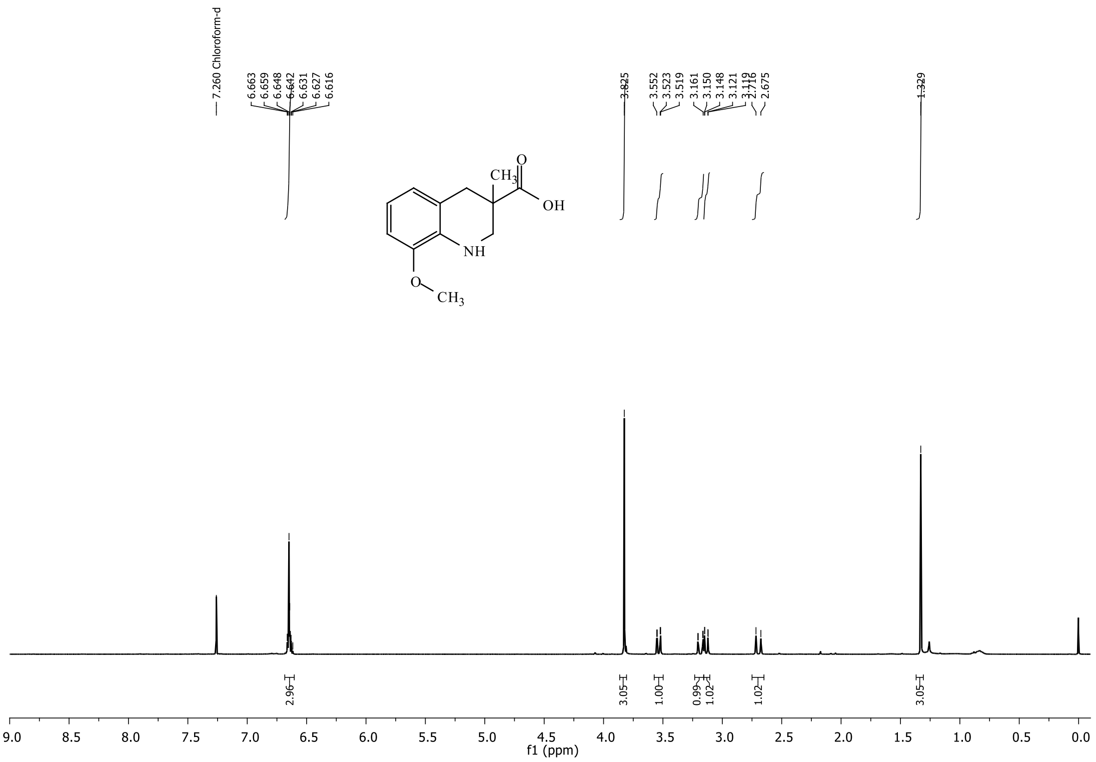

^{13}C NMR (101 MHz, $CDCl_3$)

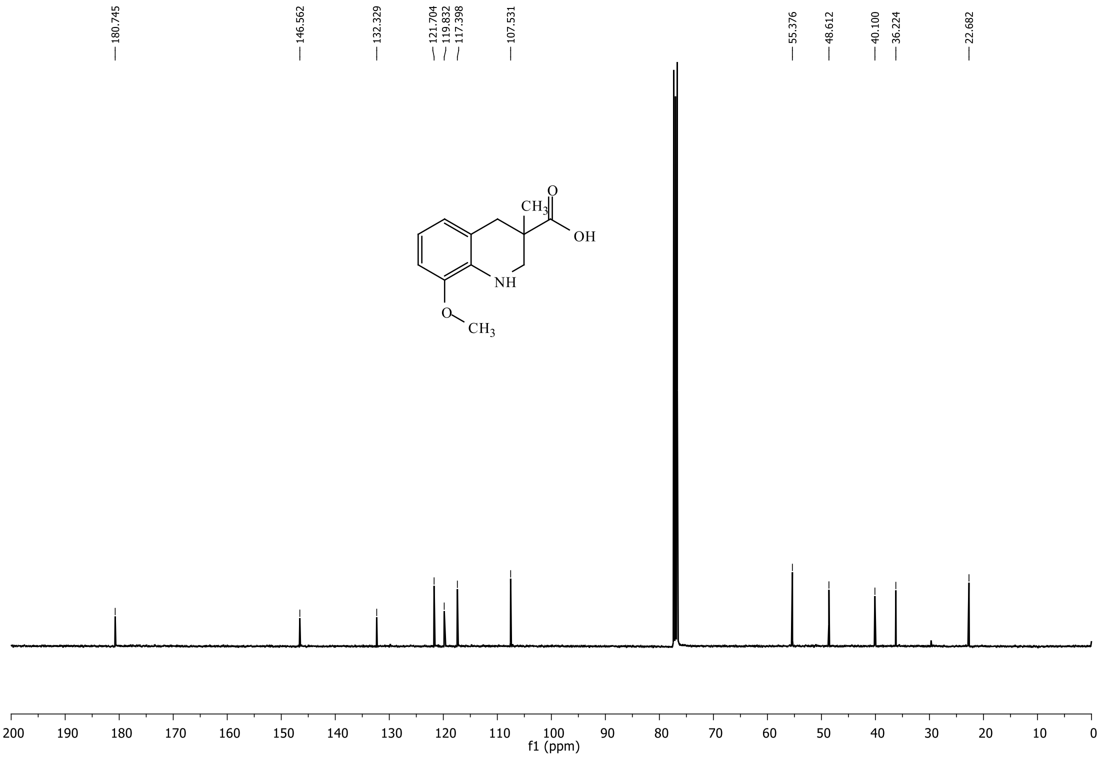

6-Fluoro-3-methyl-1,2,3,4-tetrahydroquinoline-3-carboxylic acid (2p)

^{1}H NMR (400 MHz, $CDCl_3$)

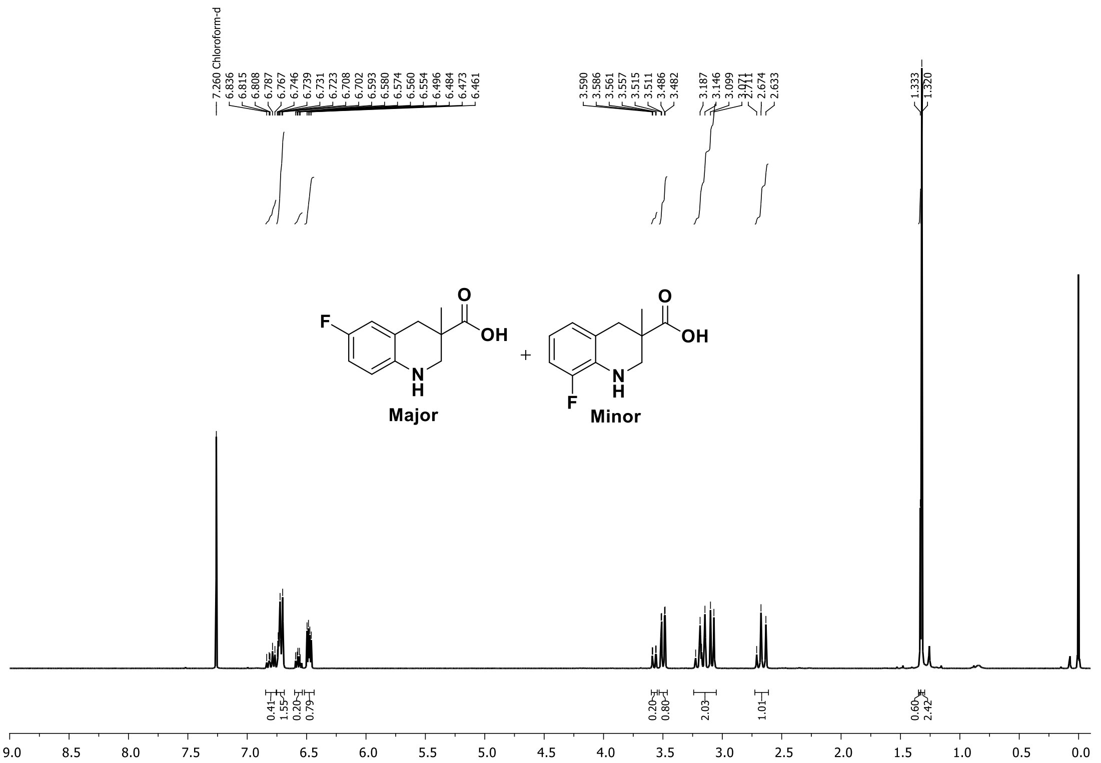

^{13}C NMR (101 MHz, $CDCl_3$)

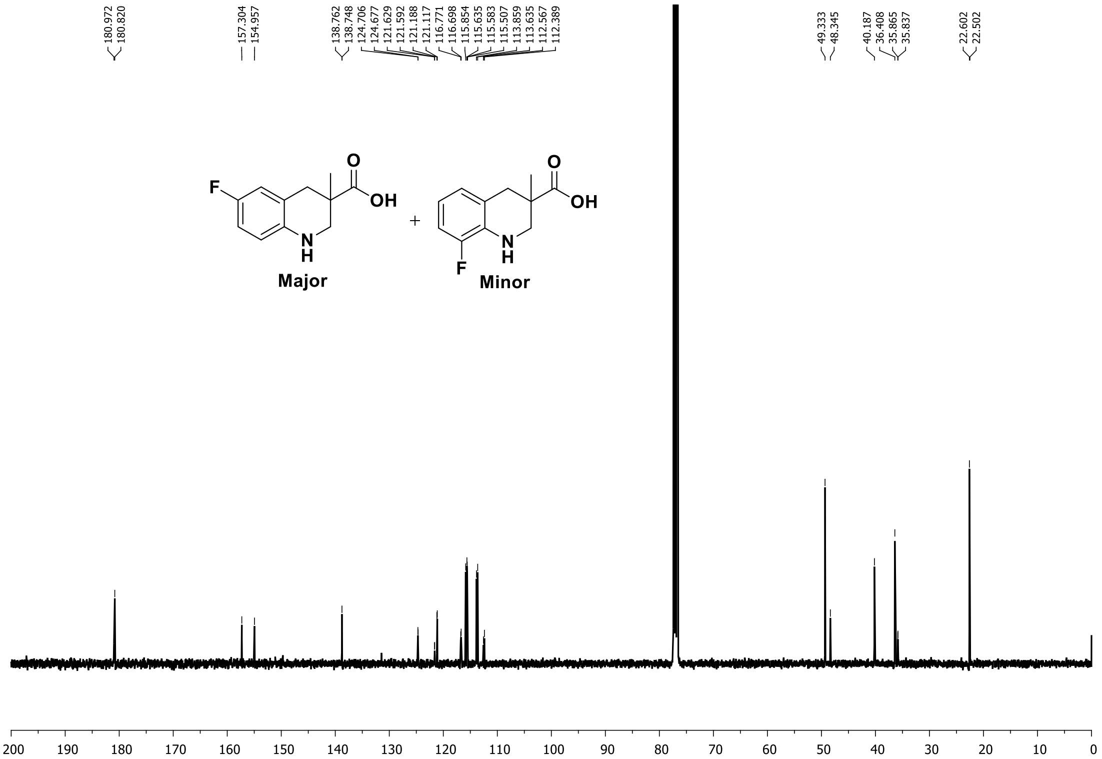

^{19}F NMR (376 MHz, $CDCl_3$)

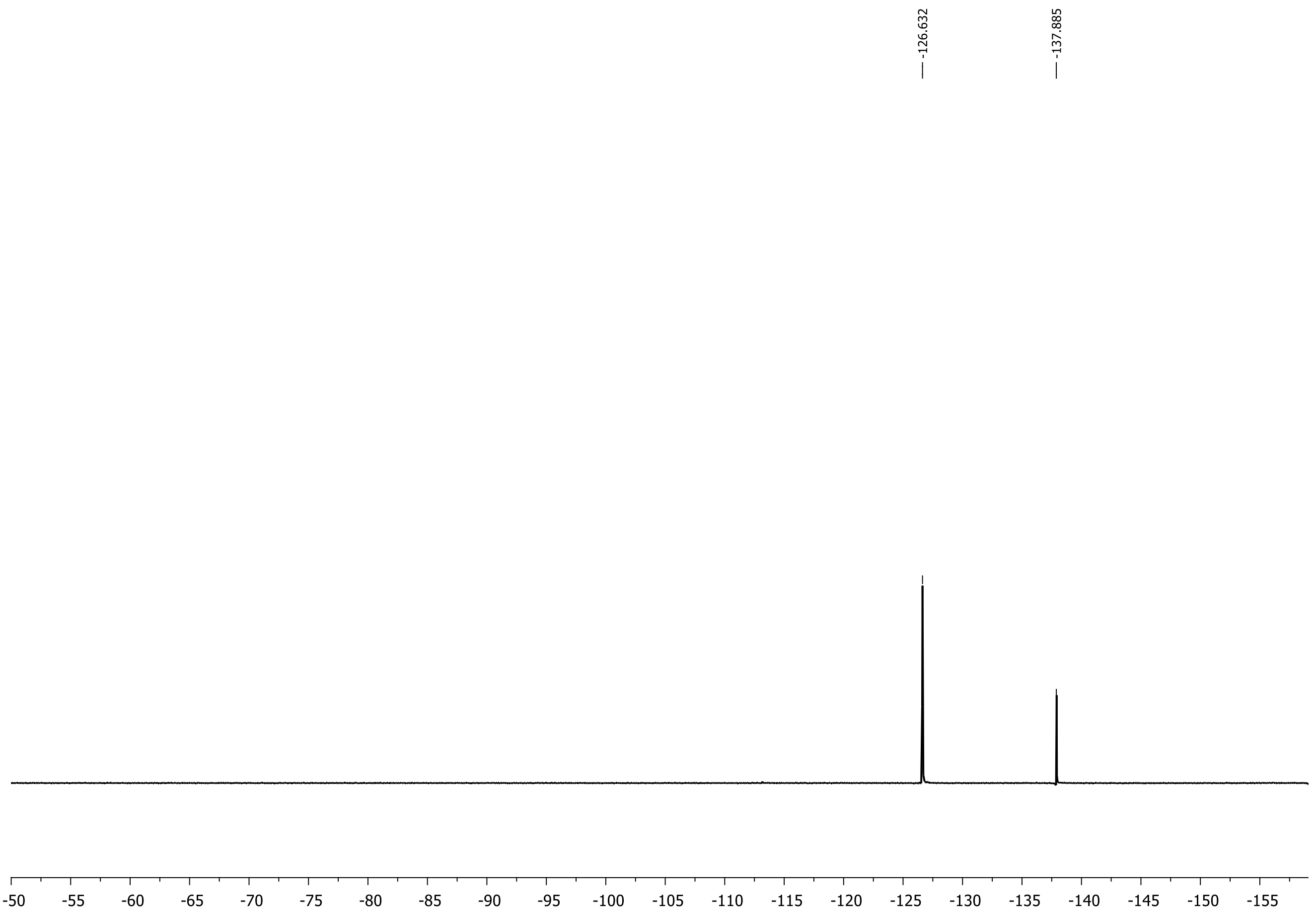

3-Methyl-7-(4,4,5,5-tetramethyl-1,3-dioxolan-2-yl)-1,2,3,4-tetrahydroquinoline-3-carboxylic acid (2q)

^{1}H NMR (400 MHz, $CDCl_3$)

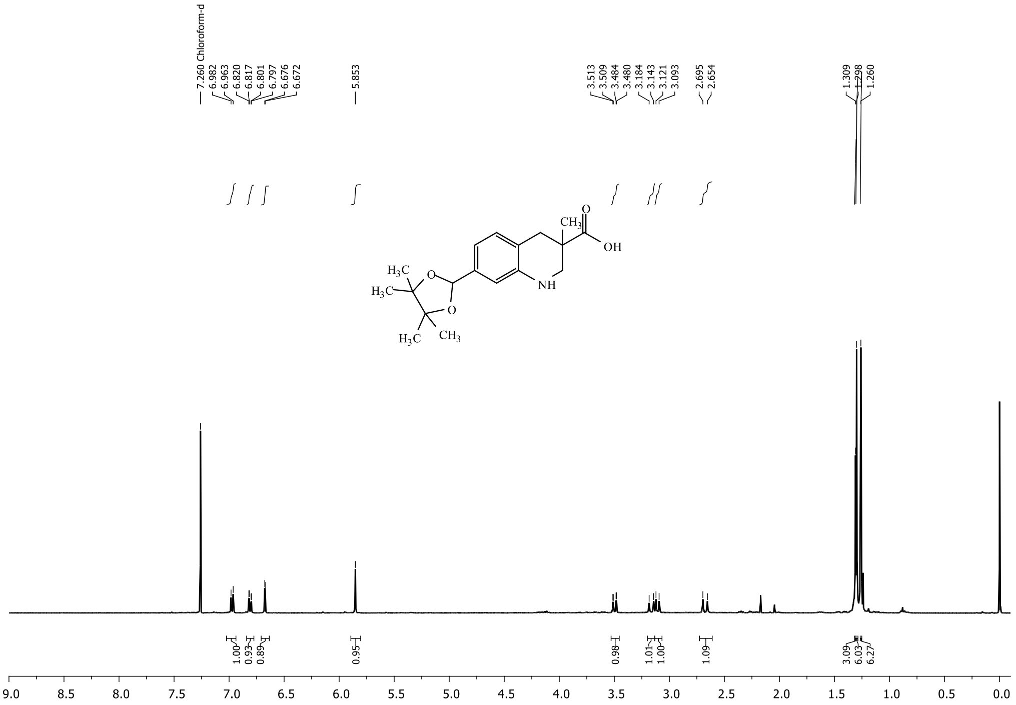

^{13}C NMR (101 MHz, $CDCl_3$)

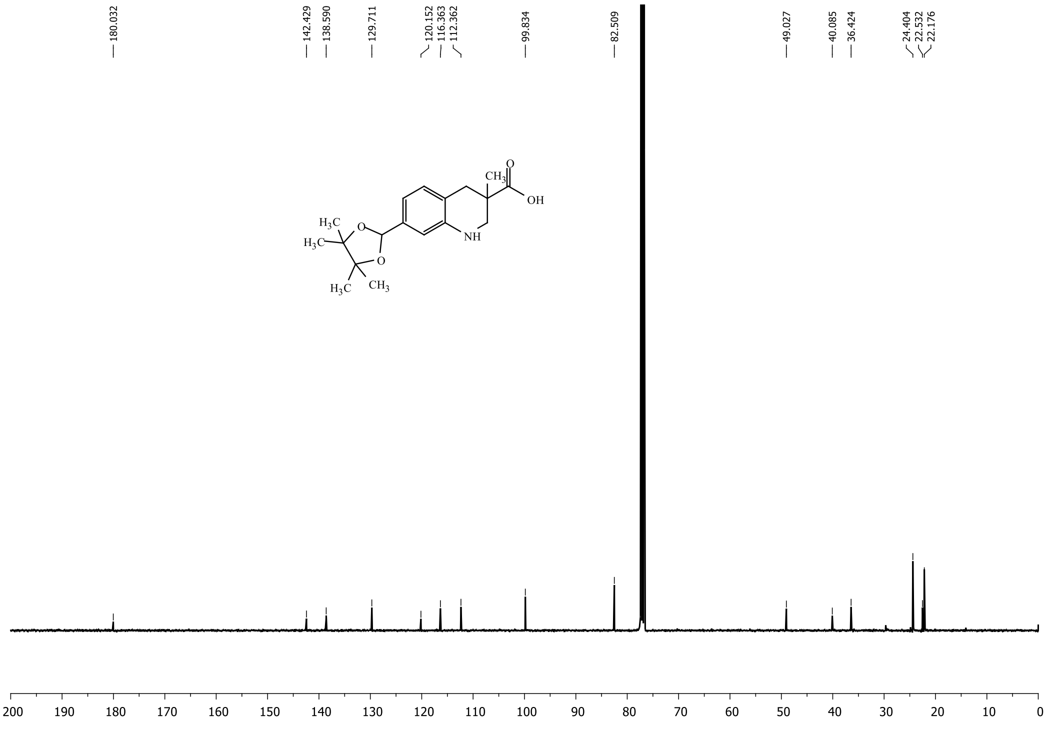

3-Methyl-7-((triisopropylsilyl)ethynyl)-1,2,3,4-tetrahydroquinoline-3-carboxylic acid (2r)

^{1}H NMR (400 MHz, $CDCl_3$)

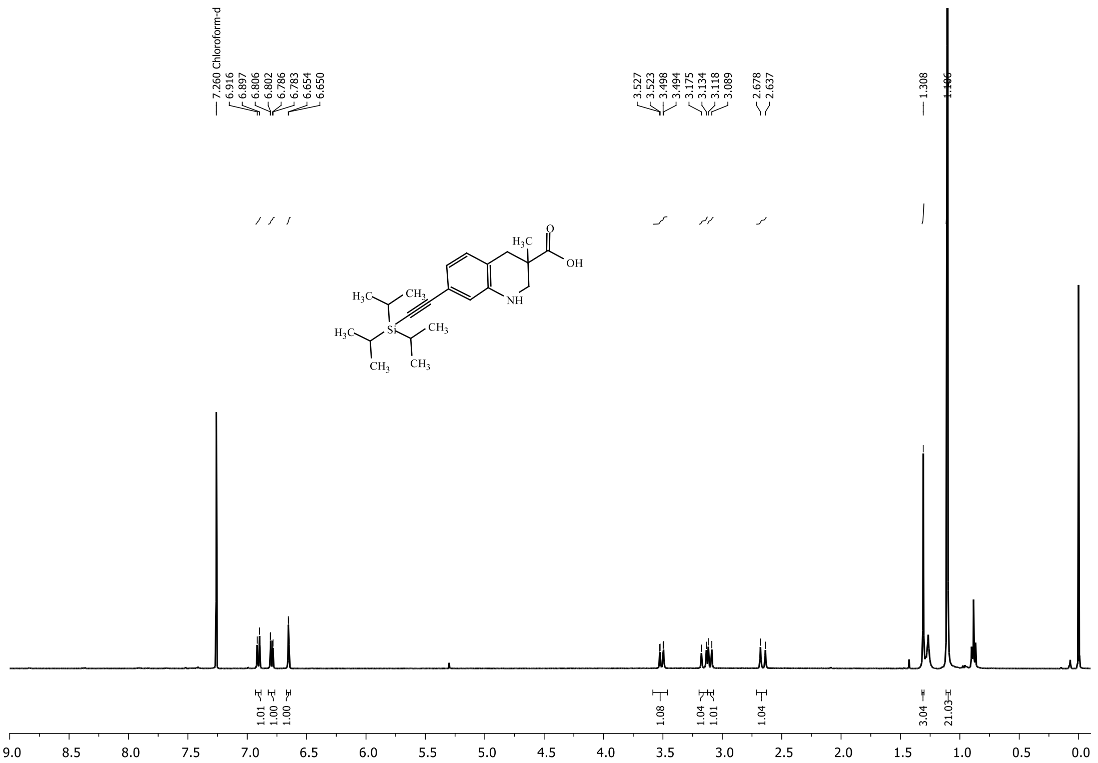

^{13}C NMR (101 MHz, $CDCl_3$)

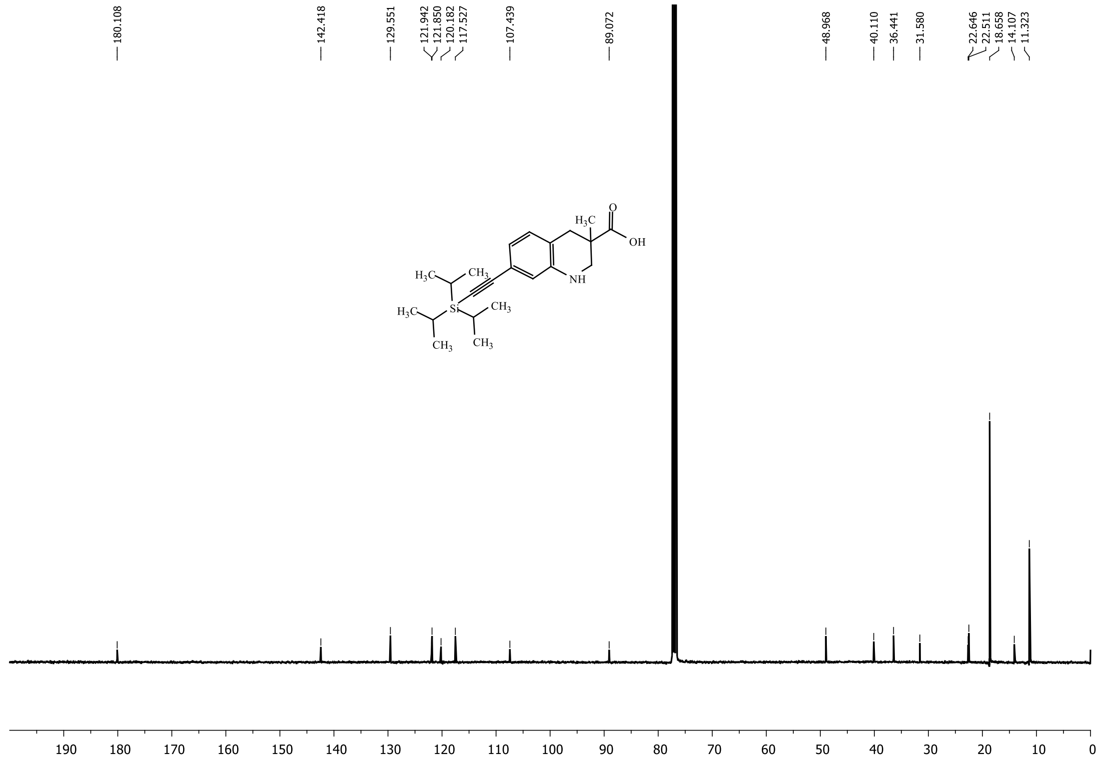

1,2,3,4-Tetrahydrobenzo[g]quinoline-3-carboxylic acid (2s)

^{1}H NMR (400 MHz, $CDCl_3$)

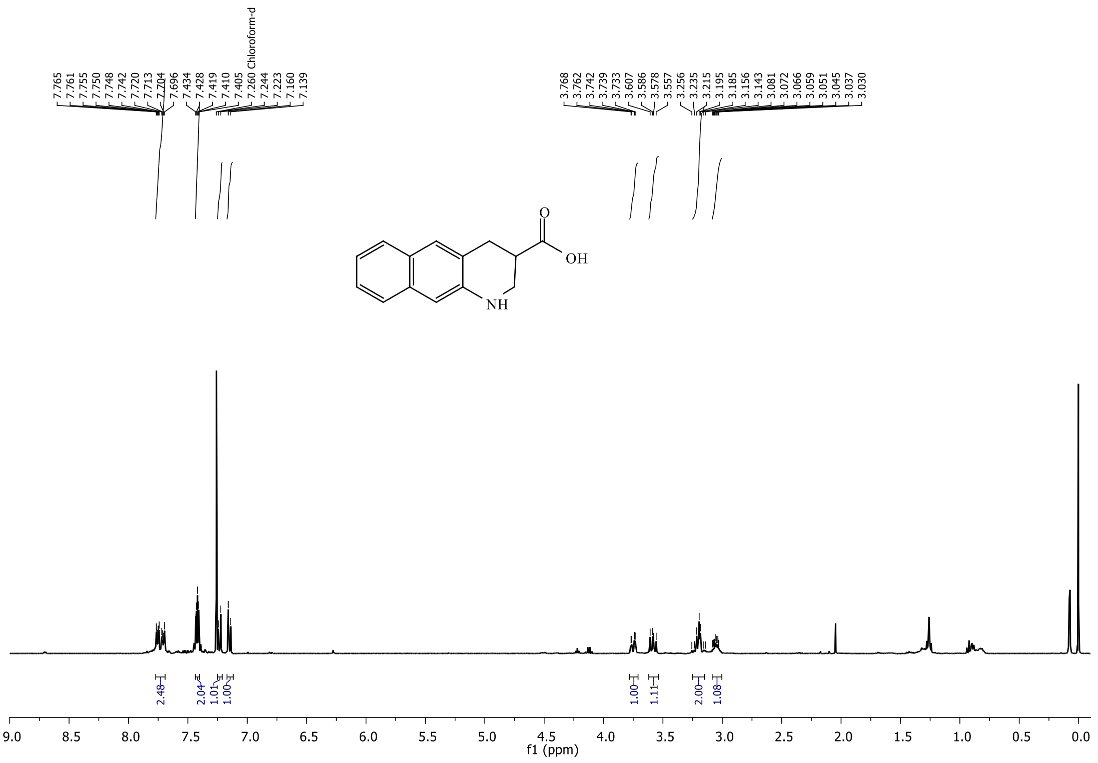

^{13}C NMR (101 MHz, $CDCl_3$)

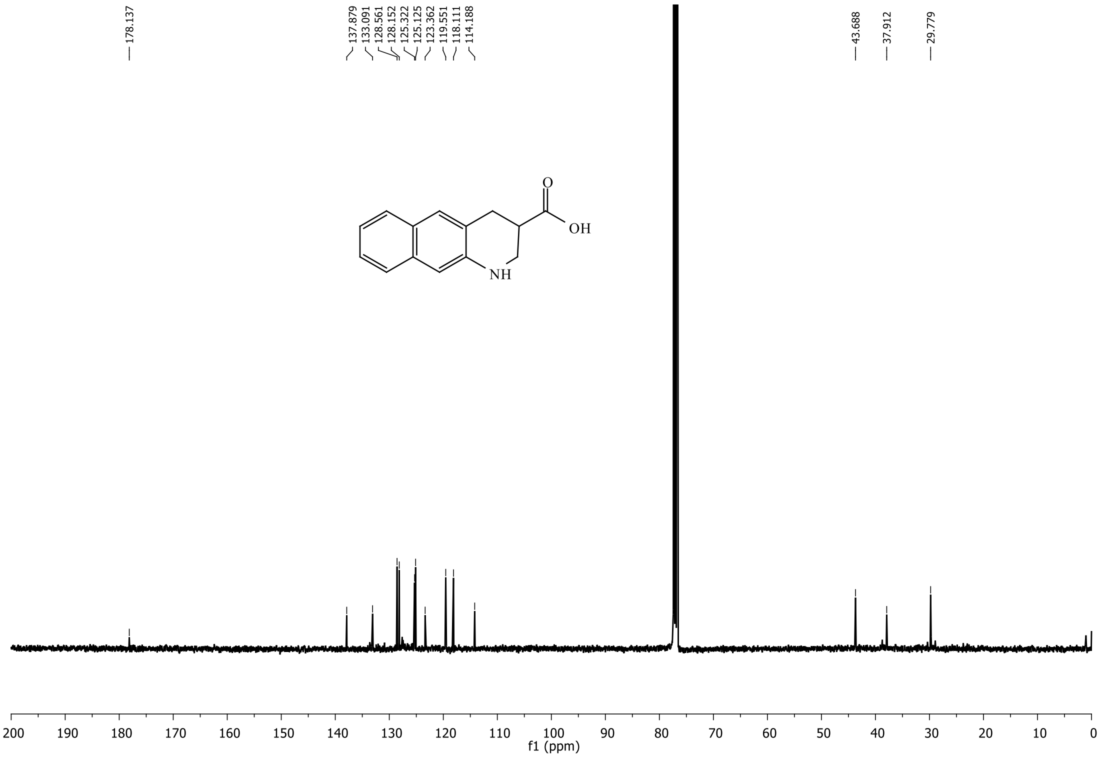

2-(1,2,3,4-Tetrahydroquinolin-2-yl)acetic acid (7)

^{1}H NMR (400 MHz, CDCl$_3$)

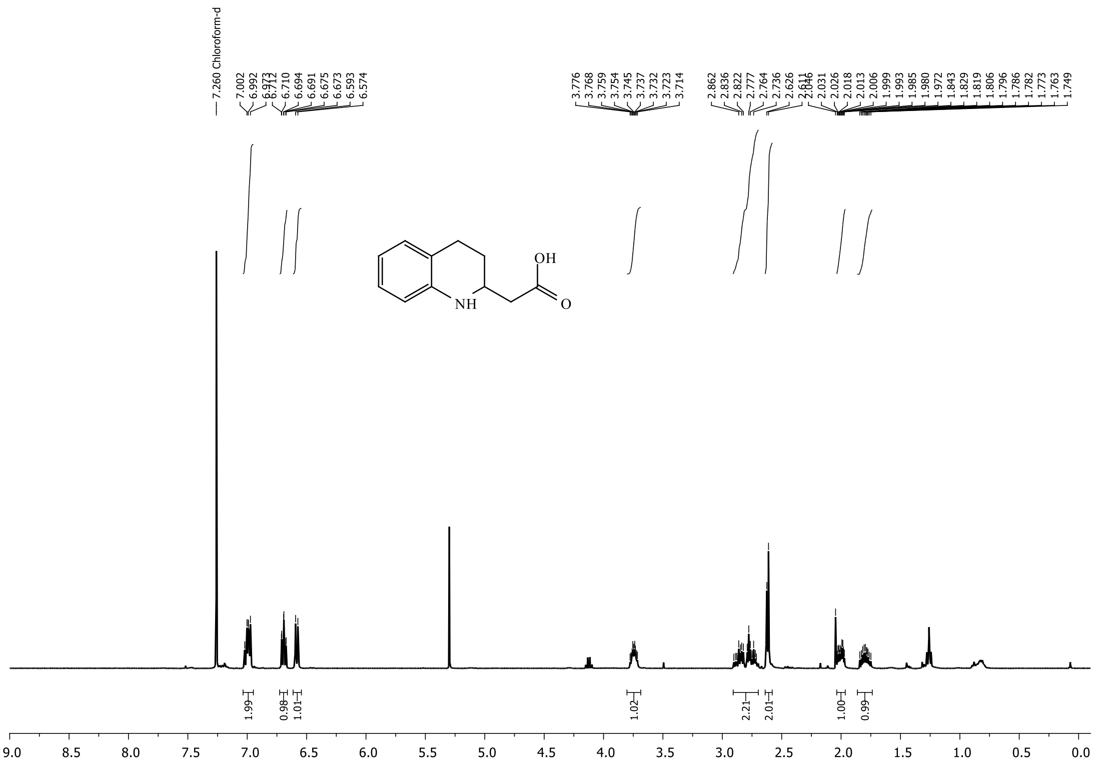

^{13}C NMR (101 MHz, CDCl$_3$)

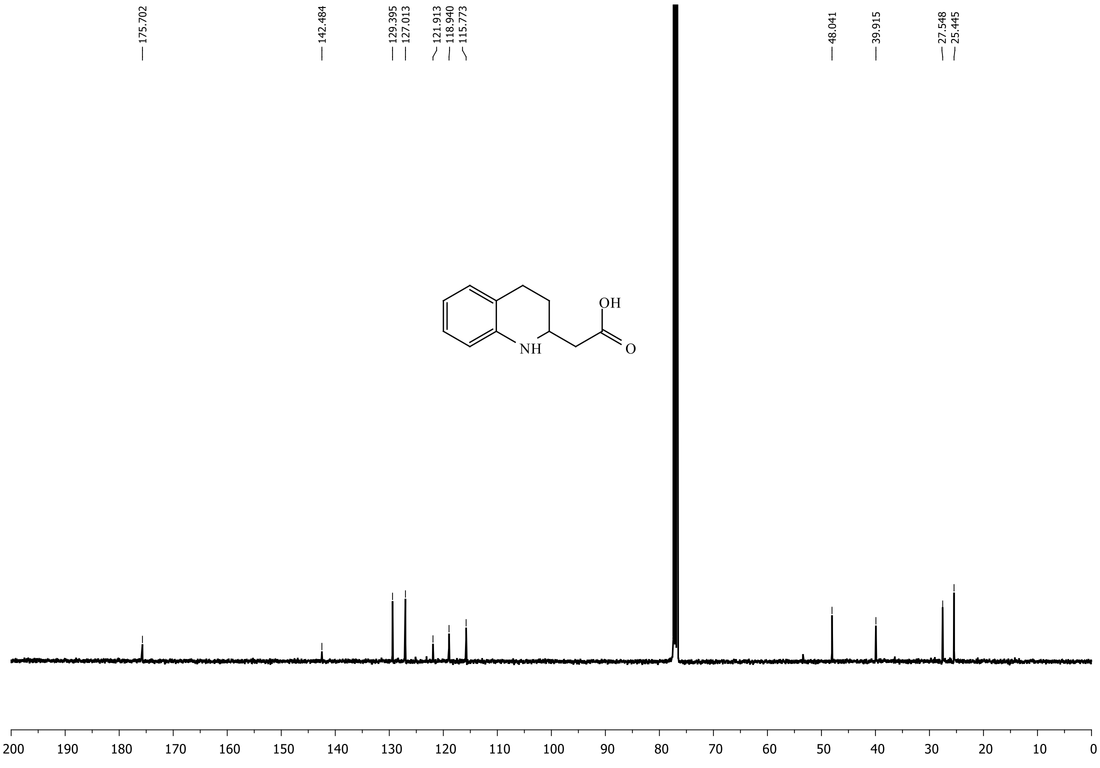

3-Methyl-8-oxo-1-azaspiro[4.5]deca-6,9-dien-1-ium-3-carboxylate (16)

^{1}H NMR (400 MHz, D_2O)

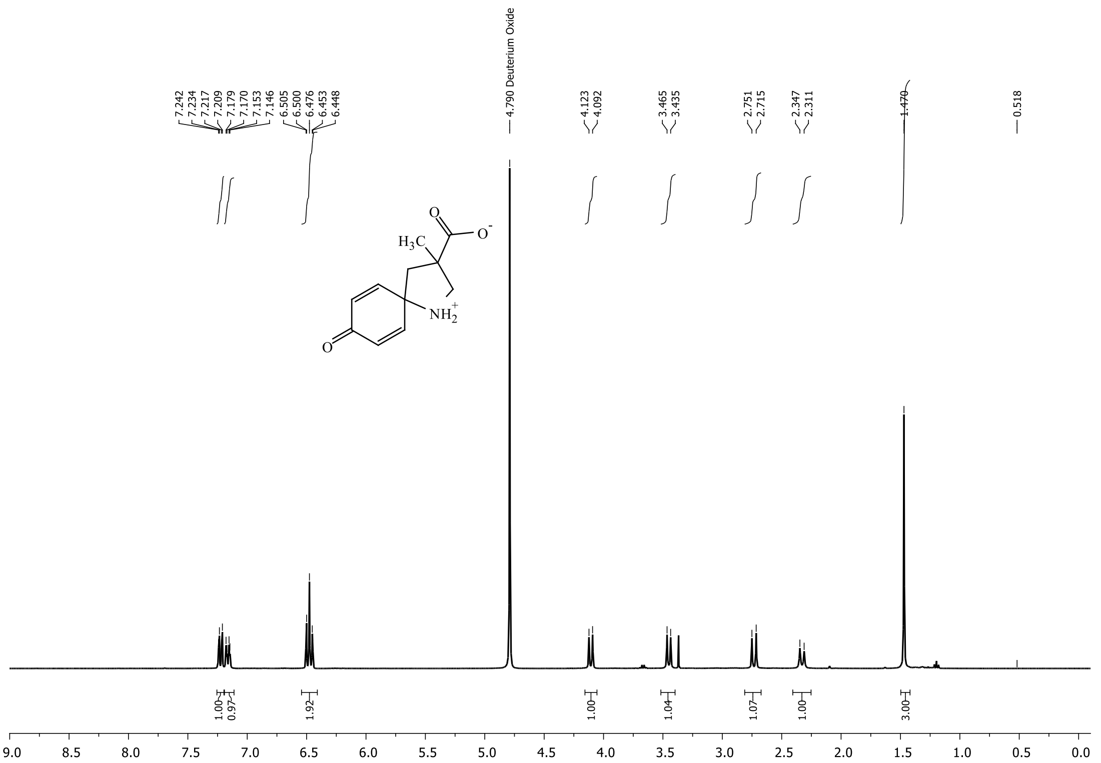

^{13}C NMR (151 MHz, DMSO-d_6)

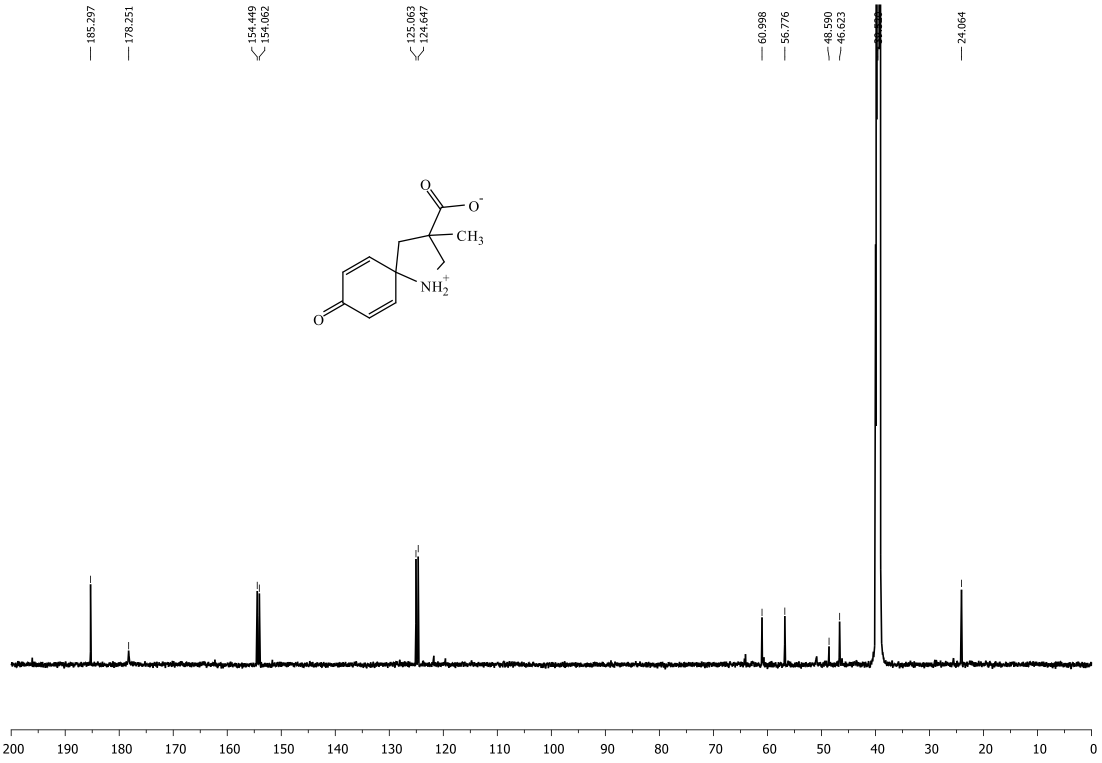

7-Hydroxy-3-methyl-1,2,3,4-tetrahydroquinoline-3-carboxylic acid (17)

^{1}H NMR (600 MHz, CD_3OD)

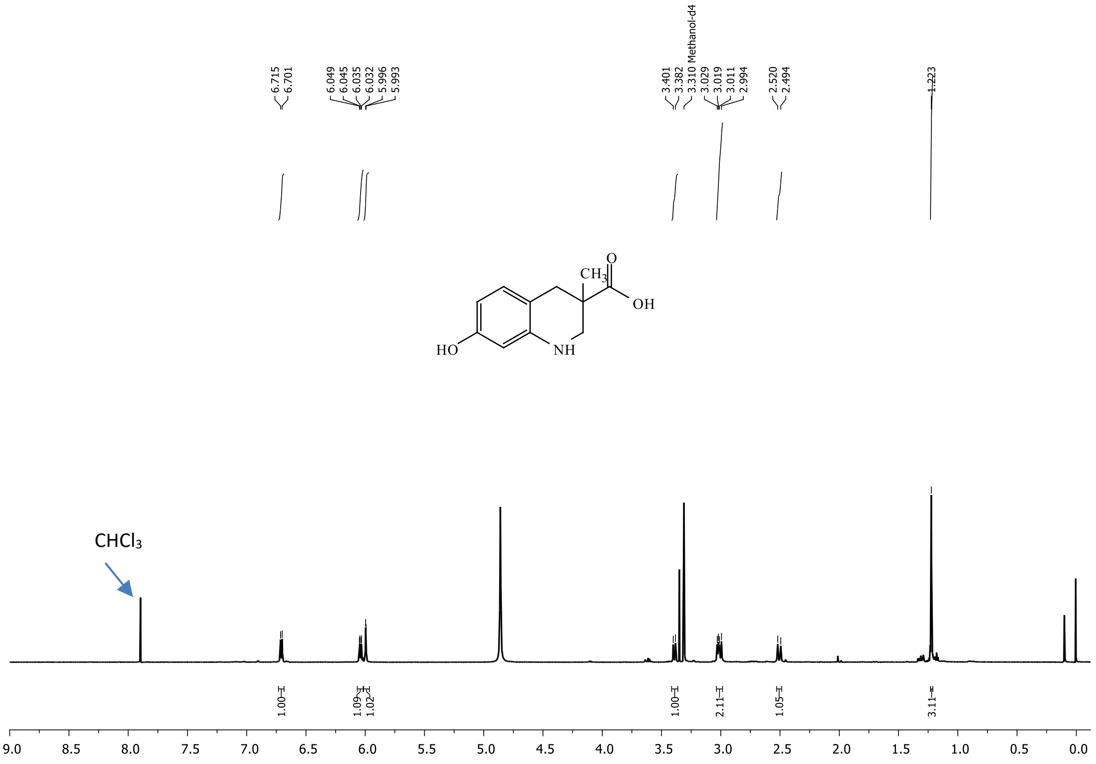

^{13}C NMR (151 MHz, CD_3OD)

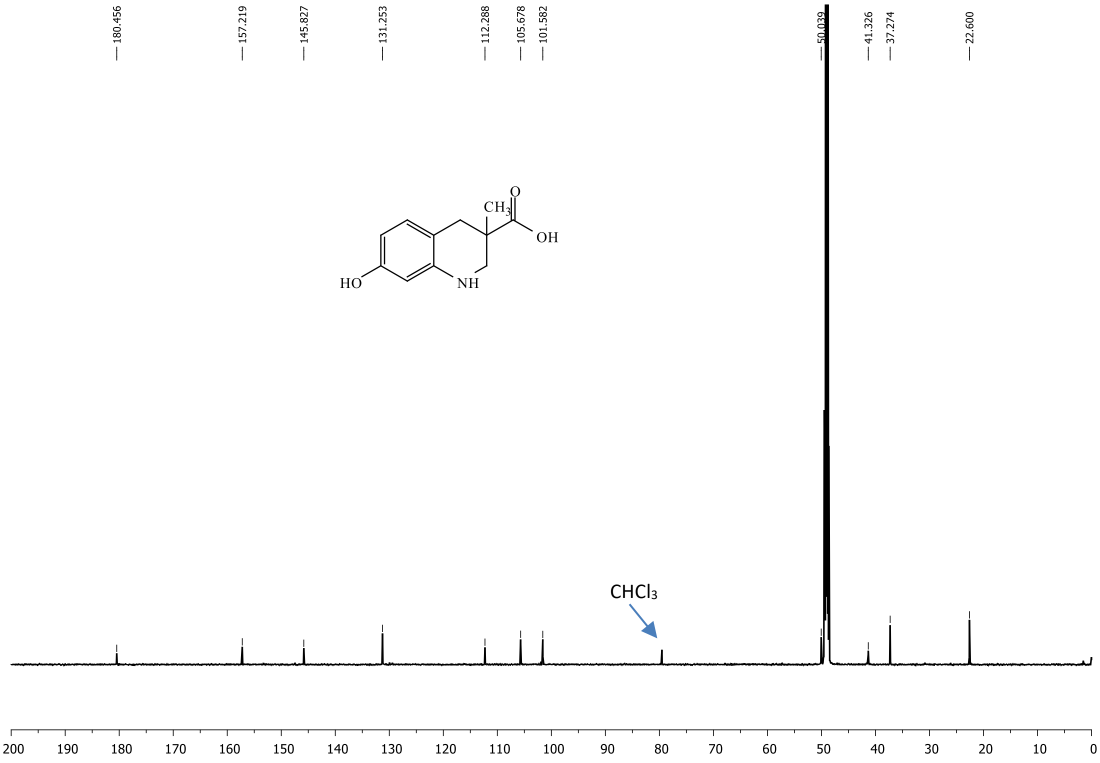

DEPT 135

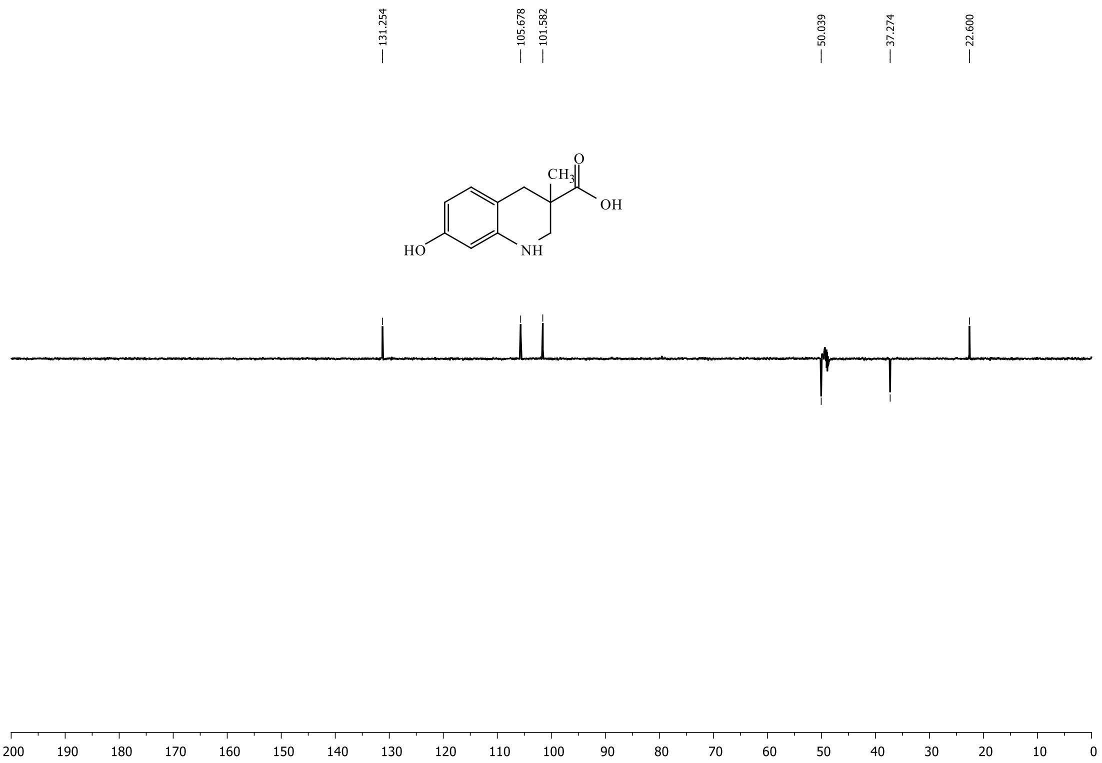
131.254
105.678
101.582
50.039
37.274
22.600
CH3
O
OH
HO
NH
200
190
180
170
160
150
140
130
120
110
100
90
80
70
60
50
40
30
20
10
0

5-(4-Hydroxyphenyl)-3-methylpyrrolidine-3-carboxylic acid (19)

^{1}H NMR (400 MHz, D_2O)

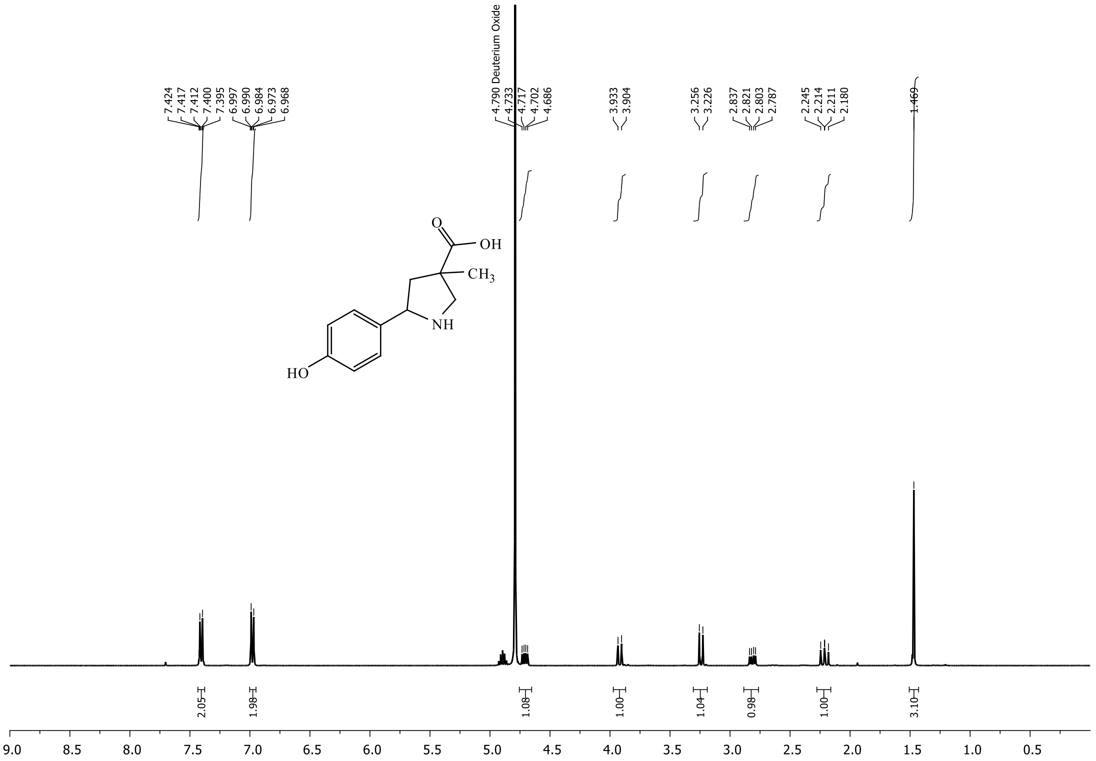

^{13}C NMR (101 MHz, D_2O)

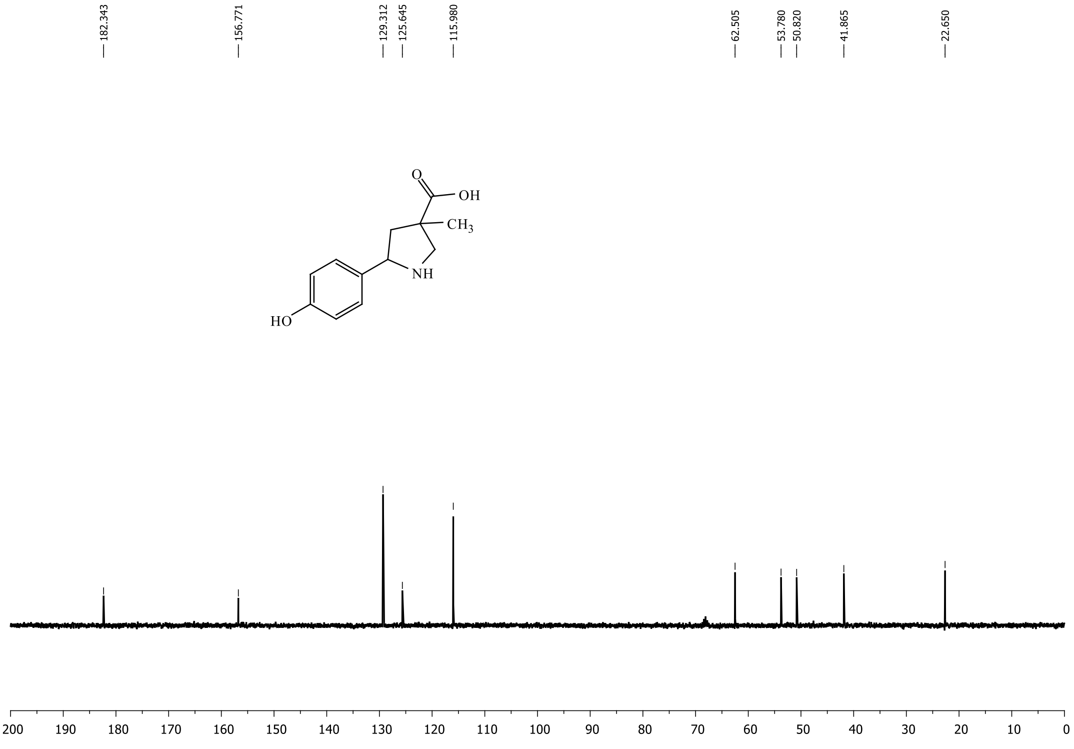

3-Methyl-9-oxo-1-azaspiro[5.5]undeca-7,10-dien-1-ium-3-carboxylate (20)

^{1}H NMR (400 MHz, D_2O)

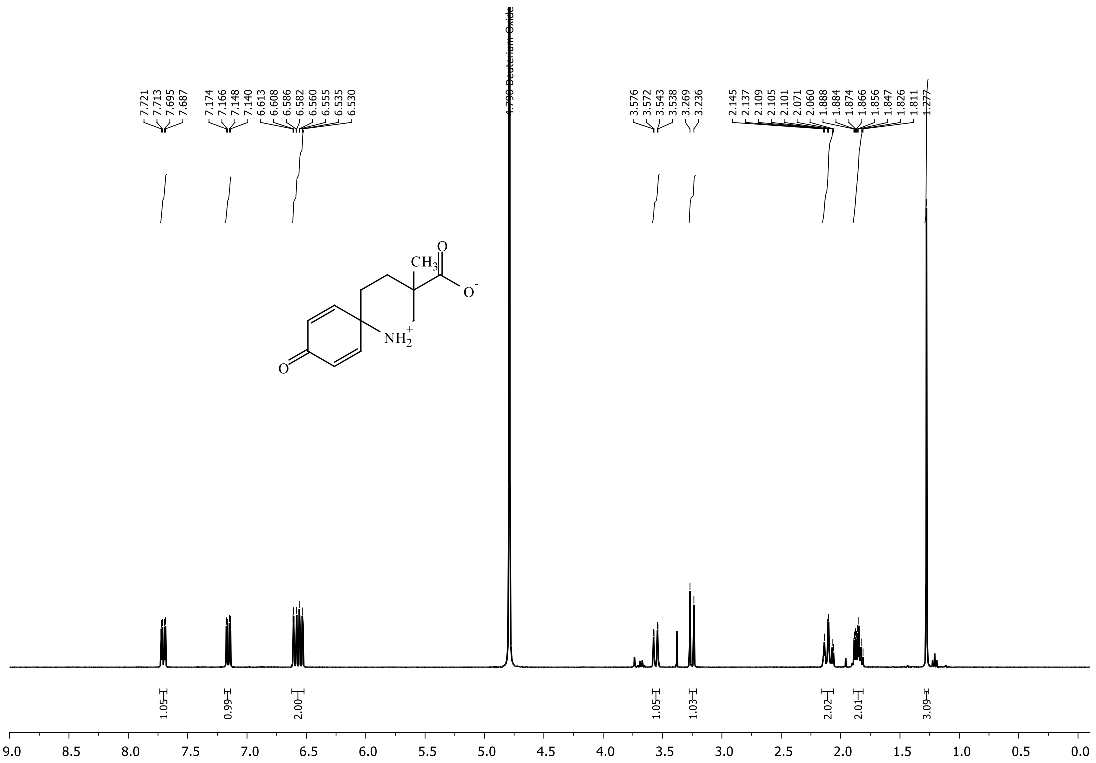

^{13}C NMR (101 MHz, D_2O)

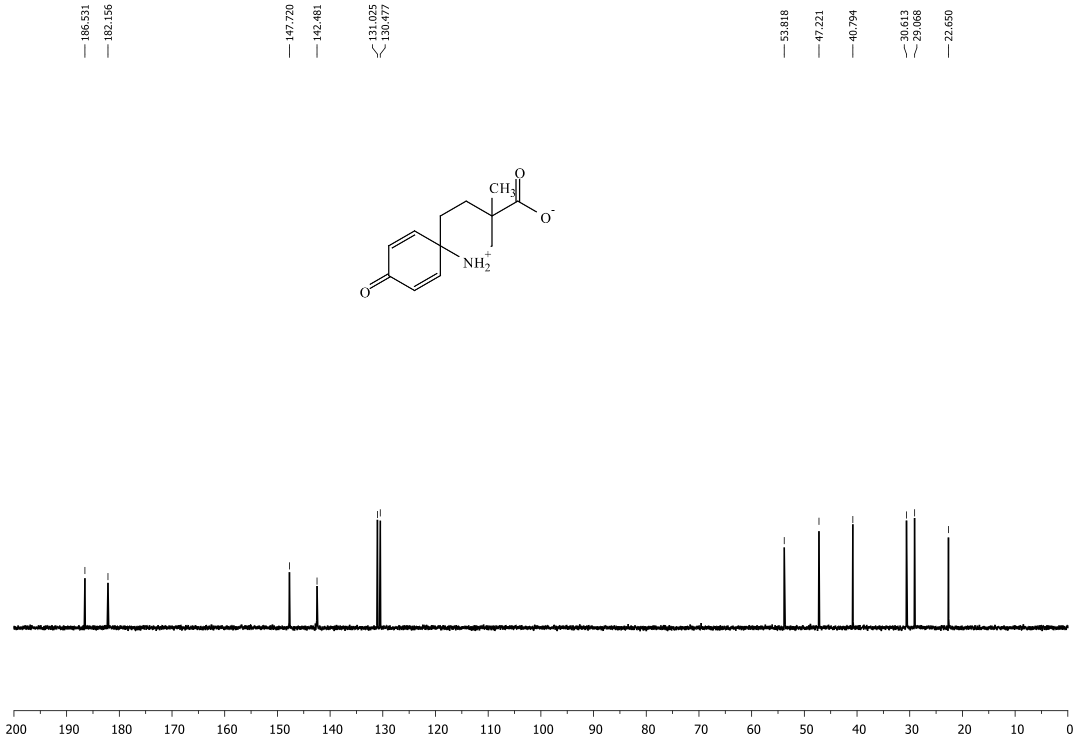

DEPT 90

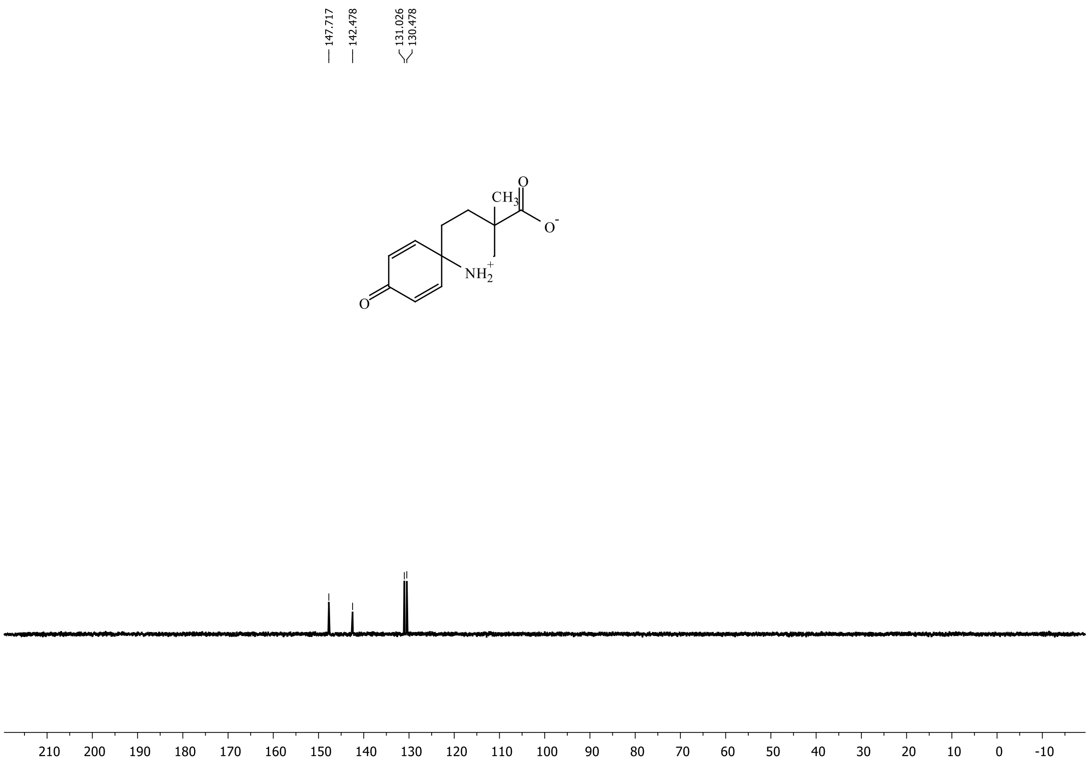

DEPT 135

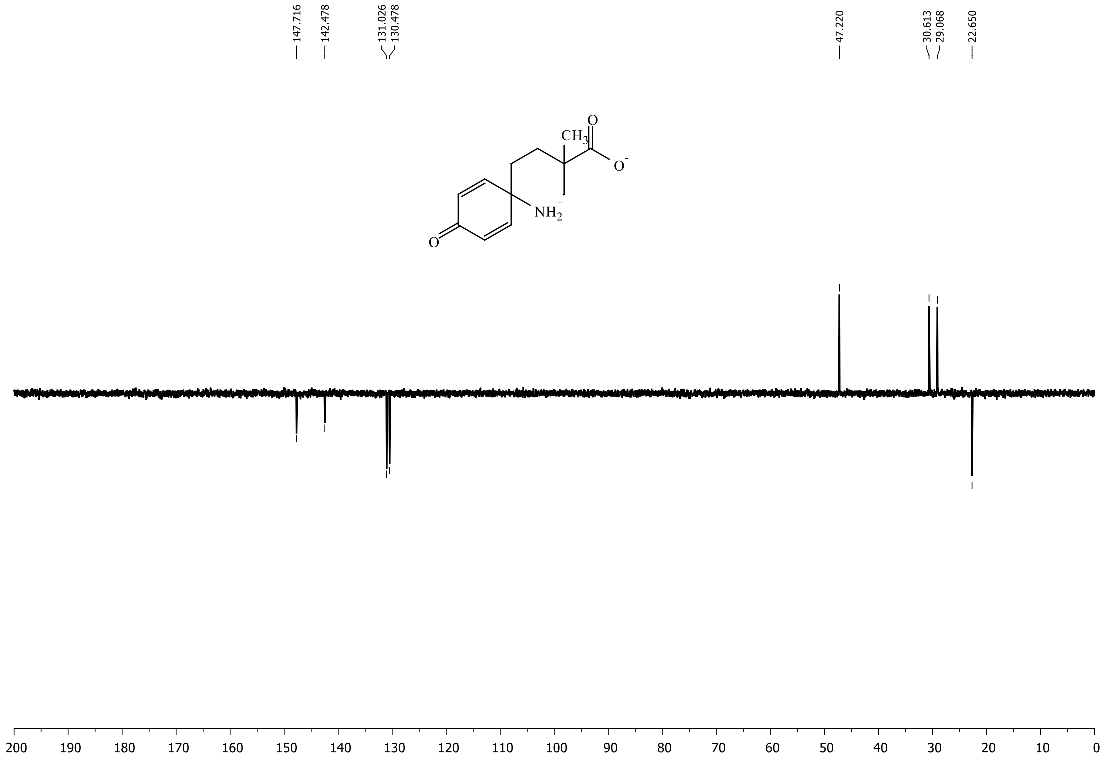

(2*R*,3*R*)-3-Benzyl-2-phenyl-1,2,3,4-tetrahydroquinoline-3-carboxylic acid (14)

^{1}H NMR (400 MHz, $CDCl_3$)

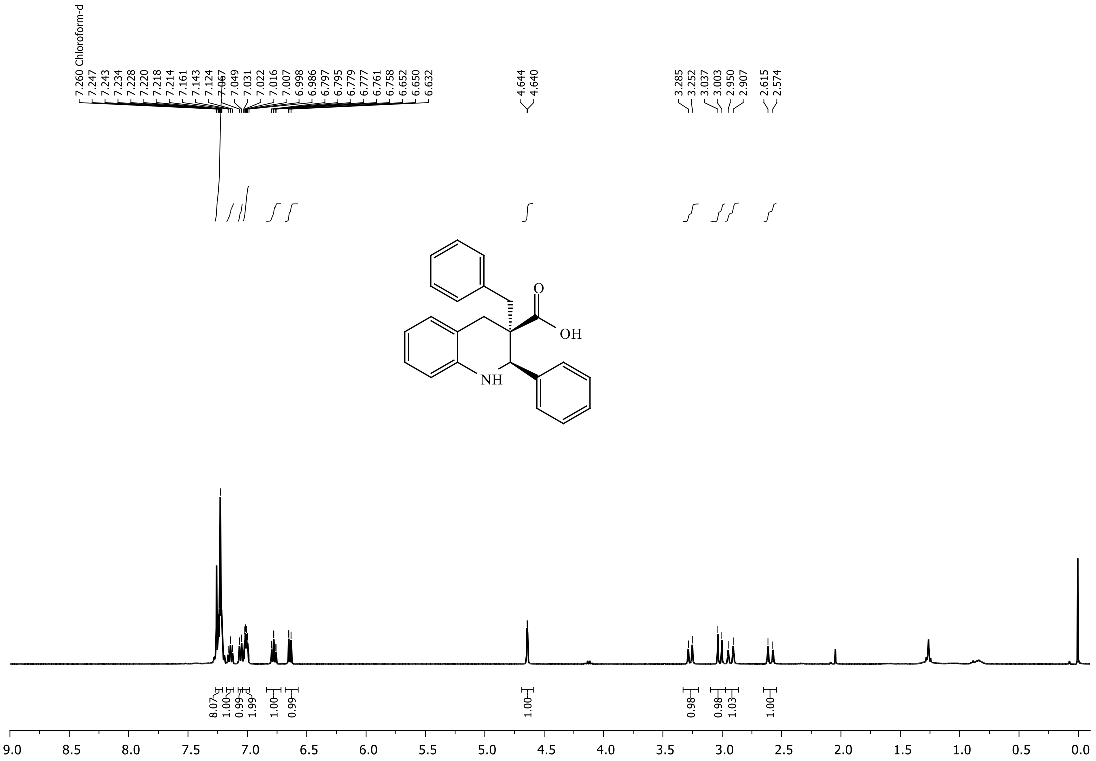

^{13}C NMR (101 MHz, $CDCl_3$)

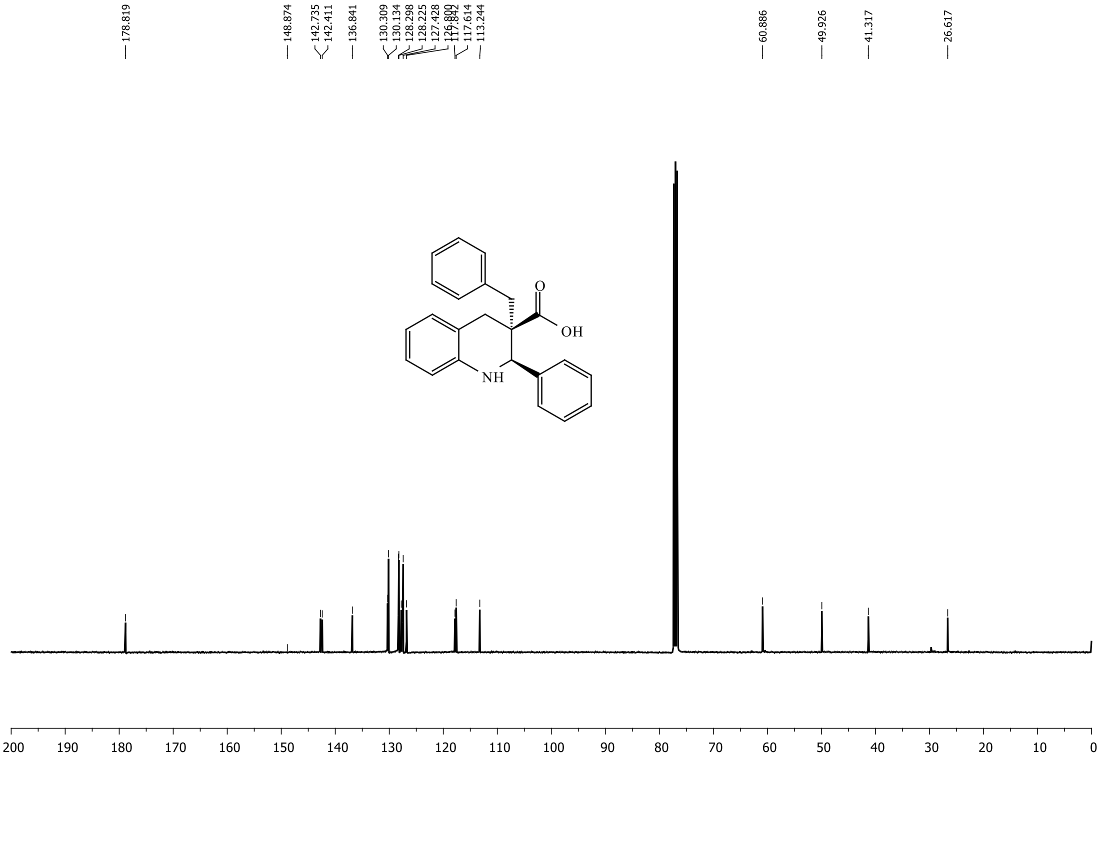

HSQC

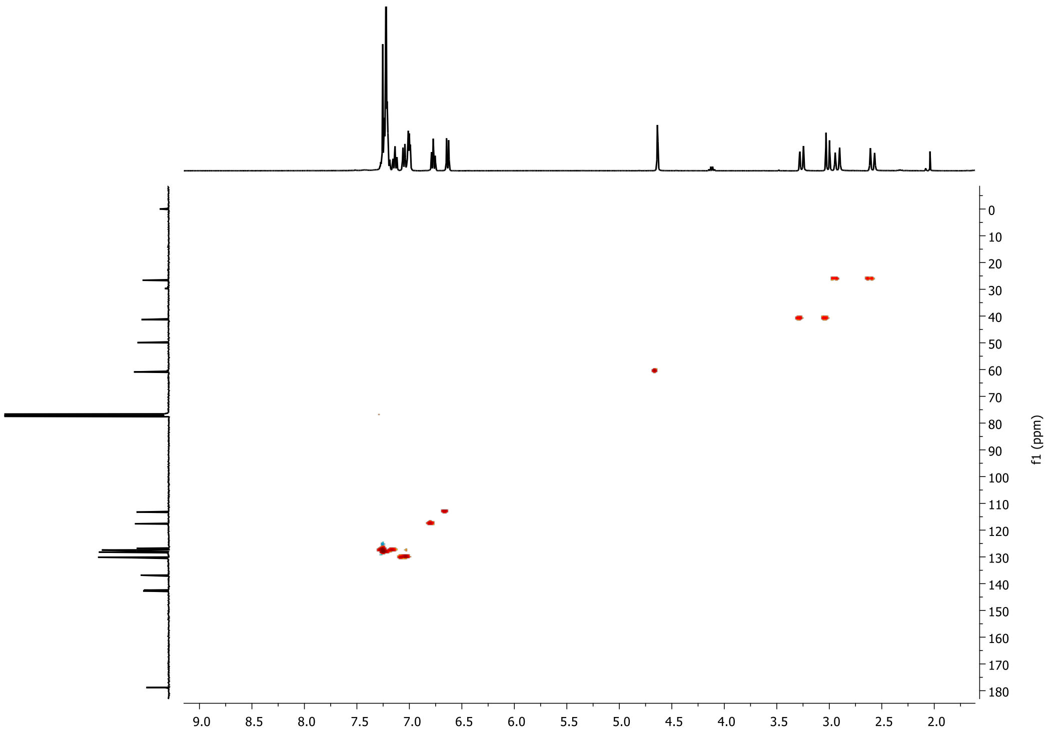
f1 (ppm)
0
10
20
30
40
50
60
70
80
90
100
110
120
130
140
150
160
170
180
9.0
8.5
8.0
7.5
7.0
6.5
6.0
5.5
5.0
4.5
4.0
3.5
3.0
2.5
2.0

NOE

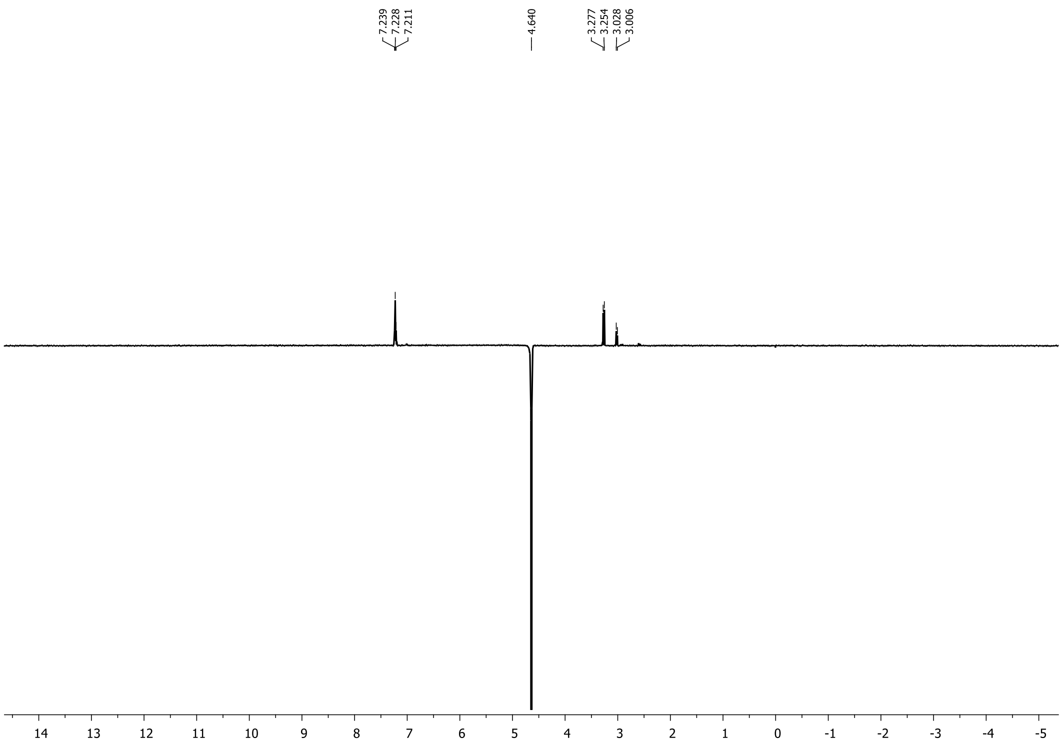
7.239
7.228
7.211
4.640
3.277
3.254
3.028
3.006
14
13
12
11
10
9
8
7
6
5
4
3
2
1
0
-1
-2
-3
-4
-5

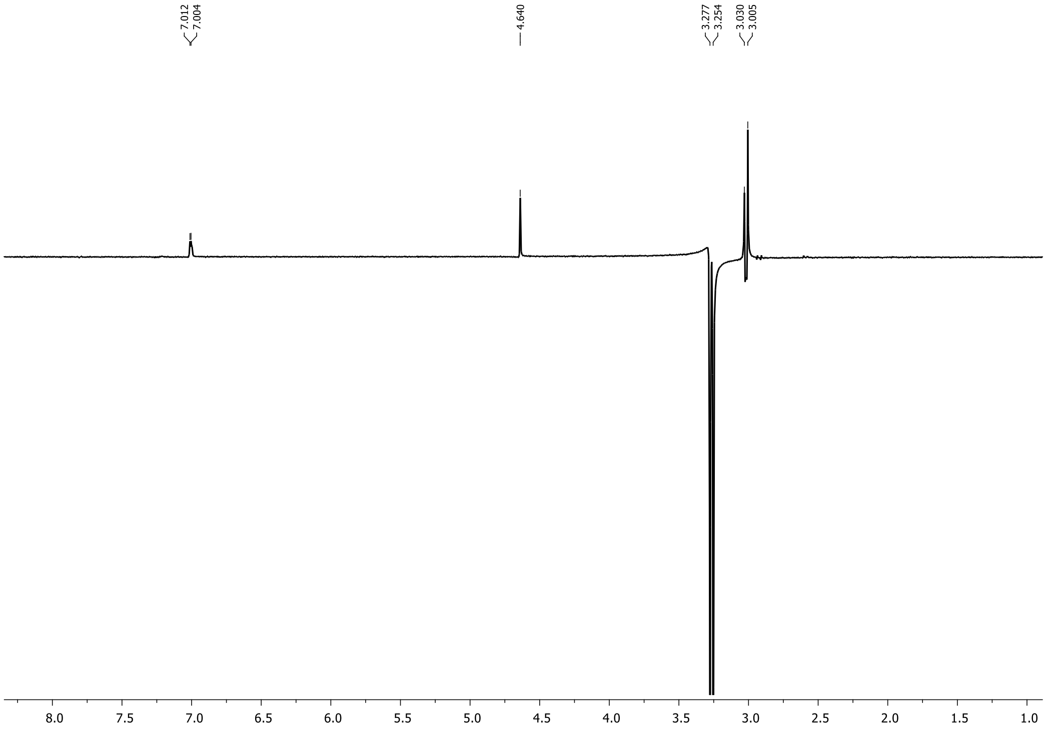
7.012
7.004
4.640
3.277
3.254
3.030
3.005
8.0
7.5
7.0
6.5
6.0
5.5
5.0
4.5
4.0
3.5
3.0
2.5
2.0
1.5
1.0

(*R*)-3-Benzyl-1,2,3,4-tetrahydroquinoline-3-carboxylic acid (S5)

^{1}H NMR (400 MHz, $CDCl_3$)

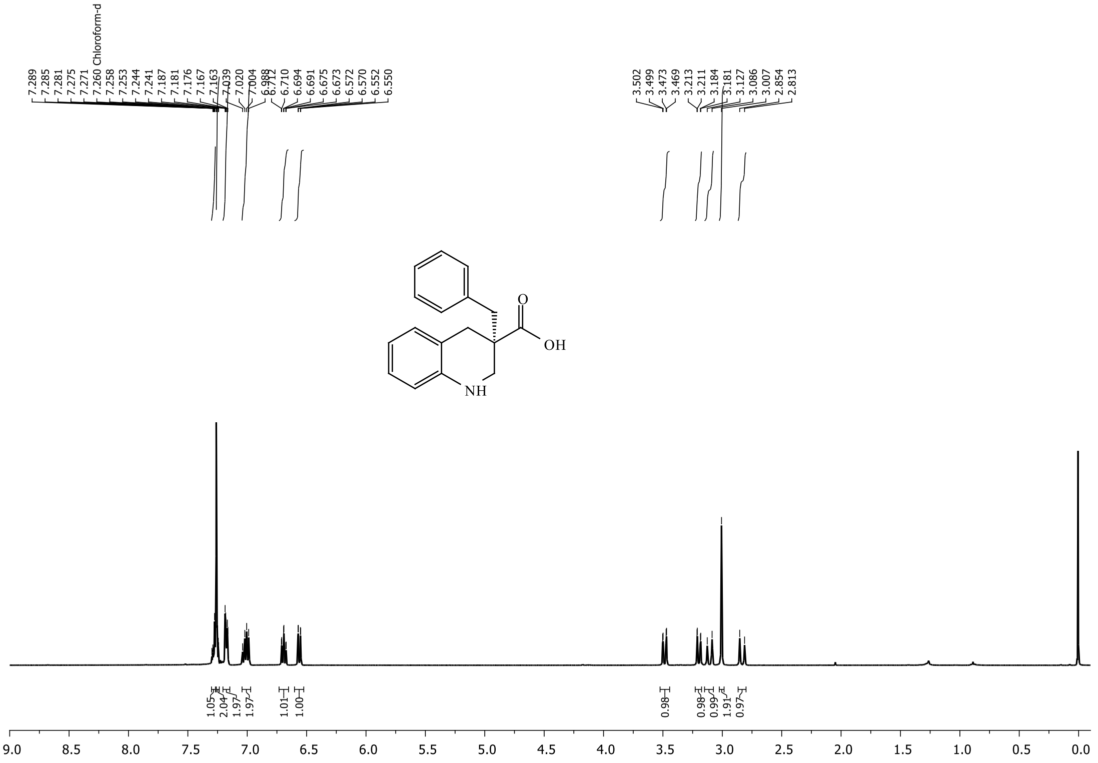

^{13}C NMR (101 MHz, $CDCl_3$)

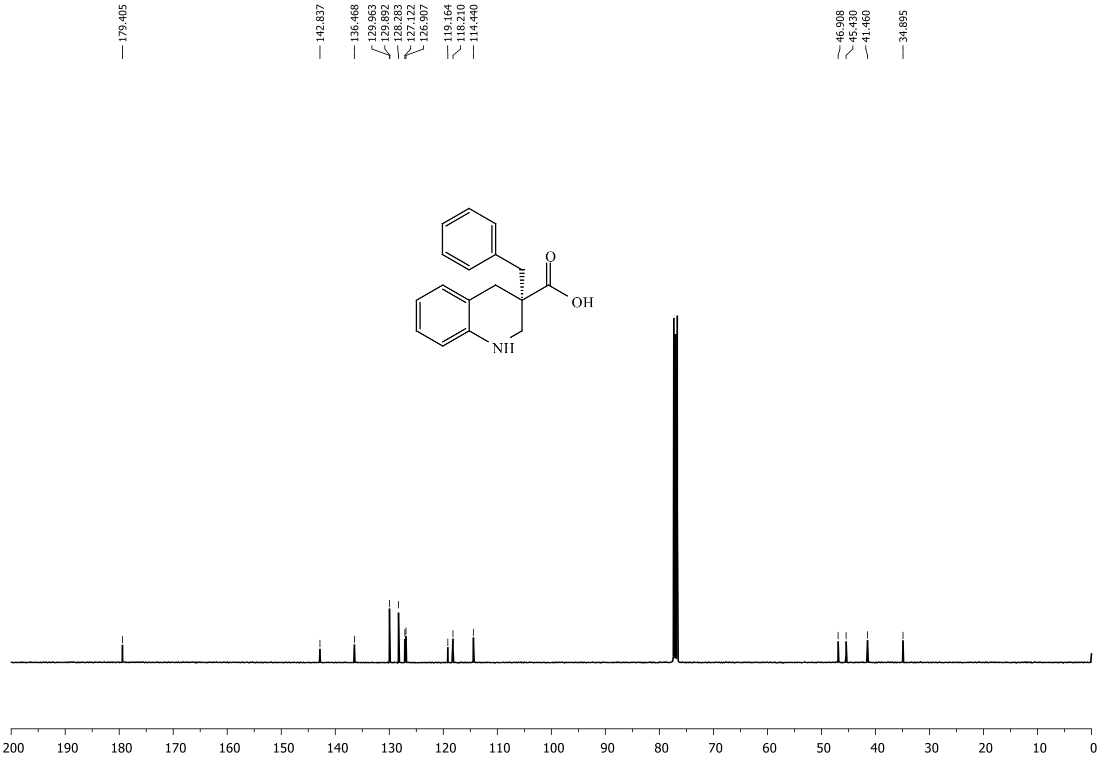

Methyl (*R*)-3-benzyl-1,2,3,4-tetrahydroquinoline-3-carboxylate (9)

^{1}H NMR (400 MHz, $CDCl_3$)

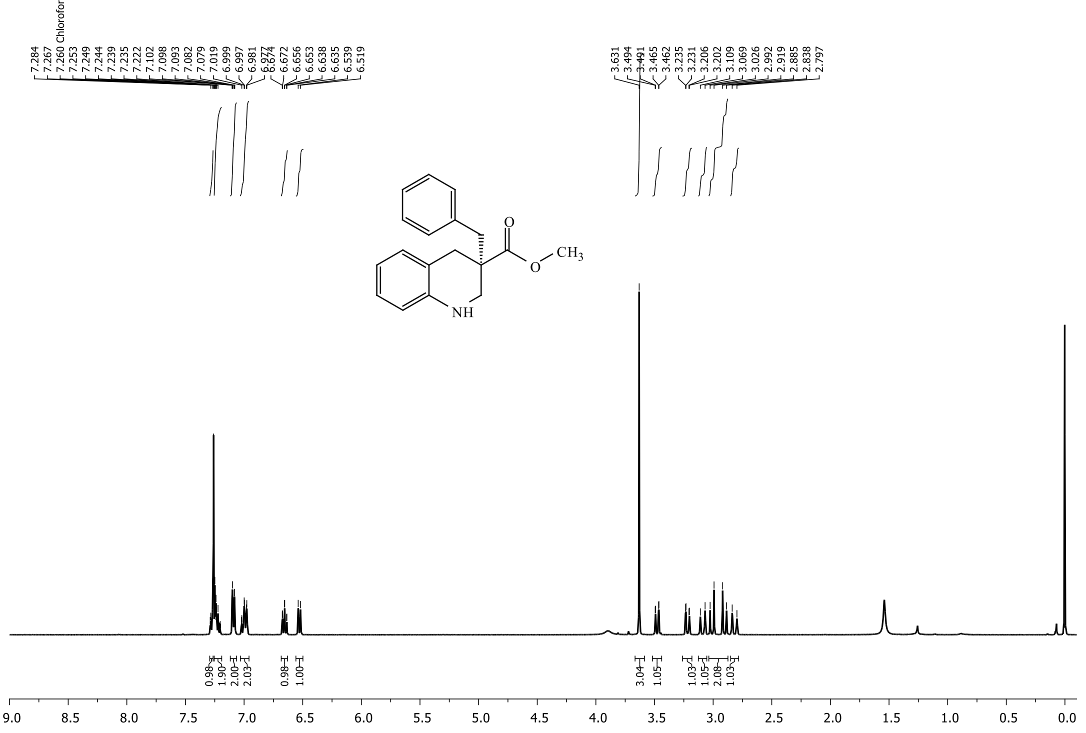

^{13}C NMR (101 MHz, $CDCl_3$)

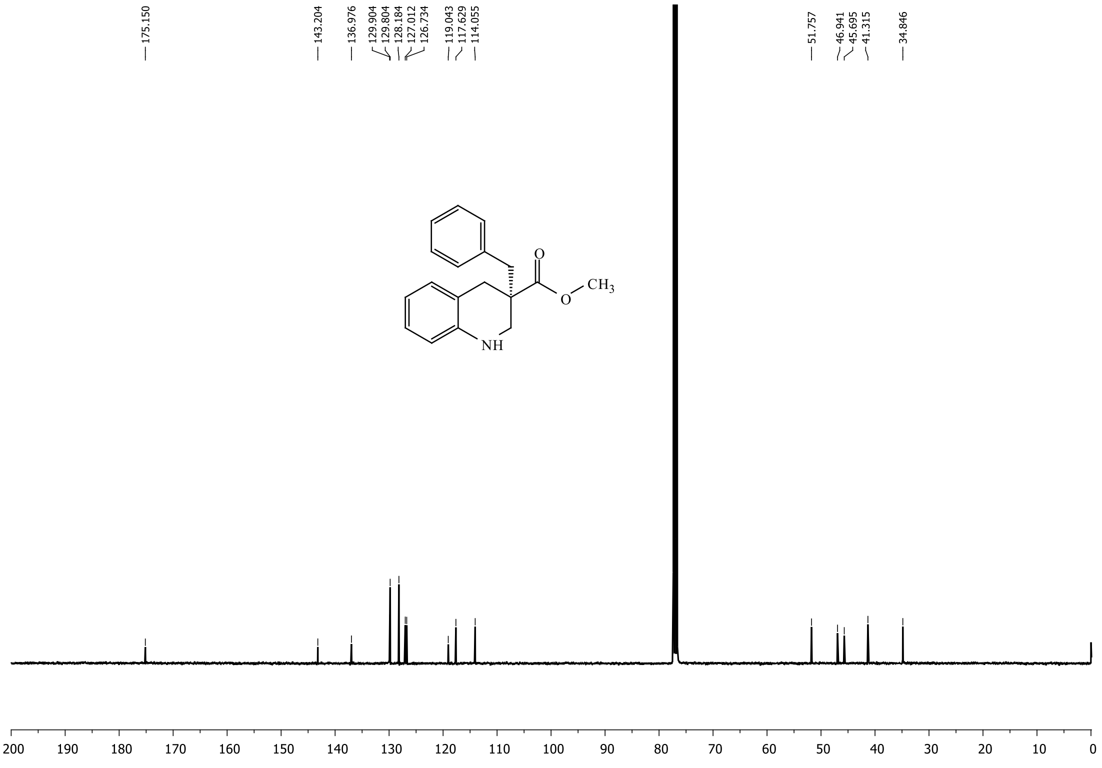

^{13}C-Acetophenone

^{1}H NMR (400 MHz, $CDCl_3$)

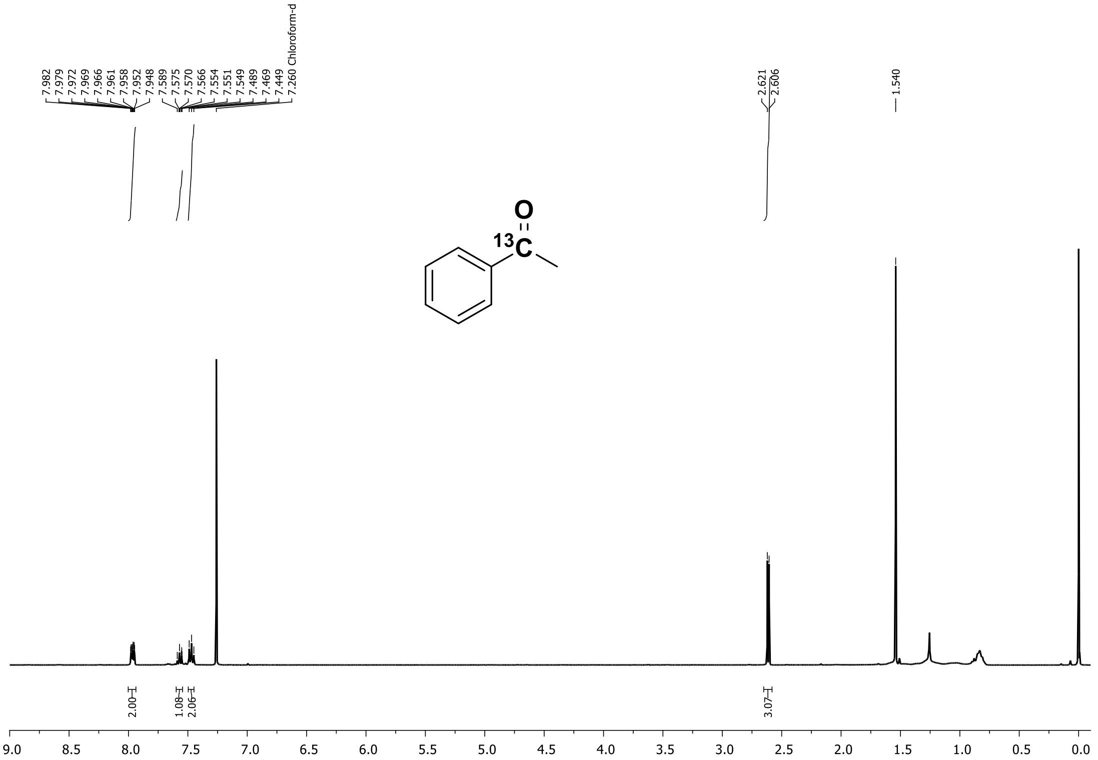

^{13}C NMR (101 MHz, $CDCl_3$)

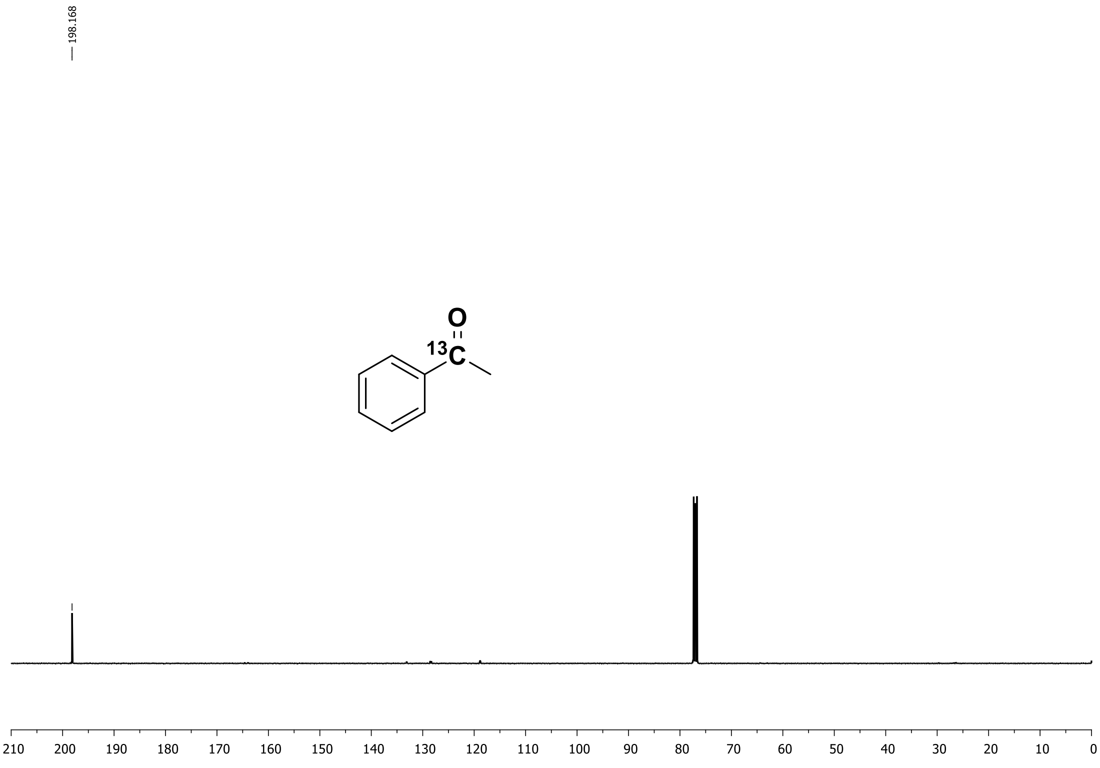

Supplement: SC-012-D1SC01419F-s001 [file SC-012-D1SC01419F-s001.pdf]
